# Comparative maternal protein profiling of mouse biparental and uniparental embryos

**DOI:** 10.1093/gigascience/giac084

**Published:** 2022-09-03

**Authors:** Fumei Chen, Buguo Ma, Yongda Lin, Xin Luo, Tao Xu, Yuan Zhang, Fang Chen, Yanfei Li, Yaoyao Zhang, Bin Luo, Qingmei Zhang, Xiaoxun Xie

**Affiliations:** Department of Histology and Embryology, School of Pre-Clinical Medicine, Guangxi Medical University, Nanning, Guangxi 530021, P. R. China; Department of Histology and Embryology, School of Pre-Clinical Medicine, Guangxi Medical University, Nanning, Guangxi 530021, P. R. China; Central Laboratory, School of Pre-Clinical Medicine, Guangxi Medical University, Nanning, Guangxi 530021, P. R. China; Department of Histology and Embryology, School of Pre-Clinical Medicine, Guangxi Medical University, Nanning, Guangxi 530021, P. R. China; Central Laboratory, School of Pre-Clinical Medicine, Guangxi Medical University, Nanning, Guangxi 530021, P. R. China; Department of Histology and Embryology, School of Pre-Clinical Medicine, Guangxi Medical University, Nanning, Guangxi 530021, P. R. China; Department of Histology and Embryology, School of Pre-Clinical Medicine, Guangxi Medical University, Nanning, Guangxi 530021, P. R. China; Department of Histology and Embryology, School of Pre-Clinical Medicine, Guangxi Medical University, Nanning, Guangxi 530021, P. R. China; Department of Histology and Embryology, School of Pre-Clinical Medicine, Guangxi Medical University, Nanning, Guangxi 530021, P. R. China; Central Laboratory, School of Pre-Clinical Medicine, Guangxi Medical University, Nanning, Guangxi 530021, P. R. China; Department of Histology and Embryology, School of Pre-Clinical Medicine, Guangxi Medical University, Nanning, Guangxi 530021, P. R. China; Central Laboratory, School of Pre-Clinical Medicine, Guangxi Medical University, Nanning, Guangxi 530021, P. R. China; Department of Histology and Embryology, School of Pre-Clinical Medicine, Guangxi Medical University, Nanning, Guangxi 530021, P. R. China; Central Laboratory, School of Pre-Clinical Medicine, Guangxi Medical University, Nanning, Guangxi 530021, P. R. China; Department of Histology and Embryology, School of Pre-Clinical Medicine, Guangxi Medical University, Nanning, Guangxi 530021, P. R. China; Central Laboratory, School of Pre-Clinical Medicine, Guangxi Medical University, Nanning, Guangxi 530021, P. R. China; Department of Histology and Embryology, School of Pre-Clinical Medicine, Guangxi Medical University, Nanning, Guangxi 530021, P. R. China; Central Laboratory, School of Pre-Clinical Medicine, Guangxi Medical University, Nanning, Guangxi 530021, P. R. China; Department of Histology and Embryology, School of Pre-Clinical Medicine, Guangxi Medical University, Nanning, Guangxi 530021, P. R. China; Central Laboratory, School of Pre-Clinical Medicine, Guangxi Medical University, Nanning, Guangxi 530021, P. R. China

**Keywords:** maternal protein, parthenogenesis, early embryo, proteome, mouse

## Abstract

**Background:**

Maternal proteins have important roles during early embryonic development. However, our understanding of maternal proteins is still very limited. The integrated analysis of mouse uniparental (parthenogenetic) and biparental (fertilized) embryos at the protein level creates a protein expression landscape that can be used to explore preimplantation mouse development.

**Results:**

Using label-free quantitative mass spectrometry (MS) analysis, we report on the maternal proteome of mouse parthenogenetic embryos at pronucleus, 2-cell, 4-cell, 8-cell, morula, and blastocyst stages and highlight dynamic changes in protein expression. In addition, comparison of proteomic profiles of parthenogenotes and fertilized embryos highlights the different fates of maternal proteins. Enrichment analysis uncovered a set of maternal proteins that are strongly correlated with the subcortical maternal complex, and we report that in parthenogenotes, some of these maternal proteins escape the fate of protein degradation. Moreover, we identified a new maternal factor-Fbxw24, and highlight its importance in early embryonic development. We report that Fbxw24 interacts with Ddb1-Cul4b and may regulate maternal protein degradation in mouse.

**Conclusions:**

Our study provides an invaluable resource for mechanistic analysis of maternal proteins and highlights the role of the novel maternal factor Fbw24 in regulating maternal protein degradation during preimplantation embryo development.

## Background

During oogenesis, proteins from the oocyte genome (maternal genome) with important roles in fertilization and early embryonic development, including protein degradation, activation of the embryonic genome, epigenetic modifications, and cell signal transduction, are largely accumulated [1]. The depletion or abnormal expression of maternal proteins not only affects embryo development but can also even lead to embryonic death [2–8]. Therefore, analyzing maternal proteins can deepen our understanding of the regulatory mechanisms underlying embryonic development.

Despite the focus of many studies of mammalian preimplantation development on transcriptomic data [9, 10], embryonic protein databases provide invaluable information for the study of maternal proteins. Protein abundance, which is closer to the phenotype, have more predictive value than messenger RNAs (mRNAs). In some cases, there is even anticorrelation, in which the mRNA is rapidly degraded after fertilization, whereas proteins persist throughout the blastocyst stage [11].

Quantitative proteomics is an effective strategy to construct protein databases for gametes or embryos. There are mass spectrometry (MS)–derived databases of early embryos, including bovine [12], zebrafish [13], and Xenopus [14, 15]. In addition, the mouse oocyte and embryo proteomes have been previously described [11, 16–18]. *Wang et al*. [16] collected 7,000 mouse oocytes at different developmental stages, including the germinal vesicle stage, the metaphase II (MII) stage, and fertilized oocytes (zygotes), and successfully identified 2,781 proteins in germinal vesicle oocytes, 2,973 proteins in MII oocytes, and 2,082 proteins in zygotes through semiquantitative MS analysis. Moreover, *Gao et al*. [17] identified nearly 5,000 proteins across 6 developmental stages (from the zygote to the blastocyst) by tandem mass tag (TMT) labeling, which they performed in duplicate. In total, 4,608 and 4,590 proteins were quantified in each experiment, and the data from the 2 experiments showed 3,767 common proteins. *Israel et al*. [11] collected and processed a total of ∼12,600 oocytes or embryos, with 3 biological replicates of ∼600 oocytes/embryos per developmental stage: unfertilized oocytes, fertilized oocytes with pronuclei, and preimplantation embryos at the 2-, 4-, and 8-cell; advanced morula; and blastocyst stages. The detected proteome comprised 6,550 proteins identified by stable isotope labeling with amino acids in cell culture (SILAC). Among the detected proteins, 5,217 were detected in ≥2 replicates of ≥1 developmental stages, and 1,709 proteins were detected in both replicates at all developmental stages.

Mouse parthenogenesis is a well-developed model to explore embryonic development [19]. Under natural conditions, an oocyte can be activated without intervention of the male counterpart. This form of reproduction, known as parthenogenesis, occurs spontaneously in various lower organisms [20, 21]. In mammals, oocytes can be activated using different methods, including high or low temperature and electrical or chemical treatment [22]. Oocyte activation rates depend on various factors, including species, age of the female, and culture conditions [22]. Moreover, inducing artificial oocyte activation is vital for somatic cell nuclear transfer research [23]. Some transcriptomic studies have expanded our understanding of the genetic programs underlying mammal parthenogenesis [24–30]; however, the protein database of parthenogenetic embryos has not been reported yet. A comparative analysis of these 2 types of embryos (biparental and uniparental embryos) may help understand maternal proteins, as parthenogenetic embryos only have maternal information, while fertilized embryos have the information of the biparental genome.

In this study, we prepared mouse uniparental embryos artificially activated and cultured *in vitro* for proteomic analysis to identify dynamic changes in maternal proteins during early embryonic development (from the pronucleus to the blastocyst stage). A comparative analysis of protein expression was performed in mature oocytes , fertilized and parthenogenetic embryos. We also performed enrichment analysis of maternal proteins strongly correlated with subcortical maternal complex (SCMC) components and found a group of maternal proteins that may escape degradation in mouse parthenogenesis. Additionally, identification and functional analyses were performed for a new key maternal factor, Fbxw24. Our uniparental embryo proteomic database is the first complete mammalian parthenogenetic embryo proteome characterized to date. This proteome dataset enables a more direct investigation of mammalian developmental processes regulated by the maternal genome at the protein level and complements the knowledge on the proteomics underlying mammalian embryonic development.

## Results

### Definition and dynamics of maternal protein expression in mouse uniparental embryos

**Figure 1:**
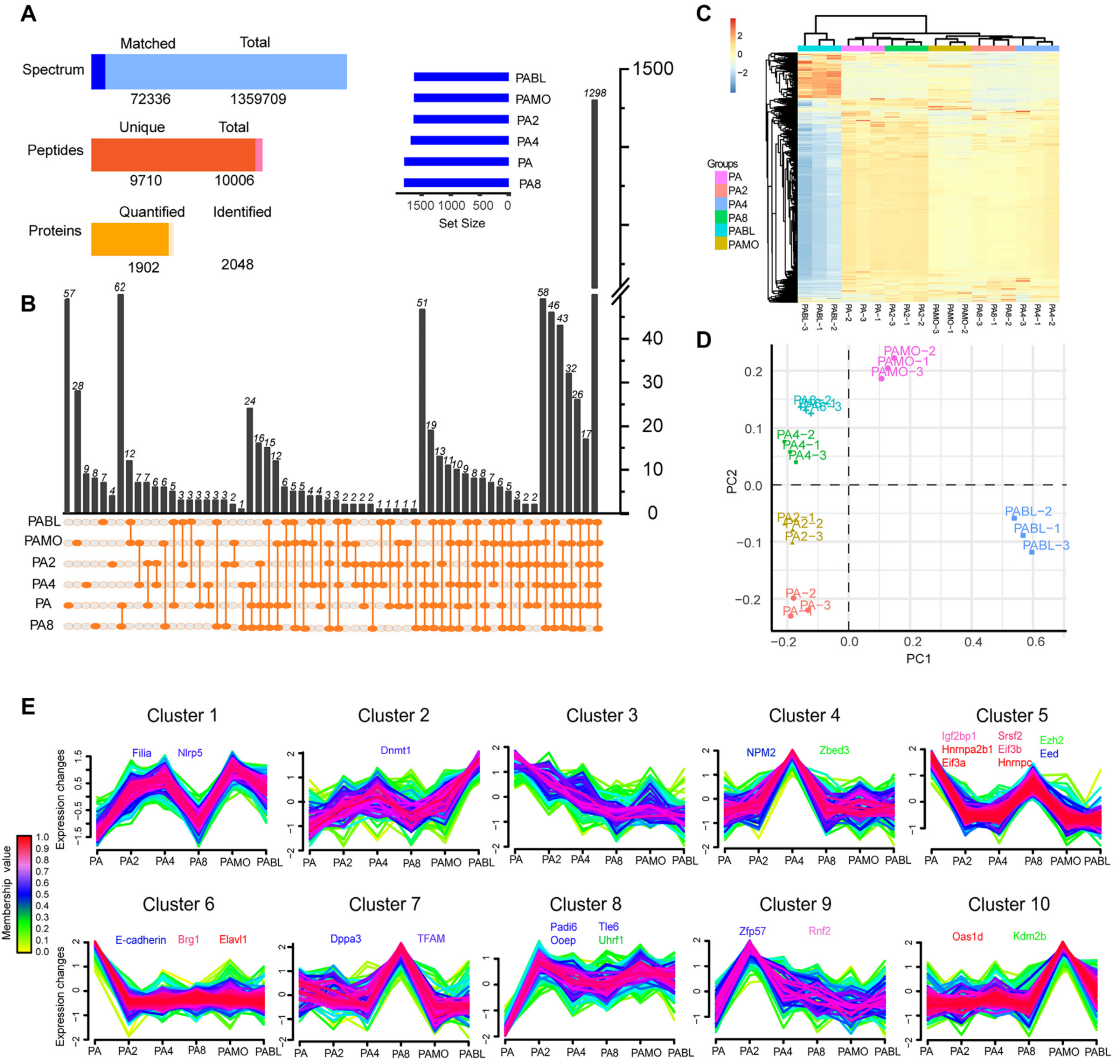
Temporal profiles of maternal protein expression in mouse uniparental embryos. (A) Statistical histogram of mass spectrometry results from mouse uniparental embryos. In total, 2,048 proteins were identified and 1,902 proteins were quantified. Total spectrum: total number of secondary spectra; Matched spectrum: the number of spectra matched to the database; Total peptides: the number of all peptides; Unique peptides: the number of unique peptides; Detected proteins: the number of proteins detected; Quantified proteins: The number of proteins that can be quantified. (B) UpSet intersection diagram of proteins detected in 6 stages of mouse uniparental embryos. The blue horizontal bar shows the number of proteins identified from each group, while the line with solid orange dots below the X-axis indicates an intersection among different groups, and the black vertical bar on the X-axis shows the number of proteins for the corresponding intersection. PA, PA2, PA4, PA8, PAMO, and PABL represent different embryonic stages of parthenogenesis: pronucleus stage (PA), 2-cell stage (PA2), 4-cell stage (PA4), 8-cell stage (PA8), morula stage (PAMO), and blastocyst stage (PABL). (C) Hierarchical grouping analysis of maternal proteins in 6 developmental stages of mouse uniparental embryos. (D) Maternal protein expression patterns analysis using principal component analysis (PCA). (E) Fuzzy c-means clustering analysis of protein expression during uniparental embryonic development. Color gradient corresponds to the cluster membership value. All protein expression changes quantified in this study are centered and scaled around a mean of 0 and a standard deviation of 1. Purple and red indicate proteins with membership values that are above 0.5, whereas yellow and green denote proteins with membership values that are less than 0.5.

The protein expression profiles of 6 embryonic stages of mouse parthenogenesis—that is, pronucleus (PA), 2-cell (PA2), 4-cell (PA4), 8-cell (PA8), morula (PAMO), and blastocyst (PABL)—were detected by label-free quantitative MS. The developmental rate of parthenogenetic embryos and their morphology are shown in Supplementary Fig. S1. For each stage, 6,000 embryos were used and the experiment was performed in triplicate. In the 3 repetitions, 1,900, 1,944, and 1,960 proteins were detected, respectively, and the total number was 2,048, of which 1,902 proteins were quantified (quantified proteins could be detected in ≥1 embryonic stage and >2 biological replicates; Fig. 1A and Supplementary Table S1). All identified proteins and peptides are shown in Supplementary Table S1.

Among the quantified proteins, 1,298 proteins were detected in all 6 successive developmental stages (Fig. 1B); however, few were detected in only 1 stage. For example, 57 proteins were only detected in the PA stage (after oocyte activation; Fig. 1B), including CCCTC binding factor, fcf1 ribosomal RNA processing protein, the general transcription factor IIF, and polypeptide 1, involved in various processes, including RNA processing, gene expression, and nitrogen compound metabolic processes. Detailed information and the annotations of intersecting groups are shown in Supplementary Table S2. The hierarchical cluster analysis of quantified proteins closely clustered PA-4 and PA-8 embryos along with PA-2 and PA embryos. However, the morula and blastocysts were separated (Fig. 1C). These results are consistent with those from principal component analysis, another clustering method (Fig. 1D). These results reveal that the blastocyst stage differed from other stages during development, consistent with the corresponding analysis of fertilized embryos [11, 17]. In mouse biparental and uniparental embryos, a major dynamic change in proteins occurs at the blastocyst stage.

Next, a fuzzy c-means analysis was performed; 10 distinct expression pattern clusters were identified (Fig. 1E and Supplementary Table S3). Five known components of the SCMC, a protein structure essential for preimplantation development [3, 31], including Ooep, Tle6, Padi6 (cluster 8 in Fig. 1E), Nlrp5 (cluster 1 in Fig. 1E), and Zbed3 (cluster 4 in Fig. 1E), were detected in this study. This indicated that these maternal proteins are essential for early embryonic development in both mouse biparental and uniparental embryos. In addition, some N6-methyladenosine (m6A) readers, including Eif3a, Eif3b, Elavl1, Hnrnpa2b1, Hnrnpc, Igf2bp1, and Srsf2, were detected (cluster 5 and cluster 6 in Fig. 1E) [32].

A cluster of highly abundant proteins was detected at PA2 (cluster 9 in Fig. 1E), including zinc finger protein 57 (Zfp57) and ring finger protein 2 (Rnf2). Lack of Zfp57 in oocytes results in failed maternal methylation imprinting at the Snrpn imprinted region; Zfp57 is also required for the postfertilization maintenance of maternal and paternal methylation imprints at multiple imprinted domains [33]. Meanwhile, Rnf2 is a component of polycomb-repressive complex 1 that functions as a redundant transcriptional factor during oogenesis and essential for proper zygotic genome activation [34].

A cluster of highly abundant proteins was detected at the PA4 stage (cluster 4 in Fig. 1E), including nucleophosmin/nucleoplasmin 2 (Npm2) and zinc finger and BED type containing 3 (Zbed3). Mouse Npm2 accumulates in oocyte nuclei and persists in preimplantation embryos. Moreover, *Npm2* knockout females have fertility defects owing to failed preimplantation embryonic development [1].

A cluster of highly abundant proteins was detected at the PA8 stage (cluster 7 in Fig. 1E), including developmental pluripotency-associated 3 (Dppa3) and mitochondrial transcription factor A (Tfam). Dppa3, also known as PGC7/Stella, protects the maternal genome from demethylation only after nucleus localization and is indispensable for the maintenance of methylation required for epigenetic reprogramming after fertilization [35]. In zebrafish embryos, knocking down *Tfam*, a regulator of mitochondrial DNA (mtDNA) replication, results in mtDNA copy number reduction and deficient oxidative phosphorylation [36].

A cluster of highly abundant proteins was detected at the morula stage (cluster 10 in Fig. 1E), including 2′-5′ oligoadenylate synthetase 1D (Oas1d) and lysine (K)–specific demethylase 2B (Kdm2b). Mutant mice lacking Oas1d display lower fertility due to ovarian follicle developmental defects, decreased ovulation efficiency, and fertilization arrest at the 1-cell stage [37]. Kdm2b, also known as Fbxl10 (F-box and leucine-rich repeat protein 10), is a JmjC domain-containing histone demethylase that contributes to embryonic neural development in mice by regulating cell proliferation and cell death [38].

Other important proteins in early embryonic development were also quantified in mouse uniparental embryos, including Filia [39] (cluster 1 in Fig. 1E), Dnmt1 [40] (cluster 2 in Fig. 1E), Eed [41] and Ezh2 [42] (cluster 5 in Fig. 1E), E-cadherin [43] and Brg1 [44] (cluster 6 in Fig. 1E), and Uhrf1 [45] (cluster 8 in Fig. 1E).

Gene Ontology (GO) analysis of proteins from each cluster (Supplementary Fig. S2A) revealed that these proteins performed many functions, including protein folding, cellular localization, cellular component biogenesis, and cellular metabolic processes. These processes provide basic energy and materials for embryonic growth and development. The interaction networks of GO terms from 4 categories, including “translation,” “peptide metabolic process,” “nucleic acid metabolic process,” and “cellular metabolic process,” with each network providing an elaborate view of the proteins participating in these biological processes, are shown in Supplementary Fig. S2B–E.

### Comparison of maternal protein expression in mouse biparental and uniparental embryos

**Figure 2:**
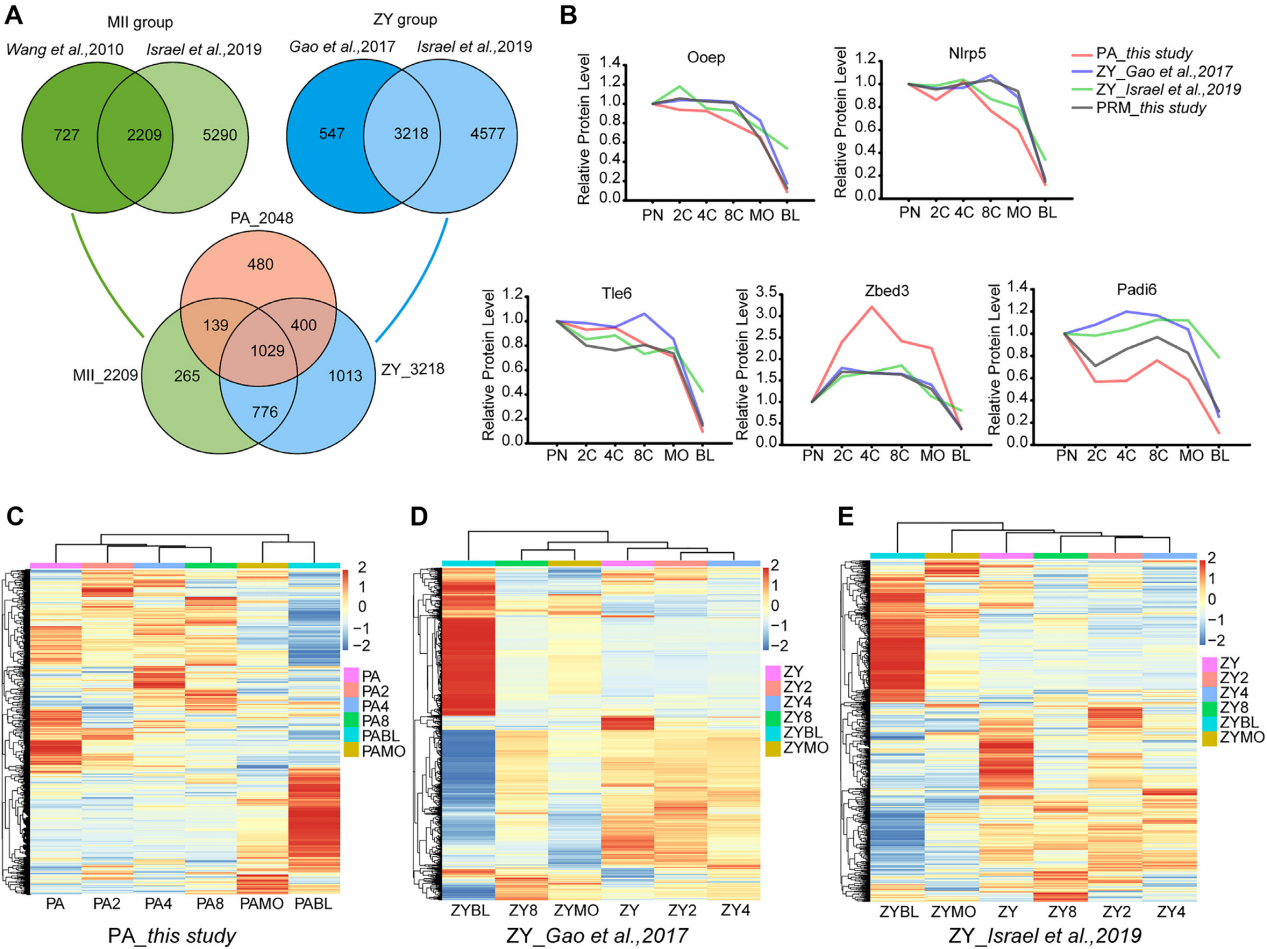
General comparative analysis of 3 proteomic databases constructed by parthenogenetic embryo (uniparental embryo, PA group), fertilized embryo (biparental embryo, ZY group), and mature oocyte (MII). (A) Venn diagram (green) shows the intersection of MII groups, quantified by Wang and Israel, respectively; Venn diagram (blue) shows the intersection of ZY groups, quantified by Gao and Israel, respectively. A sum of 1,029 proteins was found among the 3 different groups (PA, MII, ZY). (B) The expression patterns of 5 SCMC components (Ooep, Nlrp5, Tle6, Zbed3, and Padi6, named the SCMCs in this study) in fertilized and parthenogenetic embryos and their verification by parallel reaction monitoring (PRM) assay in fertilized embryo. PA_*this study*, quantified in this study; ZY_*Gao et al.,2017*, quantified by Gao et al., 2017; ZY_*Israel et al.,2019*, quantified by Israel et al., 2019; PRM_*this study*, validated by PRM assay using fertilized embryos in this study. PN, pronucleus stage; 2C, 2-cell stage; 4C, 4-cell stage; 8C, 8-cell stage; MO, morula stage; BL, blastocyst stage. Pronucleus stage was used as control. (C–E) Hierarchical clustering of protein expression for the 1,029 proteins in the PA group (C), ZY group quantified by Gao et al., 2017 (D), and ZY group quantified by Israel et al., 2019 (E), respectively.

Maternal proteins have different fates after oocyte activation, including degradation or persistence. In this study, we compared the 3 proteome databases constructed using mature oocytes (MII group), fertilized embryos (biparental embryos, ZY group/ZY embryo), and parthenogenetic embryos (uniparental embryos, PA group/PA embryo, without paternal genome). To eliminate methodological differences and ensure the reliability of these results, we used the intersection of 2 reported MII oocyte protein databases [16, 18] as the MII group (2,209 proteins, Wang and Israel, Fig. 2A) and the intersection of 2 reported fertilized embryo proteome databases [17, 18] as the ZY group (3,218 proteins, Gao and Israel, Fig. 2A). Detailed results of the Venn diagram in Fig. 2A are shown in Supplementary Table S4.

A total of 1,029 proteins derived from mature oocytes were continuously detected during preimplantation of uniparental and biparental embryos (Fig. 2A), indicating that the expression of these proteins may be independent from paternal genome regulation. We found similar expression changes of 5 SCMCs (Ooep, Nlrp5, Tle6, Zbed3, and Padi6) in the 3 embryo proteome databases (this study, *Gao et al*., and *Israel et al*.), consistent with our verification in fertilized embryos using the parallel reaction monitoring (PRM) assay (Fig. 2B), suggesting that the 5 components may play similar biological functions in both biparental and uniparental mouse embryos. Moreover, hierarchical clustering analysis of 1,029 proteins in uniparental embryos showed major protein expression changes at the blastocyst stage (Fig. 2C). Correspondingly, in biparental embryos, the major expression change was also found at the blastocyst stage (Fig. 2D and E).

**Figure 3:**
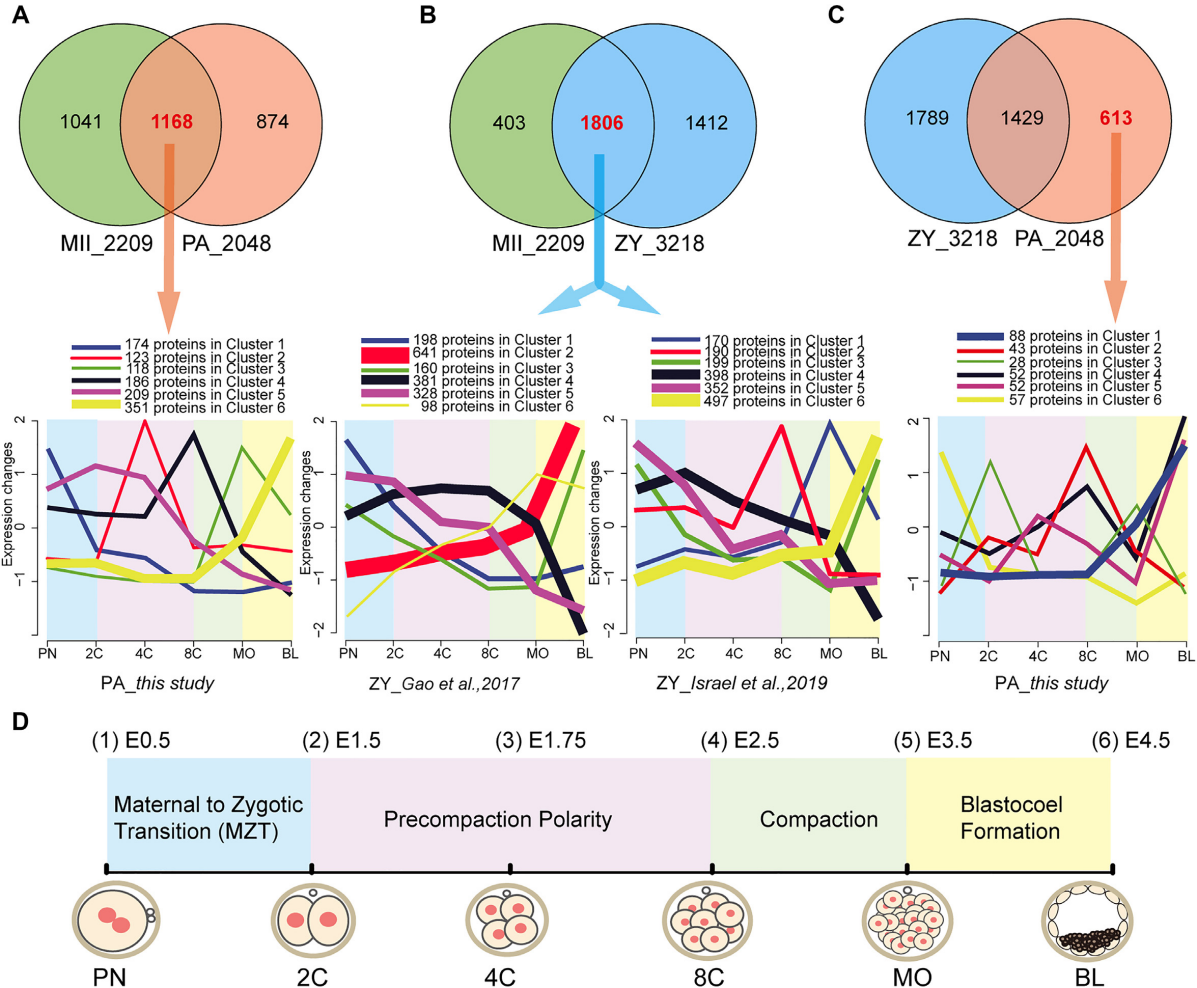
(A) Venn diagrams between MII and PA groups. In total, 1,168 proteins were detected in both MII and PA groups, and *c*-means clustering analysis was conducted for the 1,168 proteins through the development of the uniparental embryo. (B) Venn diagrams between MII and ZY groups. In total, 1,806 proteins were detected in both MII and ZY groups, and *c*-means clustering analysis was conducted for the 1,806 proteins using the protein expression database of the biparental embryo identified by Gao and Israel, respectively. (C) Venn diagrams between ZY and PA groups. In total, 613 proteins were only detected in the PA group, and c-means clustering analysis was conducted for the 613 proteins through the development of the uniparental embryo. (D) Schematic diagram of key biological events during mouse embryogenesis. E0.5 represents the 0.5 days after fertilization.

After artificial activation, 1,168 proteins in mature oocytes were detected in uniparental embryos (Fig. 3A). Correspondingly, 1,806 proteins in mature oocytes were detected in biparental embryos (Fig. 3B). Compared to biparental embryos, 613 proteins were only detected in uniparental embryos in this study (Fig. 3C). Furthermore, to reveal the dynamics of protein expression during development, fuzzy c-means clustering was performed (Fig. 3A–C and Supplementary Table S5). We found 2 similar protein expression patterns in uniparental (PA group) and biparental embryos (ZY group). First, some protein levels peak at the blastocyst stage (cluster 6 in Fig. 3A; cluster 2–Gao and cluster 6–Israel in Fig. 3B), which accounts for their highest protein number compared to other clusters, followed by a small change at the 4 early embryonic stages (PN, 2-cell, 4-cell, and 8-cell), and a great change after the 8-cell stage (compaction initiation, Fig. 3D), including proteins involved in mitotic cell cycle regulation (Tpr, Hnrnpu, Hmgb1, Mta3, Rpl17, Eif4g1, and Lmnb1) and cell–cell junction organization (Itgb1, Cttn, Cdh1, Rcc2, Ctnna1, Actn4, and Coro1c). Intriguingly, a cluster of maternal proteins detected only in uniparental embryos (PA group) in this study also followed this trend (cluster 1 in Fig. 3C), suggesting that embryonic compaction after the 8-cell stage is accompanied by protein expression changes in both biparental and uniparental embryos.

Second, the high abundance of some proteins detected in the pronucleus stage decreased during cleavage (maternal to zygotic transition, Fig. 3D); their abundance was lowest at the morula or blastocyst stage (maternal expression pattern, cluster 1 in Fig. 3A, cluster 1–Gao and cluster 5–Israel in Fig. 3B), including proteins involved in egg activation (Astl, Plat) and the posttranscriptional regulation of gene expression (Fxr1, Ddx6, Eif4enif1, Fxr2, Ybx2, Igf2bp2, Lsm14b, and Lsm14a). A similar pattern was detected in maternal proteins regulated only by the maternal genome (cluster 6 in Fig. 3C). This may underlie the successful development beyond the 2-cell stage in uniparental embryos.

### Enrichment of maternal proteins strongly correlated with SCMC components during preimplantation

The SCMC is a macromolecular complex encoded by maternal genes, mainly found in oocytes and early embryos and functionally conserved in mammals [1]. This complex is directly or indirectly involved in early embryonic development, in processes such as cell division, cytoskeleton and organelle rearrangement, maternal RNA regulation, and zygotic genome activation [1, 31]. Currently, unveiling the molecular function of the SCMC would help understand the maternal regulatory network and oocyte biology in mammals [46, 47]. In addition, the SCMC can be used as a valuable reference for the identification of important mammalian maternal factors [3]. Expression correlation analysis also aids in analyzing current “omic” data, which can screen out potential key proteins or genes. The establishment of embryo proteomes during embryonic development provides data support for such analyses.

**Figure 4:**
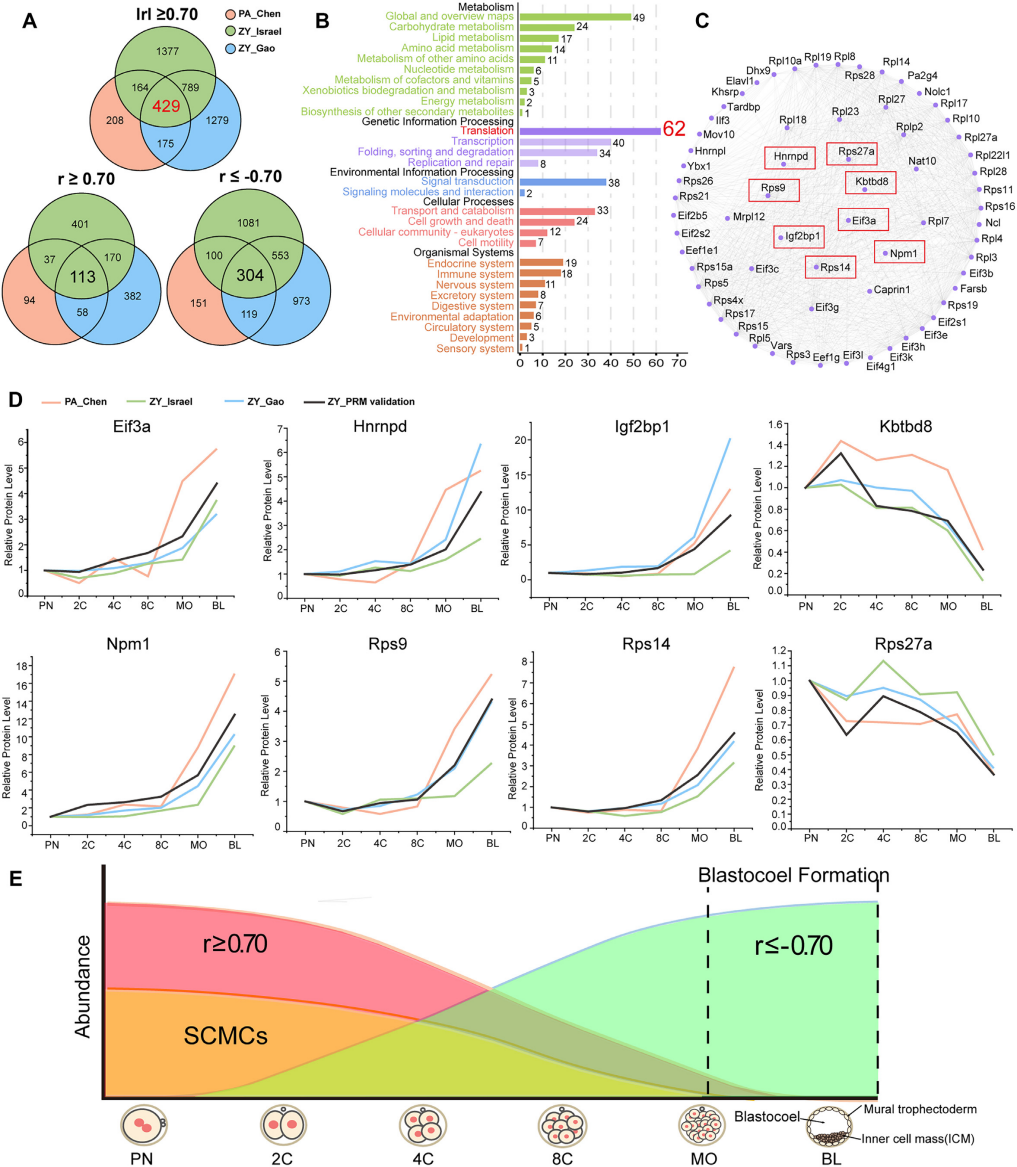
Maternal proteins that are strongly correlated with the SCMCs in mouse biparental and uniparental embryos. (A) Venn diagram shows the intersection between 3 groups (PA_Chen, representing the proteins that are strongly correlated with the SCMCs in mouse uniparental embryos; ZY_Israel, representing the proteins that are strongly correlated with the SCMCs in the proteome of the mouse biparental embryos quantified by Israel; ZY_Gao, representing the proteins that are strongly correlated with the SCMCs in the proteome of the mouse biparental embryos quantified by Gao). In total, 429 proteins were detected in both mouse biparental and uniparental embryos; 113 were strongly positively correlated (*r*≥ 0.70), and 304 were strongly negatively correlated (*r* ≤ −0.70). (B) The KEGG annotation for the 429 candidate maternal proteins. (C) The protein–protein interaction (PPI) network of the candidate maternal proteins that are involved in the biological process of “translation.” The proteins highlighted in the red box were used for further validation by the PRM assay. (D) The validation results by the PRM assay for the selected 8 key candidate proteins using fertilized embryos. PA_Chen, representing the expression pattern in mouse uniparental embryos; ZY_Israel, representing the expression pattern in the proteome of the mouse biparental embryos quantified by Israel; ZY_Gao, representing the expression pattern in the proteome of the mouse biparental embryos quantified by Gao; ZY_PRM validation, representing the validation results by the PRM assay in this study. (E) Schematic diagram shows the expression patterns between the candidate maternal proteins and the SCMCs during mouse embryogenesis.

We detected 5 components of the SCMC (Ooep, Nlrp5, Tle6, Zbed3, and Padi6, designated as SCMCs in this study), with similar patterns in mouse biparental and uniparental embryos (Fig. 2B). In this study, SCMCs were used as “target proteins” to filter candidate maternal proteins based on the correlation of their expression with SCMCs. The relationship between SCMCs and other quantified proteins in the PA (uniparental embryos) and ZY (biparental embryos, including 2 protein databases identified by Gao and Israel) groups was analyzed by Pearson's correlation coefficients (Supplementary Table S6). An absolute correlation coefficient ≥0.70 (|*r*| ≥ 0.70 and *P* ≤ 0.05) denotes a strong expression correlation [48]. Based on this criterion, there were 429 candidate proteins (|*r*| ≥ 0.70 and *P* ≤ 0.05) enriched in mouse biparental and uniparental embryos (Fig. 4A and Supplementary Table S7). Of these proteins, 113 were strongly positively correlated (*r* ≥ 0.70 and *P* ≤ 0.05, with the lowest protein level at the blastocyst stage) and 304 were strongly negatively correlated (*r* ≤ −0.70 and *P* ≤ 0.05, with the highest protein level at the blastocyst stage; Fig. 4A, Fig. 4E, and Supplementary Table S7).

Kyoto Encyclopedia of Genes and Genomes (KEGG) pathway analysis was conducted on 429 candidate proteins (Fig. 4B). These proteins were found to be widely involved in the regulation of numerous biological processes during embryonic development, including metabolism, genetic information processing, environmental information processing, and cellular processes. The protein–protein interaction (PPI) network of candidate proteins involved in “translation” was obtained using the STRING database [81], showing a complicated PPI network (Fig. 4C). To validate the expression trends of these candidate proteins, 8 candidate proteins were randomly selected (highlighted with a red box in Fig. 4C) for the PRM assay in biparental embryos. The results revealed the same protein expression pattern as in the proteome analysis (Fig. 4D).

The number of strongly negatively correlated proteins (304 proteins, Fig. 4A and Supplementary Table S7) was about 3 times that of strongly positively correlated proteins (113 proteins, Fig. 4A and Supplementary Table S7). These strongly negatively correlated proteins have complex interaction networks (Supplementary Fig. S3A). GO analysis showed that these proteins are mainly located in the nucleus and involved in translation initiation, mRNA transport, and ribosome biogenesis. The molecular functions of these proteins include translation initiation factor activity, protein activating ATPase activity, protein binding, and mRNA binding (Supplementary Fig. S3B). In addition, KEGG analysis showed that these proteins were mainly involved in the spliceosome, ribosome, and proteasome pathways (Supplementary Fig. S3B). These strongly negatively correlated maternal proteins were highly expressed in the blastocyst stage in both biparental and uniparental embryos (Fig. 4E), suggesting their role in blastocoel formation and initial differentiation of embryonic cells.

In this study, a group of candidate maternal proteins that strongly correlated with SCMCs was enriched. Their protein levels showed similar trends in biparental and uniparental embryos: highest or lowest abundance at the blastocyst stage (Fig. 4E). Obviously, their expression changes were not greatly affected by the paternal genome (or they may be regulated by the maternal genome alone). The inner cell mass (ICM) and the trophectoderm (TE) are formed at the blastocyst stage [49]. To some extent, these maternal proteins may provide the molecular basis for these processes, including cleavage, ICM and TE formation, and it may explain why uniparental embryos can develop into the blastocyst stage and embryonic stem cells can be derived from both biparental and uniparental embryos.

### Some maternal proteins that usually degrade remain in mouse uniparental embryos

Maternal protein degradation is an important process during early embryogenesis [49], necessary after embryonic genome activation [49]. In this study, to obtain more candidate proteins for further analysis, expression correlation analysis was used to explore the relationship between 15 known maternal proteins identified in both biparental and uniparental embryos (Ooep, Nlrp5, Tle6, Zbed3, Padi6, Atg5, Npm2, H1foo, Zar1, Oas1d, Pou5f1, Ago2, Dnmt1, Cdh1, and Ctcf, including SCMCs) and other quantified proteins (Supplementary Table S6). The intersection of correlation analysis result in two reported fertilized embryo proteome databases used as the ZY group (1,958 proteins, Supplementary Fig. S4A). In addition, the detailed intersection relationships of the 15 maternal and other candidate proteins (|*r*| ≥ 0.70 and *P* ≤ 0.05) are shown in Supplementary Fig. S4B (PA group), Supplementary Fig. S4C (ZY, identified by Gao), and Supplementary Fig. S4D (ZY, identified by Israel).

**Figure 5:**
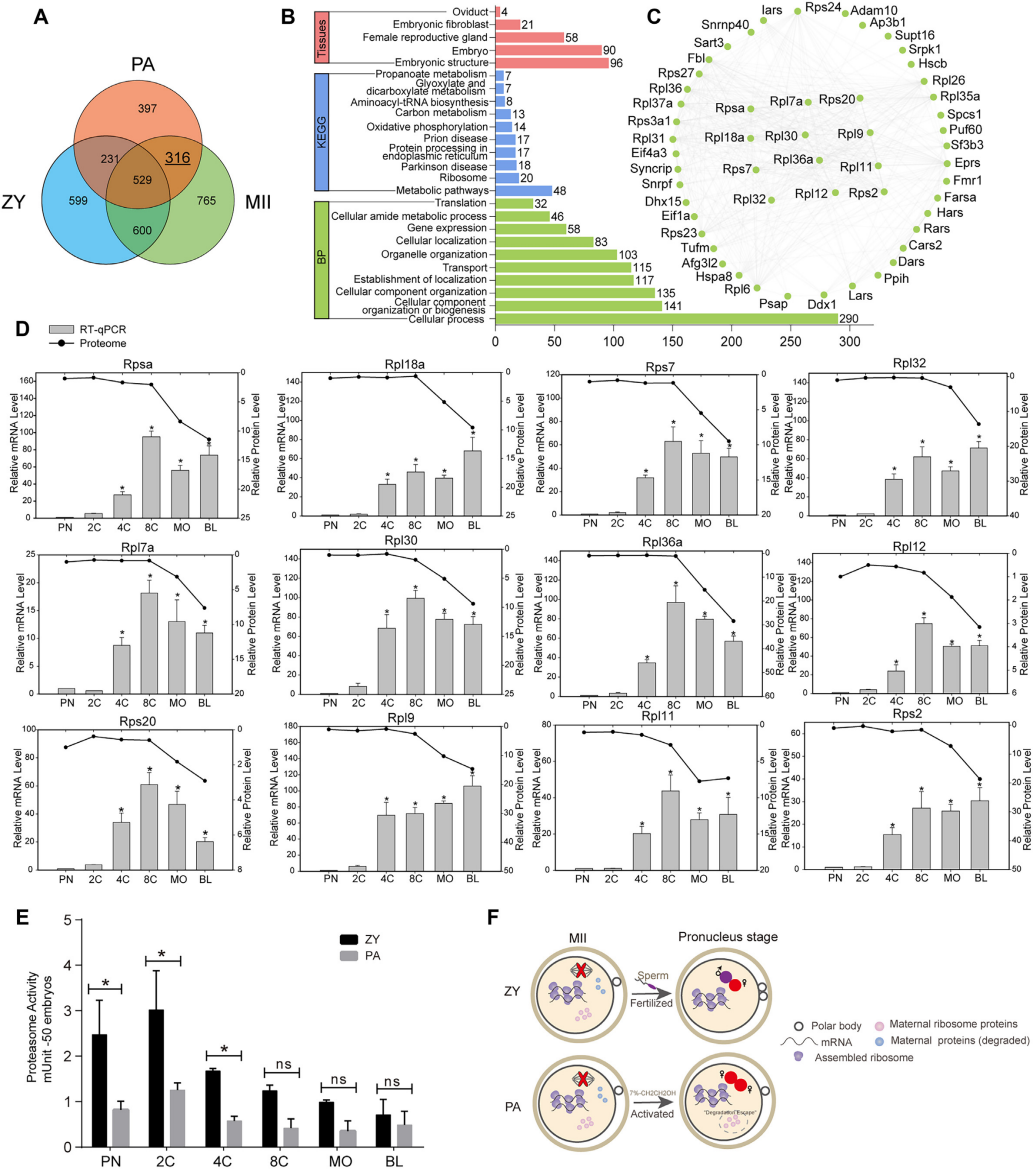
(A) The Venn diagram shows the intersection of the candidate proteins that strongly correlated with 15 key maternal proteins in 3 groups. The blue circle shows the number of candidate proteins that were analyzed from the proteome of biparental embryos (ZY group, identified by Gao and Israel), the green circle shows the number of the candidate proteins that were analyzed from the proteome of uniparental embryos (PA group, identified in this study), and the light orange circle shows the maternal proteins quantified in metaphase II (MII) stage oocytes (MII group, quantified by Wang and Israel). In total, 316 maternal proteins detected only in PA and MII groups and in biparental embryos (ZY) were detected in the MII stage oocyte but degraded after fertilization. (B) The annotation results for the 316 maternal proteins. The green columns show the annotation of the biological process, the blue columns show the annotation of KEGG, and the red columns show the localization in different tissues. (C) The protein–protein interaction (PPI) network of the candidate maternal proteins that are involved in biological process of “gene expression.” (D) Twelve maternal ribosomal factors involved in the process of “gene expression” were validated by quantitative reverse transcription polymerase chain reaction (RT-qPCR) assay using uniparental embryos. The lines represent the expression pattern in protein level, and columns represent the expression pattern in mRNA level. Pronucleus stage was used as control. *P < 0.05. (E) The proteasome activity was detected in 6 developmental stages of mouse biparental and uniparental embryos using a fluorometric proteasome activity assay kit. One unit of proteasome activity is defined as the amount of proteasome that generates 1.0 nmol of the fluorescently tagged 7-amino-4-methylcoumarin (AMC) per minute at 37°C. (F) Schematic diagram of “degradation escape” maternal proteins. MII, metaphase II of oocyte; 7% CH2CH2OH, M2 solution containing 7% ethanol.

The Venn analysis among the MII, PA, and ZY groups differently sorted these candidate proteins (Fig. 5A, Supplementary Table S8): 765 proteins were detected only in MII oocytes, indicating that these maternal proteins may be involved in oogenesis and oocyte maturation; 397 proteins were detected only in PA embryos, indicating their role in the development of uniparental embryos and possible regulation by the maternal genome; 599 proteins were detected only in ZY embryos, indicating that these proteins may be necessary for the development of biparental embryos and are regulated by the zygote genome; 529 proteins were detected in all groups (MII, PA, ZY), indicating that these maternal proteins may be involved not only in oogenesis and oocyte maturation but also in embryonic development (including biparental and uniparental embryos); 231 proteins were detected in PA and ZY embryos, indicating their roles in early embryonic development in biparental and uniparental embryos (after fertilization or artificial activation); and 600 proteins were detected in both MII oocytes and ZY embryos, which reveals that these proteins may not only play a role in oogenesis and oocyte maturation but also remain during embryonic development.

In particular, 316 proteins were detected in MII oocytes and PA embryos but not in ZY embryos, indicating that these maternal proteins may be degraded after fertilization in ZY embryos. However, in uniparental embryos, these maternal proteins appear to escape degradation, which indicates a role in parthenogenesis. In the normal mammalian bisexual reproduction, their function may only be regulating oocyte development or maturation. GO analysis showed that these proteins were mainly located in embryos and involved in translation, gene expression, and transport. Moreover, KEGG results suggest their involvement in pathways related to metabolism, ribosomes, and oxidative phosphorylation (Fig. 5B). Furthermore, quantitative polymerase chain reaction (PCR) verification in uniparental embryos on 12 maternal ribosomal factors involved in “gene expression” (Fig. 5C) showed coinciding mRNA levels to the protein level (peak at morula or blastocyst stage) (Fig. 5D), suggesting that these maternal proteins remain present though the early development of mouse uniparental embryos.

In biparental embryos, protein degradation occurs after fertilization [49] and is dependent on the maternally derived ubiquitin-proteasome system and autophagy [50–52]. After the inhibition of proteasomal activity, polyubiquinated proteins accumulate after fertilization [53]. In this study, we further detected the proteasomal activity of biparental and uniparental embryos in 6 early developmental stages. The proteasome activity of biparental embryos before the 8-cell stage was stronger than that of uniparental embryos (Fig. 5E). To some extent, this may allow for the “degradation escape” of these maternal proteins in uniparental embryos. We speculate that in PA embryos, these proteins may escape degradation; thus, we called this phenomenon “degradation escape” in uniparental embryos (Fig. 5F).

### Expression and functional analysis of a new maternal factor

The components of the SCMC include Ooep (Floped), Nlrp5 (Mater), Tle6, Filia, Zbed3, Nlrp2, and possibly Padi6 and Nlrp7 [8, 31, 54]. Among them, Ooep, Nlrp5, and Tle6 directly interact with each other and are necessary to maintain the stability of the complex [1, 6, 31, 55].

In this study, a comparative analysis of candidate proteins strongly correlated with the core SCMC components (Ooep, Nlrp5, and Tle6) in mouse biparental and uniparental embryos highlighted a family of F-box/WD40 repeat-containing proteins (Fbxws). Only Fbxw11 and Fbxw15 were detected in uniparental embryos (PA group, this study), while Fbxw8, Fbxw11, Fbxw13, Fbxw15, Fbxw16, Fbxw18, Fbxw19, Fbxw20, Fbxw21, Fbxw22, Fbxw24, Fbxw26, and Fbxw28 were detected in biparental embryos (ZY group, identified by Gao and Israel; Table 1). Obviously, the quantity of Fbxws in biparental embryos is higher than in uniparental embryos. Furthermore, in uniparental embryos, Fbxw11 was not correlated with SCMC components, but in biparental embryos, it showed a strong negative correlation (*r* ≤ −0.70, marked in green, Table 1) with SCMC components. In addition, the expression correlation between Fbxw15 and the SCMC components was the opposite in the 2 embryos: Fbxw15 had a strong negative correlation (*r* ≤ −0.70, marked in green, Table 1) with SCMC components in uniparental embryos but a strong positive correlation (*r* ≥ 0.70, marked in red, Table 1) with SCMC components in biparental embryos (Table 1). Finally, Fbxw24 was selected for further analysis, because its correlation with the other Fbxws was similar to that of SCMC components in biparental embryos (Table 1), and its role in preimplantation embryonic development has not been studied at all yet.

**Table 1:** The expression correlation coefficient of Fbxws and SCMC components

Gene name	Groups	Ooep		Nlrp5		Tle6		Fbxw24	
		*P*	*r*	*P*	*r*	*P*	*r*	*P*	*r*
Fbxw8	PA_this study	—	—	—	—	—	—	—	—
	ZY_Israel	0.5822	0.2864	0.6216	0.2580	0.3570	0.4614	0.2948	0.5159
	ZY_Gao	0.0009	−0.9749	0.0000	−0.9946	0.0002	−0.9889	0.0021	−0.9628
Fbxw11	PA_this study	0.7422	0.1736	0.5370	0.3195	0.7132	0.1936	—	—
	ZY_Israel	0.0570	−0.7981	0.0067	−0.9325	0.0287	−0.8582	0.0502	−0.8109
	ZY_Gao	0.0561	−0.7997	0.1031	−0.7250	0.0924	−0.7404	0.0346	−0.8440
Fbxw13	PA_this study	—	—	—	—	—	—	—	—
	ZY_Israel	0.3686	0.4517	0.1762	0.6342	0.3035	0.5081	0.1168	0.7062
	ZY_Gao	0.0000	0.9949	0.0009	0.9755	0.0004	0.9837	0.0002	0.9898
Fbxw15	PA_this study	0.0111	−0.9128	0.0247	−0.8687	0.0063	−0.9342	—	—
	ZY_Israel	0.0137	0.9027	0.0153	0.8972	0.0228	0.8742	0.0009	0.9755
	ZY_Gao	0.0010	0.9742	0.0037	0.9499	0.0021	0.9626	0.0014	0.9696
Fbxw16	PA_this study	—	—	—	—	—	—	—	—
	ZY_Israel	0.0246	0.8689	0.0135	0.9037	0.0680	0.7787	0.0090	0.9214
	ZY_Gao	0.0003	0.9865	0.0019	0.9645	0.0010	0.9744	0.0002	0.9895
Fbxw18	PA_this study	—	—	—	—	—	—	—	—
	ZY_Israel	0.0057	0.9376	0.0065	0.9336	0.0208	0.8797	0.0013	0.9708
	ZY_Gao	0.0005	0.9810	0.0044	0.9452	0.0026	0.9585	0.0008	0.9775
Fbxw19	PA_this study	—	—	—	—	—	—	—	—
	ZY_Israel	0.0329	0.8479	0.0299	0.8554	0.0337	0.8461	0.0486	0.8141
	ZY_Gao	0.0000	0.9978	0.0003	0.9850	0.0001	0.9909	0.0001	0.9942
Fbxw20	PA_this study	—	—	—	—	—	—	—	—
	ZY_Israel	0.0462	0.8189	0.0308	0.8530	0.0325	0.8491	0.0025	0.9585
	ZY_Gao	0.0001	0.9917	0.0013	0.9705	0.0006	0.9798	0.0002	0.9896
Fbxw21	PA_this study	—	—	—	—	—	—	—	—
	ZY_Israel	0.0178	0.8891	0.0019	0.9644	0.0090	0.9216	0.0019	0.9638
	ZY_Gao	0.0000	0.9944	0.0008	0.9772	0.0003	0.9851	0.0001	0.9923
Fbxw22	PA_this study	—	—	—	—	—	—	—	—
	ZY_Israel	0.0040	0.9480	0.0037	0.9497	0.0251	0.8677	0.0044	0.9454
	ZY_Gao	0.0001	0.9931	0.0006	0.9795	0.0003	0.9865	0.0002	0.9893
Fbxw26	PA_this study	—	—	—	—	—	—	—	—
	ZY_Israel	0.0328	0.8482	0.0438	0.8238	0.0175	0.8900	0.0640	0.7857
	ZY_Gao	0.0000	0.9947	0.0002	0.9871	0.0001	0.9921	0.0000	0.9967
Fbxw28	PA_this study	—	—	—	—	—	—	—	—
	ZY_Israel	0.0209	0.8796	0.0238	0.8712	0.0095	0.9192	0.0107	0.9142
	ZY_Gao	0.0021	0.9621	0.0071	0.9303	0.0044	0.9451	0.0035	0.9510

**Figure 6:**
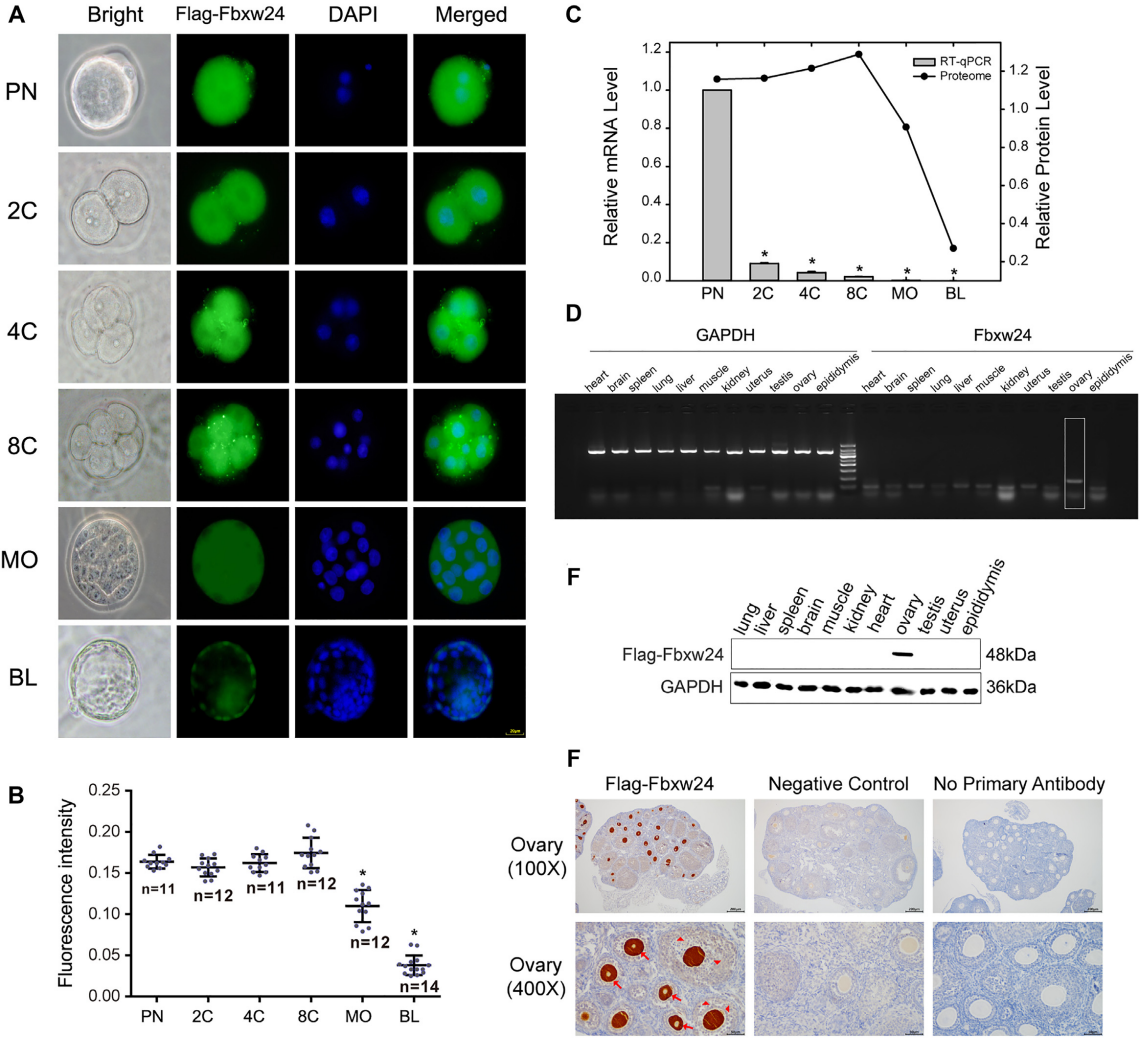
The expression analysis of Fbxw24 in mouse. (A) Cellular localization of Fbxw24. The different-stage embryos were cultured from the zygotes of C57BL/6J-*Fbxw24*^em(Linker-3xFlag)^ mice. Each sample was performed using DAPI to allow for the visualization of DNA (blue). Scale bar: 20 µm. PN, pronucleus stage; 2C, 2-cell stage; 4C, 4-cell stage; 8C, 8-cell stage; MO, morula stage; BL, blastocyst stage. (B) Quantification of Fbxw24 fluorescence intensity in 6 biparental embryo stages. Pronucleus stage was used as control. *P < 0.05. (C) The relative transcripts and protein abundance of Fbxw24 in mouse fertilized embryos. *P < 0.05. (D) The mRNA level of Fbxw24 in different tissues of the mouse. GAPDH was used as a control. (E) Immunoblots of lysates isolated from different tissues of C57BL/6J-*Fbxw24*^em(Linker-3xFlag)^ mice. GAPDH was used as a loading control. (F) Immunohistochemical analysis of Fbxw24 in ovary. The sections obtained from 4-week-old C57BL/6J-*Fbxw24*^em(Linker-3xFlag)^ heterozygous female mice. The wild-type female littermate was a negative control. The representative oocytes with positivity at different follicular stages are indicated by red arrows and granulosa cells with positivity are indicated by red arrowheads. (Magnification: 100×, 400×)

First, an immunofluorescence assay showed predominant cytoplasmic location of Fbxw24 in oocytes and early embryos (Supplementary Fig. S5 and Fig. 6A) and decreasing fluorescence intensity after the 8-cell stage (Fig. 6B). After fertilization, the mRNA level of Fbxw24 was high at the pronuclear stage, significantly decreasing after the 2-cell stage (Fig. 6C). With cleavage progression, the mRNA level continued to decrease and was almost undetectable at the blastocyst stage; its protein level sharply decreased from the 8-cell to the blastocyst stage (Fig. 6C), coinciding with the fluorescence intensity detected. Although the mouse zygotic genome was activated after the 2-cell stage, *Fbxw24* transcript level remained low. These results reveal that Fbxw24 molecules mainly accumulate during oocyte maturation and that *Fbxw24* probably plays a role as a maternal-effect gene in early embryonic development.

Second, *Fbxw24* mRNA level was detected using different mouse tissues. We found its transcripts in the ovary but not in 10 other tissues (including testis; Fig. 6D). Immunohistochemistry and immunoblotting were used to assess Fbxw24 expression and location at the protein level. Immunoblotting for 11 tissues detected Fbxw24 protein (∼48 kDa) in the mouse ovary but not in 10 other tissues (Fig. 6E), and the immunohistochemistry results indicated that Fbxw24 was located in oocytes (red arrows) and granulosa cells (red arrowheads) at different follicular stages (Fig. 6F).

**Figure 7:**
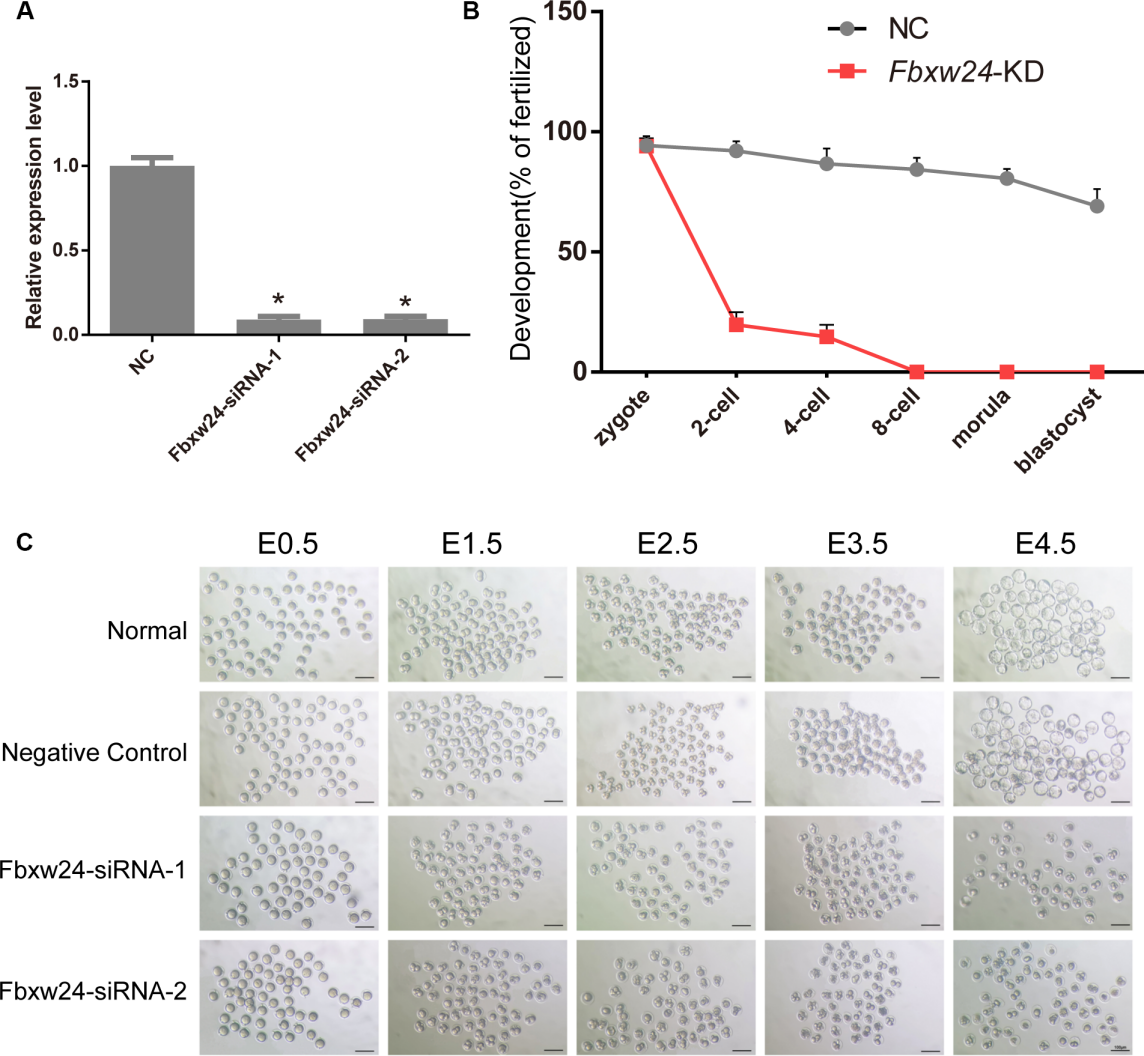
The function analysis of *Fbxw24* during mouse embryo development. (A) Efficiency of siRNA-mediated knockdown of *Fbxw24*. Standard deviation (SD) is shown by the error bars. (B) Percentage of successfully developed embryos at each stage following *Fbxw24* knockdown. The development rate is calculated with the quantity of zygotes as the denominator. SD is shown by the error bars. (C) Knockdown of *Fbxw24* results in the developmental arrest that started from E1.5. Scale bars represent 100 µm.

Next, small interfering RNAs (siRNAs) were injected into MII oocytes followed by intracytoplasmic sperm injection (ICSI) to knock down *Fbxw24* during early embryogenesis (Fig. 7A). Compared with the control, the developmental competence of *Fbxw24*-knockdown embryos decreased from the 2-cell stage onward, and the embryonic development was arrested at the 8-cell stage, failing at the morula and blastocyst stages (Fig. 7B and C). *Fbxw24*-knockdown embryos were mainly arrested between the 2- and 8-cell stages, showing similar results to those of known maternal-effect genes, such as *Nlrp5* [7], *Nlrp2* [56], and *Padi6* [8].

**Figure 8:**
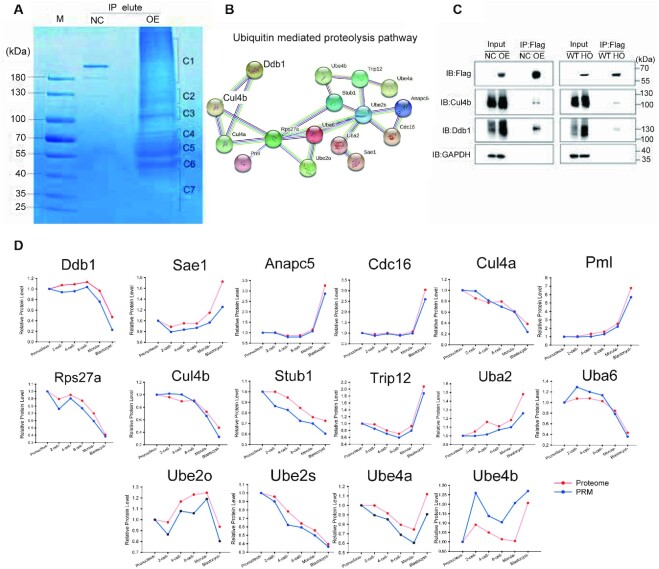
Fbxw24 interacts with Ddb1-Cul4b in mouse. (A) Fbxw24 was pulled down and eluted by an N-terminal 3xFlag tag. Sodium dodecyl sulfate–polyacrylamide gel electrophoresis was used to isolate the extracts obtained from the 3xFlag target, and then the extracts were observed by Coomassie brilliant blue staining. Extraction of 7 Fbxw24 immunoprecipitated products was performed for subsequent in-gel trypsin digestion. (B) Sixteen members of ubiquitin-mediated proteolysis pathways were identified in Fbxw24 pull-down complexes by liquid chromatography/tandem MS analysis. (C) A coimmunoprecipitation assay in HEK-293T cells and ovaries of C57BL/6J-*Fbxw24*^em (Linker-3xFlag)^ mice was conducted to validate the interaction of Ddb1-Cul4b with Fbxw24. NC, negative control; OE, overexpression; WT, wild-type mice; HO, homozygote mice. (D) The expression pattern analysis of 16 members identified in Fbxw24 pull-down complexes by MS analysis and verification by PRM in fertilized embryos. The red line displays the change of protein level from the previously reported proteome of mouse fertilized embryos identified by Gao, and the blue line displays the results of PRM analysis in this study. The pronucleus stage was used as a control.

To study the underlying mechanism, we performed protein-protein interaction enrichment of Fbxw24 by stable isotope labeling of amino acids in cell culture and immunoprecipitation-MS [SILAC-IP-MS] (Fig. 8A and Supplementary Table S10). Notably, several components of the ubiquitin-mediated proteolysis pathway, including DNA damage-binding protein 1 (Ddb1), Cullin-4B (Cul4b), ubiquitin conjugation factor E4B, ubiquitin conjugation factor E4A, ubiquitin-40S ribosomal protein S27a (Rps27a), and ubiquitin-conjugating enzyme E2S (Ube2s), were detected in Fbxw24 pull-down complexes (Fig. 8B). The interaction between Fbxw24 and Ddb1-Cul4b was validated in HEK-293T cells by coimmunoprecipitation and their interaction further explored *in vivo* using C57BL/6J-*Fbxw24*^em(Linker-3xFlag)^ homozygote[HO] mouse ovaries and wild-type[WT] littermates as control (Fig. 8C). These results suggest a novel interaction between Fbxw24 and Ddb1-Cul4b. Cullin4 (CUL4) uses damaged DNA binding protein 1 (DDB1) as a linker to interact with a subset of DDB1-Cullin–associated factors [57, 58] and DDB1 is highly expressed in mouse oocytes [59]. Additionally, the expression pattern of 16 members identified in the Fbxw24 pull-down complexes was analyzed using MS analysis and verified using PRM in fertilized embryos, revealing different expression patterns during early embryonic stages (Fig. 8D). Among them, SUMO-activating enzyme subunit 1, anaphase-promoting complex subunit 5, cell division cycle protein 16 homolog, protein PML, E3 ubiquitin-protein ligase TRIP12, and SUMO-activating enzyme subunit 2 showed an upward trend; in contrast, Rps27a, Cul4b, STIP1 homology, and U box-containing protein 1 and Ube2s showed a downward trend. These results reveal that Fbxw24 may be involved in the regulation of maternal protein degradation during early embryonic development.

## Discussion

The function of maternal proteins was initially reported in invertebrates. Researchers induced mutants in *Drosophila* and found that the polarity of *Drosophila* eggs and embryos is regulated by the maternal factors. Therefore, the importance of maternal molecules for embryonic development has been demonstrated [60–66]. Subsequently, in vertebrates, the function of maternal factors was also reported, including the fusion of male and female pronucleus, zygotic genome activation, and the degradation of maternal components [1, 67]. In mammals, the first maternal factor Mater was reported in mouse [7]. Mater (maternal antigen that embryos require; also known as Nlrp5) may be related to the activation of the zygotic genome [7]. The known maternal-effect factors in the mouse have been reviewed by Li et al. [1] and Zheng and Liu [67]. Although the importance of maternal factors for embryonic development has been known for a long time, research progress in mammals has been slow due to the limitations of research materials and technology.

Mouse parthenogenetic embryos are only regulated by the maternal genome, with similar morphology compared to fertilized embryos, so some proteins expressed in parthenogenetic and fertilized embryos may have similar expression trends, and their presence and normal expression changes provide a molecular basis for the early development. Uniparental embryos derived from only the oocyte may be a unique model for studying genomic imprinting and the maternal contribution to embryonic development. In addition, the ICM of the blastocyst in parthenogenetic embryos can also be used as a source of parthenogenetic embryonic stem cells; this has been successfully established in many species, including mouse, monkey, and human [68–71]. Recently, a study reported that fertile mice can be bred from single oocytes by targeted DNA methylation by rewriting 7 imprinting control regions without sperm participation [72]. Although parthenogenetically activated oocytes cannot develop to term in mammals due to the disruption of imprinted gene expression and DNA methylation status, the protein landscape of parthenogenetic embryos has not been studied.

In this study, to obtain more information about maternal proteins, we first constructed the protein database of mouse parthenogenetic embryos before implantation and compared mouse biparental and uniparental embryos at the protein level. By label-free quantitative MS, we detected a total of 2,048 proteins in 6 preimplantation stages of mouse parthenogenesis and found 2 similar protein expression patterns in uniparental and biparental embryos; the 2 patterns may be mainly regulated by the maternal genome. Second, we used the SCMC as target proteins and explored the expression correlation between the SCMC and other identified proteins in biparental and uniparental embryos. We obtained a group of key candidate maternal proteins, among which some were strongly negatively correlated with SCMC and may play an important role in blastocoel formation.

In addition, by analyzing the relationship between 15 known maternal proteins and other proteins identified in both biparental and uniparental embryos, we found that some maternal proteins are degraded with oocyte activation in biparental embryos; however, in uniparental embryos, they remain during preimplantation, and their mRNA and protein levels show an upward trend. Moreover, the proteasome activity of biparental embryos before the 8-cell stage was stronger than that of uniparental embryos, which revealed that these maternal proteins in mouse uniparental embryos may escape degradation. Based on these results, we inferred that some maternal protein degradation after oocyte activation may require sperm participation. Fertilization may trigger the degradation of these maternal ribosomal proteins, or alternatively, the sperm contains some factors that may regulate their degradation. Since the development of uniparental embryos occurs without sperm or any paternal contribution, it leads to the “degradation escape” of maternal protein.

Moreover, among the candidate proteins that strongly correlated with the 3 core SCMC components (Ooep, Nlrp5, and Tle6), a large set of Fbxws were present in mouse biparental embryos, which may suggest the involvement of the Skp1-Cullin-F-box (SCF) complex in maternal protein degradation in mice.*Wang et al*.[16] also identified a large group of F-box proteins in mouse oocytes and zygotes. SCF protein-ubiquitin ligase complex member F-box proteins are highly abundant in oocytes and 2-cell embryos [73]. For example, Fbxw15/Fbxo12J is an F-box protein-encoding gene selectively expressed in oocytes of the mouse ovary [74]. These F-box proteins may play important roles in protein degradation after fertilization, as different F-box complexes can selectively degrade specific target proteins. Finally, Fbxw24 was selected for further analysis. We found that Fbxw24’s expression pattern was very similar to a previously reported maternal-effect factor [8, 75, 76], and following *Fbxw24* knockdown by siRNA interference, the affected embryos showed developmental arrest. By SILAC-immunoprecipitation-MS (SILAC-IP-MS) and validation *in vivo*, we confirmed a new specific interaction between Fbxw24 and Cul4b-Ddb1 (key components of the ubiquitin-proteasome pathway). We speculate that Fbxw24 may be involved in the degradation of maternal proteins in early embryos. These results suggest that *Fbxw24* is a new putative maternal-effect gene, and the comparative analysis of biparental and uniparental embryos may be helpful to find out the key maternal factors regulating early embryonic development.

There are some potential limitations to this study. Namely, the quantity of proteins identified in parthenogenetic embryos (2,048 proteins) is lower than that reported before in fertilized embryos. *Gao et al*. identified nearly 5,000 proteins across 6 developmental stages by TMT, and *Israel et al*. identified 6,550 proteins by SILAC. However, the mouse parthenogenetic embryo only contains maternal genomic information, which may cause some genuine biological absence. Additionally, differences in protein quantity may have occurred due to the following: (i) different proteome construction strategies. The known proteome of fertilized embryos was constructed using a quantitative proteomic strategy based on TMT labeling or SILAC [11, 17], while that of parthenogenetic embryos was constructed by label-free quantitative MS. (ii) Different protein databases are used for the search. The raw MS data of fertilized embryos identified by *Gao et al*. were searched with the ProLucid algorithm against the International Protein Index (IPI) mouse protein database [17], and the raw MS data of fertilized embryos identified by *Israel et al*. were searched using MaxQuant software against the Uniprot KB database [11]. The raw MS data of parthenogenetic embryos were searched using MaxQuant software against the Swiss-Prot mouse database (UP000000589). Importantly, the Swiss-Prot database is manually annotated and reviewed, and the number of proteins is lower than in the IPI and Uniprot KB databases. Moreover, some known maternal proteins, including Brg1, Dppa3, Ezh2, Filia, and Zfp57, were detected in this study (uniparental embryos) and in the proteome of biparental embryos identified by *Israel et al*., but not identified by *Gao et al*. These undetected members may hint at different technical limitations, but since these proteins were also detected in parthenogenetic embryos by label-free quantitative MS, the proteome database of uniparental embryos constructed in this study appears reliable. This study reports for the first time the proteome of parthenogenetic embryos, but there is no other similar protein database that can be used for coverage comparison to date, and the existence of false negatives in the parthenogenetic embryo proteome caused by the artificial activation method remains to be investigated.

In conclusion, we systematically constructed a maternal protein file of mouse uniparental embryos and showed the dynamic patterns of maternal proteins during preimplantation using a combined analysis of uniparental and biparental embryos. These data are valuable for further mechanistic studies of maternal proteins and aid in mining potential key players and the associated regulatory mechanisms governing early embryo development.

## Methods

### Experimental animals

C57/BL6J mice were obtained from the animal breeding colony in the animal experimental center of Guangxi Medical University, China. To produce C57BL/6J-*Fbxw24*^em(Linker-3xFlag)^ mice, CRISPR/Cas9-mediated homologous recombination (Shanghai Model Organisms Center, Inc., Shanghai, China) was used to insert the 3x flag sequence and frame it with the last exon of the Fbxw24 gene before the stop codon. The mice were kept in rooms with controlled temperatures under a 12-hour light/dark cycle. There was no restriction to food and water, and mice were fed *ad libitum*.

### Collection of mouse oocytes and embryos

Six- to eight-week-old C57BL/6 J female mice were super-ovulated following injection of 10 IU of pregnant mare serum gonadotropin (PMSG; Ningbo Second Hormone Factory, Ningbo, China) and 10 IU of human chorionic gonadotropin (hCG; Ningbo Second Hormone Factory). The injection of the 2 hormones was performed 48 hours apart. Fully grown germinal vesicle oocytes were collected 44–48 hours after PMSG injection, and MII oocytes were collected 12–14 hours after hCG injection.

Collection of parthenogenetic embryos was performed following a previously reported protocol [19]. In brief, oocytes surrounding the cumulus cells were recovered from female C57BL/6 J mice oviducts after 16–18 hours post-hCG administration. When the cumulus cells were still attached to the oocytes, they were discharged into 7% ethanol freshly prepared in M2 media and permitted to rest for 5 minutes at room temperature. Then, the oocytes were cleaned 3 times with M2 media in culture dishes, and cumulus cells were eliminated using a hyaluronidase treatment (1 mg/mL, dissolved in M2 media). Next, oocytes were transferred into drops of M16 medium (containing cytochalasin B, 5 µg/mL) and incubated for 4–5 hours at 37°C under 5% CO_2_ (suppressing the emission of the second polar body and predominantly developing diploid parthenogenetic embryos). Pronucleus formation was observed under the microscope, and activated oocytes with double pronucleus were transferred to KSOM medium for further culture. Two-cell stage embryos were collected after 22–24 hours, 4-cell to morula stage embryos were collected after 2–3 days, and the blastocyst stage was collected at day 4.

To obtain fertilized embryos, super-ovulated female mice were mated with male mice. The zygotes (pronucleus stage) were extracted from the female C57BL/6 J oviducts at 24 hours post-hCG injection. The zygotes were then transferred into a KSOM medium and incubated at 37°C under 5% CO_2_. The 2-cell stage fertilized embryos were collected after 22–24 hours. The fertilized embryos at the 4-cell to morula stages and blastocyst stage were collected 2–3 days after incubation and at day 4 after incubation, respectively.

### Quantitative MS

Label-free quantitative MS was performed on parthenogenetic embryos (pronucleus, 2-cell, 4-cell, 8-cell, morula, and blastocyst stages). For each stage, 6,000 embryos were used in 3 biological duplicates. The lysis buffer (4% sodium dodecyl sulfate [SDS], 100 mM Tris-HCl, 1 mM Dithiothreitol (DTT), pH 7.6) was used to lyse the samples and extract the proteins. The BCA protein assay kit was used to quantify proteins (Bio-Rad, Hercules, USA). Trypsin digestion was conducted in accordance with the standards for filter-aided sample preparation as previously described [77]. Briefly, around 100 µg total protein was collected from each specimen and then added into 30 µL SDS-DTT-Tris (SDT) buffer (100 mM DTT, 150 mM Tris-HCl, 4% SDS, pH 8.0). Repeated ultrafiltration (Microcon units, 10 kD) was used to filter out the DDT, detergent, and other low-molecular-weight substances using Urea (UA) buffer (8 M urea, 150 mM Tris-HCl, pH 8.0). To inhibit decreased cysteine residues, 100 µL iodoacetamide (100 mM iodoacetamide in UA buffer) was introduced into the specimens and incubated for 30 minutes in complete darkness. Subsequently, the filters were rinsed thrice using 100 µL UA buffer and rinsed 2 times using 100 µL 25 mM NH_4_HCO_3_ buffer. Last, 4 µg trypsin (Promega, USA) was used for digesting the protein suspensions overnight at 37°C in 40 µL 25 mM NH_4_HCO_3_ buffer; the obtained peptides were extracted as a filtrate. Peptides from each sample were desalted on a C18 cartridge (Empore™ SPE Cartridge C18 [standard density], bed I.D. 7 mm, volume 3 mL; Sigma-Aldrich, USA), followed by concentration using vacuum centrifugation and reconstitution in 40 µL 0.1% (v/v) formic acid. Then, liquid chromatography coupled to MS (LC-MS/MS) analysis was performed over 120 minutes using the Q Exactive mass spectrometer (Thermo Scientific, USA) coupled to an EASY-nLC (Proxeon Biosystems, Thermo Fisher Scientific). A reverse-phase trap column (100 µm × 2 cm, nanoViper C18, Thermo Scientific Acclaim PepMap100) connected to a C18 reverse-phase analytical column (10 cm long, 75 µm inner diameter, 3 µm resin; Thermo Scientific Easy Column) in buffer A (0.1% formic acid) was used to load the peptides, which were then isolated using buffer B (0.1% formic acid and 84% acetonitrile) with a linear gradient, at a flow rate of 300 nL/min regulated by IntelliFlow technology. The mass spectrometer positive was set in ion mode. By using the data-dependent technique, MS data were obtained via the dynamic selection of the most abundant precursor ions identified by the survey scan (300–1,800 *m/z*) for fragmenting higher energy collisional dissociation (HCD). 3e6 was chosen as the automatic gain control (AGC) target and 10 ms adjusted as a maximum injection duration. The duration of the dynamic exclusion was adjusted to 40 seconds. The scan resolution was 70,000 at 200 *m/z*, whereas HCD spectra were observed using a resolution of 17,500 at 200 *m/z*, with an isolation width of 2 *m/z*. The normalized collision energy was adjusted to 30 eV while the underfill ratio, which sets the minimum percentage of the target value achieved within the maximum fill time, was specified as 0.1%.

### Identification and bioinformatic analysis

The MaxQuant software (Max Planck Institute of Biochemistry, Martinsried, Germany; version: 1.5.3.17; RRID:SCR_014485) was used to analyze the raw MS data. Protein detection in the MS/MS spectra was accomplished by comparing the spectra to the Swiss-Prot mouse database (UP000000589). The following parameters were used: the enzyme chosen was trypsin; the maximum number of missed cleavages was 2; for fixed modifications, carbamidomethyl was chosen; for variable modification, oxidation was chosen; 6 ppm was chosen for the main search; and 20 ppm was chosen for the first search and MS/MS tolerance. The database analysis included the following patterns: the included contaminants term was considered true, protein and peptide false discovery rates were <1%, razor and unique peptides were used in the protein quantitation, a 2-minute interval existed between runs (match between runs), the protein quantification technique was label-free quantification (LFQ), and 1 was set as the minimum number of ratios. The proteomics data from MS have been submitted to the ProteomeXchange Consortium via the iProX partner repository [78], with the dataset identifier PXD029532. Blast2GO program was used to map and annotate sequences containing GO terms [79]. R scripts were used to visualize the results of the GO annotation. Subsequently, the investigated proteins were compared with the KEGG database [80] to get their KEGG ortholog identifications. The PPI of the investigated proteins was determined using STRING (RRID:SCR_005223) [81]. The interaction files were visualized by Cytoscape (RRID:SCR_003032; version 3.2.1) [82].

### Quantitative analysis of selected proteins using parallel reaction monitoring

Selected proteins were verified by PRM at the protein level. A total of 2,500 fertilized embryos of each sample at the 6 stages (pronucleus, 2-cell, 4-cell, 8-cell, morula, blastocyst) were acquired for protein extraction. Mass shotgun analysis was performed to obtain preexperimental results and used to select suitable peptides for PRM analysis. Briefly, to desalt tryptic peptides, they were placed onto a C18 analytical column (Thermo Scientific) prior to reversed-phase chromatography on the EASY-nLC™ 1200 system. Gradients of acetonitrile ranging between 5% and 35% in 45 minutes were used in 1-hour LC. A Q Exactive™ Plus Hybrid Quadrupole-Orbitrap™ Mass Spectrometer was used to perform the PRM analysis. The raw data were examined using the Skyline 3.5.0 software (MacCoss Lab, University of Washington; RRID:SCR_014080) [83]. The PRM validation results of selected proteins are shown in Supplementary Table S11. The proteomics data from MS have been submitted to the ProteomeXchange Consortium via the iProX partner repository [78], with the dataset identifier PXD029532.

### Fbxw24 knockdown in early embryos

siRNAs of mouse *Fbxw24* and negative control were diluted to a 5-mM final concentration using nuclease-free water. Using a Piezo-driven micromanipulator, about 10 pL of 5-mM siRNAs were delivered into the oocytes. Then, the implanted oocytes were cultured for ≥3 hours to prepare for ICSI. About 1 mL of a sperm suspension was combined with the HEPES-buffered Chatot–Ziomek–Bavister (HCZB) medium drop comprising of 10% (w/v) polyvinylpyrrolidone (Irvine Scientific, Santa Ana, CA, USA). Using several Piezo pulses, the sperm head and tail were separated and the head was then injected into the oocyte according to the procedure by Ward and Yanagimachi [84]. HCZB medium was used for gamete handling and ICSI, whereas Chatot–Ziomek–Bavister (CZB) medium was used for embryo culturing at 5% CO_2_. For embryo culture, CZB was overlaid with sterile mineral oil (Sigma-Aldrich). To analyze siRNA knockdown efficiency, the total RNA of 15 embryos at the 4-cell stage was purified by the RNeasy Mini Kit (QIAGEN, Germany, cat. 74,104). Reverse transcription was used to synthesize complementary DNA (cDNA) (Promega). The StepOneTM Real-Time PCR System (Applied Biosystems, USA) was used to perform quantitative real-time PCR, using *H2afz* as internal control. Primer sequences and siRNA-targeting sequences are shown in Supplementary Table S9.

### Measuring mRNA levels using real-time PCR

The isolation of total RNA from embryos was carried out at 6 stages (pronucleus, 2-cell, 4-cell, 8-cell, morula, blastocyst) using the RNeasy Mini Kit (QIAGEN, cat. 74,104). Reverse transcription technique (Promega) was used to synthesize cDNA. The Real-Time PCR System (Applied Biosystems) was used to perform quantitative real-time PCR analysis. The relative expression levels were calculated using the 2^–△△CT^ method. All experiments were carried out in 3 biological repetitions. The primer sequences are shown in Supplementary Table S9.

### Immunoblotting, immunofluorescence, and immunohistochemistry analysis

For immunoblotting analysis, the different tissues were obtained from C57BL/6J-*Fbxw24*^em(Linker-3xFlag)^ mice and the extracted proteins were isolated using sodium dodecyl sulfate–polyacrylamide gel electrophoresis (SDS-PAGE). Using a semidry Western blotting technology (Trans-Blot® TurboTM System; Bio-Rad, Singapore), the proteins were then deposited onto a polyvinylidene difluoride membrane. Then, the membrane was blocked with 5% nonfat milk for 1 hour at 37°C before using the primary antibodies and incubated at 4°C overnight. The membrane was cleaned 3 times using a solution composed of Tris-buffered saline and Tween 20 (TBST buffer), followed by incubation with the secondary antibodies in TBST for 1 hour at 37°C. An alkaline phosphatase detection kit (C3206; Beyotime Biotechnology, Inc., Shanghai, China) was used to identify the presence of proteins.

For immunofluorescence analysis, C57BL/6J-*Fbxw24*^em(Linker-3xFlag)^ female mice oocytes or embryos were kept at room temperature for 30 minutes in 4% polyoxymethylene. In addition, the embryos were placed into a 1% Triton X-100 phosphate-buffered saline (PBS) solution for 20 minutes, followed by a solution of PBS comprising 1% bovine serum albumin (BSA) for blocking. Next, embryos were incubated overnight at 4°C, followed by combination with a primary antibody at an effective concentration. Then, embryos were washed 3 times with a PBS solution containing 0.1% Tween-20 and 0.01% Triton X-100 for 2 minutes each. The embryos were incubated at room temperature in a diluted solution of a secondary antibody (fluorescein isothiocyanate labeled) and washed thrice for 2 minutes each with a PBS solution containing 0.1% Tween-20 and 0.01% Triton. The specimen was covered using a coverslip coated in a ProLong Gold antifade reagent containing DAPI (Life Technologies, USA) and stored in darkness until fluorescence was determined using an inverted fluorescence microscope (Olympus, IX73, Tokyo, Japan). Fluorescence intensity was measured using ImageJ (National Institutes of Health, Bethesda, MD, USA; RRID:SCR_003070).

For immunohistochemistry analysis, C57BL/6J-*Fbxw24*^em(Linker-3xFlag)^ female mouse ovaries were fixed in 4% paraformaldehyde, and paraffin was used to embed them. The ovarian sections (5 µm) were deparaffinized using xylene and rehydrated in serial ethanol dilutions. The sections were rinsed in 1% PBS-Tween-20 and treated using 2% hydrogen peroxide. The incubation with specified primary antibodies lasted 2 hours at room temperature after blocking using 3% goat serum. The sections were treated using the secondary antibody for 40 minutes at 37°C. Negative controls were processed with PBS instead of the primary antibody. Sections were analyzed with a light microscope (IX73; Olympus). The following primary antibodies were used: anti-beta anti-GAPDH rabbit monoclonal antibody (ab181602; Abcam, USA) and anti-DDDDK tag (Binds to FLAG® tag sequence) rabbit monoclonal antibody (ab205606; Abcam).

### Proteasome activity assay

Proteasome activity assays were performed as previously reported with minor modifications [85]. To assess oocyte proteasome activity at 6 embryonic development stages of mouse biparental and uniparental embryos, 100 embryos per group were collected and washed 3 times in PBS/polyvinyl pyrrolidone (PVP). All cell suspensions were lysed in 20 µL protein extraction buffer composed of 150 mM sodium chloride, 50 mM Tris, and 0.5% Triton X-100 for 30 minutes under constant rotation at 4°C. After centrifugation at 16,300 g for 15 minutes, the supernatants were transferred to a clean tube and assessed for proteasome activity using a commercial proteasome assay kit, which uses an AMC-tagged peptide substrate, which releases free, highly fluorescent AMC in case of proteolytic activity (ab107921; Abcam). In brief, 10 µL of each sample was loaded into a 96-well plate in duplicate, alongside a Jurkat cell lysate-positive control (supplied) and AMC protein standards. A total of 50 µM of the proteasome inhibitor MG132 was added to 1 well of each sample to differentiate proteasome activity from other protease activity in the samples. Plates were incubated for 25 minutes and analyzed on a TECAN Sunrise™ plate reader (TECAN, Salzburg, Austria) at 350/440 nm excitation/emission. After a further 35-minute incubation at 37°C, plates were analyzed a second time to calculate the change in relative fluorescence units in each sample. Data were analyzed following the manufacturers’ instructions, and proteasome activity was calculated such that 1 unit of proteasome activity is equivalent to the amount of proteasome activity that generates 1.0 nmol of AMC per minute at 37°C. This experiment was repeated across 3 independent biological and technical replicates using 100 embryos per assay.

### SILAC-IP-MS

Following SILAC labeling [86, 87], HEK-293T cells were maintained in Dulbecco's modified Eagle's medium (DMEM) containing 13C6-Lysine (K6) and 13C6-Arginine (R6) (Cambridge Isotope Laboratories, USA), supplemented with 10% dialyzed fetal bovine serum (Invitrogen, USA) for 7 passages for complete labeling of the cellular proteome. The mouse Fbxw24 gene (NM_001013776) was cloned into a p3×FLAG-CMV-7.1 vector (Sigma-Aldrich) between the NotI and SalI sites and transfected into HEK-293T cells maintained in normal DMEM (light, L) using Lipofectamine 2000 (Invitrogen). The empty vector p3×FLAG-CMV-7.1 was transfected into K6R6-labeled HEK-293T cells as control (heavy, H). Then, 20 µM MG132 (Selleckchem, Houston, USA) was added to the transfection solution for 6 additional hours before collection. The collected cells from the L and H groups were lysed using 150 mM NaCl, 20 mM Tris (pH 7.5), and 2% Triton X-100 supplemented with a phosphatase and protease inhibitor cocktail. Anti-FLAG beads (Sigma-Aldrich) were added for IP, and the enriched proteins were eluted by 3×FLAG peptide (Sigma-Aldrich). The final eluates obtained after Flag peptide elution were analyzed by SDS-PAGE. Coomassie brilliant blue dye was used for staining, and visible bands were excised for LC-MS/MS analysis. Peptide extraction and in-gel protein trypsin digestion were carried out according to the previously described protocol [88]. Analysis of peptide samples was performed on an EASY-nLC 1000 system (Thermo Fisher Scientific, Waltham, MA, USA) connected to an Orbitrap Fusion mass spectrometer (Thermo Fisher Scientific, San Jose, CA, USA). Then, 10 µL solvent A (water comprising 0.1% formic acid) was used to resuspend the peptide, followed by loading of 8 µL peptide sample onto a trap column (100 µm × 2 cm; Thermo Scientific Acclaim PepMap C18) for 3 minutes at a flow rate of 10 µL/min, followed by separation on an analytical column (Acclaim PepMap C18, 75 µm × 25 cm) with a linear gradient. The flow rate inside the column was maintained at 300 nL/min. Using the data-dependent method, the Orbitrap Fusion mass spectrometer switched automatically between MS and MS/MS acquisition. The Orbitrap was used to acquire survey full-scan MS spectra (*m/z* 350–1,600) with 120,000 mass resolution, 1,000,000 AGC, and a 50-ms utmost duration for injection. MS/MS acquisition was performed in an Orbitrap at maximum speed mode and a cycle period of 3 seconds, with mass resolution of 15,000, AGC target of 100,000, utmost injection duration of 80 ms, and isolation width of 1.6 *m/z*. HCD was used to break down ions with charge states 2+, 3+, and 4+ consecutively, with 30% normalized collision energy. Microscans were recorded using dynamic exclusion for 30 seconds in all cases. Maxquant software (version 1.5.2.8) [89] was used to process the MS raw data for protein identification and quantitation. We employed the Andromeda search engine [90] to search for relevant data in mouse UniProtKB/Swiss-Prot databases. The following criteria were established: (i) required peptide length ≥7 amino acids and (ii) trypsin cleavage specificity, with a maximum of 2 missing cleavages permitted. (iii) Oxidation (M) and acetylation (protein N-term) were the 2 variable modifications considered. (iv) For both precursor and fragment ions, the initial mass variation was as high as 10 ppm and 5 Da, respectively. (v) At both protein and peptide levels, the false discovery rate was set at 1%. (vi) A multiplicity of 2 was used, where 13C6-Lysine (Lys6, K6) and 13C6-Arginine (Arg6, R6) were selected as heavy (H) labels. (vii) A “requantify” option was selected. (viii) Quantification was completed with unmodified unique and razor peptides, as well as a minimum of 2 counted ratios. (ix) Any protein that sequenced ≥2 peptides was considered a reliable indentation. Statistical analysis of specific interactions was evaluated using significance B (*P* < 0.05), by Perseus (version 1.6.7.0; RRID:SCR_015753) [91] on the log_2_ L/H ratio. The significance B value indicates whether a ratio differs from the distribution of all protein ratios grouped by intensity. The proteomics data from MS have been submitted to the ProteomeXchange Consortium via the iProX partner repository [78], with the dataset identifier PXD029532.

### IP and Western blotting

We used 5 µg anti-DDDDK tag rabbit monoclonal antibody to coat magnetic Beads Protein G (binds to the FLAG tag sequence) in IP wash buffer (50 nM Tris-HCl, 150 nM sodium chloride, 0.05% NP-40, and 1 mM MgCl_2_, pH 7.4) with 5% BSA and incubated overnight at 4°C with rotation. HEK-293T cells (in normal DMEM) and C57BL/6J-*Fbxw24*^em(Linker-3xFlag)^ HO mouse ovaries were added to IP lysis buffer (50 mM Tris-HCl, 150 mM NaCl, 1 mM EDTA, 0.1% SDS, 0.5% sodium deoxycholate, 1% NP-40, 1 mM Phenylmethanesulfonyl fluoride (PMSF)/cocktail and 0.5 mM DTT, pH 7.4), followed by a 10-minute incubation on ice. HEK-293T cells (in K6R6-labeled DMEM) and ovaries of wild-type littermates were used as control, respectively. Then, the IP lysate was centrifuged at 14,000 rpm and 4°C for 10 minutes; 100 µL supernatant was removed and mixed with 900 µL of the beads–antibody complex in IP buffer (35 µL 0.5 M EDTA and 860 µL IP wash buffer), followed by overnight incubation with 4°C rotation. After rinsing, 50 mL elution buffer was added into the immunoprecipitate and the supernatant used for Western blot. Four commercial antibodies were employed: anti-Ddb1 antibody (ab109027; Abcam), anti-Cul4b antibody (12916–1-AP; ProteinTech Group, Wuhan, China), anti-DDDDK tag (Binds to FLAG^®^ tag sequence) rabbit monoclonal antibody (Binds to FLAG® tag sequence) (ab205606; Abcam), and anti-beta anti-GAPDH rabbit monoclonal antibody (ab181602; Abcam).

### Statistical analysis

Statistical analyses were conducted using the IBM Statistical software (SPSS 23.0; SPSS, Inc., Chicago, IL, USA). The mean ± standard error of the mean was used to express the data. Either a 1-way analysis of variance or an unpaired *t*test with Tukey's post hoc test was used for comparison of the statistical data (*P < 0.05).

## Data Availability

The mouse uniparental embryo proteome that was constructed in this study (deposited via the iProX partner repository) is available via the following accession number in ProteomeXchange: PXD029532. All other supporting data and materials are available in the *GigaScience* GigaDB database [92].

## Additional Files

**Supplementary Table S1**. Summary of maternal protein and peptide profiles identified in mouse uniparental embryos.

**Supplementary Table S2**. The detailed information of UpSet intersection diagram and the annotation of each intersection for the proteome of mouse uniparental embryos.

**Supplementary Table S3**. The detailed results of fuzzy c-means clustering analysis using the proteome of mouse uniparental embryos.

**Supplementary Table S4**. The detailed results of a Venn diagram in mouse mature oocytes (MII), fertilized embryos (ZY), and parthenogenetic (PA) embryos.

**Supplementary Table S5**. The detailed results of fuzzy c-means clustering analysis related to Fig. 3.

**Supplementary Table S6**. The results of expression correlation for 15 key maternal proteins and other quantified proteins in PA and ZY groups.

**Supplementary Table S7**. The maternal proteins that are strongly correlated with SCMC components in PA and ZY groups.

**Supplementary Table S8**. The detailed results of a Venn diagram related to Fig. 5A.

**Supplementary Table S9**. Primers for real-time PCR and target sequence of siRNAs.

**Supplementary Table S10**. Putative Fbxw24 interactors identified by Fbxw24 IP-MS.

**Supplementary Table S11**. The parallel reaction monitoring (PRM) validation results of selected proteins.

**Supplementary Fig. S1**. (A) The developmental morphology of different stages after activation of ethanol combined with Cytochalasin B (CB). The visualization of DNA was performed using DAPI (blue). Scale bar: 20 µm. (B) The developmental rate after activation of ethanol combined with CB in mouse oocyte.

**Supplementary Fig. S2**. Annotation for maternal protein expression clusters in mouse uniparental embryos. (A) The heatmap shows the significance value of the biological processes in 10 clusters. (B–D) The interaction networks of GO terms from 4 categories are displayed, including “translation” (B), “peptide metabolic process” (C), “nucleic acid metabolic process” (D), and “cellular metabolic process” (E).

**Supplementary Fig. S3**. (A) The protein–protein interaction (PPI) network of the candidate maternal proteins that are strongly negative with the SCMCs. (B) The GO and KEGG annotation for the candidate maternal proteins that are strongly negative with the SCMCs.

**Supplementary Fig. S4**. (A) The Venn diagram shows the intersection of the candidate proteins that strongly correlated with 15 key maternal proteins in 2 reported proteome databases of fertilized embryos identified by *Gao et al*. and *Israel etal*., respectively. (B–D) The UpSet intersection diagram of 15 key maternal proteins and their candidate proteins (|*r*| ≥ 0.70 and *P*≤ 0.05) in the PA group (B) and ZY group (C, identified by *Gao et al*.; D, identified by *Israel et al*.).

**Supplementary Fig. S5**. The presence of Fbxw24 protein in oocytes. The oocytes of GV and MII stages were collected from the C57BL/6J-*Fbxw24*^em(Linker-3xFlag)^ mice. Each sample was performed using DAPI to allow for the visualization of DNA (blue). Scale bar: 20 µm. Germinal vesicle (GV); Metaphase II (MII).

giac084_GIGA-D-22-00094_Original_Submission

giac084_GIGA-D-22-00094_Revision_1

giac084_Response_to_Reviewer_Comments_Original_Submission

giac084_Reviewer_1_Report_Original_Submission Saffet Ozturk -- 5/10/2022 Reviewed

giac084_Reviewer_2_Report_Original_Submission Michele Boiani -- 6/2/2022 Reviewed

giac084_Reviewer_2_Report_Revision_1 Michele Boiani -- 7/7/2022 Reviewed

giac084_Supplemental_Figures_and_Tables

## Ethics Approval

All experimental protocols were reviewed and approved by the Animal Care and Use Committee of Guangxi Medical University (No. 201901012).

## Competing Interests

The authors declare that they have no competing interests.

## Funding

This study was funded by China Postdoctoral Science Foundation (2019M653810XB), Guangxi Natural Science Foundation (2019JJB140131), and Guangxi First-Class Discipline Project for Basic Medicine Sciences (No.GXFCDP-BMS-2018).

## Authors' Contributions

F.C., X.L., T.X., and Y.Z. performed the experiments. B.M., Y.L., F.C., Y.L., and Y.Z. supervised and analyzed experimental data. F.C., Q.Z., B.L., and X.X. wrote the manuscript.
