# Supplementary material for: Comparative maternal protein profiling of mouse biparental and uniparental embryos
Source: Gigascience. 2022 Sep 3;11:giac084. doi: 10.1093/gigascience/giac084 (PMC9440387; doi:10.1093/gigascience/giac084)
Supplement: giac084_GIGA-D-22-00094_Original_Submission [file giac084_giga-d-22-00094_original_submission.pdf]

GigaScience

Comparative maternal protein profiling of mouse biparental and uniparental embryos

--Manuscript Draft--

**Manuscript Number:**	GIGA-D-22-00094
**Full Title:**	Comparative maternal protein profiling of mouse biparental and uniparental embryos
**Article Type:**	Research
**Funding Information:**	China Postdoctoral Science Foundation (2019M653810XB) — Dr Fumei Chen<br>Guangxi First-class Discipline Project for Basic medicine Sciences (GXFCDP-BMS-2018) — Dr Fumei Chen<br>Guangxi Natural Science Foundation Program (2019JJB140131) — Dr Fumei Chen
**Abstract:**	Background: During early embryonic development, maternal proteins act as important roles. However, our cognition of maternal proteins is still very limited. The integrated analysis of mouse uniparental (parthenogenetic) and biparental (fertilized) embryos in protein level may provide us more information for maternal proteins. Although proteome of mouse fertilized embryos has been reported, the protein expression landscape of mouse parthenogenesis remains unexplored.<br>Results: Using label-free quantitative mass spectrometry (MS) analysis, we firstly reported the maternal proteome of mouse parthenogenetic embryos (pronucleus, 2-cell, 4-cell, 8-cell, morula and blastocyst) and showed its dynamic changes. Then the combined comparison of proteins profile for parthenogenesis and fertilized embryos shown the different fates of maternal proteins. We enriched a group of maternal proteins that are strongly correlated with the subcortical maternal complex (SCMC), and found some maternal proteins may escape from the fate of degradation in mouse parthenogenesis. Moreover, we identified a new maternal factor (Fbxw24) and showed its importance in early embryonic development. Especially, we found that Fbxw24 interact with Ddb1-Cul4b and may regulate maternal degradation in mouse.<br>Conclusions: Our study provides a valuable resource for further mechanistic studies of maternal proteins, and suggests a new maternal factor regulating pre-implantation embryo development.
**Corresponding Author:**	Fumei Chen<br>Guangxi Medical University<br>Nanning, CHINA
**Corresponding Author Secondary Information:**	
**Corresponding Author's Institution:**	Guangxi Medical University
**Corresponding Author's Secondary Institution:**	
**First Author:**	Fumei Chen
**First Author Secondary Information:**	
**Order of Authors:**	Fumei Chen
	Buguo Ma
	Yongda Lin
	Xin Luo
	Tao Xu
	Yuan Zhang
	Fang Chen
	Yanfei Li

Powered by Editorial Manager® and ProduXion Manager® from Aries Systems Corporation

	Yaoyao Zhang
	Bin Luo
	Qingmei Zhang
	Xiaoxun Xie
**Order of Authors Secondary Information:**	
**Additional Information:**	
**Question**	**Response**
Are you submitting this manuscript to a special series or article collection?	No
**Experimental design and statistics**<br><br>Full details of the experimental design and statistical methods used should be given in the Methods section, as detailed in our Minimum Standards Reporting Checklist. Information essential to interpreting the data presented should be made available in the figure legends.<br><br>Have you included all the information requested in your manuscript?	Yes
**Resources**<br><br>A description of all resources used, including antibodies, cell lines, animals and software tools, with enough information to allow them to be uniquely identified, should be included in the Methods section. Authors are strongly encouraged to cite Research Resource Identifiers (RRIDs) for antibodies, model organisms and tools, where possible.<br><br>Have you included the information requested as detailed in our Minimum Standards Reporting Checklist?	Yes
**Availability of data and materials**<br><br>All datasets and code on which the conclusions of the paper rely must be either included in your submission or	Yes

Powered by Editorial Manager® and ProduXion Manager® from Aries Systems Corporation

deposited in publicly available repositories (where available and ethically appropriate), referencing such data using a unique identifier in the references and in the "Availability of Data and Materials" section of your manuscript.<br><br>Have you have met the above requirement as detailed in our Minimum Standards Reporting Checklist?	

Revised Manuscript
Click here to access/download;Manuscript;Revised Manuscript.docx

Comparative maternal protein profiling of mouse biparental and uniparental embryos

Fumei Chen[1], Buguo Ma[1,2], Yongda Lin[1,2], Xin Luo[1], Tao Xu[1], Yuan Zhang[1], Fang Chen[1,2], Yanfei Li[1,2], Yaoyao Zhang[1,2], Bin Luo[1,2,], Qingmei Zhang[1,2,*], Xiaoxun Xie[1,2,*]

[1]Department of Histology and Embryology, School of Pre-Clinical Medicine, Guangxi Medical University, Nanning, Guangxi, P. R. China

[2]Central Laboratory, School of Pre-Clinical Medicine, Guangxi Medical University, Nanning, Guangxi, P. R. China

E-mail addresses for all authors:

Fumei Chen: gxchenfumei@163.com

Buguo Ma: mabuguo@gxmu.edu.cn

Yongda Lin: linyongda@hotmail.com

Xin Luo: 295268654@qq.com

Tao Xu: 1031895151@qq.com

Yuan Zhang: 13033466195@163.com

Fang Chen: 756007230@qq.com

Yanfei Li: 1195220977@qq.com

Yaoyao Zhang: 1575762156@qq.com

Bin Luo: glbinbin2002@yahoo.com

*Correspondence:

*Qingmei Zhang: zhangqingmei2017@outlook.com

*Xiaoxun Xie: xiaoxunxie@hotmail.com

Abstract

Background: During early embryonic development, maternal proteins act as important roles. However, our cognition of maternal proteins is still very limited. The integrated analysis of mouse uniparental (parthenogenetic) and biparental (fertilized) embryos in protein level may provide us more information for maternal proteins. Although proteome of mouse fertilized embryos has been reported, the protein expression landscape of mouse parthenogenesis remains unexplored.

Results: Using label-free quantitative mass spectrometry (MS) analysis, we firstly reported the maternal proteome of mouse parthenogenetic embryos (pronucleus, 2-cell, 4-cell, 8-cell, morula and blastocyst) and showed its dynamic changes. Then the combined comparison of proteins profile for parthenogenesis and fertilized embryos shown the different fates of maternal proteins. We enriched a group of maternal proteins that are strongly correlated with the subcortical maternal complex (SCMC), and found some maternal proteins may escape from the fate of degradation in mouse parthenogenesis. Moreover, we identified a new maternal factor (Fbxw24) and showed its importance in early embryonic development. Especially, we found that Fbxw24 interact with Ddb1-Cul4b and may regulate maternal degradation in mouse.

Conclusions: Our study provides a valuable resource for further mechanistic studies of maternal proteins, and suggests a new maternal factor regulating pre-implantation embryo development.

Keywords: Maternal protein; Parthenogenesis; Early embryo; Proteome; Mouse

Background

During oogenesis, proteins from the oocyte genome (maternal genome) are largely accumulated, with important roles in fertilization and early embryonic development, including the degradation of proteins, activation of the embryonic genome, epigenetic modifications and cell signal transduction [1]. Various studies report that the depletion or abnormal expression of maternal proteins not only affects embryo development, but can even lead to embryo death [2-8]. The analysis of maternal proteins is helpful to deepen our understanding of the regulatory mechanism of embryonic development.

Although many studies of mammalian preimplantation development rely on transcriptomic data [9, 10], protein databases of embryos provide invaluable information for the study of maternal proteins. Protein abundances, which are closer to the phenotype, show more predictive value than mRNAs. There have been cases of anti-correlation in which the mRNA is rapidly degraded after fertilization, whereas proteins persist throughout the blastocyst stage, and may be candidate maternal proteins [11].

Quantitative proteomics is an effective strategy to construct protein databases for gametes or embryos in different species. There have been some protein databases of early embryos constructed by mass spectrometry (MS) strategies, including bovine [12], zebrafish [13], and Xenopus [14, 15]. In addition, mouse oocyte and embryo proteomes have been quantified [11, 16-18]. Wang et al. collected 7,000 mouse

oocytes at different developmental stages, including the germinal vesicle stage, the metaphase II (MII) stage and fertilized oocytes (zygotes), and they successfully identified 2,781 proteins present in germinal vesicle oocytes, 2,973 proteins in MII oocytes, and 2,082 proteins in zygotes through semiquantitative MS analysis [16]. Gao et al. collected each stage embryo consisting of 8,000 and identified nearly 5,000 proteins across six developmental stages (from the zygote to the blastocyst) by the tandem mass tag (TMT) labelled method, which they performed in biological duplicates. In total, 4,608 and 4,590 proteins were quantified in each of the two experiments, and the two experiments had 3,767 proteins in common [17]. Israel et al. collected and processed a total of ~12,600 oocytes or embryos, in three biological replicates of ~600 oocytes/embryos per developmental stage: unfertilized oocytes, fertilized oocytes with pronuclei, and preimplantation embryos at the 2-, 4-, 8-cell, advanced morula and blastocyst stages. Their detected proteome was comprised of 6,550 proteins identified by stable isotope labelling with amino acids in cell culture (the SILAC method). Among the detected proteins, 5,217 proteins were detected in at least two replicates of one or more developmental stages, and 1,709 proteins were detected in both replicates of all developmental stages [11].

Mouse parthenogenesis is a well-developed model to explore embryo development [19]. Under natural conditions, an oocyte can be activated without the intervention of the male counterpart. This form of reproduction, known as parthenogenesis, occurs spontaneously in various lower organisms [20, 21]. In mammals, oocytes can be

activated using different methods, including high or low temperature, electrical or chemical treatment [22]. The rates of oocyte activation depend on various factors, including species, female age and culture conditions [22]. Also, methods of inducing artificial oocyte activation are vital for somatic cell nuclear transfer (SCNT) research [23]. Although some transcriptomic studies have enriched our understanding of the genetic programs underlying mammal parthenogenesis [24-30], the protein database of parthenogenetic embryos has not been reported yet. The comparative analysis of two types of embryos (biparental and uniparental embryos) may be helpful to obtain more valuable information about maternal proteins, as parthenogenetic embryos only represent the information of the maternal genome, while fertilized embryos represent the information of the biparental genome.

In this study, we made mouse uniparental embryos that were activated artificially and cultured in vitro as a source of embryonic material for proteomic analysis, to identify dynamic changes in maternal proteins during early embryonic development from the pronucleus to the blastocyst stage. A comparative analysis of protein expression was performed in mature oocytes, fertilized embryos and parthenogenetic embryos. We also conducted an enrichment analysis of maternal proteins that are strongly correlated with the subcortical maternal complex (SCMC) components, and we found a group of maternal proteins that may escape from degradation in mouse parthenogenesis. Additionally, identification and functional analyses were performed for a new key maternal factor, Fbxw24. Our uniparental embryo proteomic database is

the first complete parthenogenetic embryo proteome of a mammalian species characterized to date. This proteome dataset enables a more direct investigation of mammalian developmental processes that are regulated by the maternal genome at the protein level, and the measurement of proteins using mouse parthenogenesis is an important complement to the proteomics of mammalian dynamic embryonic development.

Results

Definition and dynamics of maternal protein expression in mouse uniparental embryos

The protein expression profiles of six embryonic stages of mouse parthenogenesis, i.e. pronucleus (PA), 2-cell (PA2), 4-cell (PA4), 8-cell (PA8), morula (PAMO) and blastocyst (PABL), were detected by label-free quantitative mass spectrometry (MS). The developmental rate of parthenogenetic embryo and their morphology were shown in Supplementary Fig. S1. For each stage, 6,000 embryos were used, and the experiment was performed in three biological replicates. From three repetitions, 1,900, 1,944, and 1,960 proteins were identified. A total of 2,048 proteins were commonly detected in all three, of which 1,902 proteins were quantified (the quantified proteins refer to the proteins that could be detected in at least one embryonic stage and more than two biological replicates) (Fig. 1A and Supplementary Table S1). All identified proteins and peptides are shown in Supplementary Table S1.

Among the quantified proteins, 1,298 proteins were detected in all six successive developmental stages (Fig. 1B); however, few proteins were detected in only one stage. For example, 57 proteins were only detected in the PA stage (after oocyte activation) (Fig. 1B), including CCCTC binding factor, fcf1 rRNA processing protein, the general transcription factor IIF and polypeptide 1, which are involved in various biological processes including RNA processing, gene expression and nitrogen compound metabolic processes. Detailed information and the annotations of intersection groups are displayed in Supplementary Table S2. The hierarchical cluster analysis of quantified proteins revealed that the PA-4 and PA-8 embryos along with the PA-2 and PA embryos were closely clustered together. However, the morula and blastocyst were separated (Fig. 1C). These results are consistent with the principal component analysis (PCA), another clustering method (Fig. 1D). These results reveal that the blastocyst stage differed from the other stages during development, which is consistent with the corresponding analysis of fertilized embryos [11, 17]. In mouse biparental and uniparental embryos, a major dynamic change in proteins occurs in the blastocyst stage.

Fuzzy c-means analysis was then performed, and 10 distinct expression pattern clusters were found (Fig. 1E and Supplementary Table S3). Five known components of the subcortical maternal complex (SCMCs), a protein structure that is essential for pre-implantation development [3, 31], including Ooep, Tle6 and Padi6 (cluster 8 in

Fig. 1E), Nlrp5 (cluster 1 in Fig. 1E), and Zbed3 (cluster 4 in Fig. 1E) were detected in this study. This reveals that these maternal proteins are essential for early embryonic development in both mouse biparental and uniparental embryos. In addition, some N6-methyladenosine (m6A) readers, including Eif3a, Eif3b, Elavl1, Hnrnpa2b1, Hnrnpc, Igf2bp1 and Srsf2 were detected in this study (cluster 5 and cluster 6 in Fig. 1E) [32].

A cluster of proteins with high abundance was detected at the 2-cell stage (cluster 9 in Fig. 1E), including zinc finger protein 57 (Zfp57) and ring finger protein 2 (Rnf2). The absence of Zfp57 in oocytes results in the failure of maternal methylation imprinting at the Snrpn imprinted region; it is also required for the post-fertilization maintenance of maternal and paternal methylation imprints at multiple imprinted domains [33]. Rnf2 is a component of Polycomb-repressive complex 1 and functions as a redundant transcriptional factor during oogenesis, which is essential for proper zygotic genome activation (ZGA) [34].

A cluster of proteins with high abundance was detected at the 4-cell stage (cluster 4 in Fig. 1E), including nucleophosmin/nucleoplasmin 2 (Npm2), zinc finger and BED type containing 3 (Zbed3). Mouse Npm2 accumulates in oocyte nuclei and persists in preimplantation embryos. Npm2 knockout females have fertility defects owing to failed preimplantation embryo development [1].

A cluster of proteins with high abundance was detected at the 8-cell stage (cluster 7 in Fig. 1E), including developmental pluripotency-associated 3 (Dppa3) and mitochondrial transcription factor A (Tfam). Dppa3, also known as PGC7/Stella, protects the maternal genome from demethylation only after localizing to the nucleus, and is indispensable for the maintenance of methylation involved in epigenetic reprogramming after fertilization [35]. In zebrafish embryos, the knock down of Tfam, a regulator of mitochondrial DNA (mtDNA) replication, results in mtDNA copy number reduction and deficient oxidative phosphorylation [36].

A cluster of proteins with high abundance was detected at the morula stage (cluster 10 in Fig. 1E), including 2'-5' oligoadenylate synthetase 1D (Oas1d) and lysine (K)-specific demethylase 2B (Kdm2b). Mutant mice lacking Oas1d display a reduction in fertility due to ovarian follicle developmental defects, decreased ovulation efficiency and fertilization arrest at the one-cell stage [37]. Kdm2b, also known as Fbxl10 (F-box and leucine-rich repeat protein 10), is a JmjC domain-containing histone demethylase that contributes to embryonic neural development in mice by regulating cell proliferation and cell death [38].

Other proteins that play an important role in early embryonic development were also quantified in mouse uniparental embryos, including Filia [39] (cluster 1 in Fig. 1E), Dnmt1 [40] (cluster 2 in Fig. 1E), Eed [41] and Ezh2 [42] (cluster 5 in Fig. 1E), E-cadherin [43] and Brg1 [44] (cluster 6 in Fig. 1E), and Uhrf1 [45] (cluster 8 in Fig.

1E).

Gene ontology (GO) analysis of proteins from each cluster (Supplementary Fig. S2A) revealed that these proteins performed many functions, including protein folding, cellular localization, cellular component biogenesis and cellular metabolic process. These processes provide basic energy and materials for embryonic growth and development. The interaction networks of GO terms from four categories are displayed (Supplementary Fig. S2B-E), including ‘translation,’ ‘peptide metabolic process,’ ‘nucleic acid metabolic process’ and ‘cellular metabolic process’, with each network providing an elaborate view of the proteins participating in these biological processes.

The expression comparison of maternal proteins in mouse biparental and uniparental embryos

Maternal proteins have different fates after oocyte activation, including degradation or persistence. In this study, a comparison analysis was performed on the three proteome databases that were constructed using mature oocytes (MII group), fertilized embryos (biparental embryos, ZY group/ZY embryo) and parthenogenetic embryos (uniparental embryos, PA group/PA embryo, without the paternal genome). In order to eliminate the differences caused by different proteomic methods and to ensure the reliability of these comparison results, we used the intersection of two reported MII

oocyte protein databases [16, 18] as the MII group (2,209 proteins, Wang and Israel, Fig. 2A), and the intersection of two reported fertilized embryo proteome databases [17, 18] as the ZY group (3,218 proteins, Gao and Israel, Fig. 2A). The detailed results of the Venn diagram in Fig. 2A are shown in Supplementary Table S4.

A total of 1,029 proteins derived from mature oocytes were continuously detected during the preimplantation development of uniparental and biparental embryos (Fig. 2A), which indicates that the expression of these proteins may be independent from paternal genome regulation. We found that the expression changes of five SCMCs (Ooep, Nlrp5, Tle6, Zbed3 and Padi6) were similar in the three groups of embryo proteome databases (this study, Gao and Israel), which was also consistent with our verification results in fertilized embryos using the parallel reaction monitoring (PRM) assay (Fig. 2B), suggesting that the five components may play similar biological functions in both mouse biparental and uniparental embryos. Moreover, hierarchical clustering analysis of 1,029 proteins in uniparental embryos, the major protein expression change was found in the morula stage (PAMO) (Fig. 2C). Correspondingly, in biparental embryos, the major expression change was found in the blastocyst stage (ZYBL) (Fig. 2D and 2E), suggesting a regulation gap between the maternal genome and the embryonic genome at the protein level.

After artificial activation, 1,168 proteins stored in mature oocytes were also detected in uniparental embryos (Fig. 3A). Correspondingly, 1,806 proteins stored in mature

oocytes were also detected in biparental embryos (Fig. 3B). Compared to the biparental embryos, 613 proteins were only detected in uniparental embryos in this study (Fig. 3C). Furthermore, to reveal the expression dynamics of these proteins during development, Fuzzy c-means clustering was performed (Fig. 3A-C, and Supplementary Table S5). We found that two protein expression patterns in the uniparental embryos (PA group) and biparental embryos (ZY group) were similar. First, the level of some proteins peak at the blastocyst stage (cluster 6 in Fig. 3A; cluster 2-Gao and cluster 6-Israel in Fig. 3B), which accounts for the highest protein number compared to other clusters, followed by a gentle change in four early embryonic stages (PN, 2-cell, 4-cell, and 8-cell), then changed to a high level after the 8-cell stage (the beginning of compaction, Fig. 3D), including the proteins involved in mitotic cell cycle regulation (Tpr, Hnrnpu, Hmgb1, Mta3, Rpl17, Eif4g1, Lmnb1), and cell-cell junction organization (Itgb1, Cttn, Cdh1, Rcc2, Ctnna1, Actn4, Coro1c). Intriguingly, a cluster of maternal proteins that was detected only in uniparental embryos (PA group) in this study also followed this expression trend (cluster 1 in Fig. 3C), suggesting that embryonic compaction after the 8-cell stage is accompanied by protein expression changes both in biparental and uniparental embryos.

Second, the abundance of some proteins was highly detected in the pronucleus stage, and decreased during cleavage (the maternal to zygotic transition, Fig. 3D); their abundance was lowest at the morula or blastocyst stage (a maternal expression pattern, cluster 1 in Fig. 3A, cluster 1-Gao and cluster 5-Israel in Fig. 3B), including proteins

involved in the process of egg activation (Astl, Plat) and the post-transcriptional regulation of gene expression (Fxr1, Ddx6, Eif4enif1, Fxr2, Ybx2, Igf2bp2, Lsm14b, Lsm14a). A similar pattern was also detected in maternal proteins regulated by only the maternal genome (cluster 6 in Fig. 3C). This may provide the molecular basis for successful development beyond the 2-cell stage in uniparental embryos.

The enrichment of maternal proteins that are strongly correlated with SCMC components during preimplantation development

The subcortical maternal complex (SCMC) is a macromolecular complex with components encoded by maternal effect genes, and is mainly located in oocytes and early embryos; it is functionally conserved in mammals [1]. This complex is directly or indirectly involved in many important processes of early embryonic development, such as cell division, cytoskeleton and organelle rearrangement, maternal RNA regulation, and zygotic genome activation [1, 31]. Currently, the clarification of molecular mechanisms of the SCMC represents a milestone to understand the maternal regulatory network and oocyte biology in mammals [46, 47]. In addition, the SCMC can be used as a valuable reference for the identification of important mammalian maternal factors [3]. Expression correlation analysis also aids in analyzing current 'omic' data, which can screen out potential key proteins or genes. The establishment of embryo proteomes during embryonic development provides data support for such analyses.

As mentioned before, five components of the SCMC (Ooep, Nlrp5, Tle6, Zbed3 and Padi6, designated as SCMCs in this study) were detected and presented similar patterns in both mouse biparental and uniparental embryos (Fig. 2B). In this study, SCMCs were used as 'target proteins' to filter candidate maternal proteins based on their expression correlations with SCMCs. The relationship between SCMCs and other quantified proteins in the PA (uniparental embryos) and ZY (biparental embryos, including two protein databases identified by Gao and Israel) groups were analysed by Pearson's correlation coefficients (Supplementary Table S6). An absolute value of the correlation coefficient greater than or equal to 0.70 ($|r| \geq 0.70$ and $p \leq 0.05$) denotes a strong expression correlation [48]. Based on this criterion, there were 429 candidate proteins ($|r| \geq 0.70$ and $p \leq 0.05$) enriched in mouse biparental and uniparental embryos (Fig. 4A and Supplementary Table S7). Of these proteins, 113 were strongly positively correlated ($r \geq 0.70$ and $p \leq 0.05$, with the lowest protein level at the blastocyst stage) and 304 were strongly negatively correlated ($r \leq -0.70$ and $p \leq 0.05$, with the highest protein level at the blastocyst stage) (Fig. 4A, Fig. 4E and Supplementary Table S7).

Kyoto Encyclopedia of Genes and Genomes (KEGG) pathway analysis was conducted on 429 candidate proteins (Fig. 4B). These proteins were found to be widely involved in the regulation of many biological processes during embryonic development, including metabolism, genetic information processing, environmental

information processing and cellular processes. The protein-protein interaction (PPI) network of candidate proteins involved in ‘Translation’ was obtained using the STRING database (https://string-db.org/), which shows a complicated interaction protein network (Fig. 4C). To validate the expression trend of these candidate proteins, eight candidate proteins were randomly selected (highlighted with a red box in Fig. 4C) for the PRM assay using biparental embryos. The results reveal that the protein expression pattern coincided with the proteome results (Fig. 4D).

It should be pointed out that the number of strongly negatively correlated proteins (304 proteins, Fig. 4A and Supplementary Table S7) was about three times that of strongly positively correlated proteins (113 proteins, Fig. 4A and Supplementary Table S7). These strongly negatively correlated proteins have complex interaction networks (Supplementary Fig. S3A). After GO annotation, it was found that these proteins were mainly involved in translation initiation, mRNA transport and ribosome biogenesis, and these proteins were mainly located in the nucleus. The molecular functions of these proteins include translation initiation factor activity, protein activating ATPase activity, protein binding and mRNA binding (Supplementary Fig. S3B). In addition, the KEGG analysis results showed that these proteins were mainly involved in spliceosome, ribosome and proteome pathways (Supplementary Fig. S3B). These strong negatively correlated maternal proteins were mainly highly expressed in the blastocyst stage in both biparental and uniparental embryos (Fig. 4E), suggesting that these proteins may play an important role in the formation of the blastocoel and

the first differentiation of embryonic cells.

In this study, a group of candidate maternal proteins that strongly correlated with SCMCs was enriched. Their protein levels showed a similar trend in both biparental and uniparental embryos: the highest or the lowest expression level at the blastocyst stage (Fig. 4E). Obviously, their expression changes were not greatly affected by the paternal genome (or they may be regulated by the maternal genome alone). The definitive formation of two distinct cell lineages occurs at the blastocyst stage with the formation of the inner cell mass (ICM) and trophectoderm (TE) [49]. To some extent, this may explain why uniparental embryos can develop to the blastocyst stage and embryonic stem cells can be derived from both biparental and uniparental embryos. These maternal proteins may provide the molecular basis for these process, including cleavage and the formation of ICM and TE.

Some maternal proteins that should have been degraded may remain present in mouse uniparental embryos

Maternal protein degradation is an important process during early embryogenesis [49], and the degradation of maternal proteins is certainly necessary after embryonic genome activation (EGA) [49]. In this study, to obtain more candidate proteins for further analysis, expression correlation analysis was used to explore the relationship between 15 known maternal proteins identified in both biparental and uniparental

embryos (Ooep, Nlrp5, Tle6, Zbed3, Padi6, Atg5, Npm2, H1foo, Zar1, Oas1d, Pou5f1, Ago2, Dnmt1, Cdh1 and Ctcf, including SCMCs) and other quantified proteins (Supplementary Table S6). The intersection of correlation analysis result in two reported fertilized embryo proteome databases used as the ZY group (1,958 proteins, Supplementary Fig. S4A). In addition, detailed intersection relationships of the 15 maternal proteins and their candidate proteins ($|r| \geq 0.70$ and $p \leq 0.05$) are shown in Supplementary Fig. S4B (PA group), Supplementary Fig. S4C (ZY, identified by Gao) and Supplementary Fig. S4D (ZY, identified by Israel).

The Venn analysis among the MII, PA and ZY groups sorted these candidate proteins into different sets (Fig. 5A, Supplementary Table S8): 765 proteins were detected only in MII oocytes, indicating that these maternal proteins may be involved in the processes of oogenesis and oocyte maturation; 397 proteins were detected only in PA embryos, indicating that these proteins may be necessary for the development of uniparental embryos and may be regulated by the maternal genome; 599 proteins were detected only in ZY embryos, indicating that these proteins may be necessary for the development of biparental embryos and regulated by the zygote genome; 529 proteins were detected in three groups (MII, PA, ZY), indicating that these maternal proteins may not only be involved in the process of oogenesis and oocyte maturation, but also play important roles in embryonic development (including biparental and uniparental embryos); 231 proteins were detected in PA and ZY embryos, indicating they may play roles in early embryonic development in biparental and uniparental embryos

(after fertilization or artificial activation); 600 proteins were detected in both MII oocytes and ZY embryos, which reveals that, in the process of sexual reproduction, these proteins may not only play a role in oogenesis and oocyte maturation, but also remain present during embryonic development.

Especially, 316 proteins were detected in MII oocytes and PA embryos, but not in ZY embryos, indicating that these maternal proteins may be degraded after fertilization in ZY embryos. However, in uniparental embryos, these maternal proteins seem to escape degradation, which indicates that they may play a key role in parthenogenesis. In the normal bisexual reproduction of mammals, their function may only be in the regulation of oocyte development or maturation. The annotation results of these proteins show that they were mainly located in embryos and were mainly involved in biological processes of translation, gene expression and transport. The KEGG results suggest that these proteins are mainly involved in pathways related to metabolism, ribosomes and oxidative phosphorylation (Fig. 5B). Furthermore, we performed qPCR verification on 12 maternal ribosomal factors involved in the process of ‘gene expression’ (Fig. 5C) using uniparental embryos. The results show that their mRNA levels coincided with the protein level (peak at the morula or blastocyst stage) (Fig. 5D), suggesting that these maternal proteins remain present though the early development of mouse uniparental embryos.

In biparental embryos, protein degradation occurs after fertilization [49], and this

early degradation is dependent on the maternally derived ubiquitin-proteasome system (UPS) and autophagy [50-52]. After the inhibition of proteasomal activity, polyubiquinated proteins become accumulated after fertilization [53]. In this study, we further detected the proteasomal activity level of biparental and uniparental embryos in six early developmental stages. The results reveal that the proteasome activity of biparental embryos before the 8-cell stage was stronger than that of uniparental embryos (Fig. 5E). To some extent, this may provide the conditions for the 'degradation escape' of these maternal proteins in uniparental embryos. We speculate that these proteins in PA embryos may escape the fate of degradation, so we have called this phenomenon 'degradation escape' in uniparental embryos (Fig. 5F).

The expression and functional analysis of a new maternal factor

The components of the SCMC include Ooep (Floped), Nlrp5 (Mater), Tle6, Filia, Zbed3, Nlrp2, and possibly Padi6 and Nlrp7 [8, 31, 54]. Among them, Ooep, Nlrp5 and Tle6 interact with each other directly and are necessary to maintain the stability of the complex [1, 6, 31, 55].

In this study, among the candidate proteins that strongly correlated with the core SCMC components (Ooep, Nlrp5 and Tle6), a family of F-box/WD40 repeat-containing proteins (Fbxws) attracted our attention. We found that only Fbxw11 and Fbxw15 were detected in uniparental embryos (PA group, identified in

this study), while Fbxw8, Fbxw11, Fbxw13, Fbxw15, Fbxw16, Fbxw18, Fbxw19, Fbxw20, Fbxw21, Fbxw22, Fbxw24, Fbxw26 and Fbxw28 were detected in biparental embryos (ZY group, identified by Gao and Israel) (Table 1). Obviously, the quantity of Fbxws in biparental embryos is much more than in uniparental embryos. Furthermore, in uniparental embryos, Fbxw11 was not correlated with SCMC components, but in biparental embryos it had a strong negative correlation ($r \leq -0.70$, marked in green) with SCMC components. Also, the expression correlation between Fbxw15 and the SCMC components was the opposite in the two embryos: Fbxw15 had a strong negative correlation ($r \leq -0.70$, marked in green) with SCMC components in uniparental embryos, but a strong positive correlation ($r \geq 0.70$, marked in red) with SCMC components in biparental embryos (Table 1). Finally, Fbxw24 was selected for further analysis, because its correlation relationship with the other Fbxws was similar to that of SCMC components in biparental embryos (Table 1). This suggests that it may be of importance in normal embryonic development from the zygote, and its role in preimplantation embryo development has not been studied at all yet.

Firstly, we utilized an immunofluorescence assay to assess the location of Fbxw24 in early embryos. The results demonstrated a predominant cytoplasmic location of Fbxw24 in early embryos (Fig. 6A), and the fluorescence intensity decreased after the 8-cell stage (Fig. 6B). After fertilization, the mRNA level of Fbxw24 was high at the pronuclear stage and decreased significantly starting with 2-cell embryos (Fig. 6C). With the progress of cleavage, its mRNA level continued to decrease, and was almost

undetectable at the blastocyst stage, and its protein level sharply decreased from the 8-cell to the blastocyst stage (Fig. 6C), which coincides with the results of fluorescence intensity detection. Although the mouse zygotic genome is activated after the 2-cell stage, the transcriptional activity of Fbxw24 remained low. These results reveal that molecules of Fbxw24 accumulate mainly during oocyte maturation, and *Fbxw24* probably plays a role as a maternal effect gene in early embryonic development.

Secondly, the mRNA level of Fbxw24 was detected using different mouse tissues. We found that its transcripts in ovary, but not in 10 other tissues (including testis) (Fig. 6D). Immunohistochemistry and immunoblotting were also used to assess the expression and location analysis of Fbxw24 at the protein level. The immunoblotting results for 11 tissues reveal that Fbxw24 (~48 kDa) was detected in the mouse ovary, but not in 10 other tissues (Fig. 6E). The immunohistochemistry results indicate that the Fbxw24 was located in oocytes (red arrows) and granulosa cells (red arrowheads) at different follicular stages (Fig. 6F).

Next, small-interfering RNAs (siRNAs) were injected into MII oocytes followed by intracytoplasmic sperm injection (ICSI) to knock down Fbxw24 during early embryogenesis (Fig. 7A). Compared with the control, the developmental competence of Fbxw24-knockdown (KD) embryos decreased from the 2-cell stage, and the development of embryos was arrested at the 8-cell stage, then failed in the morula and

blastocyst stages (Fig. 7B-C). Fbxw24 knockdown embryos were mainly arrested between the 2- and 8-cell stages, showing results similar to those of known maternal-effect genes, such as Nlrp5 [7], Nlrp2 [56] and Padi6 [8].

To study the underlying mechanism, we performed protein-protein interaction enrichment of Fbxw24 by immunoprecipitation followed by MS (IP-MS) of FLAG-Fbxw24 (Fig. 8A and Supplementary Table S10). Notably, several components of the ubiquitin-mediated proteolysis pathway, including DNA damage-binding protein 1 (Ddb1), Cullin-4B (Cul4b), Ubiquitin conjugation factor E4 B (Ube4b), Ubiquitin conjugation factor E4 A (Ube4a), Ubiquitin-40S ribosomal protein S27a (Rps27a) and Ubiquitin-conjugating enzyme E2S(Ube2s), were detected as the main proteins in Fbxw24 pull-down complexes (Fig. 8B). The interaction between Fbxw24 and Ddb1-Cul4b was validated in HEK-293T cells by co-immunoprecipitation experiments, and their interaction was further explored in vivo using C57BL/6J-*Fbxw24*$^{\text{em(Linker-3xFlag)}}$ homozygote mice ovaries (HO) (wild type littermates (WT) as the control) (Fig. 8C). These results suggest that there is a novel interaction between Fbxw24 and Ddb1-Cul4b. Cullin4 (CUL4) utilises damaged DNA binding protein-1 (DDB1) as a linker to interact with a subset of DDB1-Cullin-associated factors (DCAFs) [57, 58], and DDB1 is highly expressed in mouse oocytes [59]. Additionally, the expression pattern of 16 members identified in the Fbxw24 pull-down complexes were analysed using MS analysis and verified using PRM in fertilized embryos, which revealed different expression patterns of these members

during the early embryo stages (Fig. 8D). Among them, SUMO-activating enzyme subunit 1 (Sae1), Anaphase-promoting complex subunit 5 (Anapc5), Cell division cycle protein 16 homolog (Cdc16), Protein PML (Pml), E3 ubiquitin-protein ligase TRIP12 (Trip12) and SUMO-activating enzyme subunit 2 (Uba2) showed an upward trend; in contrast, Ubiquitin-40S ribosomal protein S27a (Rps27a), Cullin-4B (Cul4b), STIP1 homology and U box-containing protein 1 (Stub1), and Ubiquitin-conjugating enzyme E2 S (Ube2s) showed a downward trend. These results reveal that Fbxw24 may be involved in the regulation of maternal protein degradation during early embryonic development.

Discussion

The function of maternal proteins was reported initially in invertebrates. Researchers induced mutants in *Drosophila* and found that the polarity of *Drosophila* eggs and embryos is regulated by the maternal factors. Therefore, the importance of maternal molecules for embryonic development has been demonstrated [60-66]. Subsequently, in vertebrates, the function of maternal factors was also reported, including the fusion of male and female pronucleus, zygotic genome activation (ZGA) and the degradation of maternal components [1, 67]. In mammals, the first maternal factor Mater was reported in mouse [7]. Mater (maternal antigen that embryos require; also known as Nlrp5) may be related to the activation of the zygotic genome [7]. The known maternal effect factors in the mouse have been reviewed by Li and Zheng [1, 67].

Although the importance of maternal factors for embryonic development has been known for a long time, research progress in mammals has been slow due to the limitations of research materials and technology.

Mouse parthenogenetic embryos are only regulated by the maternal genome, with similar morphology compared to fertilized embryos, so some proteins expressed in parthenogenetic and fertilized embryos may have similar expression trends, and their presence and normal expression changes provide a molecular basis for the early development of the two embryos. Uniparental embryos derived from only the oocyte may be a unique model for studying genomic imprinting and the maternal contribution to embryonic development. In addition, the inner cell mass of the blastocyst in parthenogenetic embryos can also be used as a source of parthenogenetic embryonic stem cells (pESCs); this has been successfully established in many species, including mouse, monkey and human [68-71]. Recently, a study reported that fertile mice can be bred from single oocytes by targeted DNA methylation by rewriting seven imprinting control regions without sperm participation [72]. Although parthenogenetically activated oocytes cannot develop to term in mammals due to the disruption of imprinted gene expression and DNA methylation status, the protein landscape of parthenogenetic embryos has not been studied. .

In this study, to obtain more information about maternal proteins, our first consideration was to construct the protein database of mouse parthenogenetic

embryos before implantation and perform a comparison of mouse biparental and uniparental embryos at the protein level. By the label-free quantitative mass spectrometry (MS) method, we detected a total of 2,048 proteins in six preimplantation stages of mouse parthenogenesis. By employing the comparison of maternal proteins in mouse biparental and uniparental embryos, we found two similar protein expression patterns in the uniparental and biparental embryos, revealing that these two patterns may be regulated mainly by the maternal genome. Our second consideration was to use the SCMC as target proteins and explored the expression correlation between the SCMC and other identified proteins in both biparental and uniparental embryos. As expected, a number of key candidate maternal proteins were obtained, and among them, some maternal proteins that were strongly negatively correlated with SCMC may play an important role in the process of blastocoel formation.

In addition, by analysing the relationship between 15 known maternal proteins and other proteins identified in both biparental and uniparental embryos, we found that some maternal proteins are degraded with the activation of oocytes in biparental embryos; however, in uniparental embryos, they remain present during preimplatation development, and their mRNA and protein levels showed an upward trend. Also, the proteasome activity of biparental embryos before the 8-cell stage was stronger than that of uniparental embryos, which revealed that these maternal proteins in mouse uniparental embryos may escape degradation. Based on this, we inferred that some

maternal protein degradation after oocyte activation may require sperm participation. Fertilization may trigger the degradation of these maternal ribosomal proteins, or the small amount of cytoplasm carried by the sperm contains some factors that may regulate their degradation. The development of uniparental embryos occurs without sperm or any paternal factor, so it leads to a phenomenon of maternal protein 'degradation escape'. The fate of incomplete development in uniparental embryos provides clues on early abnormal maternal protein expression, such as 'degradation escape'.

Moreover, among the candidate proteins that strongly correlated with the three core SCMC components (Ooep, Nlrp5 and Tle6), a large set of F-box/WD40 repeat-containing proteins (Fbxws) were present in mouse biparental embryos, which may suggest the involvement of the Skp1-Cullin-F-box (SCF) complex in maternal protein degradation in mice. Wang et al. [16] also identified a large group of F-box proteins in mouse oocytes and zygotes. SCF protein-ubiquitin ligase complex member F-box proteins are highly abundant in oocytes and two-cell embryos [73]. For example, Fbxw15/Fbxo12J is an F-box protein-encoding gene that is selectively expressed in oocytes of the mouse ovary [74]. These F-box proteins may play important roles in protein degradation after fertilization, and different F-box complexes can selectively degrade specific target proteins. Finally, a poorly studied F-box/WD40 repeat-containing protein (Fbxw24) was selected for further analysis. We found that the expression pattern of Fbxw24 was very similar to a maternal effect

factors reported before [8, 75, 76] and, following the knock down of Fbxw24 by siRNA interference, the affected embryos showed a phenotype of developmental arrest. By the construction of the Fbxw24-specific interaction proteome using Stable isotope labeling of amino acids in cell culture and immunoprecipitation-MS (SILAC-IP-MS) and validation in vivo, we confirmed a new specific interaction between Fbxw24 and Cul4b-Ddb1 (key components of the ubiquitin proteasome pathway). We speculate that Fbxw24 may also be involved in the degradation of maternal proteins in early embryos. These results suggest that *Fbxw24* is a new putative maternal effect gene, and the comparative analysis of biparental and uniparental embryos may be helpful to find out the key maternal factors regulating early embryonic development.

Then, the potential limitation of this study is that the quantity of proteins identified in parthenogenetic embryos (2,048 proteins) is less than that in fertilised embryo reported before: Gao et al. identified nearly 5,000 proteins across six developmental stages by the tandem mass tag (TMT) labelled method, and Israel et al. identified 6,550 proteins by stable isotope labelling with amino acids in cell culture (SILAC). However, the mouse parthenogenetic embryo only contains maternal genomic information, which may cause some genuine biological absence. Additionally, the difference in protein quantity may have occurred for the following reasons: 1. different proteome construction strategies. The known proteome of fertilised embryos was constructed using a quantitative proteomic strategy based on TMT labelling and

the SILAC method [11, 17], while the proteome of parthenogenetic embryos was constructed by label-free quantitative mass spectrometry. 2. Different protein databases used for the search. The raw mass spectrometry (MS) data of fertilised embryos identified by Gao were searched with the ProLucid algorithm against the International Protein Index (IPI) mouse protein database [17], and the raw MS data of fertilised embryos identified by Israel were searched using MaxQuant software against the Uniprot KB database [11]. The raw MS data of parthenogenetic embryos were search using MaxQuant software against the Swiss-Prot mouse database (UP000000589). It must be noted that the Swiss-Prot database is manually annotated and reviewed, and the number of proteins is less than those of the IPI and Uniprot KB databases. Also, some known maternal proteins, including Brg1, Dppa3, Ezh2, Filia and Zfp57, were detected in this study (uniparental embryos) and in the proteome of biparental embryos identified by Israel, but not identified by Gao. These undetected members may hint at a different technical limitations, but since these proteins were also detected in parthenogenetic embryos by label-free quantitative mass spectrometry, to some extent, it can be concluded that the proteome database of uniparental embryos constructed in this study is reliable. To date, the proteome of parthenogenetic embryos is reported for the first time in this study, and there is no other protein database derived from maternal uniparental embryos that can be used for the comparison of coverage. It remains to be confirmed that the existence of false negatives in the parthenogenetic embryo proteome are caused by different artificial activation methods.

Taken together, we systematically constructed a maternal protein file of mouse uniparental embryos and showed the dynamic patterns of maternal proteins during preimplantation development using a combined analysis of uniparental and biparental embryos. These data provide valuable resources for further mechanistic studies of maternal proteins and aid in mining potential key players and their regulatory mechanisms governing early embryo development.

Methods

Experimental animals

C57/BL6J mice were obtained from the animal breeding colony in the Animal experimental center of Guangxi Medical University, China. In order to design C57BL/6J-*Fbxw24*$^{em(Linker\text{-}3xFlag)}$ mice, CRISPR/Cas9-mediated homologous recombination (Shanghai Model Organisms Center, Inc) was used to insert the 3x flag sequence and frame it with the last exon of Fbxw24 gene before the stop codon. A 12h light-12h dark cycle was created for the mice that were kept in rooms with controlled temperatures. Food and water were sufficient, and mice were free to eat.

Collection of mouse embryos

Six- to eight-week-old C57BL/6J female mice were super-ovulated following the injection of 10IU of pregnant mare serum gonadotropin (PMSG; Ningbo Second

Hormone Factory, China) and 10IU of human chorionic gonadotropin (hCG; Ningbo Second Hormone Factory, China). The injection of two hormones was 48 h apart.

Collection of parthenogenetic embryos was performed, following a previously reported protocol [19]. In brief, the oocytes surrounding the cumulus cells were recovered from female C57BL/6J mice oviducts after 16–18 h post-hCG administration. When the cumulus cells were still attached to the oocytes, they were discharged into 7 percent ethanol freshly prepared in M2 media and permitted to rest for 5 minutes at room temperature. Then the oocytes were cleaned three times with M2 media in culture dishes and cumulus cells were then eliminated using a hyaluronidase treatment (1 mg/ml, dissolved in M2 media). Then transferred the oocytes into drops of M16 medium (containing cytochalasin B, 5ug/ml)) and incubate them for 4-5 hours at 37℃ under 5% CO_2 (suppressing the emission of the second polar body and predominantly developing diploid parthenogenetic embryos). The formation of pronucleus was observed under the microscope, and the activated oocytes with double pronucleus were transferred to KSOM medium for further culture. The 2-cell stage embryos were collected after 22-24 hours and 4-cell to morula stages embryos were collected after 2–3 days, and the blastocyst stage collected on day 4.

To obtain fertilized embryos, super-ovulated female mice were mated with male mice. The zygotes (pronucleus stage) were taken from the female C57BL/6J oviducts after 24 h post-hCG injection. The zygotes were then transferred into a KSOM medium and

incubated at 37℃ under 5% $CO2$. The two-cell stage fertilized embryos were collected after 22-24 h. The fertilized embryos at the 4- to morula stages and blastocyst stage were collected 2-3 days after incubation and on day 4 after incubation, respectively.

Quantitative mass

The label-free quantitative mass experiment was performed on parthenogenetic embryos (Pronucleus-, 2-cell-, 4-cell-, 8-cell-, morula-, and blastocyst-stage). 6000 embryos for each stage and conducted in three biological duplicates. The lysis buffer (4% SDS，100mM Tris-HCl，1mM DTT，pH7.6) was utilized to lyse the samples and extract the proteins. The BCA protein assay kit was utilized to quantify the proteins (Bio-Rad, USA). Trypsin digestion of proteins was conducted in accordance with the standards for filter-aided sample preparation (FASP) as described [77]. Briefly, around 100 μg of total proteins were collected from each specimen and then added into 30 μl SDT buffer (100 mM DTT, 150 mM Tris-HCl, 4% SDS, pH 8.0). Repeated ultrafiltration (Microcon units, 10 kD) was used to filter out the DDT, detergent, as well as other low-molecular-weight substances utilizing UA buffer (8 M Urea, 150 mM Tris-HCl, pH 8.0). In order to inhibit decreased cysteine residues, 100 μl Iodoacetamide (100 mM IAA in UA buffer) was introduced into the specimens, and they were subsequently subjected to incubation for 30 minutes in complete darkness. Subsequently, the filters were rinsed thrice using 100 µl of UA buffer and then rinsed two times using 100 µl of 25mM NH_4HCO_3 buffer. Lastly, 4 µg trypsin (Promega) was utilized for digesting the protein suspensions at a temperature of 37°C over night

in 40 µl 25mM NH_4HCO_3 buffer, and the obtained peptides were extracted as the filtrate. Peptides from each sample were desalted on a C18 cartridge (Empore™ SPE Cartridge C18 (standard density), bed I.D. 7 mm, volume 3 ml, Sigma), followed by concentration using vacuum centrifugation and reconstitution in 40 µl of 0.1% (v/v) formic acid. Then 120 minutes were spent performing the liquid chromatography coupled to MS (LC-MS/MS) analysis utilizing the Q Exactive mass spectrometer (Thermo Scientific) that was coupled to an Easy nLC (Proxeon Biosystems, nowThermo Fisher Scientific). A reverse-phase trap column (100 μm×2 cm, nanoViper C18, Thermo Scientific Acclaim PepMap100) connected to a C18-reverse-phase analytical column (10 cm long, 75 μm inner diameter, 3μm resin, Thermo Scientific Easy Column) in buffer A (0.1 percent Formic acid) was used to load the peptides, which were then isolated utilizing buffer B (0.1% Formic acid and 84% acetonitrile) with a linear gradient, at a flow rate of 300nL/min regulated by IntelliFlow technology. The positive ion mode of the mass spectrometer was set for this experiment. By utilizing the data-dependent technique, the MS data were obtained via the dynamic selection of precursor ions that are most abundant as identified by the survey scan (300–1800 m/z) for fragmenting HCD (Higher energy collisional dissociation). 3e6 was chosen as the automatic gain control (AGC) target, and the value of 10 milliseconds was adjusted as a maximum injection duration. The duration of the dynamic exclusion was adjusted as 40 seconds. The resolution of scan was adjusted to 70,000 at 200 m/z, whereas HCD spectra were observed using a resolution of 17,500 at 200m/z, with an isolation width of 2m/z. 30 eV was adjusted

as the normalized collision energy (NCE), while the underfill ratio that sets the minimum percentage of the target value that can be achieved within the maximum fill time was specified as 0.1 percent.

Identification and bioinformatic analysis

The MaxQuant software (Max Planck Institute of Biochemistry, Martinsried, Germany, version:1.5.3.17) was used to analyze the raw MS data. The detection of proteins in the MS/MS spectra was accomplished by comparing the spectra to the Swiss-Prot mouse database (UP000000589), which includes 55,366 protein sequences. The following parameters were used: the enzyme chosen was trypsin; the maximum number of missed cleavages were two; for fixed modifications, carbamidomethyl was chosen while oxidation for variable modifications; for the main search, 6 ppm was chosen, and for the first search and MS/MS tolerance, 20 ppm was chosen. The database analysis included the following patterns: the included contaminants term was considered true; protein and peptide false discovery rates were under 1%; Razor and unique peptides were used in the protein quantitation; a 2-minute interval existed between each of the runs (the match between runs); The protein quantification technique used in this study was LFQ; one was set as the minimum number of ratios required. The proteomics data from mass spectrometry have been submitted to the ProteomeXchange Consortium (http://proteomecentral.proteomexchange.org) via the iProX partner repository [78], with the dataset identifier PXD029532. Blast2GO program was used to map and annotate the sequences containing GO terms [79]. R

scripts were used to visualize the results of the GO annotation. Subsequently, the investigated proteins were compared with the KEGG database (http://geneontology.org/) to get their KEGG orthology identifications. The protein-protein interaction (PPI) of the investigated proteins was determined using STRING software (http://string-db.org/) [80]. The interaction files were visualized by Cytoscape software (http://www.cytoscape.org/, version 3.2.1) [81].

Quantitative analysis of selected proteins using parallel reaction monitoring

Some selected proteins were verified by parallel reaction monitoring (PRM) in protein level. A total of 2500 fertilized embryos of each sample from the six stages (Pronucleus-, 2-cell-, 4-cell-, 8-cell-, morula-, blastocyst-) were acquired for protein extraction. Mass shotgun analysis was performed to obtain pre-experimental results, which were used to select suitable peptides for PRM analysis. Briefly, in order to desalt tryptic peptides, they were put onto C18 analytical column (Thermo Scientific) prior to reversed-phase chromatography on the EASY-nLC™ 1200 system. Gradients of acetonitrile ranging from 5% to 35% in 45 minutes were utilized in one-hour liquid chromatography. A Q Exactive ™ Plus Hybrid Quadrupole-Orbitrap ™ Mass Spectrometer was used to perform the PRM analysis. Using the Skyline 3.5.0 software (MacCoss Lab, University of Washington), the raw data was examined [82]. The PRM validation results of selected proteins are shown in Supplementary Table S11. The proteomics data from mass spectrometry have been submitted to the ProteomeXchange Consortium via the iProX partner repository [78], with the dataset

identifier PXD029532.

The knockdown of fbxw24 in early embryos

The small-interfering RNAs (siRNAs) of mouse *Fbxw24* and the negative control were diluted to 5 mM final concentration using nuclease-free water. Using a Piezo-driven micromanipulator, about 10 pL of 5-mM siRNAs were delivered into the oocytes. Then, the implanted oocytes were cultured for at least 3 hours in order to prepare for intracytoplasmic sperm injection (ICSI). About 1 mL of the sperm suspension was combined with the HEPES-buffered Chatot-Ziomek-Bavister (HCZB) medium drop comprising of 10% (w/v) polyvinylpyrrolidone (Irvine Scientific, Santa Ana, CA, USA). Using several Piezo pulses, the sperm head and tail were separated and the head was then injected into the oocyte in accordance with the procedure provided by Ward and Yanagimachi [83]. HCZB medium was used for gamete handling and ICSI, whereas Chatot-Ziomek-Bavister (CZB) medium was used for embryo culturing in an atmosphere of 5% CO2. For embryo culture, CZB was overlaid with sterile mineral oil (Sigma). To analyze the knockdown (KD) efficiency of siRNA, total RNA of 15 embryos at 4-cell stage was purified by the RNeasy Mini Kit (QIAGEN, catalog number 74104). A reverse transcription technique was used to synthesize the cDNA (Promega). The StepOneTM Real-Time PCR System (Applied Biosystems) was used to perform the quantitative real-time PCR, and H2afz as the internal control. Primer sequences and siRNA-targeting sequences are shown in the Supplementary Table S9.

Measuring mRNA levels using Real-time PCR

The isolation of the total RNA from embryos was carried out at six stages (Pronucleus-, 2-cell, 4-cell, 8-cell, Morula-, Blastocyst-) utilizing the RNeasy Mini Kit (QIAGEN, catalog number 74104). A reverse transcription technique (Promega) was used to synthesize the complementary DNA. The Real-Time PCR System (Applied Biosystems) was used to perform the quantitative real-time PCR analysis. The relative expression levels were calculated using the $2^{-\Delta\Delta CT}$ method. All experiments were carried out in three biological repetitions. The primer sequences are shown in Supplementary Table S9.

Immunoblotting, immunofluorescence and immunohistochemistry analysis

For immunoblotting analysis, the different tissues was obtained from C57BL/6J-*Fbxw24*$^{em(Linker\text{-}3xFlag)}$ mice and the extracted proteins were isolated using sodium dodecyl sulfate-polyacrylamide gel electrophoresis (SDS-PAGE). Using a semidry western blotting technology (Trans-Blot® TurboTM System, Bio-Rad, Singapore), the proteins were then deposited onto a polyvinylidene difluoride membrane. Then the membrane was blocked with 5 percent nonfat milk for 1 hour at 37°C before using the primary antibodies to incubate it at 4°C overnight. Cleaning of the membrane was performed three times using a solution composed of Tris-buffered saline (TBS) and Tween 20 (TBST buffer), followed by the incubation for one hour at 37°C with the secondary antibodies in TBST. An alkaline phosphatase detection kit

(C3206, Beyotime Biotechnology Inc, Shanghai, China) was utilized to identify the presence of proteins.

For immunofluorescence analysis, C57BL/6J-*Fbxw24*$^{em(Linker\text{-}3xFlag)}$ female mice embryos were kept at room temperature for 30 minutes in 4% polyoxymethylene. In addition, the embryos were placed into a 1% Triton X-100 phosphate-buffered saline (PBS) solution for 20 minutes, then a solution of PBS comprising of 1% bovine serum albumin (BSA) was utilized to block them. In the next step, incubation of the embryos was performed overnight at 4℃, followed by a combination with a primary antibody at an effective concentration. Then Embryos were washed three times with a PBS solution containing 0.1% Tween-20 and 0.01% Triton X-100 for 2 minutes each. It took one hour to incubate the embryos at room temperature in a diluted solution consisting of a secondary antibody (fluorescein isothiocyanate labeled) and then washed thrice for 2 minutes each time with a PBS solution containing 0.1% Tween-20 and 0.01% Triton. The specimen was covered using a coverslip coated in a ProLong Gold antifade reagent containing DAPI (Life Technologies) and stored in the darkness until fluorescence was determined using an inverted fluorescence microscope (Olympus, IX73, Japan). Fluorescence intensity for immunofluorescence was measured using ImageJ (National Institutes of Health).

For immunohistochemistry analysis, C57BL/6J-*Fbxw24*$^{em(Linker\text{-}3xFlag)}$ female mice ovaries were fixed in 4% paraformaldehyde, and paraffin was used to embed them.

The ovarian sections (5μm) were deparaffinized using xylenes and rehydrated in serials ethanol dilutions. The sections were rinsed in 1% PBS-Tween-20 and then it was treated using 2% hydrogen peroxide. Incubation with specified primary antibodies for 2 hours at room temperature after blocking using 3 percent goat serum was performed. The sections were treated using the secondary antibody for 40 minutes at 37℃. Negative controls were processed with PBS instead of the primary antibody. Sections were analyzed with the aid of a light microscope (Olympus, IX73, Japan). The following primary antibodies were utilized: anti-beta anti-GAPDH rabbit monoclonal antibody (ab181602, Abcam) and anti-DDDDK tag (Binds to FLAG® tag sequence) rabbit monoclonal antibody (ab205606, Abcam).

Proteasome activity assay

Proteasome activity assays were performed as previously reported with minor modifications [84]. To assess oocyte proteasome activity in six stages embryonic development of mouse biparental and uniparental embryos, 100 embryos per group were collected and washed three times in PBS/PVP. All cell suspensions were lysed in 20 μl of protein extraction buffer composed of 150 mM sodium chloride, 50 mM Tris, and 0.5% Triton X-100 for 30 min under constant rotation at 4℃. After centrifugation at 16,300 g for 15 min, supernatants were transferred to a clean tube and then assessed for proteasome activity using a commercial proteasome assay kit, which utilizes an AMC tagged peptide substrate which releases free, highly fluorescent AMC in the presence of proteolytic activity (Abcam, ab107921). In brief, 10 μl of each sample

was loaded into a 96-well plate in duplicate, alongside a Jurkat cell lysate-positive control (supplied) and AMC protein standards. A total of 50 μM proteasome inhibitor MG132 was added to one well of each sample to differentiate proteasome activity from other protease activity that may be present in the samples. Plates were incubated for 25 min and then analyzed on a TECAN Sunrise™ plate reader (Austria) at an excitation/emission of 350/440 nm. Following a further 35 min incubation at 37℃, plates were analyzed a second time to allow the change in relative fluorescence units to be calculated for each sample. Data were analyzed following the manufacturers' instructions, and proteasome activity was calculated such that one unit of proteasome activity is equivalent to the amount of proteasome activity that generates 1.0 nmol of AMC per min at 37℃. This experiment was repeated across three independent biological and technical replicates using 100 embryos per assay.

Stable isotope labeling of amino acids in cell culture and immunoprecipitation-MS (SILAC-IP-MS)

Following the SILAC (stable isotope labeling of amino acids in the cell culture) labeling procedure [85, 86], HEK-293T was maintained in DMEM medium containing 13C6-Lysine (K6) and 13C6-Arginine (R6) (Cambridge Isotope Laboratories, Inc.), which was supplemented with 10 percent dialyzed fetal bovine serum (Invitrogen) for seven passages for the complete labeling of the cellular proteome. The mouse Fbxw24 gene (NM_001013776) was cloned into a p3× FLAG-CMV-7.1 vector (Sigma-Aldrich) between the NotI and SalI sites and

transfected into HEK-293T cells maintained in normal DMEM medium (Light, L) using lipofectamine 2000. The empty vector of p3×FLAG-CMV-7.1 was transfected into K6R6-labeled HEK-293T cells as the control group (Heavy, H). 20μM MG132 (SelleckChem) was added to the transfection solution for an additional 6 hours before collection. The collected cells from the L and H groups were lysed using 150mM NaCl, 20mM Tris (pH 7.5) and 2 percent Triton X-100 supplemented with phosphatase and protease inhibitor cocktail. Anti-FLAG beads (Sigma-Aldrich) were added to the groups for immunoprecipitation (IP) and the enriched proteins were eluted by 3×FLAG peptide (Sigma-Aldrich). The final eluates obtained after Flag peptide elution were analyzed by SDS-PAGE. Coomassie Brilliant Blue dye was used for staining and the visible bands were excised for the next LC-MS/MS analysis. The peptide extraction, as well as in-gel protein trypsin digestion, were carried out according to the previously described protocol [87]. Analysis of peptide samples was performed on an EASY-nLC 1000 system (Thermo Fisher Scientific, Waltham, MA) that was connected to an Orbitrap Fusion mass spectrometer (Thermo Fisher Scientific, San Jose, CA). 10μl of solvent A (water comprising 0.1% formic acid) was utilized to resuspend the peptide, followed by the loading of 8μl peptide sample onto a trap column (100μm×2cm, Thermo Scientific Acclaim PepMap C18) for 3 minutes at a flow rate of 10μl/min, followed by the separation on an analytical column (Acclaim PepMap C18, 75 μm x 25 cm) with a linear gradient. This gradient ranged from 5% B (water containing 0.1% formic acid, 90% acetonitrile) to 35% B in 75min, 35% to 60% B in 5min, 60% to 90% B in 5min, and 90% to 2% B in 5min. The flow

rate inside the column was sustained at 300nL/min. Using the data-dependent method, the Orbitrap Fusion mass spectrometer switched automatically between MS and MS/MS acquisition. The Orbitrap was utilized to acquire survey full-scan MS spectra (m/z 350-1600) with 120,000 adjusted as the mass resolution, 1000,000 adjusted as the AGC, and 50ms adjusted as the utmost duration for injection. The acquisition of MS/MS was performed in an orbitrap at maximum speed mode and a cycle period of 3 seconds, with the mass resolution adjusted to 15,000, the AGC target adjusted to 100,000, the utmost injection duration adjusted to 80 milliseconds, and the isolation width adjusted to 1.6 m/z. HCD was used to break down ions with charge states 2+, 3+, and 4+ consecutively, with a normalized collision energy (NCE) of 30%. Microscans were recorded utilizing dynamic exclusion for 30s in all cases. Maxquant software (version 1.5.2.8, available at http://www.maxquant.org/) was utilized to process the MS raw data in order to identify and quantify the proteins [88]. We employed the Andromeda search engine [89] to search for relevant data in the mouse UniProtKB/Swiss-Prot databases. The following criteria were established: (1) The required length of the peptide was at least 7 amino acids. (2) The trypsin cleavage specificity was used with a maximum of 2 missing cleavages permitted. (3) Oxidation (M) and acetylation (protein N-term) were the two types of variable modifications considered. (4) For both the precursor and fragment ions, the initial mass variation was as high as 10 ppm and 5 Da, respectively. (5) At both the protein and peptide levels, the false discovery rate (FDR) was set as 1%. (6) Multiplicity of 2 was used, where 13C6-Lysine (Lys6, K6) and 13C6-Arginine (Arg6, R6) were selected as heavy

(H) labels. (7) "Re-quantify" option was selected. (8) Utilizing unmodified unique and razor peptides, as well as a minimum of 2 counted ratios, the Quantification was carried out. (9) Any protein that sequenced a minimum of two peptides was deemed to be a reliable indentation. Statistical analysis of the determination of specific interaction was evaluated utilizing Significance B ($p<0.05$), by Perseus (version 1.6.7.0, http://www.perseusframework.org/) on the log2 L/H ratio. The significance B value indicates whether a ratio is different from the distribution of all protein ratios grouped according to intensity [88]. The proteomics data from mass spectrometry have been submitted to the ProteomeXchange Consortium via the iProX partner repository [78], with the dataset identifier PXD029532.

IP and western blotting

5 µg of anti-DDDDK tag rabbit monoclonal antibody was utilized to coat magnetic Beads Protein G (binds to the FLAG tag sequence) in IP wash buffer (50 nM Tris-HCl, 150 nM sodium chloride, 0.05% NP-40 and 1 mM $MgCl_2$, pH 7.4) with 5percent BSA at 4°C and incubated overnight with rotation. The HEK-293T cells (in normal DMEM medium) and C57BL/6J-*Fbxw24*$^{\text{em(Linker-3xFlag)}}$ HO mice ovaries were ground by liquid nitrogen and then added to IP lysis buffer (50 mM Tris-HCl, 150 mM NaCl, 1 mM EDTA, 0.1% SDS, 0.5% sodium deoxycholate, 1% NP-40, 1 mM PMSF/cocktail and 0.5 mM DTT, pH: 7.4), followed by the incubation for 10 minutes on ice. The HEK-293T cells (in K6R6-labeled DMEM medium) and ovaries of wild-type littermates were used as the control, respectively. Then, the IP lysate was centrifuged

at 14,000 rpm and 4°C for 10 minutes; 100 µl supernatant was removed and mixed with 900 µl of the beads-antibody complex in IP buffer (35 µl 0.5 M EDTA and 860 µl IP wash buffer), followed by overnight incubation with 4°C rotation. 50 mL elution buffer was added into the immunoprecipitate after rinsing, and the supernatant was utilized for western blot analysis. Four commercial antibodies were employed: anti-Ddb1 antibody (ab109027, Abcam), anti-Cul4b antibody (12916-1-AP, Proteintech Group), anti-DDDDK tag (Binds to FLAG® tag sequence) rabbit monoclonal antibody (Binds to FLAG® tag sequence) (ab205606, Abcam), and anti-beta anti-GAPDH rabbit monoclonal antibody (ab181602, Abcam).

Statistical analysis

Statistical analyses were conducted using the IBM Statistical software (SPSS 23.0). The mean ± standard error of the mean was used to express the data. One-way analysis of variance or the unpaired t-test with Tukey's post hoc test was utilized to perform the comparison of the statistical data (*$P < 0.05$).

Availability of supporting data

The mouse uniparental embryo proteome that constructed in this study (deposited via the PRIDE partner repository) with accession number ProteomeXchange: PXD029532.

Ethics approval

All experimental protocols were reviewed and approved by the Animal Care and Use

Committee of Guangxi Medical University (No.201901012).

Competing interests

The authors declare that they have no competing interests.

Funding

This study was funded by China Postdoctoral Science Foundation (2019M653810XB), the Guangxi Natural Science Foundation Program (2019JJB140131) and Guangxi First-class Discipline Project for Basic medicine Sciences (No.GXFCDP-BMS-2018).

Author Contributions

FC, XL, TX and YZ performed the experiments. BM, YL, FC, YL and YZ supervised, and analyzed experimental data. FC, QZ, BL and XX wrote the manuscript.

Acknowledgments

We thank Dr. Shisheng Wang (West China Hospital, Sichuan University) and Dr. Chengpin Shen (Omicsolution Co., Ltd) for giving some advices about data analysis and 'Wu Kong' platform (https://www.omicsolution.com/wkomics/main/) for the Fuzzy C-means clustering, PCA, hierarchical clustering analysis, upset plot and venn diagram analysis.

Figure legends

Figure 1. Temporal profiles of maternal protein expression in mouse uniparental embryos. (**A**) Statistical histogram of mass spectrometry results from mouse uniparental embryos. 2048 proteins were identified and 1902 proteins were quantified. Total spectrum: total number of secondary spectra; Matched spectrum: the number of

spectra matched to the database; Total Peptides: the number of all peptides; Unique peptides: the number of unique peptides; Detected Proteins: the number of proteins detected; Quantified Proteins: The number of proteins that can be quantified. (**B**) UpSet intersection diagram of proteins detected in six stages of mouse uniparental embryos. The blue horizontal bar shows the number of proteins identified from each group, while the line with solid orange dots below the X-axis indicates an intersection among different groups, and the black vertical bar on the X-axis shows the number of proteins for the corresponding intersection. PA, PA2, PA4, PA8, PAMO, and PABL represent different embryonic stages of parthenogenesis: Pronucleus stage (PA), 2-cell stage (PA2), 4-cell stage (PA4), 8-cell stage (PA8), Morula stage (PAMO), Blastocyst stage (PABL). (**C**) Hierarchical grouping analysis of maternal proteins in six developmental stages of mouse uniparental embryos. (**D**) Maternal protein expression patterns analysis using principal component analysis (PCA). (**E**) Fuzzy c-means clustering analysis of protein expression during uniparental embryonic development. Color gradient corresponds to the cluster membership value. All protein expression changes quantified in this study are centred and scaled around a mean of 0 and a standard deviation of 1. The colors purple and red indicate proteins with membership values that are above 0.5, whereas yellow and green denote proteins with membership values that are less than 0.5.

Figure 2. General comparative analysis of three proteomic databases constructed by parthenogenetic embryo (uniparental embryo, PA group), fertilized embryo

(biparental embryo, ZY group), and mature oocyte (MII). (**A**) Venn diagram(green) showed the intersection of MII groups, quantified by Wang and Israel, respectively; Venn diagram(blue) showed the intersection of ZY groups, quantified by Gao and Israel, respectively. A sum of 1029 proteins was found among the three different groups(PA,MII,ZY). (**B**) The expression patterns of five SCMC components (Ooep, Nlrp5, Tle6, Zbed3 and Padi6, named the SCMCs in this study) in fertilized and parthenogenetic embryos, and their verification by parallel reaction monitoring (PRM) assay in fertilized embryo. PA_*this study*, quantified in this study; ZY_*Gao et al.,2017*, quantified by Gao et al, 2017; ZY_*Israel et al.,2019*, quantified by Israel et al, 2019; PRM_*this study*, validated by PRM assay using fertilized embryos in this study. PN-pronucleus stage; 2C-2-cell stage; 4C-4-cell stage; 8C-8-cell stage; MO-morula stage; BL-blastocyst stage. Pronucleus stage was used as control. (**C-E**) Hierarchical clustering of protein expression for the 1029 proteins in PA group (**C**), ZY group quantified by Gao et al.,2017 (**D**) and ZY group quantified by Israel et al.,2019 (**E**) , respectively.

Figure 3. (**A**) Venn diagrams between MII and PA groups. 1168 proteins detected both in MII and PA groups, and k-means clustering (k=6) analysis is conducted for the 1168 proteins through the development of uniparental embryo. (**B**) Venn diagrams between MII and ZY groups. 1806 proteins detected both in MII and ZY groups, and k-means clustering (k=6) analysis is conducted for the 1806 proteins using the protein expression database of biparental embryo identified by Gao and Israel, respectively.

(**C**) Venn diagrams between ZY and PA groups. 613 proteins were only detected in PA group, and k-means clustering (k=6) analysis is conducted for the 613 proteins through the development of uniparental embryo. (**D**) Schematic diagram of key biological events during mouse embryogenesis. E0.5 represents the 0.5 days after fertilization.

Figure 4. Maternal proteins that are strongly correlated with the SCMCs in mouse biparental and uniparental embryos. (**A**) Venn diagram showed the intersection between three groups (PA_Chen, represented the proteins that are strongly correlated with the SCMCs in mouse uniparental embryos; ZY_Israel, represented the proteins that are strongly correlated with the SCMCs in the proteome of the mouse biparental embryos quantified by Israel; ZY_Gao, represented the proteins that are strongly correlated with the SCMCs in the proteome of the mouse biparental embryos quantified by Gao). 429 proteins were detected in both mouse biparental and uniparental embryos; 113 were strongly positively correlated ($r \geq 0.70$), 304 were strongly negatively correlated ($r \leq -0.70$). (**B**) The KEGG annotation for the 429 candidate maternal proteins. (**C**) The protein-protein interaction (PPI) network of the candidate maternal proteins that are involved in biological process of "Translation".The proteins highlighted in the red box were used for further validation by PRM assay. (**D**) The validation results by PRM assay for the selected 8 key candidate proteins using fertilized embryos. PA_Chen, represented the expression pattern in mouse uniparental embryos; ZY_Israel, represented the expression pattern

in the proteome of the mouse biparental embryos quantified by Israel; ZY_Gao, represented the expression pattern in the proteome of the mouse biparental embryos quantified by Gao. ZY_PRM validation, represented the validation results by PRM assay in this study. (**E**) Schematic diagram showed the expression patterns between the candidate maternal proteins and the SCMCs during mouse embryogenesis.

Figure 5. (**A**) The Venn diagram showed the intersection of the candidate proteins that strongly correlated with 15 key maternal proteins in three groups. The blue circle shows the number of candidate proteins that were analyzed from the proteome of biparental embryos (ZY group, identified by Gao and Israel), the green circle shows the number of the candidate proteins that were analyzed from the proteome of uniparental embryos (PA group, identified in this study), and the light orange circle shows the maternal proteins quantified in metaphase II (MII) stage oocytes (MII group, quantified by Wang and Israel). 316 maternal proteins that are only detected in PA and MII groups, and in biparental embryos (ZY), these proteins were detected in the MII stage oocyte but degraded after fertilized. (**B**) The annotation results for the 316 maternal proteins. The green columns showed the annotation of biological process, the blue columns showed the annotation of KEGG and the red columns showed the localization in different tissues. (**C**) The protein-protein interaction (PPI) network of the candidate maternal proteins that are involved in biological process of "gene expression". (**D**) 12 maternal ribosomal factors involved in the process of "gene expression" were validated by RT-qPCR assay using uniparental embryos. The lines

represented the expression pattern in protein level, and columns represented the expression pattern in mRNA level. Pronucleus stage was used as control. *$P < 0.05$. (**E**) The proteasome activity was detected in six developmental stages of mouse biparental and uniparental embryos using a fluorometric proteasome activity assay kit. One unit of proteasome activity is defined as the amount of proteasome that generates 1.0 nmol of the fluorescently tagged AMC per min at 37°C. (**F**). Schematic diagram of "Degradation Escape" maternal proteins. MII, metaphase II of oocyte; 7% CH2CH2OH, M2 solution contains 7% ethanol. Some maternal proteins may "escape" from degradation in mouse uniparental embryos.

Figure 6. The expression analysis of Fbxw24 in mouse. (**A**) Cellular localization of Fbxw24. The different stage embryos were cultured from the zygotes of C57BL/6J-*Fbxw24*$^{em(Linker\text{-}3xFlag)}$ mice. Each sample was performed using DAPI to allow for the visualization of DNA (blue). scale bar: 20 μm. PN-pronucleus stage; 2C-2-cell stage; 4C-4-cell stage; 8C-8-cell stage; MO-morula stage; BL-blastocyst stage. (**B**) Quantification of Fbxw24 fluorescence intensity in six biparental embryo stages. Pronucleus stage was used as control. *$P < 0.05$. (**C**) The relative transcripts and protein abundance of Fbxw24 in mouse fertilized embryos.*$P < 0.05$. (**D**) The mRNA level of Fbxw24 in different tissues of the mouse. GAPDH was used as a control. (**E**) Immunoblots of lysates isolated from different tissues of C57BL/6J-*Fbxw24*$^{em(Linker\text{-}3xFlag)}$ mice. GAPDH used as a loading control. (**F**) Immunohistochemical analysis of Fbxw24 in ovary. The sections obtained from

4-week-old C57BL/6J-*Fbxw24*$^{em(Linker\text{-}3xFlag)}$ heterozygous female mice. The wild-type female littermate was a negative control. The representative oocytes with positivity at different follicular stages are indicated by red arrows and granulosa cells with positivity are indicated by red arrowheads. (Magnification: 100×,400×).

Figure 7. The function analysis of Fbxw24 during mouse embryo development. (**A**) Efficiency of siRNA-mediated knockdown of Fbxw24. SD is shown by the error bars. (**B**) Percentage of successfully developed embryos at each stage following Fbxw24 knockdown. The development rate is calculated with the quantity of zygotes as the denominator. SD is shown by the error bars. (**C**) Knockdown of Fbxw24 results in the developmental arrest that started from E1.5. Scale bars represent 100 μm.

Figure 8. Fbxw24 interact with Ddb1-Cul4b in mouse. (**A**) Fbxw24 was pulled down and eluted by an N-terminal 3xFlag tag. An SDS-PAGE was used to isolate the extracts obtained from the 3xFlag-target, then the extracts were observed by Coomassie brilliant blue staining. Extraction of seven Fbxw24 immunoprecipitated products were performed for subsequent in-gel trypsin digestion. (**B**) Sixteen members of ubiquitin-mediated proteolysis pathways were identified in Fbxw24 pull-down complexes by LC-MS/MS analysis. (**C**) A Co-IP assay in HEK-293T cells and ovaries of C57BL/6J-*Fbxw24*$^{em\ (Linker\text{-}3xFlag)}$ mice was conducted to validate the interaction of Ddb1-Cul4b with Fbxw24. NC: Negative Control; OE: Overexpression; WT: Wild Type mice; HO: Homozygote mice. (**D**) The expression pattern analysis of

sixteen members identified in Fbxw24 pull-down complexes by MS analysis and verification by PRM in fertilized embryos. The red line displayed the change of protein level from the previously reported proteome of mouse fertilized embryos identified by Gao, and the blue line displayed the results of PRM analysis in this study. The pronucleus stage was used as a control.

Tables

Table 1. The expression correlation coefficient of Fbxws and SCMC components.

Additional files

Table S1. Summary of maternal protein and peptide profiles identified in mouse uniparental embryos.

Table S2. The detailed information of UpSet intersection diagram and the annotation of each intersection for the proteome of mouse uniparental embryos.

Table S3. The detailed results of Fuzzy c-means clustering analysis using the proteome of mouse uniparental embryos.

Table S4. The detailed results of Venn diagram in mouse mature oocytes (MII), fertilized (ZY) and parthenogenetic (PA) embryos.

Table S5. The detailed results of Fuzzy c-means clustering analysis related to Fig.3.

Table S6. The results of expression correlation for 15 key maternal proteins and other quantified proteins in PA and ZY groups.

Table S7. The maternal proteins that are strongly correlated with SCMC components

in PA and ZY groups.

Table S8. The detailed results of Venn diagram related to Fig.5A.

Table S9. Primers for Realtime PCR and target sequence of siRNAs.

Table S10. Putative Fbxw24 interactors identified by Fbxw24 IP-MS.

Table S11. The parallel reaction monitoring (PRM) validation results of selected proteins.

Figure S1. (A) The developmental morphology of different stages after activation of ethanol combined with CB. The visualization of DNA was performed using DAPI (blue). Scale bar: 20μm. (B) The developmental rate after activation of ethanol combined with CB in mouse oocyte.

Figure S2. Annotation for maternal protein expression clusters in mouse uniparental embryos.(A).The heat map showed the significance value of the biological processes in 10 clusters.(B-D).The interaction networks of GO terms from four categories are displayed, including “translation”(B), “peptide metabolic process”(C), “nucleic acid metabolic process” (D) and “cellular metabolic process” (E).

Figure S3. (A).The protein-protein interaction (PPI) network of the candidate maternal proteins that are strongly negative with the SCMCs. (B). The GO and KEGG annotation for the the candidate maternal proteins that are strongly negative with the SCMCs.

Figure S4. (A) The Venn diagram showed the intersection of the candidate proteins that strongly correlated with 15 key maternal proteins in two reported proteome databases of fertilized embryos identified by Gao and Israel, respectively. (B-D) The

UpSet intersection diagram of 15 key maternal proteins and their candidate proteins (|r| ≥ 0.70 and p≤0.05) in PA group (B) and ZY group (C-identified by Gao, D-identified by Israel).

References

1. Li L, Zheng P, Dean J. Maternal control of early mouse development. Development. 2010;137:859–70. doi:10.1242/dev.039487.
2. Burns KH, Viveiros MM, Ren Y, Wang P, DeMayo FJ, Frail DE, et al. Roles of NPM2 in chromatin and nucleolar organization in oocytes and embryos. Science. 2003;300:633–6. doi:10.1126/science.1081813.
3. Gao Z, Zhang X, Yu X, Qin D, Xiao Y, Yu Y, et al. Zbed3 participates in the subcortical maternal complex and regulates the distribution of organelles. J Mol Cell Biol. 2018;10:74–88. doi:10.1093/jmcb/mjx035.
4. Mahadevan S, Sathappan V, Utama B, Lorenzo I, Kaskar K, van den Veyver IB. Maternally expressed NLRP2 links the subcortical maternal complex (SCMC) to fertility, embryogenesis and epigenetic reprogramming. Sci Rep. 2017;7:44667. doi:10.1038/srep44667.
5. Tashiro F, Kanai-Azuma M, Miyazaki S, Kato M, Tanaka T, Toyoda S, et al. Maternal-effect gene Ces5/Ooep/Moep19/Floped is essential for oocyte cytoplasmic lattice formation and embryonic development at the maternal-zygotic stage transition. Genes Cells. 2010;15:813–28. doi:10.1111/j.1365-2443.2010.01420.x.

6. Yu X-J, Yi Z, Gao Z, Qin D, Zhai Y, Chen X, et al. The subcortical maternal complex controls symmetric division of mouse zygotes by regulating F-actin dynamics. Nat Commun. 2014;5:4887. doi:10.1038/ncomms5887.
7. Tong ZB, Gold L, Pfeifer KE, Dorward H, Lee E, Bondy CA, et al. Mater, a maternal effect gene required for early embryonic development in mice. Nat Genet. 2000;26:267–8. doi:10.1038/81547.
8. Yurttas P, Vitale AM, Fitzhenry RJ, Cohen-Gould L, Wu W, Gossen JA, Coonrod SA. Role for PADI6 and the cytoplasmic lattices in ribosomal storage in oocytes and translational control in the early mouse embryo. Development. 2008;135:2627–36. doi:10.1242/dev.016329.
9. Fan X, Tang D, Liao Y, Li P, Zhang Y, Wang M, et al. Single-cell RNA-seq analysis of mouse preimplantation embryos by third-generation sequencing. PLoS Biol. 2020;18:e3001017. doi:10.1371/journal.pbio.3001017.
10. Xue Z, Huang K, Cai C, Cai L, Jiang C, Feng Y, et al. Genetic programs in human and mouse early embryos revealed by single-cell RNA sequencing. Nature. 2013;500:593–7. doi:10.1038/nature12364.
11. Israel S, Ernst M, Psathaki OE, Drexler HCA, Casser E, Suzuki Y, et al. An integrated genome-wide multi-omics analysis of gene expression dynamics in the preimplantation mouse embryo. Sci Rep. 2019;9:13356. doi:10.1038/s41598-019-49817-3.
12. Deutsch DR, Fröhlich T, Otte KA, Beck A, Habermann FA, Wolf E, Arnold GJ. Stage-specific proteome signatures in early bovine embryo development. J

Proteome Res. 2014;13:4363–76. doi:10.1021/pr500550t.

13. Lucitt MB, Price TS, Pizarro A, Wu W, Yocum AK, Seiler C, et al. Analysis of the zebrafish proteome during embryonic development. Mol Cell Proteomics. 2008;7:981–94. doi:10.1074/mcp.M700382-MCP200.
14. Peshkin L, Wühr M, Pearl E, Haas W, Freeman RM, Gerhart JC, et al. On the Relationship of Protein and mRNA Dynamics in Vertebrate Embryonic Development. Dev Cell. 2015;35:383–94. doi:10.1016/j.devcel.2015.10.010.
15. Wühr M, Freeman RM, Presler M, Horb ME, Peshkin L, Gygi S, Kirschner MW. Deep proteomics of the Xenopus laevis egg using an mRNA-derived reference database. Curr Biol. 2014;24:1467–75. doi:10.1016/j.cub.2014.05.044.
16. Wang S, Kou Z, Jing Z, Zhang Y, Guo X, Dong M, et al. Proteome of mouse oocytes at different developmental stages. Proc Natl Acad Sci U S A. 2010;107:17639–44. doi:10.1073/pnas.1013185107.
17. Gao Y, Liu X, Tang B, Li C, Kou Z, Li L, et al. Protein Expression Landscape of Mouse Embryos during Pre-implantation Development. Cell Rep. 2017;21:3957–69. doi:10.1016/j.celrep.2017.11.111.
18. Israel S, Casser E, Drexler HCA, Fuellen G, Boiani M. A framework for TRIM21-mediated protein depletion in early mouse embryos: recapitulation of Tead4 null phenotype over three days. BMC Genomics. 2019;20:755. doi:10.1186/s12864-019-6106-2.
19. Kaufman MH. Parthenogenetic Activation of Oocytes. Cold Spring Harb Protoc 2018. doi:10.1101/pdb.prot094409.

20. Cuellar O. Animal parthenogenesis. Science. 1977;197:837–43. doi:10.1126/science.887925.

21. Brevini TAL, Pennarossa G, Vanelli A, Maffei S, Gandolfi F. Parthenogenesis in non-rodent species: developmental competence and differentiation plasticity. Theriogenology. 2012;77:766–72. doi:10.1016/j.theriogenology.2011.11.010.

22. Peng M, Huang H, Jin F. Progress in research on oocytes parthenogenetic activation. Zhejiang Da Xue Xue Bao Yi Xue Ban. 2007;36:307–12. doi:10.3785/j.issn.1008-9292.2007.03.017.

23. Tachibana M, Amato P, Sparman M, Gutierrez NM, Tippner-Hedges R, Ma H, et al. Human embryonic stem cells derived by somatic cell nuclear transfer. Cell. 2013;153:1228–38. doi:10.1016/j.cell.2013.05.006.

24. Ahn J, Hwang I-S, Park M-R, Cho I-C, Hwang S, Lee K. The Landscape of Genomic Imprinting at the Porcine SGCE/PEG10 Locus from Methylome and Transcriptome of Parthenogenetic Embryos. G3 (Bethesda). 2020;10:4037–47. doi:10.1534/g3.120.401425.

25. Ahn J, Wu H, Lee J, Hwang I-S, Yu D, Ahn J-S, et al. Identification of a Novel Imprinted Transcript in the Porcine GNAS Complex Locus Using Methylome and Transcriptome of Parthenogenetic Fetuses. Genes (Basel) 2020. doi:10.3390/genes11010096.

26. Chen F, Fu Q, Pu L, Zhang P, Huang Y, Hou Z, et al. Integrated Analysis of Quantitative Proteome and Transcriptional Profiles Reveals the Dynamic Function of Maternally Expressed Proteins After Parthenogenetic Activation of

Buffalo Oocyte. Mol Cell Proteomics. 2018;17:1875–91. doi:10.1074/mcp.RA118.000556.

27. Du Z-Q, Liang H, Liu X-M, Liu Y-H, Wang C, Yang C-X. Single cell RNA-seq reveals genes vital to in vitro fertilized embryos and parthenotes in pigs. Sci Rep. 2021;11:14393. doi:10.1038/s41598-021-93904-3.
28. Kajdasz A, Warzych E, Derebecka N, Madeja ZE, Lechniak D, Wesoly J, Pawlak P. Lipid Stores and Lipid Metabolism Associated Gene Expression in Porcine and Bovine Parthenogenetic Embryos Revealed by Fluorescent Staining and RNA-seq. Int J Mol Sci 2020. doi:10.3390/ijms21186488.
29. Leng L, Sun J, Huang J, Gong F, Yang L, Zhang S, et al. Single-Cell Transcriptome Analysis of Uniparental Embryos Reveals Parent-of-Origin Effects on Human Preimplantation Development. Cell Stem Cell. 2019;25:697-712.e6. doi:10.1016/j.stem.2019.09.004.
30. Zhang C, Li C, Yang L, Leng L, Jovic D, Wang J, et al. The Dynamic Changes of Transcription Factors During the Development Processes of Human Biparental and Uniparental Embryos. Front Cell Dev Biol. 2021;9:709498. doi:10.3389/fcell.2021.709498.
31. Lu X, Gao Z, Qin D, Li L. A Maternal Functional Module in the Mammalian Oocyte-To-Embryo Transition. Trends Mol Med. 2017;23:1014–23. doi:10.1016/j.molmed.2017.09.004.
32. Zaccara S, Ries RJ, Jaffrey SR. Reading, writing and erasing mRNA methylation. Nat Rev Mol Cell Biol. 2019;20:608–24. doi:10.1038/s41580-019-0168-5.

33. Li X, Ito M, Zhou F, Youngson N, Zuo X, Leder P, Ferguson-Smith AC. A maternal-zygotic effect gene, Zfp57, maintains both maternal and paternal imprints. Dev Cell. 2008;15:547–57. doi:10.1016/j.devcel.2008.08.014.
34. Posfai E, Kunzmann R, Brochard V, Salvaing J, Cabuy E, Roloff TC, et al. Polycomb function during oogenesis is required for mouse embryonic development. Genes Dev. 2012;26:920–32. doi:10.1101/gad.188094.112.
35. Nakamura T, Arai Y, Umehara H, Masuhara M, Kimura T, Taniguchi H, et al. PGC7/Stella protects against DNA demethylation in early embryogenesis. Nat Cell Biol. 2007;9:64–71. doi:10.1038/ncb1519.
36. Otten ABC, Kamps R, Lindsey P, Gerards M, Pendeville-Samain H, Muller M, et al. Tfam Knockdown Results in Reduction of mtDNA Copy Number, OXPHOS Deficiency and Abnormalities in Zebrafish Embryos. Front Cell Dev Biol. 2020;8:381. doi:10.3389/fcell.2020.00381.
37. Yan W, Ma L, Stein P, Pangas SA, Burns KH, Bai Y, et al. Mice deficient in oocyte-specific oligoadenylate synthetase-like protein OAS1D display reduced fertility. Mol Cell Biol. 2005;25:4615–24. doi:10.1128/MCB.25.11.4615-4624.2005.
38. Fukuda T, Tokunaga A, Sakamoto R, Yoshida N. Fbxl10/Kdm2b deficiency accelerates neural progenitor cell death and leads to exencephaly. Mol Cell Neurosci. 2011;46:614–24. doi:10.1016/j.mcn.2011.01.001.
39. Zheng P, Dean J. Role of Filia, a maternal effect gene, in maintaining euploidy during cleavage-stage mouse embryogenesis. Proc Natl Acad Sci U S A.

2009;106:7473–8. doi:10.1073/pnas.0900519106.

40. Hirasawa R, Chiba H, Kaneda M, Tajima S, Li E, Jaenisch R, Sasaki H. Maternal and zygotic Dnmt1 are necessary and sufficient for the maintenance of DNA methylation imprints during preimplantation development. Genes Dev. 2008;22:1607–16. doi:10.1101/gad.1667008.

41. Inoue A, Chen Z, Yin Q, Zhang Y. Maternal Eed knockout causes loss of H3K27me3 imprinting and random X inactivation in the extraembryonic cells. Genes Dev. 2018;32:1525–36. doi:10.1101/gad.318675.118.

42. O'Carroll D, Erhardt S, Pagani M, Barton SC, Surani MA, Jenuwein T. The polycomb-group gene Ezh2 is required for early mouse development. Mol Cell Biol. 2001;21:4330–6. doi:10.1128/MCB.21.13.4330-4336.2001.

43. Larue L, Ohsugi M, Hirchenhain J, Kemler R. E-cadherin null mutant embryos fail to form a trophectoderm epithelium. Proc Natl Acad Sci U S A. 1994;91:8263–7. doi:10.1073/pnas.91.17.8263.

44. Bultman SJ, Gebuhr TC, Pan H, Svoboda P, Schultz RM, Magnuson T. Maternal BRG1 regulates zygotic genome activation in the mouse. Genes Dev. 2006;20:1744–54. doi:10.1101/gad.1435106.

45. Maenohara S, Unoki M, Toh H, Ohishi H, Sharif J, Koseki H, Sasaki H. Role of UHRF1 in de novo DNA methylation in oocytes and maintenance methylation in preimplantation embryos. PLoS Genet. 2017;13:e1007042. doi:10.1371/journal.pgen.1007042.

46. Li R, Albertini DF. The road to maturation: somatic cell interaction and

self-organization of the mammalian oocyte. Nat Rev Mol Cell Biol. 2013;14:141–52. doi:10.1038/nrm3531.

47. Bebbere D, Masala L, Albertini DF, Ledda S. The subcortical maternal complex: multiple functions for one biological structure? J Assist Reprod Genet. 2016;33:1431–8. doi:10.1007/s10815-016-0788-z.

48. Akoglu H. User's guide to correlation coefficients. Turk J Emerg Med. 2018;18:91–3. doi:10.1016/j.tjem.2018.08.001.

49. Toralova T, Kinterova V, Chmelikova E, Kanka J. The neglected part of early embryonic development: maternal protein degradation. Cell Mol Life Sci. 2020;77:3177–94. doi:10.1007/s00018-020-03482-2.

50. Huo L-J, Fan H-Y, Zhong Z-S, Chen D-Y, Schatten H, Sun Q-Y. Ubiquitin-proteasome pathway modulates mouse oocyte meiotic maturation and fertilization via regulation of MAPK cascade and cyclin B1 degradation. Mech Dev. 2004;121:1275–87. doi:10.1016/j.mod.2004.05.007.

51. Tsukamoto S, Kuma A, Murakami M, Kishi C, Yamamoto A, Mizushima N. Autophagy is essential for preimplantation development of mouse embryos. Science. 2008;321:117–20. doi:10.1126/science.1154822.

52. Yamamoto A, Mizushima N, Tsukamoto S. Fertilization-induced autophagy in mouse embryos is independent of mTORC1. Biol Reprod. 2014;91:7. doi:10.1095/biolreprod.113.115816.

53. Higuchi C, Shimizu N, Shin S-W, Morita K, Nagai K, Anzai M, et al. Ubiquitin-proteasome system modulates zygotic genome activation in early

mouse embryos and influences full-term development. J Reprod Dev. 2018;64:65–74. doi:10.1262/jrd.2017-127.

54. Akoury E, Gupta N, Bagga R, Brown S, Déry C, Kabra M, et al. Live births in women with recurrent hydatidiform mole and two NLRP7 mutations. Reprod Biomed Online. 2015;31:120–4. doi:10.1016/j.rbmo.2015.03.011.
55. Li L, Baibakov B, Dean J. A subcortical maternal complex essential for preimplantation mouse embryogenesis. Dev Cell. 2008;15:416–25. doi:10.1016/j.devcel.2008.07.010.
56. Peng H, Chang B, Lu C, Su J, Wu Y, Lv P, et al. Nlrp2, a maternal effect gene required for early embryonic development in the mouse. PLoS One. 2012;7:e30344. doi:10.1371/journal.pone.0030344.
57. Angers S, Li T, Yi X, MacCoss MJ, Moon RT, Zheng N. Molecular architecture and assembly of the DDB1-CUL4A ubiquitin ligase machinery. Nature. 2006;443:590–3. doi:10.1038/nature05175.
58. Jackson S, Xiong Y. CRL4s: the CUL4-RING E3 ubiquitin ligases. Trends Biochem Sci. 2009;34:562–70. doi:10.1016/j.tibs.2009.07.002.
59. Yu C, Zhang Y-L, Pan W-W, Li X-M, Wang Z-W, Ge Z-J, et al. CRL4 complex regulates mammalian oocyte survival and reprogramming by activation of TET proteins. Science. 2013;342:1518–21. doi:10.1126/science.1244587.
60. Anderson KV, Nüsslein-Volhard C. Information for the dorsal—ventral pattern of the Drosophila embryo is stored as maternal mRNA. Nature. 1984;311:223–7. doi:10.1038/311223a0.

61. Anderson KV, Bokla L, Nüsslein-Volhard C. Establishment of dorsal-ventral polarity in the drosophila embryo: The induction of polarity by the Toll gene product. Cell. 1985;42:791–8. doi:10.1016/0092-8674(85)90275-2.
62. Driever W, Nüsslein-Volhard C. The bicoid protein determines position in the Drosophila embryo in a concentration-dependent manner. Cell. 1988;54:95–104. doi:10.1016/0092-8674(88)90183-3.
63. Nüsslein-Volhard C, Lohs-Schardin M, Sander K, Cremer C. A dorso-ventral shift of embryonic primordia in a new maternal-effect mutant of Drosophila. Nature. 1980;283:474–6. doi:10.1038/283474a0.
64. Nüsslein-Volhard C, Frohnhöfer HG, Lehmann R. Determination of anteroposterior polarity in Drosophila. Science. 1987;238:1675–81. doi:10.1126/science.3686007.
65. Nüsslein-Volhard C, Wieschaus E. Mutations affecting segment number and polarity in Drosophila. Nature. 1980;287:795–801. doi:10.1038/287795a0.
66. Schupbach T, Wieschaus E. Germline autonomy of maternal-effect mutations altering the embryonic body pattern of Drosophila. Developmental Biology. 1986;113:443–8. doi:10.1016/0012-1606(86)90179-x.
67. Zheng W, Liu K. Maternal control of mouse preimplantation development. Results Probl Cell Differ. 2012;55:115–39. doi:10.1007/978-3-642-30406-4_7.
68. Chen Y, Ai A, Tang ZY, Zhou GD, Liu W, Cao Y, Zhang WJ. Mesenchymal-like stem cells derived from human parthenogenetic embryonic stem cells. Stem Cells Dev. 2012;21:143–51. doi:10.1089/scd.2010.0585.

69. Elling U, Taubenschmid J, Wirnsberger G, O'Malley R, Demers S-P, Vanhaelen Q, et al. Forward and reverse genetics through derivation of haploid mouse embryonic stem cells. Cell Stem Cell. 2011;9:563–74. doi:10.1016/j.stem.2011.10.012.

70. Leeb M, Wutz A. Derivation of haploid embryonic stem cells from mouse embryos. Nature. 2011;479:131–4. doi:10.1038/nature10448.

71. Yang H, Liu Z, Ma Y, Zhong C, Yin Q, Zhou C, et al. Generation of haploid embryonic stem cells from Macaca fascicularis monkey parthenotes. Cell Res. 2013;23:1187–200. doi:10.1038/cr.2013.93.

72. Wei Y, Yang C-R, Zhao Z-A. Viable offspring derived from single unfertilized mammalian oocytes. Proc Natl Acad Sci U S A. 2022;119:e2115248119. doi:10.1073/pnas.2115248119.

73. Knowles BB, Evsikov AV, Vries WN de, Peaston AE, Solter D. Molecular control of the oocyte to embryo transition. Philos Trans R Soc Lond B Biol Sci. 2003;358:1381–7. doi:10.1098/rstb.2003.1330.

74. La Chesnaye E de, Kerr B, Paredes A, Merchant-Larios H, Méndez JP, Ojeda SR. Fbxw15/Fbxo12J is an F-box protein-encoding gene selectively expressed in oocytes of the mouse ovary. Biol Reprod. 2008;78:714–25. doi:10.1095/biolreprod.107.063826.

75. Mahadevan S, Sathappan V, Utama B, Lorenzo I, Kaskar K, van den Veyver IB. Erratum: Maternally expressed NLRP2 links the subcortical maternal complex (SCMC) to fertility, embryogenesis and epigenetic reprogramming. Sci Rep.

2017;7:46434. doi:10.1038/srep46434.

76. Yurttas P, Morency E, Coonrod SA. Use of proteomics to identify highly abundant maternal factors that drive the egg-to-embryo transition. Reproduction. 2010;139:809–23. doi:10.1530/REP-09-0538.

77. Wiśniewski JR, Zougman A, Nagaraj N, Mann M. Universal sample preparation method for proteome analysis. Nat Methods. 2009;6:359–62. doi:10.1038/nmeth.1322.

78. Ma J, Chen T, Wu S, Yang C, Bai M, Shu K, et al. iProX: an integrated proteome resource. Nucleic Acids Res. 2019;47:D1211-D1217. doi:10.1093/nar/gky869.

79. Conesa A, Götz S, García-Gómez JM, Terol J, Talón M, Robles M. Blast2GO: a universal tool for annotation, visualization and analysis in functional genomics research. Bioinformatics. 2005;21:3674–6. doi:10.1093/bioinformatics/bti610.

80. Szklarczyk D, Franceschini A, Wyder S, Forslund K, Heller D, Huerta-Cepas J, et al. STRING v10: protein-protein interaction networks, integrated over the tree of life. Nucleic Acids Res. 2015;43:D447-52. doi:10.1093/nar/gku1003.

81. Shannon P, Markiel A, Ozier O, Baliga NS, Wang JT, Ramage D, et al. Cytoscape: a software environment for integrated models of biomolecular interaction networks. Genome Res. 2003;13:2498–504. doi:10.1101/gr.1239303.

82. MacLean B, Tomazela DM, Shulman N, Chambers M, Finney GL, Frewen B, et al. Skyline: an open source document editor for creating and analyzing targeted proteomics experiments. Bioinformatics. 2010;26:966–8. doi:10.1093/bioinformatics/btq054.

83. Ward MA, Yanagimachi R. Intracytoplasmic Sperm Injection in Mice. Cold Spring Harb Protoc 2018. doi:10.1101/pdb.prot094482.

84. Mihalas BP, Bromfield EG, Sutherland JM, Iuliis GN de, McLaughlin EA, Aitken RJ, Nixon B. Oxidative damage in naturally aged mouse oocytes is exacerbated by dysregulation of proteasomal activity. J Biol Chem. 2018;293:18944–64. doi:10.1074/jbc.RA118.005751.

85. Ong S-E, Mann M. A practical recipe for stable isotope labeling by amino acids in cell culture (SILAC). Nat Protoc. 2006;1:2650–60. doi:10.1038/nprot.2006.427.

86. Ong S-E, Blagoev B, Kratchmarova I, Kristensen DB, Steen H, Pandey A, Mann M. Stable isotope labeling by amino acids in cell culture, SILAC, as a simple and accurate approach to expression proteomics. Mol Cell Proteomics. 2002;1:376–86. doi:10.1074/mcp.m200025-mcp200.

87. Shevchenko A, Tomas H, Havlis J, Olsen JV, Mann M. In-gel digestion for mass spectrometric characterization of proteins and proteomes. Nat Protoc. 2006;1:2856–60. doi:10.1038/nprot.2006.468.

88. Cox J, Mann M. MaxQuant enables high peptide identification rates, individualized p.p.b.-range mass accuracies and proteome-wide protein quantification. Nat Biotechnol. 2008;26:1367–72. doi:10.1038/nbt.1511.

89. Cox J, Neuhauser N, Michalski A, Scheltema RA, Olsen JV, Mann M. Andromeda: a peptide search engine integrated into the MaxQuant environment. J Proteome Res. 2011;10:1794–805. doi:10.1021/pr101065j.

Table 1 The expression correlation coefficient of Fbxws and SCMC components

Gene name	Groups	Ooep		Nlrp5		Tle6		Fbxw24	
		p	r	p	r	p	r	p	r
Fbxw8	PA_this study	—	—	—	—	—	—	—	—
	ZY_Israel	0.5822	0.2864	0.6216	0.2580	0.3570	0.4614	0.2948	0.5159
	ZY_Gao	0.0009	-0.9749	0.0000	-0.9946	0.0002	-0.9889	0.0021	-0.9628
Fbxw11	PA_this study	0.7422	0.1736	0.5370	0.3195	0.7132	0.1936	—	—
	ZY_Israel	0.0570	-0.7981	0.0067	-0.9325	0.0287	-0.8582	0.0502	-0.8109
	ZY_Gao	0.0561	-0.7997	0.1031	-0.7250	0.0924	-0.7404	0.0346	-0.8440
Fbxw13	PA_this study	—	—	—	—	—	—	—	—
	ZY_Israel	0.3686	0.4517	0.1762	0.6342	0.3035	0.5081	0.1168	0.7062
	ZY_Gao	0.0000	0.9949	0.0009	0.9755	0.0004	0.9837	0.0002	0.9898
Fbxw15	PA_this study	0.0111	-0.9128	0.0247	-0.8687	0.0063	-0.9342	—	—
	ZY_Israel	0.0137	0.9027	0.0153	0.8972	0.0228	0.8742	0.0009	0.9755
	ZY_Gao	0.0010	0.9742	0.0037	0.9499	0.0021	0.9626	0.0014	0.9696
Fbxw16	PA_this study	—	—	—	—	—	—	—	—
	ZY_Israel	0.0246	0.8689	0.0135	0.9037	0.0680	0.7787	0.0090	0.9214
	ZY_Gao	0.0003	0.9865	0.0019	0.9645	0.0010	0.9744	0.0002	0.9895
Fbxw18	PA_this study	—	—	—	—	—	—	—	—
	ZY_Israel	0.0057	0.9376	0.0065	0.9336	0.0208	0.8797	0.0013	0.9708
	ZY_Gao	0.0005	0.9810	0.0044	0.9452	0.0026	0.9585	0.0008	0.9775
Fbxw19	PA_this study	—	—	—	—	—	—	—	—
	ZY_Israel	0.0329	0.8479	0.0299	0.8554	0.0337	0.8461	0.0486	0.8141
	ZY_Gao	0.0000	0.9978	0.0003	0.9850	0.0001	0.9909	0.0001	0.9942
Fbxw20	PA_this study	—	—	—	—	—	—	—	—
	ZY_Israel	0.0462	0.8189	0.0308	0.8530	0.0325	0.8491	0.0025	0.9585
	ZY_Gao	0.0001	0.9917	0.0013	0.9705	0.0006	0.9798	0.0002	0.9896
Fbxw21	PA_this study	—	—	—	—	—	—	—	—
	ZY_Israel	0.0178	0.8891	0.0019	0.9644	0.0090	0.9216	0.0019	0.9638
	ZY_Gao	0.0000	0.9944	0.0008	0.9772	0.0003	0.9851	0.0001	0.9923
Fbxw22	PA_this study	—	—	—	—	—	—	—	—
	ZY_Israel	0.0040	0.9480	0.0037	0.9497	0.0251	0.8677	0.0044	0.9454
	ZY_Gao	0.0001	0.9931	0.0006	0.9795	0.0003	0.9865	0.0002	0.9893
Fbxw26	PA_this study	—	—	—	—	—	—	—	—
	ZY_Israel	0.0328	0.8482	0.0438	0.8238	0.0175	0.8900	0.0640	0.7857
	ZY_Gao	0.0000	0.9947	0.0002	0.9871	0.0001	0.9921	0.0000	0.9967
Fbxw28	PA_this study	—	—	—	—	—	—	—	—
	ZY_Israel	0.0209	0.8796	0.0238	0.8712	0.0095	0.9192	0.0107	0.9142
	ZY_Gao	0.0021	0.9621	0.0071	0.9303	0.0044	0.9451	0.0035	0.9510

Figure.1

Click here to access/download;Figure;Figure.1.tif

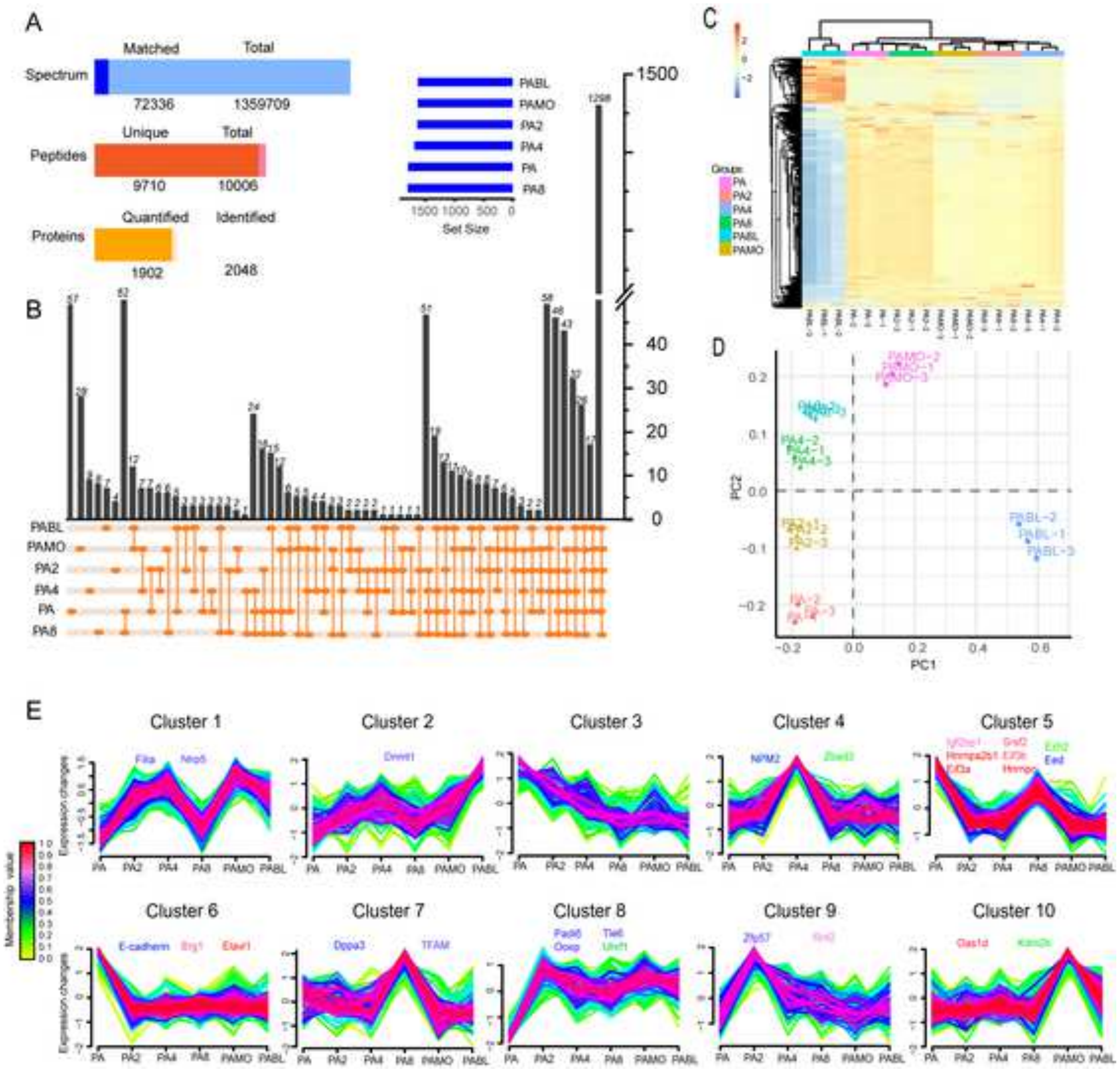

Figure.2

Click here to access/download;Figure;Figure.2.tif

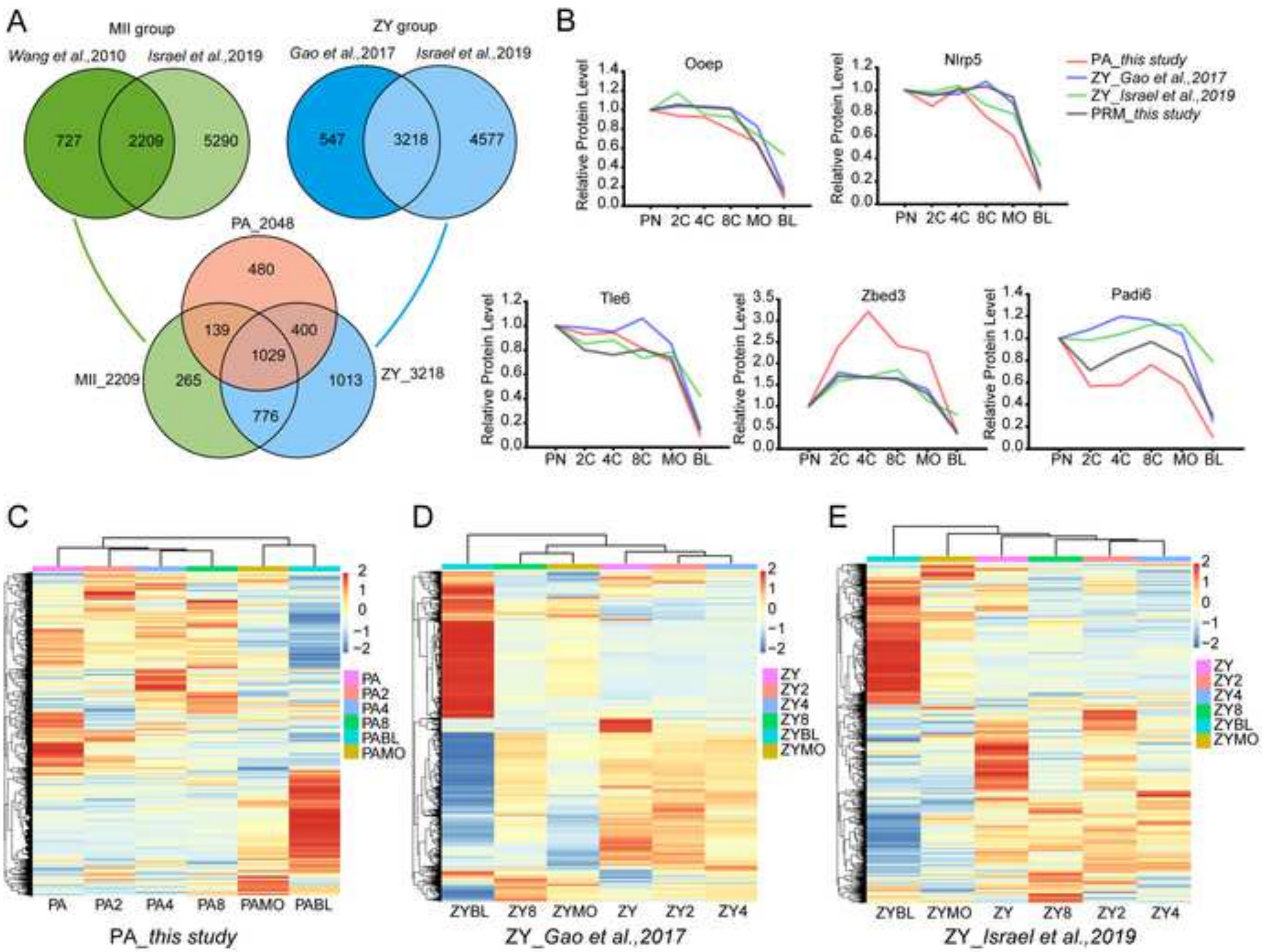

Figure.3

Click here to access/download;Figure;Figure.3.tif

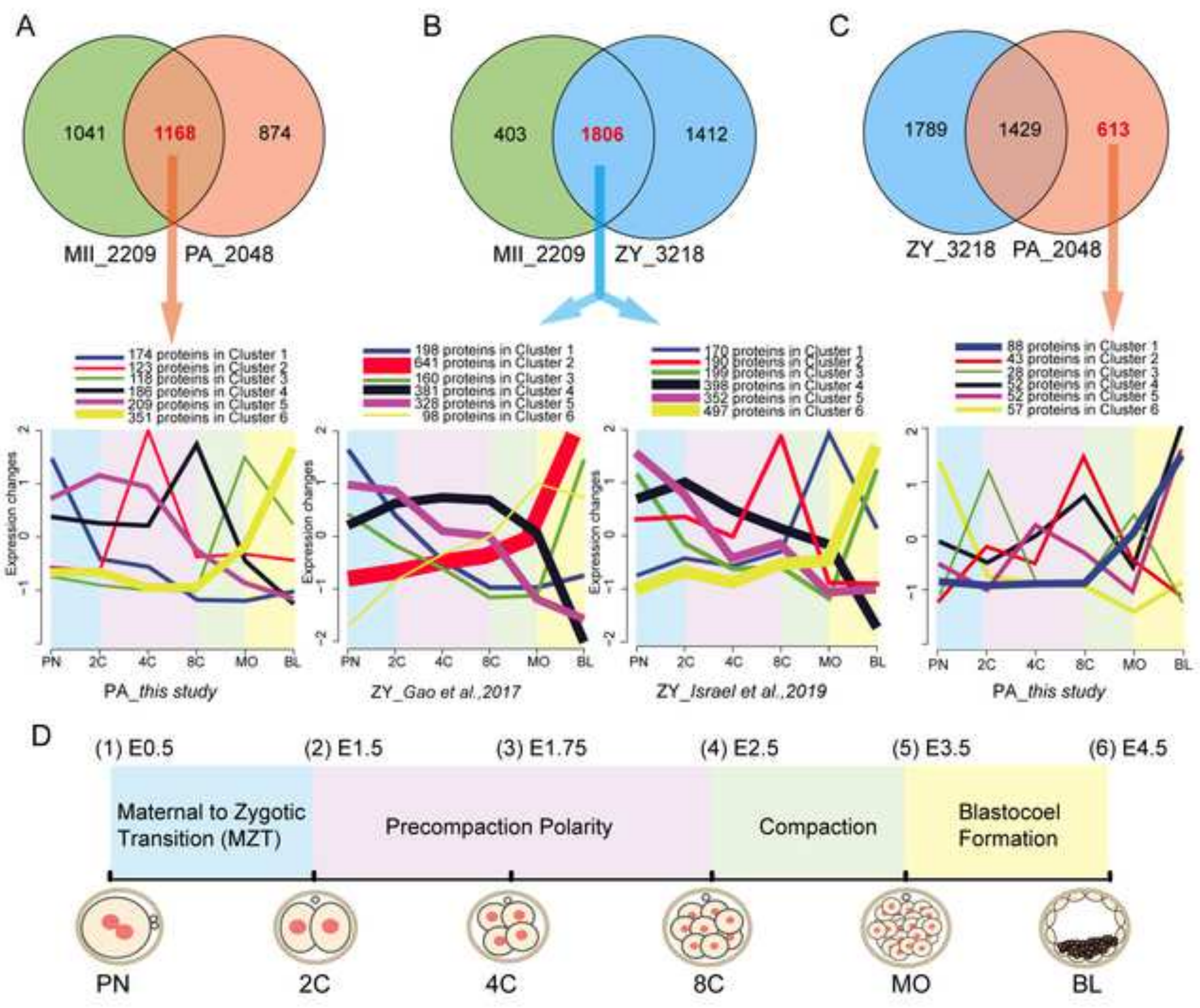

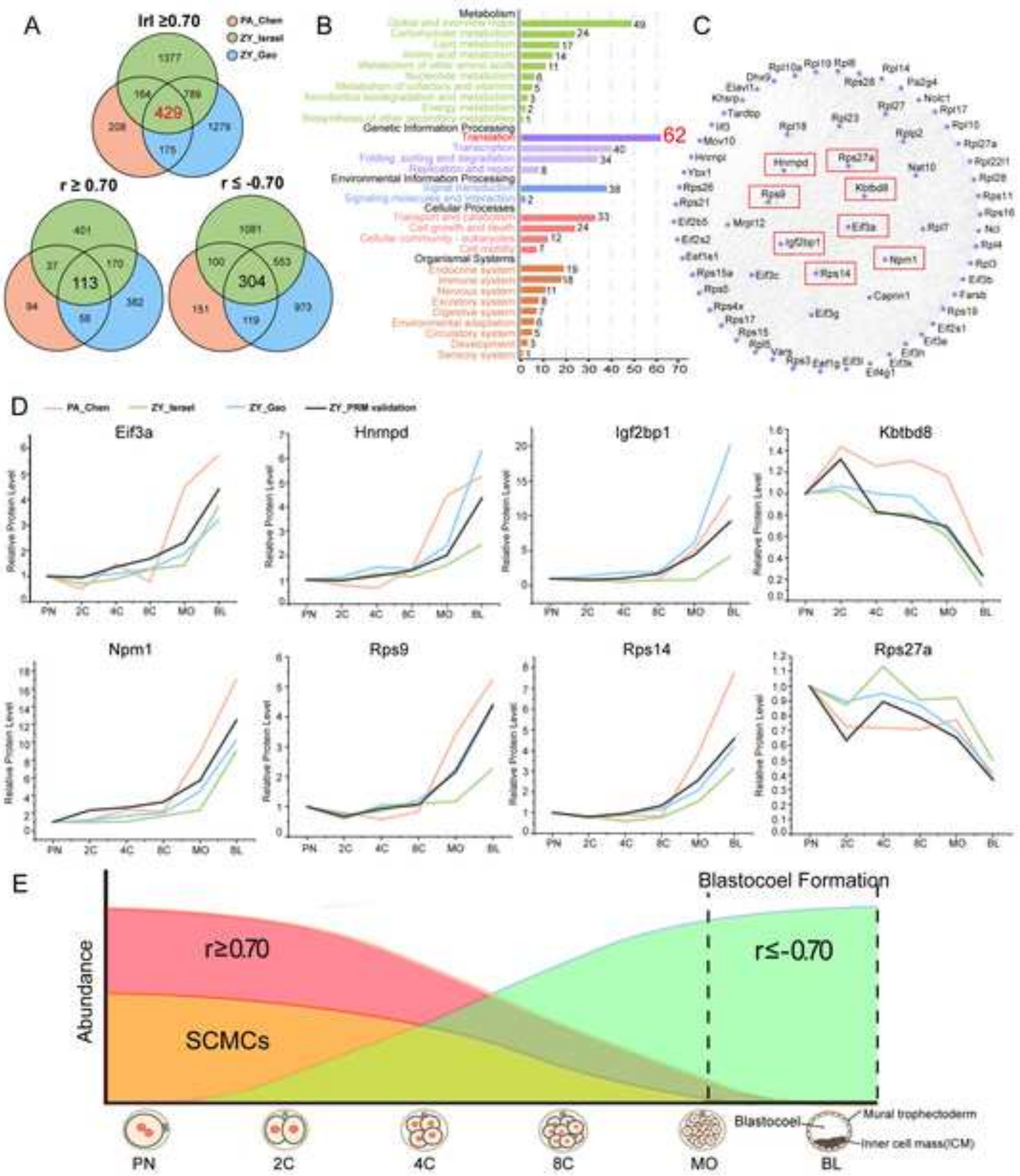
A
|r| ≥0.70
PA_Chen
ZY_Israel
ZY_Gao
r ≥ 0.70
r ≤ -0.70
B
C
D
E
Blastocoel Formation
r≥0.70
r≤-0.70
SCMCs
Abundance
PN
2C
4C
8C
MO
BL
Blastocoel
Mural trophectoderm
Inner cell mass(ICM)

Figure.5

Click here to access/download;Figure;Figure.5.tif

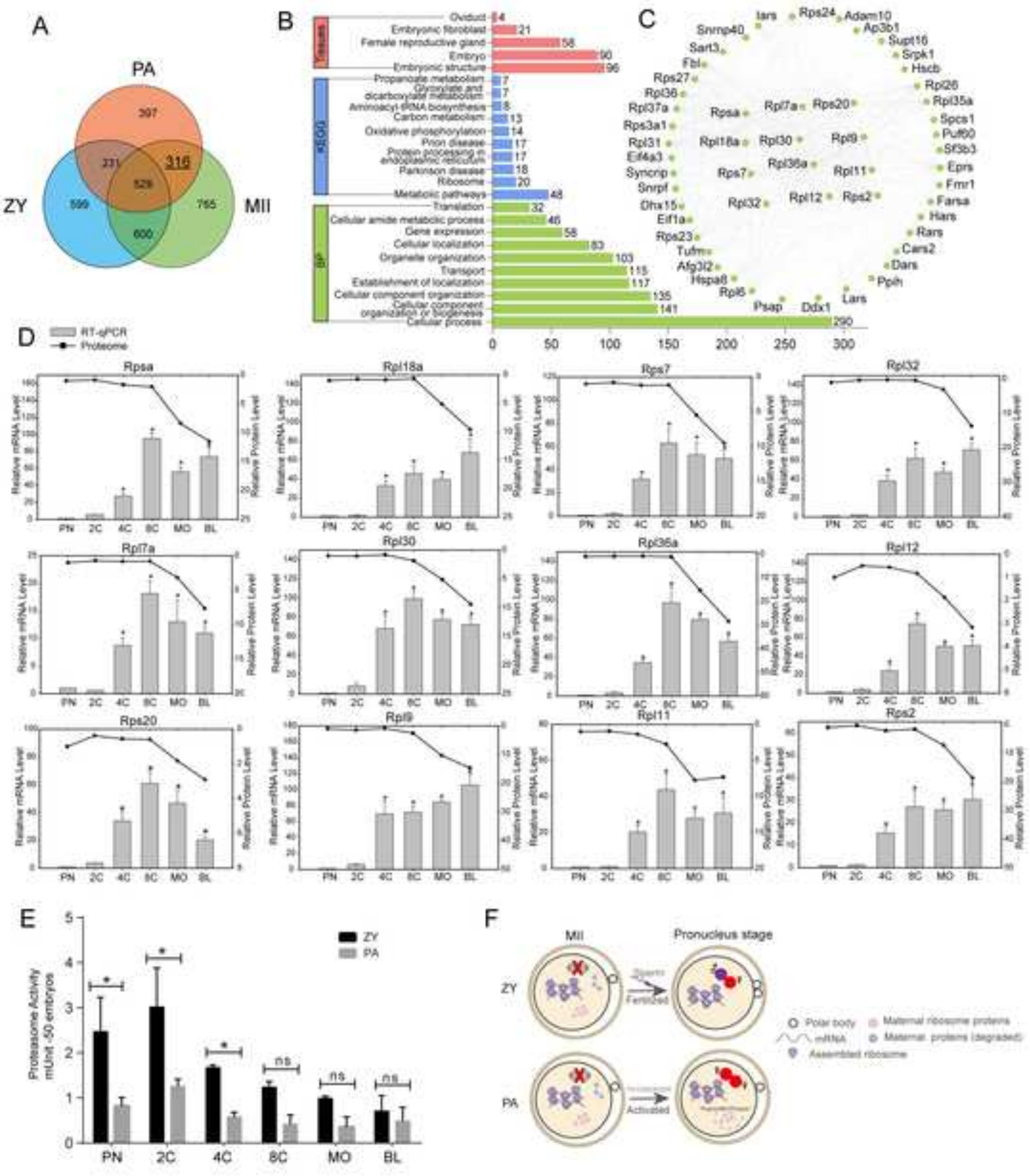

Figure.6

Click here to access/download;Figure;Figure.6.tif

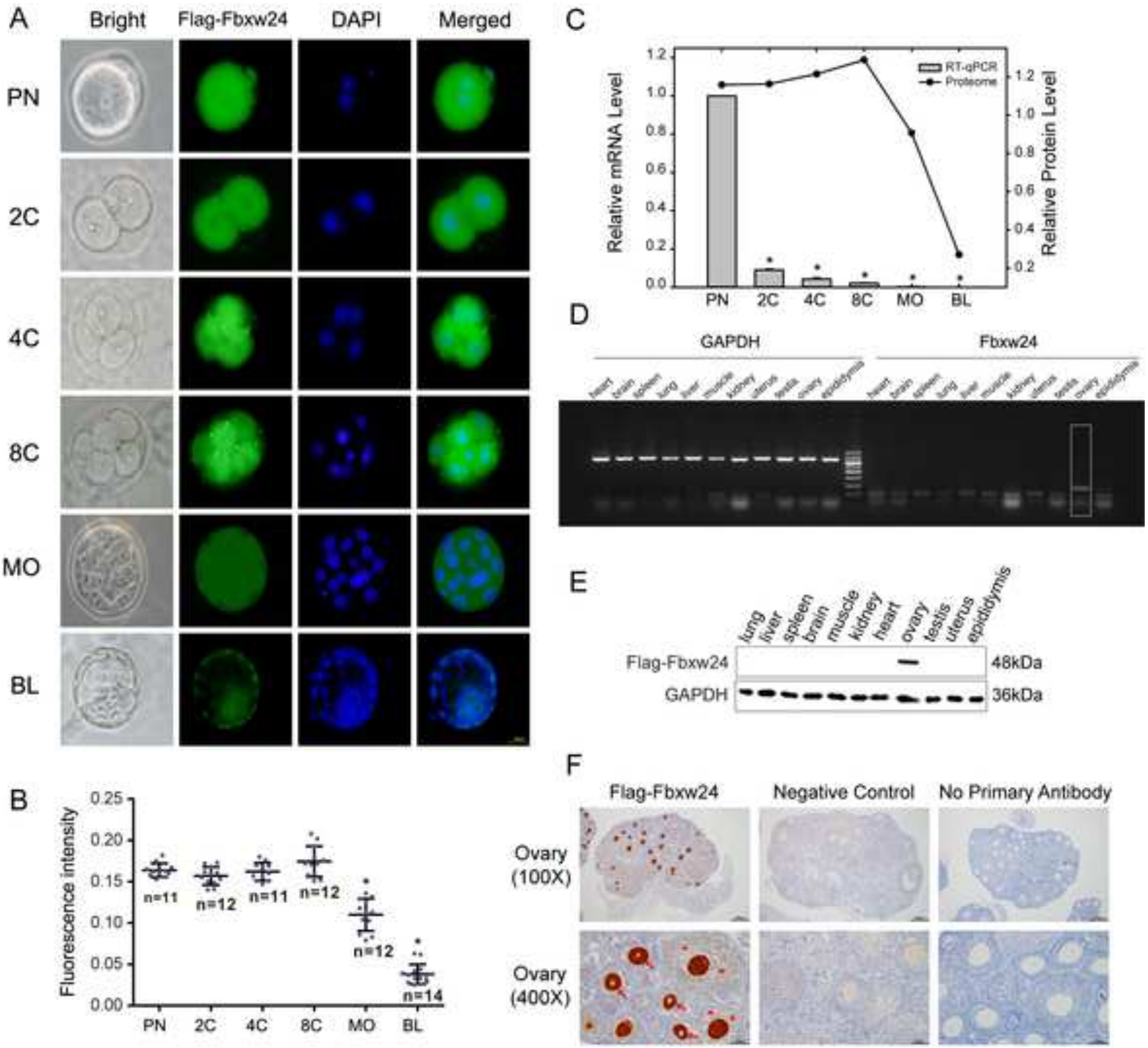

Figure.7

Click here to access/download;Figure;Figure.7.tif

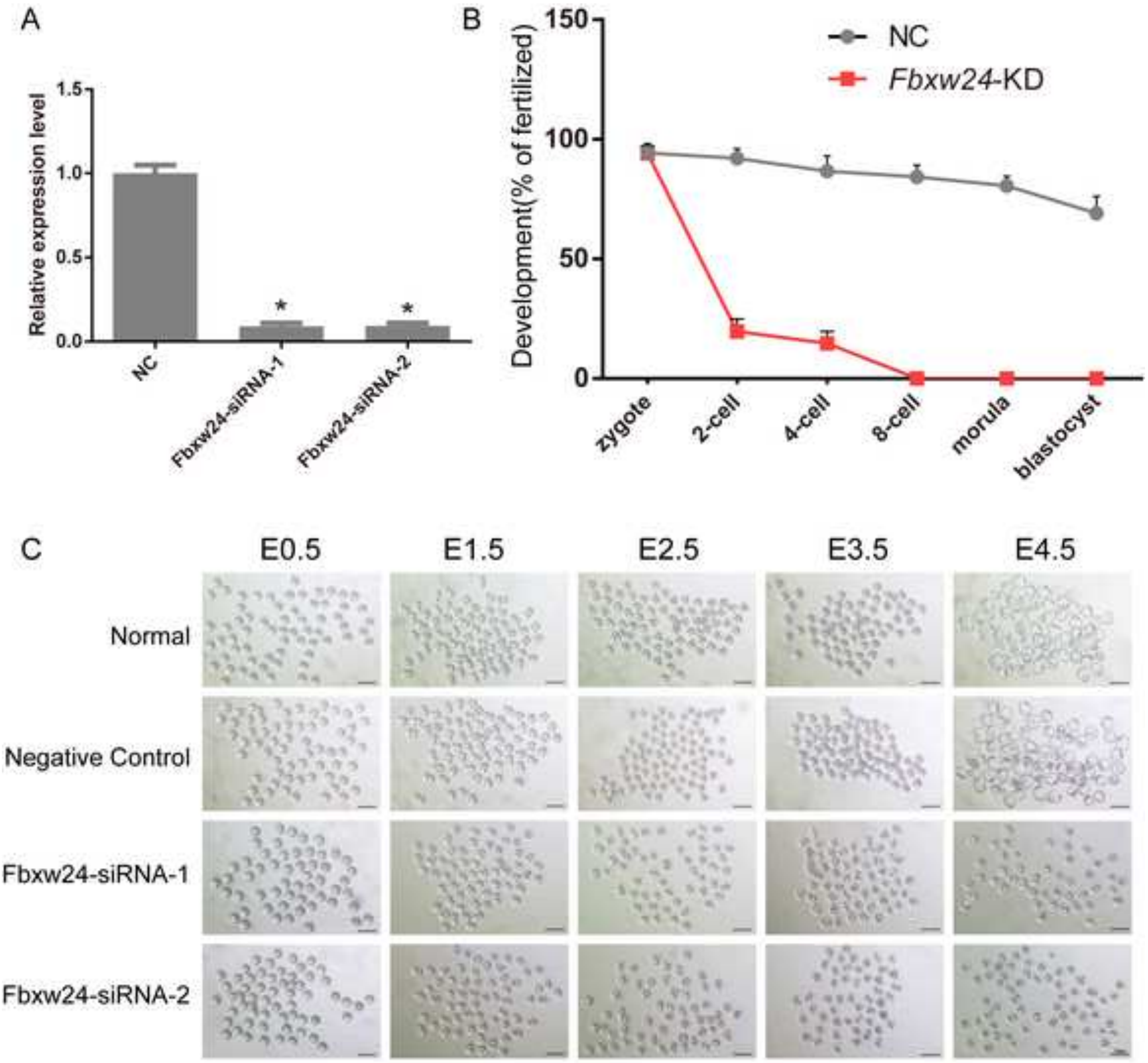

Figure.8

Click here to access/download;Figure;Figure.8.tif

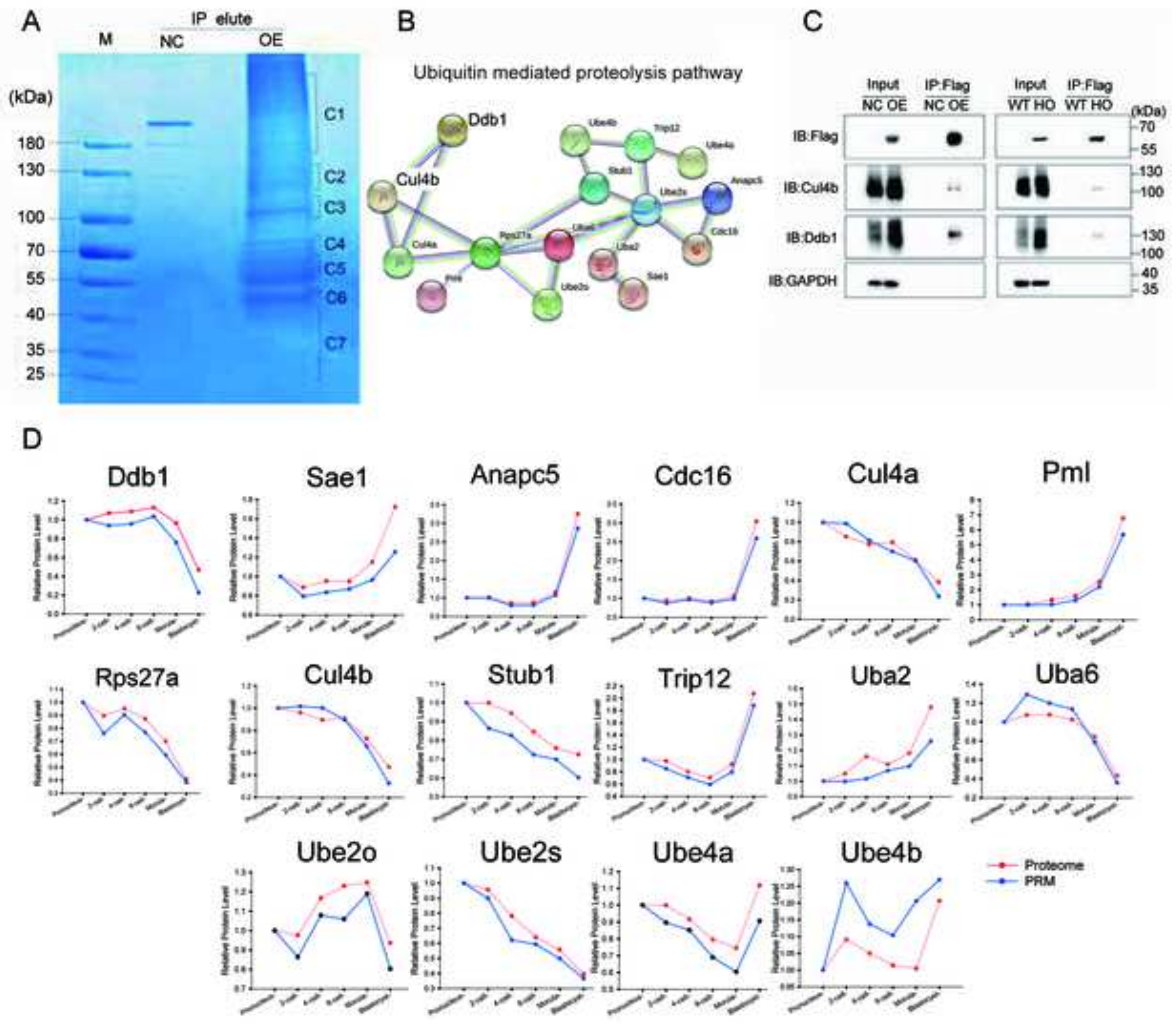

Cover letter

Click here to access/download
Supplementary Material
Cover letter.docx

Figure.S1

Click here to access/download
Supplementary Material
Figure.S1.tif

Figure.S2

Click here to access/download
Supplementary Material
Figure.S2.tif

Figure.S3

Click here to access/download
Supplementary Material
Figure.S3.tif

Figure.S4

Click here to access/download
Supplementary Material
Figure.S4.tif

Click here to access/download
Supplementary Material
Table 1_for Reviewer 2.xlsx

Table S1

Click here to access/download
Supplementary Material
Table S1.xlsx

Click here to access/download
Supplementary Material
Table S2.xlsx

Table S3

Click here to access/download

Supplementary Material

Table S3.xlsx

Table S4

Click here to access/download
Supplementary Material
Table S4.xlsx

Table S5

Click here to access/download
Supplementary Material
Table S5.xlsx

Table S6

Click here to access/download
Supplementary Material
Table S6.xlsx

Table S7

Click here to access/download
Supplementary Material
Table S7.xlsx

Table S8

Click here to access/download
Supplementary Material
Table S8.xlsx

Table S9

Click here to access/download
Supplementary Material
Table S9.xlsx

Table S10

Click here to access/download
Supplementary Material
Table S10.xlsx

Click here to access/download
Supplementary Material
Table S11.xlsx
