# Supplementary material for: Comparative maternal protein profiling of mouse biparental and uniparental embryos
Source: Gigascience. 2022 Sep 3;11:giac084. doi: 10.1093/gigascience/giac084 (PMC9440387; doi:10.1093/gigascience/giac084)
Supplement: giac084_GIGA-D-22-00094_Revision_1 [file giac084_giga-d-22-00094_revision_1.pdf]

GigaScience

# Comparative maternal protein profiling of mouse biparental and uniparental embryos

--Manuscript Draft--

| | |
|---|---|
| **Manuscript Number:** | GIGA-D-22-00094R1 |
| **Full Title:** | Comparative maternal protein profiling of mouse biparental and uniparental embryos |
| **Article Type:** | Research |
| **Funding Information:** | China Postdoctoral Science Foundation (2019M653810XB) — Dr Fumei Chen<br>Guangxi Natural Science Foundation Program (2019JJB140131) — Dr Fumei Chen<br>Guangxi First-class Discipline Project for Basic medicine Sciences (GXFCDP-BMS-2018) — Dr Fumei Chen |
| **Abstract:** | Background: During early embryonic development, maternal proteins act as important roles. However, our cognition of maternal proteins is still very limited. The integrated analysis of mouse uniparental (parthenogenetic) and biparental (fertilized) embryos in protein level may provide us more information for maternal proteins. Although proteome of mouse fertilized embryos has been reported, the protein expression landscape of mouse parthenogenesis remains unexplored.<br>Results: Using label-free quantitative mass spectrometry (MS) analysis, we firstly reported the maternal proteome of mouse parthenogenetic embryos (pronucleus, 2-cell, 4-cell, 8-cell, morula and blastocyst) and showed its dynamic changes. Then the combined comparison of proteins profile for parthenogenesis and fertilized embryos shown the different fates of maternal proteins. We enriched a group of maternal proteins that are strongly correlated with the subcortical maternal complex (SCMC), and found some maternal proteins may escape from the fate of degradation in mouse parthenogenesis. Moreover, we identified a new maternal factor (Fbxw24) and showed its importance in early embryonic development. Especially, we found that Fbxw24 interact with Ddb1-Cul4b and may regulate maternal degradation in mouse.<br>Conclusions: Our study provides a valuable resource for further mechanistic studies of maternal proteins, and suggests a new maternal factor regulating pre-implantation embryo development. |
| **Corresponding Author:** | Fumei Chen<br>Guangxi Medical University<br>Nanning, CHINA |
| **Corresponding Author Secondary Information:** | |
| **Corresponding Author's Institution:** | Guangxi Medical University |
| **Corresponding Author's Secondary Institution:** | |
| **First Author:** | Fumei Chen |
| **First Author Secondary Information:** | |
| **Order of Authors:** | Fumei Chen<br>Buguo Ma<br>Yongda Lin<br>Xin Luo<br>Tao Xu<br>Yuan Zhang<br>Fang Chen<br>Yanfei Li |

Powered by Editorial Manager® and ProduXion Manager® from Aries Systems Corporation

| | |
|---|---|
| | Yaoyao Zhang |
| | Bin Luo |
| | Qingmei Zhang |
| | Xiaoxun Xie |
| **Order of Authors Secondary Information:** | |

**Response to Reviewers:**

Dear Editor:

Thank you for your letter and all comments concerning our manuscript "Comparative maternal protein profiling of mouse biparental and uniparental embryos" (GIGA-D-22-00094), and they are highly valuable and helpful for improving our paper. We have studied these comments carefully and have made corrections which we hope meet with approval. For the detailed response, please see below.

Responds to the Reviewer #1:

In the revised version of the article, the authors largely corrected the article based on my previous comments. I am satisfied with the revised version.
Only a few minor errors remained to be corrected, which are given below.

1). Please write the phrase "et al." in an italic format in the following sites, Gao et al., Israel et al., etc.
Response: Thank you for your comments. The corresponding corrections have been made in the revised manuscript: Line 66, Line 71, Line 75, Line 565, Line 588, Line 589.

2). Please use full stop instead of comma in the following numbers 5,217; 1,709 etc.
Response: Thank you for your comments. The numbers, including 5,217; 1,709 etc , represents the amount of protein, not a decimal point, and we think this typing of number is common in many published documents.

3). The phrase "in vivo" should be written in an italic format.
Response: Thank you for your comments.The corresponding corrections have been made in the revised manuscript: Line 477, Line 578.

4). Please correct the "mice ovaries" into mouse ovaries.
Response: Thank you for your comments. The corresponding corrections have been made in the revised manuscript: Line 478, Line 817, Line 923.

5). Other typing errors should be corrected throughout the article.
Response: Thank you for your comments. We have checked and made corrections throughout the manuscript, and all details are presented in the revised manuscript.

6). Figures 1, 4, and 5 qualities should be increased to provide more easily reading the typing and images.
Response: Thank you for your comments. The resolution of Figures 1, 4, and 5 has been increased from 300dpi to 600dpi, and we think it may increased the qualities of these figures.

Responds to the Reviewer #2:

1). if the Authors say that Fbxw24 is a maternal factor, then they need to show that Fbxw24 is present in metaphase II oocytes prior to parthenogenetic activation or fertilization.
Response: Thank you for your comments. I). Fbxw24 protein was detected by immunofluorescence assay using the oocytes of germinal vesicle(GV) stage and metaphase II (MII) stage that obtained from C57BL/6J-Fbxw24em(Linker-3xFlag) mice. The results show that Fbxw24 are detected in both GV and MII oocytes in protein level (as shown in the figure below). This result is supplemented in the revised manuscript as Figure S5 and its corresponding descriptions were also added in revised manuscript: Line 436-439, Line 642-643 and Line 1152-1155.

Powered by Editorial Manager® and ProduXion Manager® from Aries Systems Corporation

II). The immunohistochemistry results also indicate that the Fbxw24 protein was located in oocytes (red arrows) and granulosa cells (red arrowheads) at different follicular stages (Fig. 6F) (Line 455-457 in the revised manuscript)

III). The Fbxw24 also detected in the protein database of MII oocytes reported by Israel et al., (PMID: 31527703, PMID:31638890).

2). If the proteomic dataset of this study does not include Fbxw24 (Table 1 and Supplementary Table 1), then this is somewhat a logical glitch
Response: Thank you for your comments. I). Although Fbxw24 was not detected in the proteome of parthenogenetic embryos, the selection of fbxw24 is based on the comparison results of the two embryonic proteome (biparental/fertilized and uniparental/parthenogenetic embryos). During the analysis, a family of F-box/WD40 repeat-containing proteins (Fbxws) attracted our attention, and the selection of Fbxw24 for further analysis based on the following detailed reasons:
①We found that only Fbxw11 and Fbxw15 were detected in uniparental embryos(PA group, identified in this study ), while Fbxw8, Fbxw11, Fbxw13, Fbxw15, Fbxw16, Fbxw18, Fbxw19, Fbxw20, Fbxw21, Fbxw22, Fbxw24, Fbxw26, Fbxw28 were detected in biparental embryos (ZY group, identified by Gao and Israel) (Table 1). Obviously, the quantity of Fbxws in biparental embryos is much more than uniparental embryos.
②Furthermore, in uniparental embryos, Fbxw11 was not correlated with the SCMC components, but in biparental embryos it had a strong negative correlation (r≤-0.70, marked in green, Table 1) with the SCMC components; Also the expression correlation between Fbxw15 and the SCMC components is quite opposite in two embryos: Fbxw15 has strong negative correlations (r≤-0.70, marked in green, Table 1) with the SCMC components in uniparental embryos, but strong positive correlations (r≥0.70, marked in red, Table 1) with the SCMC components in biparental embryos.
③Finally, Fbxw24 was selected for further analysis, because its correlation relationship with other Fbxws is similar to that of the SCMC components in biparental embryos (Table 1), which reveal it probably be of importance in normal embryonic development from zygote.
④To date, the role of Fbxw24 during early embryonic development remain unstudied. (The corresponding descriptions were presented in revised manuscript, Line 415-434).

II). The selection of fbxw24 was based on the comparison results of the two embryonic proteome, and the parthenogenetic embryo proteome reported in this study is a critical reference. The finding that Fbxw24 may play a key role as a maternal factor in the development of preimplantation embryos is a successful case of using parthenogenetic embryos as a reference. It also shows that it may be valuable for the identification of key maternal factors by comparing the maternal protein files of parthenogenetic embryos and fertilized embryos.

3). How can it be claimed that it is a maternal factor, if it is not detected in oocytes? I understand that probably there is no valid antibody to reveal native Fbxw24 by immunofluorescence or by western blotting, however, the claim that Fbxw24 is a maternal factor demands evidence that Fbxw24 is present in metaphase II oocytes as protein. This is my major point. Maybe the Authors can 'borrow' the information from other studies, and/or expand the use of their FLAG system.
Response: Thank you for your comments. I). Fbxw24 protein was detected by immunofluorescence assay using the oocytes of germinal vesicle(GV) stage and metaphase II (MII) stage that obtained from C57BL/6J-Fbxw24em(Linker-3xFlag) mice. The results show that Fbxw24 are detected in both GV and MII oocytes in protein level(as shown in the figure below). This result is supplemented in the revised manuscript as Figure S5 and its corresponding descriptions were also added in revised manuscript: Line 436-439, Line 642-643 and Line 1152-1155.

II). The immunohistochemistry results also indicate that the Fbxw24 protein was located in oocytes (red arrows) and granulosa cells (red arrowheads) at different follicular stages (Fig. 6F) (Line 455-457 in the revised manuscript)

III). The Fbxw24 also detected in the protein database of MII oocytes reported by Israel et al., (PMID: 31527703, PMID:31638890).

Powered by Editorial Manager® and ProduXion Manager® from Aries Systems Corporation

4). Line 128: I understand what the Authors are saying; 'commonly' may be interpreted as meaning 'in common' that is, shared; I guess the Authors mean the union of the 1900, 1944 and 1960, not the intersection (in common). I suggest to check that the sentence is conveying the Authors' intended meaning.
Response: Thank you for your comments. As you understand, the 2,048 proteins mean the union of the 1900, 1944 and 1960 in deed. We have made the corresponding corrections: In three repetitions, 1,900, 1,944 and 1,960 proteins were detected respectively, and the number of their union was 2,048, of which 1,902 proteins were quantified (Line127-129 in the revised manuscript).

5). Line 408: "The expression and functional analysis of a new maternal factor". It appears that Fbxw24 was not detected in the parthenogenetic proteome. If so, then to name it 'maternal factor' does not seem justified. The Authors need to show that Fbxw24 is present as protein in metaphase II oocytes. If there is no suitable commercial antibody available against Fbxw24, would it then be possible to use the Flag system of Figure 6A to show expression in metaphase II oocytes? As alternative, would it be possible to infer the presence of Fbxw24 in MII oocytes from other studies?
Response: Thank you for your comments. I). Fbxw24 protein was detected by immunofluorescence assay using the oocytes of germinal vesicle(GV) stage and metaphase II (MII) stage that obtained from C57BL/6J-Fbxw24em(Linker-3xFlag) mice. The results show that Fbxw24 are detected in both GV and MII oocytes in protein level(as shown in the figure below). This result is supplemented in the revised manuscript as Figure S5 and its corresponding descriptions were also added in revised manuscript: Line 436-439, Line 642-643 and Line 1152-1155.

II). The immunohistochemistry results also indicate that the Fbxw24 protein was located in oocytes (red arrows) and granulosa cells (red arrowheads) at different follicular stages (Fig. 6F) (Line 455-457 in the revised manuscript)

III). The Fbxw24 also detected in the protein database of MII oocytes reported by Israel et al., (PMID: 31527703, PMID:31638890).

6). And would it be possible to add parthenogenetic embryos to Figure 6A?
Response: Thank you for your comments. In this study, during the comparison of the two embryonic proteome, the role of parthenogenetic embryos is as a reference and it is valuable for the identification of new key maternal factors. But parthenogenesis is not the common reproductive mode of mammals, and we think that the further functional research on a detailed maternal factor (such as Fbxw24) should mainly focus on fertilized embryos rather than parthenogenetic embryos.

7).Line 546: typo, preimplatation ('n' is missing)
Response: Many thanks for your careful check, and we have corrected it (Line 548 in the revised manuscript).

8). Line 634: 'mice were free to eat'; I suggest to say 'mice were fed ad libitum'.
Response: Thank you for your suggestions. We have made the corresponding corrections (Line 635 in the revised manuscript).

9). Line 444: I suggest to say 'transcript level' instead of 'transcriptional activity'
Response: Thank you for your suggestions. We have made the corresponding corrections (Line 446 in the revised manuscript).

Thank you again for your many professional suggestions, and they hold a very highly value for guiding our future studies.

We tried our best to improve the manuscript and made corresponding corrections in the revised manuscript. And we appreciate for Editors/Reviewers' warm work earnestly, and hope that the correction will meet with approval.

Once again, thank you very much for your comments and suggestions.

Powered by Editorial Manager® and ProduXion Manager® from Aries Systems Corporation

| | |
|---|---|
| | Best regards,<br><br>Dr. Fumei Chen<br>Department of Histology and Embryology, School of Pre-Clinical Medicine, Guangxi Medical University, Nanning, Guangxi, P. R. China<br>E-Mail: gxchenfumei@163.com |
| **Additional Information:** | |
| **Question** | **Response** |
| Are you submitting this manuscript to a special series or article collection? | No |
| **Experimental design and statistics**<br><br>Full details of the experimental design and statistical methods used should be given in the Methods section, as detailed in our Minimum Standards Reporting Checklist. Information essential to interpreting the data presented should be made available in the figure legends.<br><br>Have you included all the information requested in your manuscript? | Yes |
| **Resources**<br><br>A description of all resources used, including antibodies, cell lines, animals and software tools, with enough information to allow them to be uniquely identified, should be included in the Methods section. Authors are strongly encouraged to cite Research Resource Identifiers (RRIDs) for antibodies, model organisms and tools, where possible.<br><br>Have you included the information requested as detailed in our Minimum Standards Reporting Checklist? | Yes |
| **Availability of data and materials**<br><br>All datasets and code on which the conclusions of the paper rely must be either included in your submission or deposited in publicly available repositories (where available and ethically | Yes |

| | |
|---|---|
| appropriate), referencing such data using a unique identifier in the references and in the “Availability of Data and Materials” section of your manuscript.<br><br>Have you have met the above requirement as detailed in our Minimum Standards Reporting Checklist? | |

*Powered by Editorial Manager® and ProduXion Manager® from Aries Systems Corporation*

Manuscript
Click here to access/download;Manuscript;Manuscript_the_updated_version

# Comparative maternal protein profiling of mouse biparental and uniparental embryos

Fumei Chen[1], Buguo Ma[1,2], Yongda Lin[1,2], Xin Luo[1], Tao Xu[1], Yuan Zhang[1], Fang Chen[1,2], Yanfei Li[1,2], Yaoyao Zhang[1,2], Bin Luo[1,2,], Qingmei Zhang[1,2,*], Xiaoxun Xie[1,2,*]

[1]Department of Histology and Embryology, School of Pre-Clinical Medicine, Guangxi Medical University, Nanning, Guangxi, P. R. China

[2]Central Laboratory, School of Pre-Clinical Medicine, Guangxi Medical University, Nanning, Guangxi, P. R. China

E-mail addresses for all authors:

Fumei Chen: gxchenfumei@163.com, ORCID: 0000-0002-0955-1277

Buguo Ma: mabuguo@gxmu.edu.cn

Yongda Lin: linyongda@hotmail.com

Xin Luo: 295268654@qq.com

Tao Xu: 1031895151@qq.com

Yuan Zhang: 13033466195@163.com

Fang Chen: 756007230@qq.com

Yanfei Li: 1195220977@qq.com

Yaoyao Zhang: 1575762156@qq.com

Bin Luo: glbinbin2002@yahoo.com

*Correspondence:

*Qingmei Zhang: zhangqingmei2017@outlook.com

*Xiaoxun Xie: xiaoxunxie@hotmail.com, ORCID: 0000-0001-6683-5180

## Abstract

**Background**: Maternal proteins have important roles during early embryonic development. However, our understanding of maternal proteins is still very limited. The integrated analysis of mouse uniparental (parthenogenetic) and biparental (fertilised) embryos at the protein level creates a protein expression landscape that can be used to explore pre-implantation mouse development.

**Results**: Using label-free quantitative mass spectrometry (MS) analysis, we report on the maternal proteome of mouse parthenogenetic embryos at pronucleus, 2-cell, 4-cell, 8-cell, morula and blastocyst stages, and highlight dynamic changes in protein expression. In addition, comparison of proteomic profiles of parthenogenotes and fertilised embryos highlight the different fates of maternal proteins. Enrichment analysis uncovered a set of maternal proteins that are strongly correlated with the subcortical maternal complex (SCMC), and we report that in parthenogenotes some of these maternal proteins escape the fate of protein degradation. Moreover, we identified a new maternal factor - Fbxw24 - and highlight its importance in early embryonic development. We report that Fbxw24 interacts with Ddb1-Cul4b, and may regulate maternal protein degradation in mouse.

**Conclusions**: Our study provides an invaluable resource for mechanistic analaysis of maternal proteins, and highlights the role of the novel maternal factor Fbw24 in regulating maternal protein degradation during pre-implantation embryo development.

**Keywords**: Maternal protein; Parthenogenesis; Early embryo; Proteome; Mouse

## Background

During oogenesis, proteins from the oocyte genome (maternal genome) with important roles in fertilisation and early embryonic development, including the protein degradation, activation of the embryonic genome, epigenetic modifications and cell signal transduction are largely accumulated [1]. The depletion or abnormal expression of maternal proteins not only affects embryo development, it can even lead to embryonic death [2-8]. Therefore, analysing maternal proteins can deepen our understanding of the regulatory mechanisms underlying embryonic development.

Despite the focus of many studies of mammalian preimplantation development on transcriptomic data [9, 10], embryonic protein databases provide invaluable information for the study of maternal proteins. Protein abundance, which is closer to the phenotype have more predictive value than mRNAs. In some cases there is even anti-correlation, in which the mRNA is rapidly degraded after fertilisation, whereas proteins persist throughout the blastocyst stage [11].

Quantitative proteomics is an effective strategy to construct protein databases for gametes or embryos. There are mass spectrometry (MS)-derived databases of early embryos, including bovine [12], zebrafish [13] and Xenopus [14, 15]. In addition, the mouse oocyte and embryo proteomes have been previously described [11, 16-18]. Wang *et al*. collected 7,000 mouse oocytes at different developmental stages, including the germinal vesicle stage, the metaphase II (MII) stage and fertilised oocytes (zygotes)

and successfully identified 2,781 proteins in germinal vesicle oocytes, 2,973 proteins in MII oocytes and 2,082 proteins in zygotes through semiquantitative MS analysis [16]. Moreover, Gao *et al*. identified nearly 5,000 proteins across six developmental stages (from the zygote to the blastocyst) by tandem mass tag (TMT) labelling, which they performed in duplicate. In total, 4,608 and 4,590 proteins were quantified in each experiment, and the data from the two experiments showed 3,767 common proteins [17]. Israel *et al*. collected and processed a total of ~12,600 oocytes or embryos, with three biological replicates of ~600 oocytes/embryos per developmental stage: unfertilised oocytes, fertilised oocytes with pronuclei and preimplantation embryos at the 2-, 4-, 8-cell, advanced morula and blastocyst stages. The detected proteome comprised 6,550 proteins identified by stable isotope labelling with amino acids in cell culture (stable isotope labelling with amino acids in cell culture, SILAC). Among the detected proteins, 5,217 were detected in ≥2 replicates of ≥1 developmental stages, and 1,709 proteins were detected in both replicates at all developmental stages [11].

Mouse parthenogenesis is a well-developed model to explore embryonic development [19]. Under natural conditions, an oocyte can be activated without intervention of the male counterpart. This form of reproduction, known as parthenogenesis, occurs spontaneously in various lower organisms [20, 21]. In mammals, oocytes can be activated using different methods, including high or low temperature, electrical or chemical treatment [22]. Oocyte activation rates depend on various factors, including species, age of the female and culture conditions [22]. Moreover, inducing artificial

oocyte activation is vital for somatic cell nuclear transfer research [23]. Some transcriptomic studies have expanded our understanding of the genetic programmes underlying mammal parthenogenesis [24-30]; however, the protein database of parthenogenetic embryos has not been reported yet. A comparative analysis of these two types of embryos (biparental and uniparental embryos) may help understand maternal proteins, as parthenogenetic embryos only have maternal information, while fertilised embryos have the information of the biparental genome.

In this study, we prepared mouse uniparental embryos artificially activated and cultured in vitro for proteomic analysis to identify dynamic changes in maternal proteins during early embryonic development (from the pronucleus to the blastocyst stage). A comparative analysis of protein expression was performed in mature oocytes and fertilised and parthenogenetic embryos. We also performed enrichment analysis of maternal proteins strongly correlated with subcortical maternal complex (SCMC) components and found a group of maternal proteins that may escape degradation in mouse parthenogenesis. Additionally, identification and functional analyses were performed for a new key maternal factor, Fbxw24. Our uniparental embryo proteomic database is the first complete mammalian parthenogenetic embryo proteome characterised to date. This proteome dataset enables a more direct investigation of mammalian developmental processes regulated by the maternal genome at the protein level, and complements the knowledge on the proteomics underlying mammalian embryonic development.

## Results

### Definition and dynamics of maternal protein expression in mouse uniparental embryos

The protein expression profiles of six embryonic stages of mouse parthenogenesis, i.e., pronucleus (PA), 2-cell (PA2), 4-cell (PA4), 8-cell (PA8), morula and blastocyst, were detected by label-free quantitative MS. The developmental rate of parthenogenetic embryos and their morphology are shown in Supplementary Fig. S1. For each stage, 6,000 embryos were used and the experiment was performed in triplicate. In the three repetitions, 1,900, 1,944 and 1,960 proteins were detected, respectively, and the total number was 2,048, of which 1,902 proteins were quantified (quantified proteins could be detected in ≥1 embryonic stage and >2 biological replicates; Fig. 1A and Supplementary Table S1). All identified proteins and peptides are shown in Supplementary Table S1.

Among the quantified proteins, 1,298 proteins were detected in all six successive developmental stages (Fig. 1B); however, few were detected in only one stage. For example, 57 proteins were only detected in the PA stage (after oocyte activation; Fig. 1B), including CCCTC binding factor, fcf1 rRNA processing protein, the general transcription factor IIF and polypeptide 1, involved in various processes including RNA processing, gene expression and nitrogen compound metabolic processes. Detailed

information and the annotations of intersecting groups are shown in Supplementary Table S2. The hierarchical cluster analysis of quantified proteins closely clustered PA-4 and PA-8 embryos along with PA-2 and PA embryos. However, the morula and blastocyst were separated (Fig. 1C). These results are consistent with those from principal component analysis, another clustering method (Fig. 1D). These results reveal that the blastocyst stage differed from other stages during development, consistent with the corresponding analysis of fertilised embryos [11, 17]. In mouse biparental and uniparental embryos, a major dynamic change in proteins occurs at the blastocyst stage.

Next, a Fuzzy c-means analysis was performed; 10 distinct expression pattern clusters were identified (Fig. 1E and Supplementary Table S3). Five known components of the SCMC, a protein structure essential for preimplantation development [3, 31], including Ooep, Tle6, Padi6 (cluster 8 in Fig. 1E), Nlrp5 (cluster 1 in Fig. 1E) and Zbed3 (cluster 4 in Fig. 1E) were detected in this study. This indicated that these maternal proteins are essential for early embryonic development in both mouse biparental and uniparental embryos. In addition, some N6-methyladenosine (m6A) readers, including Eif3a, Eif3b, Elavl1, Hnrnpa2b1, Hnrnpc, Igf2bp1 and Srsf2 were detected (cluster 5 and cluster 6 in Fig. 1E) [32].

A cluster of highly abundant proteins was detected at PA2 (cluster 9 in Fig. 1E), including zinc finger protein 57 (Zfp57) and ring finger protein 2 (Rnf2). Lack of Zfp57 in oocytes results in failed maternal methylation imprinting at the Snrpn imprinted

region; Zfp57 is also required for the post-fertilisation maintenance of maternal and paternal methylation imprints at multiple imprinted domains [33]. Meanwhile, Rnf2 is a component of Polycomb-repressive complex 1 that functions as a redundant transcriptional factor during oogenesis and essential for proper zygotic genome activation [34].

A cluster of highly abundant proteins was detected at the PA4 stage (cluster 4 in Fig. 1E), including nucleophosmin/nucleoplasmin 2 (Npm2) and zinc finger and BED type containing 3 (Zbed3). Mouse Npm2 accumulates in oocyte nuclei and persists in preimplantation embryos. Moreover, Npm2 knockout females have fertility defects owing to failed preimplantation embryonic development [1].

A cluster of highly abundant proteins was detected at the PA8 stage (cluster 7 in Fig. 1E), including developmental pluripotency-associated 3 (Dppa3) and mitochondrial transcription factor A (Tfam). Dppa3, also known as PGC7/Stella, protects the maternal genome from demethylation only after nucleus localisation and is indispensable for the maintenance of methylation required for epigenetic reprogramming after fertilisation [35]. In zebrafish embryos, knocking down Tfam, a regulator of mitochondrial DNA (mtDNA) replication, results in mtDNA copy number reduction and deficient oxidative phosphorylation [36].

A cluster of highly abundant proteins was detected at the morula stage (cluster 10 in

Fig. 1E), including 2'-5' oligoadenylate synthetase 1D (Oas1d) and lysine (K)-specific demethylase 2B (Kdm2b). Mutant mice lacking Oas1d display lower fertility due to ovarian follicle developmental defects, decreased ovulation efficiency and fertilisation arrest at the one-cell stage [37]. Kdm2b, also known as Fbxl10 (F-box and leucine-rich repeat protein 10), is a JmjC domain-containing histone demethylase that contributes to embryonic neural development in mice by regulating cell proliferation and cell death [38].

Other important proteins in early embryonic development were also quantified in mouse uniparental embryos, including Filia [39] (cluster 1 in Fig. 1E), Dnmt1 [40] (cluster 2 in Fig. 1E), Eed [41] and Ezh2 [42] (cluster 5 in Fig. 1E), E-cadherin [43] and Brg1 [44] (cluster 6 in Fig. 1E) and Uhrf1 [45] (cluster 8 in Fig. 1E).

Gene ontology (GO) analysis of proteins from each cluster (Supplementary Fig. S2A) revealed that these proteins performed many functions, including protein folding, cellular localisation, cellular component biogenesis and cellular metabolic processes. These processes provide basic energy and materials for embryonic growth and development. The interaction networks of GO terms from four categories, including ‘translation’, ‘peptide metabolic process’, ‘nucleic acid metabolic process’ and ‘cellular metabolic process’, with each network providing an elaborate view of the proteins participating in these biological processes are shown in Supplementary Fig. S2B-E.

## Comparison of maternal protein expression in mouse biparental and uniparental embryos

Maternal proteins have different fates after oocyte activation, including degradation or persistence. In this study, we compared the three proteome databases constructed using mature oocytes (MII group), fertilised (biparental embryos, ZY group/ZY embryo) and parthenogenetic embryos (uniparental embryos, PA group/PA embryo, without paternal genome). To eliminate methodological differences and ensure the reliability of these results, we used the intersection of two reported MII oocyte protein databases [16, 18] as MII group (2,209 proteins, Wang and Israel, Fig. 2A) and the intersection of two reported fertilised embryo proteome databases [17, 18] as ZY group (3,218 proteins, Gao and Israel, Fig. 2A). Detailed results of the Venn diagram in Fig. 2A are shown in Supplementary Table S4.

A total of 1,029 proteins derived from mature oocytes were continuously detected during preimplantation of uniparental and biparental embryos (Fig. 2A), indicating that the expression of these proteins may be independent from paternal genome regulation. We found similar expression changes of five SCMCs (Ooep, Nlrp5, Tle6, Zbed3 and Padi6) in the three embryo proteome databases (this study, Gao's and Israel's), consistent with our verification in fertilised embryos using the parallel reaction monitoring (PRM) assay (Fig. 2B), suggesting that the five components may play similar biological functions in both biparental and uniparental mouse embryos.

Moreover, hierarchical clustering analysis of 1,029 proteins in uniparental embryos, showed major protein expression changes in the morula stage (Fig. 2C). Correspondingly, in biparental embryos, the major expression change was found at the blastocyst stage (Fig. 2D and 2E).

After artificial activation, 1,168 proteins in mature oocytes were detected in uniparental embryos (Fig. 3A). Correspondingly, 1,806 proteins in mature oocytes were detected in biparental embryos (Fig. 3B). Compared to biparental embryos, 613 proteins were only detected in uniparental embryos in this study (Fig. 3C). Furthermore, to reveal the dynamics of protein expression during development, Fuzzy c-means clustering was performed (Fig. 3A-C and Supplementary Table S5). We found two similar protein expression patterns in uniparental (PA group) and biparental embryos (ZY group). First, some protein levels peak at the blastocyst stage (cluster 6 in Fig. 3A; cluster 2-Gao and cluster 6-Israel in Fig. 3B), which accounts for their highest protein number compared to other clusters, followed by a small change at the four early embryonic stages (PN, 2-cell, 4-cell and 8-cell), and a great change after the 8-cell stage (compaction initiation, Fig. 3D), including proteins involved in mitotic cell cycle regulation (Tpr, Hnrnpu, Hmgb1, Mta3, Rpl17, Eif4g1 and Lmnb1) and cell-cell junction organisation (Itgb1, Cttn, Cdh1, Rcc2, Ctnna1, Actn4 and Coro1c). Intriguingly, a cluster of maternal proteins detected only in uniparental embryos (PA group) in this study also followed this trend (cluster 1 in Fig. 3C), suggesting that embryonic compaction after the 8-cell stage is accompanied by protein expression changes both in biparental and uniparental

embryos.

Second, the high abundance of some proteins detected in the pronucleus stage decreased during cleavage (maternal to zygotic transition, Fig. 3D); their abundance was lowest at the morula or blastocyst stage (maternal expression pattern, cluster 1 in Fig. 3A, cluster 1-Gao and cluster 5-Israel in Fig. 3B), including proteins involved in egg activation (Astl, Plat) and the post-transcriptional regulation of gene expression (Fxr1, Ddx6, Eif4enif1, Fxr2, Ybx2, Igf2bp2, Lsm14b and Lsm14a). A similar pattern was detected in maternal proteins regulated only by the maternal genome (cluster 6 in Fig. 3C). This may underlie the successful development beyond the 2-cell stage in uniparental embryos.

**Enrichment of maternal proteins strongly correlated with SCMC components during preimplantation**

The SCMC is a macromolecular complex encoded by maternal genes, mainly found in oocytes and early embryos and functionally conserved in mammals [1]. This complex is directly or indirectly involved in early embryonic development, in processes such as cell division, cytoskeleton and organelle rearrangement, maternal RNA regulation and zygotic genome activation [1, 31]. Currently, unveiling the molecular function of the SCMC would help understand the maternal regulatory network and oocyte biology in mammals [46, 47]. In addition, the SCMC can be used as a valuable reference for the

identification of important mammalian maternal factors [3]. Expression correlation analysis also aids in analysing current 'omic' data, which can screen out potential key proteins or genes. The establishment of embryo proteomes during embryonic development provides data support for such analyses.

We detected five components of the SCMC (Ooep, Nlrp5, Tle6, Zbed3 and Padi6, designated as SCMCs in this study) which similar patterns in mouse biparental and uniparental embryos (Fig. 2B). In this study, SCMCs were used as 'target proteins' to filter candidate maternal proteins based on the correlation of their expression with SCMCs. The relationship between SCMCs and other quantified proteins in the PA (uniparental embryos) and ZY (biparental embryos, including two protein databases identified by Gao and Israel) groups was analysed by Pearson's correlation coefficients (Supplementary Table S6). An absolute correlation coefficient $\geq 0.70$ ($|r| \geq 0.70$ and $p \leq 0.05$) denotes a strong expression correlation [48]. Based on this criterion, there were 429 candidate proteins ($|r| \geq 0.70$ and $p \leq 0.05$) enriched in mouse biparental and uniparental embryos (Fig. 4A and Supplementary Table S7). Of these proteins, 113 were strongly positively correlated ($r \geq 0.70$ and $p \leq 0.05$, with the lowest protein level at the blastocyst stage) and 304 were strongly negatively correlated ($r \leq -0.70$ and $p \leq 0.05$, with the highest protein level at the blastocyst stage; Fig. 4A, Fig. 4E and Supplementary Table S7).

Kyoto Encyclopedia of Genes and Genomes (KEGG) pathway analysis was conducted

on 429 candidate proteins (Fig. 4B). These proteins were found to be widely involved in the regulation of numerous biological processes during embryonic development, including metabolism, genetic information processing, environmental information processing and cellular processes. The protein-protein interaction (PPI) network of candidate proteins involved in 'Translation' was obtained using the STRING database [81], showing a complicated PPI network (Fig. 4C). To validate the expression trends of these candidate proteins, eight candidate proteins were randomly selected (highlighted with a red box in Fig. 4C) for the PRM assay in biparental embryos. The results revealed the same protein expression pattern as in the proteome analysis (Fig. 4D).

The number of strongly negatively correlated proteins (304 proteins, Fig. 4A and Supplementary Table S7) was about three times that of strongly positively correlated proteins (113 proteins, Fig. 4A and Supplementary Table S7). These strongly negatively correlated proteins have complex interaction networks (Supplementary Fig. S3A). GO analysis showed that these proteins are mainly located in the nucleus and involved in translation initiation, mRNA transport and ribosome biogenesis. The molecular functions of these proteins include translation initiation factor activity, protein activating ATPase activity, protein binding and mRNA binding (Supplementary Fig. S3B). In addition, KEGG analysis showed that these proteins were mainly involved in the spliceosome, ribosome and proteasome pathways (Supplementary Fig. S3B). These strongly negatively correlated maternal proteins were highly expressed in the blastocyst

stage in both biparental and uniparental embryos (Fig. 4E), suggesting their role in blastocoel formation and initial differentiation of embryonic cells.

In this study, a group of candidate maternal proteins that strongly correlated with SCMCs was enriched. Their protein levels showed two similar trends in biparental and uniparental embryos: highest or lowest abundance at the blastocyst stage (Fig. 4E). Obviously, their expression changes were not greatly affected by the paternal genome (or they may be regulated by the maternal genome alone). The final formation of two distinct cell lineages occurs at the blastocyst stage with the formation of the inner cell mass (ICM) and the trophectoderm (TE) [49]. To some extent, maternal proteins may provide the molecular basis for these processes, including cleavage and ICM and TE formation and explain why uniparental embryos can develop to the blastocyst stage and embryonic stem cells can be derived from both biparental and uniparental embryos.

**Some maternal proteins which usually degrade remain in mouse uniparental embryos**

Maternal protein degradation is an important process during early embryogenesis [49], necessary after embryonic genome activation [49]. In this study, to obtain more candidate proteins for further analysis, expression correlation analysis was used to explore the relationship between 15 known maternal proteins identified in both biparental and uniparental embryos (Ooep, Nlrp5, Tle6, Zbed3, Padi6, Atg5, Npm2,

H1foo, Zar1, Oas1d, Pou5f1, Ago2, Dnmt1, Cdh1 and Ctcf, including SCMCs) and other quantified proteins (Supplementary Table S6). This resulted in two reported fertilised embryo proteome databases as in the ZY group (1,958 proteins, Supplementary Fig. S4A). In addition, the detailed intersection relationships of the 15 maternal and other candidate proteins ($|r| \geq 0.70$ and $p \leq 0.05$) are shown in Supplementary Fig. S4B (PA group), Supplementary Fig. S4C (ZY, identified by Gao) and Supplementary Fig. S4D (ZY, identified by Israel).

The Venn analysis among the MII, PA and ZY groups differently sorted these candidate proteins (Fig. 5A, Supplementary Table S8): 765 proteins were detected only in MII oocytes, indicating that these maternal proteins may be involved in oogenesis and oocyte maturation; 397 proteins were detected only in PA embryos, indicating their role in the development of uniparental embryos and possible regulation by the maternal genome; 599 proteins were detected only in ZY embryos, indicating that these proteins may be necessary for the development of biparental embryos and are regulated by the zygote genome; 529 proteins were detected in all groups (MII, PA, ZY), indicating that these maternal proteins may not only be involved in oogenesis and oocyte maturation, but also in embryonic development (including biparental and uniparental embryos); 231 proteins were detected in PA and ZY embryos, indicating their roles in early embryonic development in biparental and uniparental embryos (after fertilisation or artificial activation); and 600 proteins were detected in both MII oocytes and ZY embryos, which reveals that these proteins may not only play a role in oogenesis and oocyte maturation,

but also remain during embryonic development.

Particularly, 316 proteins were detected in MII oocytes and PA embryos, but not in ZY embryos, indicating that these maternal proteins may be degraded after fertilisation in ZY embryos. However, in uniparental embryos, these maternal proteins appear to escape degradation, which indicates a role in parthenogenesis. In the normal mammalian bisexual reproduction, their function may only be regulating oocyte development or maturation. GO analysis showed that these proteins were mainly located in embryos and involved in translation, gene expression and transport. Moreover, KEGG results suggest their involved in pathways related to metabolism, ribosomes and oxidative phosphorylation (Fig. 5B). Furthermore, qPCR verification in uniparental embryos on 12 maternal ribosomal factors involved in 'gene expression' (Fig. 5C) showed coinciding mRNA levels to the protein level (peak at morula or blastocyst stage) (Fig. 5D), suggesting that these maternal proteins remain present though the early development of mouse uniparental embryos.

In biparental embryos, protein degradation occurs after fertilisation [49] and is dependent on the maternally derived ubiquitin-proteasome system and autophagy [50-52]. After the inhibition of proteasomal activity, polyubiquinated proteins accumulate after fertilisation [53]. In this study, we further detected the proteasomal activity of biparental and uniparental embryos in six early developmental stages. The proteasome activity of biparental embryos before the 8-cell stage was stronger than that of

uniparental embryos (Fig. 5E). To some extent, this may allow for the ‘degradation escape’ of these maternal proteins in uniparental embryos. We speculate that in PA embryos these proteins may escape degradation; thus, we called this phenomenon ‘degradation escape’ in uniparental embryos (Fig. 5F).

**Expression and functional analysis of a new maternal factor**

The components of the SCMC include Ooep (Floped), Nlrp5 (Mater), Tle6, Filia, Zbed3, Nlrp2 and possibly Padi6 and Nlrp7 [8, 31, 54]. Among them, Ooep, Nlrp5 and Tle6 directly interact with each other and are necessary to maintain the stability of the complex [1, 6, 31, 55].

In this study, a comparative analysis of candidate proteins strongly correlated with the core SCMC components (Ooep, Nlrp5 and Tle6) in mouse biparental and uniparental embryos highlighted a family of F-box/WD40 repeat-containing proteins (Fbxws). Only Fbxw11 and Fbxw15 were detected in uniparental embryos (PA group, this study), while Fbxw8, Fbxw11, Fbxw13, Fbxw15, Fbxw16, Fbxw18, Fbxw19, Fbxw20, Fbxw21, Fbxw22, Fbxw24, Fbxw26 and Fbxw28 were detected in biparental embryos (ZY group, identified by Gao and Israel; Table 1). Obviously, the quantity of Fbxws in biparental embryos is higher than in uniparental embryos. Furthermore, in uniparental embryos, Fbxw11 was not correlated with SCMC components, but in biparental embryos it showed a strong negative correlation ($r \leq -0.70$, marked in green, Table 1)

with SCMC components. In addition, the expression correlation between Fbxw15 and the SCMC components was the opposite in the two embryos: Fbxw15 had a strong negative correlation ($r \leq -0.70$, marked in green, Table 1) with SCMC components in uniparental embryos, but a strong positive correlation ($r \geq 0.70$, marked in red, Table 1) with SCMC components in biparental embryos (Table 1). Finally, Fbxw24 was selected for further analysis, because its correlation with the other Fbxws was similar to that of SCMC components in biparental embryos (Table 1), and its role in preimplantation embryonic development has not been studied at all yet.

First, an immunofluorescence assay showed predominant cytoplasmic location of Fbxw24 in oocytes and early embryos (Supplementary Fig. S5 and Fig. 6A) and decreasing fluorescence intensity after the 8-cell stage (Fig. 6B). After fertilisation, the mRNA level of Fbxw24 was high at the pronuclear stage, significantly decreasing after the 2-cell stage (Fig. 6C). With cleavage progression, the mRNA level continued to decrease and was almost undetectable at the blastocyst stage; its protein level sharply decreased from the 8-cell to the blastocyst stage (Fig. 6C), coinciding with the fluorescence intensity detected. Although the mouse zygotic genome is activated after the 2-cell stage, Fbxw24 transcript level remained low. These results reveal that Fbxw24 molecules mainly accumulate during oocyte maturation and that *Fbxw24* probably plays a role as a maternal-effect gene in early embryonic development.

Second, Fbxw24 mRNA level was detected using different mouse tissues. We found

that its transcripts in the ovary, but not in 10 other tissues (including testis; Fig. 6D). Immunohistochemistry and immunoblotting were used to assess Fbxw24 expression and location at the protein level. Immunoblotting for 11 tissues detected Fbxw24 protein (~48 kDa) in the mouse ovary, but not in 10 other tissues (Fig. 6E), and the immunohistochemistry results indicated that Fbxw24 was located in oocytes (red arrows) and granulosa cells (red arrowheads) at different follicular stages (Fig. 6F).

Next, small-interfering RNAs (siRNAs) were injected into MII oocytes followed by intracytoplasmic sperm injection (ICSI) to knock down Fbxw24 during early embryogenesis (Fig. 7A). Compared with the control, the developmental competence of Fbxw24-knockdown embryos decreased from the 2-cell stage onwards, and the embryonic development was arrested at the 8-cell stage, failing at the morula and blastocyst stages (Fig. 7B-C). Fbxw24 knockdown embryos were mainly arrested between the 2- and 8-cell stages, showing similar results to those of known maternal-effect genes, such as Nlrp5 [7], Nlrp2 [56] and Padi6 [8].

To study the underlying mechanism, we performed Fbxw24 protein-PPI enrichment by immunoprecipitation followed by FLAG-Fbxw24 MS (Fig. 8A and Supplementary Table S10). Notably, several components of the ubiquitin-mediated proteolysis pathway, including DNA damage-binding protein 1 (Ddb1), Cullin-4B (Cul4b), Ubiquitin conjugation factor E4 B, Ubiquitin conjugation factor E4 A, Ubiquitin-40S ribosomal protein S27a (Rps27a) and Ubiquitin-conjugating enzyme E2S (Ube2s), were detected

in Fbxw24 pull-down complexes (Fig. 8B). The interaction between Fbxw24 and Ddb1-Cul4b was validated in HEK-293T cells by co-immunoprecipitation, and their interaction further explored *in vivo* using C57BL/6J-*Fbxw24*$^{em(Linker-3xFlag)}$ homozygote mouse ovaries (HO) and wild-type littermates as control (Fig. 8C). These results suggest a novel interaction between Fbxw24 and Ddb1-Cul4b. Cullin4 (CUL4) utilises damaged DNA binding protein-1 (DDB1) as linker to interact with a subset of DDB1-Cullin-associated factors [57, 58] and DDB1 is highly expressed in mouse oocytes [59]. Additionally, the expression pattern of 16 members identified in the Fbxw24 pull-down complexes were analysed using MS analysis and verified using PRM in fertilised embryos, revealing different expression patterns during early embryonic stages (Fig. 8D). Among them, SUMO-activating enzyme subunit 1, anaphase-promoting complex subunit 5, cell division cycle protein 16 homolog, protein PML, E3 ubiquitin-protein ligase TRIP12 and SUMO-activating enzyme subunit 2 showed an upward trend; in contrast, Rps27a, Cul4b, STIP1 homology and U box-containing protein 1 and Ube2s showed a downward trend. These results reveal that Fbxw24 may be involved in the regulation of maternal protein degradation during early embryonic development.

## Discussion

The function of maternal proteins was initially reported in invertebrates. Researchers induced mutants in *Drosophila* and found that the polarity of *Drosophila* eggs and embryos is regulated by the maternal factors. Therefore, the importance of maternal

molecules for embryonic development has been demonstrated [60-66]. Subsequently, in vertebrates, the function of maternal factors was also reported, including the fusion of male and female pronucleus, zygotic genome activation and the degradation of maternal components [1, 67]. In mammals, the first maternal factor Mater was reported in mouse [7]. Mater (maternal antigen that embryos require; also known as Nlrp5) may be related to the activation of the zygotic genome [7]. The known maternal-effect factors in the mouse have been reviewed by Li and Zheng [1, 67]. Although the importance of maternal factors for embryonic development has been known for a long time, research progress in mammals has been slow due to the limitations of research materials and technology.

Mouse parthenogenetic embryos are only regulated by the maternal genome, with similar morphology compared to fertilised embryos, so some proteins expressed in parthenogenetic and fertilised embryos may have similar expression trends and their presence and normal expression changes provide a molecular basis for the early development of the two embryos. Uniparental embryos derived from only the oocyte may be a unique model for studying genomic imprinting and the maternal contribution to embryonic development. In addition, the ICM of the blastocyst in parthenogenetic embryos can also be used as a source of parthenogenetic embryonic stem cells (pESCs); this has been successfully established in many species, including mouse, monkey and human [68-71]. Recently, a study reported that fertile mice can be bred from single oocytes by targeted DNA methylation by rewriting seven imprinting control regions

without sperm participation [72]. Although parthenogenetically activated oocytes cannot develop to term in mammals due to the disruption of imprinted gene expression and DNA methylation status, the protein landscape of parthenogenetic embryos has not been studied.

In this study, to obtain more information about maternal proteins, we first constructed the protein database of mouse parthenogenetic embryos before implantation and compared mouse biparental and uniparental embryos at the protein level. By label-free quantitative MS, we detected a total of 2,048 proteins in six preimplantation stages of mouse parthenogenesis and found two similar protein expression patterns in uniparental and biparental embryos; these two patterns may be mainly regulated by the maternal genome. Second, we used the SCMC as target proteins and explored the expression correlation between the SCMC and other identified proteins in biparental and uniparental embryos. We obtained several key candidate maternal proteins among which some were strongly negatively correlated with SCMC and may play an important role in blastocoel formation.

In addition, by analysing the relationship between 15 known maternal proteins and other proteins identified in both biparental and uniparental embryos, we found that some maternal proteins are degraded with oocyte activation in biparental embryos; however, in uniparental embryos, they remain during preimplantation and their mRNA and protein levels show an upward trend. Moreover, the proteasome activity of

biparental embryos before the 8-cell stage was stronger than that of uniparental embryos, which revealed that these maternal proteins in mouse uniparental embryos may escape degradation. Based on these results, we inferred that some maternal protein degradation after oocyte activation may require sperm participation. Fertilisation may trigger the degradation of these maternal ribosomal proteins, or alternatively, the sperm contains some factors that may regulate their degradation. Since the development of uniparental embryos occurs without sperm or any paternal contribution, it leads to a maternal protein 'degradation escape'.

Moreover, among the candidate proteins that strongly correlated with the three core SCMC components (Ooep, Nlrp5 and Tle6), a large set of Fbxws were present in mouse biparental embryos, which may suggest the involvement of the Skp1-Cullin-F-box (SCF) complex in maternal protein degradation in mice. Wang *et al*. [16] also identified a large group of F-box proteins in mouse oocytes and zygotes. SCF protein-ubiquitin ligase complex member F-box proteins are highly abundant in oocytes and two-cell embryos [73]. For example, Fbxw15/Fbxo12J is an F-box protein-encoding gene selectively expressed in oocytes of the mouse ovary [74]. These F-box proteins may play important roles in protein degradation after fertilisation, as different F-box complexes can selectively degrade specific target proteins. Finally, Fbxw24 was selected for further analysis. We found that Fbxw24's expression pattern was very similar to a previously reported maternal-effect factor [8, 75, 76] and, following Fbxw24 knock down by siRNA interference, the affected embryos showed

developmental arrest. By immunoprecipitation-MS (SILAC-IP-MS) and validation *in vivo*, we confirmed a new specific interaction between Fbxw24 and Cul4b-Ddb1 (key components of the ubiquitin-proteasome pathway). We speculate that Fbxw24 may also be involved in the degradation of maternal proteins in early embryos. These results suggest that *Fbxw24* is a new putative maternal-effect gene, and the comparative analysis of biparental and uniparental embryos may be helpful to find out the key maternal factors regulating early embryonic development.

There are some potential limitations to this study. Namely, the quantity of proteins identified in parthenogenetic embryos (2,048 proteins) is lower than that reported before in fertilised embryos. Gao *et al*. identified nearly 5,000 proteins across six developmental stages by TMT, and Israel *et al*. identified 6,550 proteins by SILAC. However, the mouse parthenogenetic embryo only contains maternal genomic information, which may cause some genuine biological absence. Additionally, differences in protein quantity may have occurred due to the following: 1. different proteome construction strategies. The known proteome of fertilised embryos was constructed using a quantitative proteomic strategy based on TMT labelling or SILAC [11, 17], while that of parthenogenetic embryos was constructed by label-free quantitative MS. 2. Different protein databases are used for the search. The raw MS data of fertilised embryos identified by Gao were searched with the ProLucid algorithm against the International Protein Index mouse protein database [17], and the raw MS data of fertilised embryos identified by Israel were searched using MaxQuant software

against the Uniprot KB database [11]. The raw MS data of parthenogenetic embryos were searched using MaxQuant software against the Swiss-Prot mouse database (UP000000589). Importantly, the Swiss-Prot database is manually annotated and reviewed, and the number of proteins is lower than in the IPI and Uniprot KB databases. Moreover, some known maternal proteins, including Brg1, Dppa3, Ezh2, Filia and Zfp57 were detected in this study (uniparental embryos) and in the proteome of biparental embryos identified by Israel, but the one by Gao. These undetected members may hint at different technical limitations, but since these proteins were also detected in parthenogenetic embryos by label-free quantitative MS, the proteome database of uniparental embryos constructed in this study appears reliable. This study reports for the first time the proteome of parthenogenetic embryos, since there is no other protein database or coverage comparison, the existence of false negatives in the parthenogenetic embryo proteome caused by the artificial activation method remains to be investigated.

In conclusion, we systematically constructed a maternal protein file of mouse uniparental embryos and showed the dynamic patterns of maternal proteins during preimplantation using a combined analysis of uniparental and biparental embryos. These data are valuable for further mechanistic studies of maternal proteins and aid in mining potential key players and the associated regulatory mechanisms governing early embryo development.

## Methods

### Experimental animals

C57/BL6J mice were obtained from the animal breeding colony in the Animal experimental centre of Guangxi Medical University, China. To produce C57BL/6J-*Fbxw24*$^{em(Linker-3xFlag)}$ mice, CRISPR/Cas9-mediated homologous recombination (Shanghai Model Organisms Center, Inc) was used to insert the 3x flag sequence and frame it with the last exon of the Fbxw24 gene before the stop codon. The mice were kept in rooms with controlled temperatures under a 12 h light-dark cycle. There was no restriction to food and water, and mice were fed ad libitum.

### Collection of mouse oocytes and embryos

Six- to eight-week-old C57BL/6J female mice were super-ovulated following injection of 10 IU of pregnant mare serum gonadotropin (PMSG; Ningbo Second Hormone Factory, China) and 10 IU of human chorionic gonadotropin (hCG; Ningbo Second Hormone Factory, China). The injection of the two hormones was performed 48 h apart. Fully grown germinal vesicle (GV) oocytes were collected 44–48 h after PMSG injection and Metaphase II (MII) oocytes were collected 12–14 h after hCG injection.

Collection of parthenogenetic embryos was performed following a previously reported protocol [19]. In brief, oocytes surrounding the cumulus cells were recovered from female C57BL/6J mice oviducts after 16–18 h post-hCG administration. When the

cumulus cells were still attached to the oocytes, they were discharged into 7% ethanol freshly prepared in M2 media and permitted to rest for 5 min at room temperature. Then, the oocytes were cleaned three times with M2 media in culture dishes and cumulus cells were eliminated using a hyaluronidase treatment (1 mg/mL, dissolved in M2 media). Next, oocytes were transferred into drops of M16 medium (containing cytochalasin B, 5 ug/mL) and incubated for 4–5 h at 37℃ under 5% $CO_2$ (suppressing the emission of the second polar body and predominantly developing diploid parthenogenetic embryos). Pronucleus formation was observed under the microscope, and activated oocytes with double pronucleus were transferred to KSOM medium for further culture. 2-cell stage embryos were collected after 22–24 h, 4-cell to morula stages embryos were collected after 2–3 days, and the blastocyst stage was collected at day 4.

To obtain fertilised embryos, super-ovulated female mice were mated with male mice. The zygotes (pronucleus stage) were extracted from the female C57BL/6J oviducts at 24 h post-hCG injection. The zygotes were then transferred into a KSOM medium and incubated at 37℃ under 5% $CO_2$. The 2-cell stage fertilised embryos were collected after 22–24 h. The fertilised embryos at the 4-cell to morula stages and blastocyst stage were collected 2–3 days after incubation and at day 4 after incubation, respectively.

**Quantitative MS**

Label-free quantitative MS was performed on parthenogenetic embryos (Pronucleus-, 2-cell-, 4-cell-, 8-cell-, morula- and blastocyst-stage). For each stage, 6000 embryos

were used in three biological duplicates. The lysis buffer (4% SDS,  100 mM Tris-HCl, 1 mM DTT  pH7.6) was used to lyse the samples and extract the proteins. The BCA protein assay kit was utilised to quantify proteins (Bio-Rad, USA). Trypsin digestion was conducted in accordance with the standards for filter-aided sample preparation as previously described [77]. Briefly, around 100 μg of total protein were collected from each specimen and then added into 30 μL SDT buffer (100 mM DTT, 150 mM Tris-HCl, 4% SDS, pH 8.0). Repeated ultrafiltration (Microcon units, 10 kD) was used to filter out the DDT, detergent and other low-molecular-weight substances utilising UA buffer (8 M Urea, 150 mM Tris-HCl, pH 8.0). To inhibit decreased cysteine residues, 100 μL Iodoacetamide (100 mM IAA in UA buffer) was introduced into the specimens and incubated for 30 min in complete darkness. Subsequently, the filters were rinsed thrice using 100 μL of UA buffer and rinsed two times using 100 μL of 25mM $NH_4HCO_3$ buffer. Last, 4 μg trypsin (Promega) was utilised for digesting the protein suspensions overnight at 37°C in 40 μL 25 mM $NH_4HCO_3$ buffer; the obtained peptides were extracted as a filtrate. Peptides from each sample were desalted on a C18 cartridge (Empore™ SPE Cartridge C18 (standard density), bed I.D. 7 mm, volume 3 ml, Sigma), followed by concentration using vacuum centrifugation and reconstitution in 40 μL of 0.1% (v/v) formic acid. Then, liquid chromatography coupled to MS (LC-MS/MS) analysis was performed over 120 min utilising the Q Exactive mass spectrometer (Thermo Scientific) coupled to an EASY-nLC (Proxeon Biosystems, now Thermo Fisher Scientific). A reverse-phase trap column (100 μm × 2 cm, nanoViper C18, Thermo Scientific Acclaim PepMap100) connected to a C18-reverse-phase analytical

column (10 cm long, 75 μm inner diameter, 3 μm resin, Thermo Scientific Easy Column) in buffer A (0.1% Formic acid) was used to load the peptides, which were then isolated utilising buffer B (0.1% Formic acid and 84% acetonitrile) with a linear gradient, at a flow rate of 300 nL/min regulated by IntelliFlow technology. The mass spectrometer positive was set in ion mode. By utilising the data-dependent technique, MS data were obtained via the dynamic selection of the most abundant precursor ions identified by the survey scan (300–1800 m/z) for fragmenting higher energy collisional dissociation (HCD). 3e6 was chosen as the automatic gain control (AGC) target, and 10 ms adjusted as a maximum injection duration. The duration of the dynamic exclusion was adjusted to 40 s. The scan resolution was 70,000 at 200 m/z, whereas HCD spectra were observed using a resolution of 17,500 at 200 m/z, with an isolation width of 2 m/z. The normalised collision energy (NCE), was adjusted to 30 eV while the underfill ratio, that sets the minimum percentage of the target value achieved within the maximum fill time, was specified as 0.1%.

**Identification and bioinformatic analysis**

The MaxQuant software (Max Planck Institute of Biochemistry, Martinsried, Germany, version:1.5.3.17; RRID:SCR_014485) was used to analyse the raw MS data. Protein detection in the MS/MS spectra was accomplished by comparing the spectra to the Swiss-Prot mouse database (UP000000589), which includes 55,366 protein sequences. The following parameters were used: the enzyme chosen was trypsin; the maximum number of missed cleavages was two; for fixed modifications, carbamidomethyl was

chosen; for variable modification, oxidation was chosen; 6 ppm was chosen for the main search and 20 ppm was chosen for the first search and MS/MS tolerance. The database analysis included the following patterns: the included contaminants term was considered true; protein and peptide false discovery rates were <1%; Razor and unique peptides were used in the protein quantitation; a 2-min interval existed between runs (match between runs); the protein quantification technique was LFQ; one was set as the minimum number of ratios. The proteomics data from MS have been submitted to the ProteomeXchange Consortium via the iProX partner repository [78], with the dataset identifier PXD029532. Blast2GO programme was used to map and annotate sequences containing GO terms [79]. R scripts were used to visualise the results of the GO annotation. Subsequently, the investigated proteins were compared with the KEGG database [80] to get their KEGG ortholog identifications. The PPI of the investigated proteins was determined using STRING (RRID:SCR_005223) [81]. The interaction files were visualised by Cytoscape (RRID:SCR_003032; (version 3.2.1) [82].

**Quantitative analysis of selected proteins using parallel reaction monitoring**

Selected proteins were verified by PRM at the protein level. A total of 2500 fertilised embryos of each sample at the six stages (Pronucleus-, 2-cell-, 4-cell-, 8-cell-, morula-, blastocyst-) were acquired for protein extraction. Mass shotgun analysis was performed to obtain pre-experimental results, used to select suitable peptides for PRM analysis. Briefly, to desalt tryptic peptides, they were placed onto C18 analytical column (Thermo Scientific) prior to reversed-phase chromatography on the EASY-nLC™ 1200

system. Gradients of acetonitrile ranging between 5–35% in 45 min were utilised in 1-h LC. A Q Exactive™ Plus Hybrid Quadrupole-Orbitrap™ Mass Spectrometer was used to perform the PRM analysis. The raw data was examined Using the Skyline 3.5.0 software (MacCoss Lab, University of Washington; RRID:SCR_014080) [83]. The PRM validation results of selected proteins are shown in Supplementary Table S11. The proteomics data from MS have been submitted to the ProteomeXchange Consortium via the iProX partner repository [78], with the dataset identifier PXD029532.

**Fbxw24 knockdown in early embryos**

siRNAs of mouse *Fbxw24* and negative control were diluted to 5 mM final concentration using nuclease-free water. Using a Piezo-driven micromanipulator, about 10 pL of 5-mM siRNAs were delivered into the oocytes. Then, the implanted oocytes were cultured for ≥3 h to prepare for ICSI. About 1 mL of a sperm suspension was combined with the HEPES-buffered Chatot-Ziomek-Bavister (HCZB) medium drop comprising of 10% (w/v) polyvinylpyrrolidone (Irvine Scientific, Santa Ana, CA, USA). Using several Piezo pulses, the sperm head and tail were separated and the head was then injected into the oocyte according to the procedure by Ward and Yanagimachi [84]. HCZB medium was used for gamete handling and ICSI, whereas Chatot-Ziomek-Bavister (CZB) medium was used for embryo culturing at 5% $CO_2$. For embryo culture, CZB was overlaid with sterile mineral oil (Sigma). To analyse siRNA knockdown efficiency, the total RNA of 15 embryos at the 4-cell stage was purified by the RNeasy Mini Kit (QIAGEN, catalogue number 74104). Reverse transcription was used to

synthesise cDNA (Promega). The StepOneTM Real-Time PCR System (Applied Biosystems) was used to perform quantitative real-time PCR, using H2afz as internal control. Primer sequences and siRNA-targeting sequences are shown in Supplementary Table S9.

**Measuring mRNA levels using Real-time PCR**

The isolation of total RNA from embryos was carried out at six stages (Pronucleus-, 2-cell, 4-cell, 8-cell, Morula-, Blastocyst-) utilising the RNeasy Mini Kit (QIAGEN, catalogue number 74104). Reverse transcription technique (Promega) was used to synthesise cDNA. The Real-Time PCR System (Applied Biosystems) was used to perform quantitative real-time PCR analysis. The relative expression levels were calculated using the $2^{-\triangle\triangle CT}$ method. All experiments were carried out in three biological repetitions. The primer sequences are shown in Supplementary Table S9.

**Immunoblotting, immunofluorescence and immunohistochemistry analysis**

For immunoblotting analysis, the different tissues were obtained from C57BL/6J-*Fbxw24*$^{\mathrm{em(Linker\text{-}3xFlag)}}$ mice and the extracted proteins were isolated using sodium dodecyl sulfate-polyacrylamide gel electrophoresis (SDS-PAGE). Using a semidry western blotting technology (Trans-Blot® TurboTM System, Bio-Rad, Singapore), the proteins were then deposited onto a polyvinylidene difluoride membrane. Then, the membrane was blocked with 5% nonfat milk for 1 h at 37°C before using the primary antibodies and incubated at 4°C overnight. The membrane was cleaned three times

using a solution composed of Tris-buffered saline and Tween 20 (TBST buffer), followed by incubation with the secondary antibodies in TBST for 1 h at 37°C. An alkaline phosphatase detection kit (C3206, Beyotime Biotechnology Inc, Shanghai, China) was used to identify the presence of proteins.

For immunofluorescence analysis, C57BL/6J-*Fbxw24*$^{\mathrm{em(Linker\text{-}3xFlag)}}$ female mice oocytes or embryos were kept at room temperature for 30 min in 4% polyoxymethylene. In addition, the embryos were placed into a 1% Triton X-100 phosphate-buffered saline (PBS) solution for 20 min, followed by a solution of PBS comprising of 1% bovine serum albumin (BSA) for blocking. Next, embryos were incubated overnight at 4℃, followed by combination with a primary antibody at an effective concentration. Then, embryos were washed three times with a PBS solution containing 0.1% Tween-20 and 0.01% Triton X-100 for 2 min each. The embryos were incubated at room temperature in a diluted solution of a secondary antibody (fluorescein isothiocyanate labelled) and washed thrice for 2 min each with a PBS solution containing 0.1% Tween-20 and 0.01% Triton. The specimen was covered using a coverslip coated in a ProLong Gold antifade reagent containing DAPI (Life Technologies) and stored in the darkness until fluorescence was determined using an inverted fluorescence microscope (Olympus, IX73, Japan). Fluorescence intensity was measured using ImageJ (National Institutes of Health; RRID:SCR_003070).

For immunohistochemistry analysis, C57BL/6J-*Fbxw24*$^{\mathrm{em(Linker\text{-}3xFlag)}}$ female mouse

ovaries were fixed in 4% paraformaldehyde and paraffin was used to embed them. The ovarian sections (5 μm) were deparaffinised using xylene and rehydrated in serial ethanol dilutions. The sections were rinsed in 1% PBS-Tween-20 and treated using 2% hydrogen peroxide. The incubation with specified primary antibodies lasted 2 h at room temperature after blocking using 3% goat serum. The sections were treated using the secondary antibody for 40 min at 37℃. Negative controls were processed with PBS instead of the primary antibody. Sections were analysed with a light microscope (Olympus, IX73, Japan). The following primary antibodies were utilised: anti-beta anti-GAPDH rabbit monoclonal antibody (ab181602, Abcam) and anti-DDDDK tag (Binds to FLAG® tag sequence) rabbit monoclonal antibody (ab205606, Abcam).

**Proteasome activity assay**

Proteasome activity assays were performed as previously reported with minor modifications [85]. To assess oocyte proteasome activity at six embryonic development stages of mouse biparental and uniparental embryos, 100 embryos per group were collected and washed three times in PBS/PVP. All cell suspensions were lysed in 20 μL of protein extraction buffer composed of 150 mM sodium chloride, 50 mM Tris and 0.5% Triton X-100 for 30 min under constant rotation at 4℃. After centrifugation at 16,300 g for 15 min, the supernatants were transferred to a clean tube and assessed for proteasome activity using a commercial proteasome assay kit, which uses an AMC tagged peptide substrate which releases free, highly fluorescent AMC in case of proteolytic activity (Abcam, ab107921). In brief, 10 μL of each sample was loaded into

a 96-well plate in duplicate, alongside a Jurkat cell lysate-positive control (supplied) and AMC protein standards. A total of 50 μM of the proteasome inhibitor MG132 was added to one well of each sample to differentiate proteasome activity from other protease activity in the samples. Plates were incubated for 25 min and analysed on a TECAN Sunrise™ plate reader (Austria) at 350/440 nm excitation/emission. After a further 35 min incubation at 37℃, plates were analysed a second time to calculate the change in relative fluorescence units in each sample. Data were analysed following the manufacturers' instructions, and proteasome activity was calculated such that one unit of proteasome activity is equivalent to the amount of proteasome activity that generates 1.0 nmol of AMC per min at 37℃. This experiment was repeated across three independent biological and technical replicates using 100 embryos per assay.

**Stable isotope labelling of amino acids in cell culture and immunoprecipitation-MS (SILAC-IP-MS)**

Following SILAC labelling [86, 87], HEK-293T cells were maintained in DMEM medium containing 13C6-Lysine (K6) and 13C6-Arginine (R6) (Cambridge Isotope Laboratories, Inc.), supplemented with 10% dialysed fetal bovine serum (Invitrogen) for seven passages for complete labelling of the cellular proteome. The mouse Fbxw24 gene (NM_001013776) was cloned into a p3×FLAG-CMV-7.1 vector (Sigma-Aldrich) between the NotI and SalI sites and transfected into HEK-293T cells maintained in normal DMEM medium (Light, L) using lipofectamine 2000 (Invitrogen). The empty vector p3×FLAG-CMV-7.1 was transfected into K6R6-labelled HEK-293T cells as

control (Heavy, H). 20 μM MG132 (SelleckChem) was added to the transfection solution for six additional hours before collection. The collected cells from the L and H groups were lysed using 150 mM NaCl, 20 mM Tris (pH 7.5) and 2 % Triton X-100 supplemented with a phosphatase and protease inhibitor cocktail. Anti-FLAG beads (Sigma-Aldrich) were added for immunioprecipitation (IP) and the enriched proteins were eluted by 3×FLAG peptide (Sigma-Aldrich). The final eluates obtained after Flag peptide elution were analysed by SDS-PAGE. Coomassie Brilliant Blue dye was used for staining and visible bands were excised for LC-MS/MS analysis. Peptide extraction and in-gel protein trypsin digestion were carried out according to the previously described protocol [88]. Analysis of peptide samples was performed on an EASY-nLC 1000 system (Thermo Fisher Scientific, Waltham, MA) connected to an Orbitrap Fusion mass spectrometer (Thermo Fisher Scientific, San Jose, CA). Then, 10 μL of solvent A (water comprising 0.1% formic acid) was utilised to resuspend the peptide, followed by loading of 8 μL peptide sample onto a trap column (100 μm×2 cm, Thermo Scientific Acclaim PepMap C18) for 3 min at a flow rate of 10 μL/min, followed by separation on an analytical column (Acclaim PepMap C18, 75 μm x 25 cm) with a linear gradient. The flow rate inside the column was maintained at 300 nL/min. Using the data-dependent method, the Orbitrap Fusion mass spectrometer switched automatically between MS and MS/MS acquisition. The orbitrap was utilised to acquire survey full-scan MS spectra (m/z 350-1600) with 120,000 mass resolution, 1000,000 AGC and 50 ms utmost duration for injection. MS/MS acquisition was performed in an orbitrap at maximum speed mode and a cycle period of 3 s, with mass resolution 15,000, AGC

target 100,000, utmost injection duration 80 ms and isolation width 1.6 m/z. HCD was used to break down ions with charge states 2+, 3+ and 4+ consecutively, with 30% normalised collision energy Microscans were recorded utilising dynamic exclusion for 30 s in all cases. Maxquant software (version 1.5.2.8) [89] was utilised to process the MS raw data for protein identification and quantitation. We employed the Andromeda search engine [90] to search for relevant data in mouse UniProtKB/Swiss-Prot databases. The following criteria were established: (1) required peptide length ≥7 amino acids. (2) trypsin cleavage specificity, a maximum of two missing cleavages permitted. (3) Oxidation (M) and acetylation (protein N-term) were the two variable modifications considered. (4) For both precursor and fragment ions, the initial mass variation was as high as 10 ppm and 5 Da, respectively. (5) At both protein and peptide levels, the false discovery rate was set at 1%. (6) A multiplicity of 2 was used, where 13C6-Lysine (Lys6, K6) and 13C6-Arginine (Arg6, R6) were selected as heavy (H) labels. (7) 'Re-quantify' option was selected. (8) Quantification with unmodified unique and razor peptides, as well as a minimum of two counted ratios. (9) Any protein that sequenced ≥2 peptides was considered a reliable indentation. Statistical analysis of specific interactions was evaluated utilising significance B ($p < 0.05$), by Perseus (version 1.6.7.0; RRID:SCR_015753) [91] on the log2 L/H ratio. The significance B value indicates whether a ratio differs from the distribution of all protein ratios grouped by intensity. The proteomics data from MS have been submitted to the ProteomeXchange Consortium via the iProX partner repository [78], with the dataset identifier PXD029532.

## IP and western blotting

We used 5 µg of anti-DDDDK tag rabbit monoclonal antibody to coat magnetic Beads Protein G (binds to the FLAG tag sequence) in IP wash buffer (50 nM Tris-HCl, 150 nM sodium chloride, 0.05% NP-40 and 1 mM $MgCl_2$, pH 7.4) with 5% BSA and incubated overnight at 4°C with rotation. HEK-293T cells (in normal DMEM medium) and C57BL/6J-*Fbxw24*$^{em(Linker-3xFlag)}$ HO mouse ovaries were added to IP lysis buffer (50 mM Tris-HCl, 150 mM NaCl, 1 mM EDTA, 0.1% SDS, 0.5% sodium deoxycholate, 1% NP-40, 1 mM PMSF/cocktail and 0.5 mM DTT, pH: 7.4), followed by 10 min incubation on ice. HEK-293T cells (in K6R6-labelled DMEM medium) and ovaries of wild-type littermates were used as control, respectively. Then, the IP lysate was centrifuged at 14,000 rpm and 4°C for 10 min; 100 µL supernatant was removed and mixed with 900 µL of the beads-antibody complex in IP buffer (35 µL 0.5 M EDTA and 860 µL IP wash buffer), followed by overnight incubation with 4°C rotation. After rinsing, 50 mL elution buffer was added into the immunoprecipitate, and the supernatant utilised for western blot. Four commercial antibodies were employed: anti-Ddb1 antibody (ab109027, Abcam), anti-Cul4b antibody (12916-1-AP, Proteintech Group), anti-DDDDK tag (Binds to FLAG® tag sequence) rabbit monoclonal antibody (Binds to FLAG® tag sequence) (ab205606, Abcam) and anti-beta anti-GAPDH rabbit monoclonal antibody (ab181602, Abcam).

## Statistical analysis

Statistical analyses were conducted using the IBM Statistical software (SPSS 23.0). The mean ± standard error of the mean was used to express the data. Either a one-way analysis of variance or an unpaired t-test with Tukey's post hoc test was utilised for comparison of the statistical data (*P < 0.05).

## Data Availability

The mouse uniparental embryo proteome that constructed in this study (deposited via the PRIDE partner repository) is available via this accession number in ProteomeXchange: PXD029532. All other supporting data and materials are available in the *GigaScience* GigaDB database [92].

## Ethics approval

All experimental protocols were reviewed and approved by the Animal Care and Use Committee of Guangxi Medical University (No.201901012).

## Competing interests

The authors declare that they have no competing interests.


## Funding

This study was funded by China Postdoctoral Science Foundation (2019M653810XB), the Guangxi Natural Science Foundation Program (2019JJB140131) and Guangxi First-class Discipline Project for Basic medicine Sciences (No.GXFCDP-BMS-2018).

## Author Contributions

FC, XL, TX and YZ performed the experiments. BM, YL, FC, YL and YZ supervised, and analyzed experimental data. FC, QZ, BL and XX wrote the manuscript.

## Acknowledgments

We thank Dr. Shisheng Wang (West China Hospital, Sichuan University) and Dr. Chengpin Shen (Omicsolution Co., Ltd) for giving some advices about data analysis and 'Wu Kong' platform (https://www.omicsolution.com/wkomics/main/) for the Fuzzy C-means clustering, PCA, hierarchical clustering analysis, upset plot and venn diagram analysis.

## Figure legends

**Figure 1**. Temporal profiles of maternal protein expression in mouse uniparental embryos. (**A**) Statistical histogram of mass spectrometry results from mouse uniparental embryos. 2048 proteins were identified and 1902 proteins were quantified. Total spectrum: total number of secondary spectra; Matched spectrum: the number of spectra matched to the database; Total Peptides: the number of all peptides; Unique peptides: the number of unique peptides; Detected Proteins: the number of proteins detected; Quantified Proteins: The number of proteins that can be quantified. (**B**) UpSet intersection diagram of proteins detected in six stages of mouse uniparental embryos. The blue horizontal bar shows the number of proteins identified from each group, while

the line with solid orange dots below the X-axis indicates an intersection among different groups, and the black vertical bar on the X-axis shows the number of proteins for the corresponding intersection. PA, PA2, PA4, PA8, PAMO, and PABL represent different embryonic stages of parthenogenesis: Pronucleus stage (PA), 2-cell stage (PA2), 4-cell stage (PA4), 8-cell stage (PA8), Morula stage (PAMO), Blastocyst stage (PABL). (**C**) Hierarchical grouping analysis of maternal proteins in six developmental stages of mouse uniparental embryos. (**D**) Maternal protein expression patterns analysis using principal component analysis (PCA). (**E**) Fuzzy c-means clustering analysis of protein expression during uniparental embryonic development. Color gradient corresponds to the cluster membership value. All protein expression changes quantified in this study are centred and scaled around a mean of 0 and a standard deviation of 1. The colors purple and red indicate proteins with membership values that are above 0.5, whereas yellow and green denote proteins with membership values that are less than 0.5.

**Figure 2**. General comparative analysis of three proteomic databases constructed by parthenogenetic embryo (uniparental embryo, PA group), fertilised embryo (biparental embryo, ZY group), and mature oocyte (MII). (**A**) Venn diagram(green) showed the intersection of MII groups, quantified by Wang and Israel, respectively; Venn diagram(blue) showed the intersection of ZY groups, quantified by Gao and Israel, respectively. A sum of 1029 proteins was found among the three different groups (PA,MII,ZY). (**B**) The expression patterns of five SCMC components (Ooep, Nlrp5,

Tle6, Zbed3 and Padi6, named the SCMCs in this study) in fertilised and parthenogenetic embryos, and their verification by parallel reaction monitoring (PRM) assay in fertilised embryo. PA_*this study*, quantified in this study; ZY_*Gao et al.,2017*, quantified by Gao *et al*, 2017; ZY_*Israel et al.,2019*, quantified by Israel *et al*, 2019; PRM_*this study*, validated by PRM assay using fertilised embryos in this study. PN-pronucleus stage; 2C-2-cell stage; 4C-4-cell stage; 8C-8-cell stage; MO-morula stage; BL-blastocyst stage. Pronucleus stage was used as control. (**C-E**) Hierarchical clustering of protein expression for the 1029 proteins in PA group (**C**), ZY group quantified by Gao *et al*.,2017 (**D**) and ZY group quantified by Israel *et al*.,2019 (**E**) , respectively.

**Figure 3**. (**A**) Venn diagrams between MII and PA groups. 1168 proteins detected both in MII and PA groups, and k-means clustering (k=6) analysis is conducted for the 1168 proteins through the development of uniparental embryo. (**B**) Venn diagrams between MII and ZY groups. 1806 proteins detected both in MII and ZY groups, and k-means clustering (k=6) analysis is conducted for the 1806 proteins using the protein expression database of biparental embryo identified by Gao and Israel, respectively. (**C**) Venn diagrams between ZY and PA groups. 613 proteins were only detected in PA group, and k-means clustering (k=6) analysis is conducted for the 613 proteins through the development of uniparental embryo. (**D**) Schematic diagram of key biological events during mouse embryogenesis. E0.5 represents the 0.5 days after fertilization.

**Figure 4**. Maternal proteins that are strongly correlated with the SCMCs in mouse biparental and uniparental embryos. (**A**) Venn diagram showed the intersection between three groups (PA_Chen, represented the proteins that are strongly correlated with the SCMCs in mouse uniparental embryos; ZY_Israel, represented the proteins that are strongly correlated with the SCMCs in the proteome of the mouse biparental embryos quantified by Israel; ZY_Gao, represented the proteins that are strongly correlated with the SCMCs in the proteome of the mouse biparental embryos quantified by Gao). 429 proteins were detected in both mouse biparental and uniparental embryos; 113 were strongly positively correlated ($r \geq 0.70$), 304 were strongly negatively correlated ($r \leq -0.70$). (**B**) The KEGG annotation for the 429 candidate maternal proteins. (**C**) The protein-protein interaction (PPI) network of the candidate maternal proteins that are involved in biological process of "Translation".The proteins highlighted in the red box were used for further validation by PRM assay. (**D**) The validation results by PRM assay for the selected 8 key candidate proteins using fertilised embryos. PA_Chen, represented the expression pattern in mouse uniparental embryos; ZY_Israel, represented the expression pattern in the proteome of the mouse biparental embryos quantified by Israel; ZY_Gao, represented the expression pattern in the proteome of the mouse biparental embryos quantified by Gao. ZY_PRM validation, represented the validation results by PRM assay in this study. (**E**) Schematic diagram showed the expression patterns between the candidate maternal proteins and the SCMCs during mouse embryogenesis.

**Figure 5**. (**A**) The Venn diagram showed the intersection of the candidate proteins that strongly correlated with 15 key maternal proteins in three groups. The blue circle shows the number of candidate proteins that were analyzed from the proteome of biparental embryos (ZY group, identified by Gao and Israel), the green circle shows the number of the candidate proteins that were analyzed from the proteome of uniparental embryos (PA group, identified in this study), and the light orange circle shows the maternal proteins quantified in metaphase II (MII) stage oocytes (MII group, quantified by Wang and Israel). 316 maternal proteins that are only detected in PA and MII groups, and in biparental embryos (ZY), these proteins were detected in the MII stage oocyte but degraded after fertilised. (**B**) The annotation results for the 316 maternal proteins. The green columns showed the annotation of biological process, the blue columns showed the annotation of KEGG and the red columns showed the localization in different tissues. (**C**) The protein-protein interaction (PPI) network of the candidate maternal proteins that are involved in biological process of "gene expression". (**D**) 12 maternal ribosomal factors involved in the process of "gene expression" were validated by RT-qPCR assay using uniparental embryos. The lines represented the expression pattern in protein level, and columns represented the expression pattern in mRNA level. Pronucleus stage was used as control. *$P < 0.05$. (**E**) The proteasome activity was detected in six developmental stages of mouse biparental and uniparental embryos using a fluorometric proteasome activity assay kit. One unit of proteasome activity is defined as the amount of proteasome that generates 1.0 nmol of the fluorescently tagged AMC per min at 37°C. (**F**). Schematic diagram of “Degradation Escape” maternal

proteins. MII, metaphase II of oocyte; 7% CH2CH2OH, M2 solution contains 7% ethanol. Some maternal proteins may "escape" from degradation in mouse uniparental embryos.

**Figure 6**. The expression analysis of Fbxw24 in mouse. (**A**) Cellular localization of Fbxw24. The different stage embryos were cultured from the zygotes of C57BL/6J-*Fbxw24*$^{\mathrm{em(Linker\text{-}3xFlag)}}$ mice. Each sample was performed using DAPI to allow for the visualization of DNA (blue). scale bar: 20 μm. PN-pronucleus stage; 2C-2-cell stage; 4C-4-cell stage; 8C-8-cell stage; MO-morula stage; BL-blastocyst stage. (**B**) Quantification of Fbxw24 fluorescence intensity in six biparental embryo stages. Pronucleus stage was used as control. $^{*}P < 0.05$. (**C**) The relative transcripts and protein abundance of Fbxw24 in mouse fertilised embryos.$^{*}P < 0.05$. (**D**) The mRNA level of Fbxw24 in different tissues of the mouse. GAPDH was used as a control. (**E**) Immunoblots of lysates isolated from different tissues of C57BL/6J-*Fbxw24*$^{\mathrm{em(Linker\text{-}3xFlag)}}$ mice. GAPDH used as a loading control. (**F**) Immunohistochemical analysis of Fbxw24 in ovary. The sections obtained from 4-week-old C57BL/6J-*Fbxw24*$^{\mathrm{em(Linker\text{-}3xFlag)}}$ heterozygous female mice. The wild-type female littermate was a negative control. The representative oocytes with positivity at different follicular stages are indicated by red arrows and granulosa cells with positivity are indicated by red arrowheads. (Magnification: 100×,400×).

**Figure 7**. The function analysis of Fbxw24 during mouse embryo development. (**A**)

Efficiency of siRNA-mediated knockdown of Fbxw24. SD is shown by the error bars. (**B**) Percentage of successfully developed embryos at each stage following Fbxw24 knockdown. The development rate is calculated with the quantity of zygotes as the denominator. SD is shown by the error bars. (**C**) Knockdown of Fbxw24 results in the developmental arrest that started from E1.5. Scale bars represent 100 μm.

**Figure 8**. Fbxw24 interact with Ddb1-Cul4b in mouse. (**A**) Fbxw24 was pulled down and eluted by an N-terminal 3xFlag tag. An SDS-PAGE was used to isolate the extracts obtained from the 3xFlag-target, then the extracts were observed by Coomassie brilliant blue staining. Extraction of seven Fbxw24 immunoprecipitated products were performed for subsequent in-gel trypsin digestion. (**B**) Sixteen members of ubiquitin-mediated proteolysis pathways were identified in Fbxw24 pull-down complexes by LC-MS/MS analysis. (**C**) A Co-IP assay in HEK-293T cells and ovaries of C57BL/6J-*Fbxw24*$^{\text{em (Linker-3xFlag)}}$ mice was conducted to validate the interaction of Ddb1-Cul4b with Fbxw24. NC: Negative Control; OE: Overexpression; WT: Wild Type mice; HO: Homozygote mice. (**D**) The expression pattern analysis of sixteen members identified in Fbxw24 pull-down complexes by MS analysis and verification by PRM in fertilised embryos. The red line displayed the change of protein level from the previously reported proteome of mouse fertilised embryos identified by Gao, and the blue line displayed the results of PRM analysis in this study. The pronucleus stage was used as a control.

## Tables

Table 1. The expression correlation coefficient of Fbxws and SCMC components.

## Additional files

Table S1. Summary of maternal protein and peptide profiles identified in mouse uniparental embryos.

Table S2. The detailed information of UpSet intersection diagram and the annotation of each intersection for the proteome of mouse uniparental embryos.

Table S3. The detailed results of Fuzzy c-means clustering analysis using the proteome of mouse uniparental embryos.

Table S4. The detailed results of Venn diagram in mouse mature oocytes (MII), fertilised (ZY) and parthenogenetic (PA) embryos.

Table S5. The detailed results of Fuzzy c-means clustering analysis related to Fig.3.

Table S6. The results of expression correlation for 15 key maternal proteins and other quantified proteins in PA and ZY groups.

Table S7. The maternal proteins that are strongly correlated with SCMC components in PA and ZY groups.

Table S8. The detailed results of Venn diagram related to Fig.5A.

Table S9. Primers for Realtime PCR and target sequence of siRNAs.

Table S10. Putative Fbxw24 interactors identified by Fbxw24 IP-MS.

Table S11. The parallel reaction monitoring (PRM) validation results of selected proteins.

Figure S1. (A) The developmental morphology of different stages after activation of ethanol combined with CB. The visualization of DNA was performed using DAPI (blue). Scale bar: 20μm. (B) The developmental rate after activation of ethanol combined with CB in mouse oocyte.

Figure S2. Annotation for maternal protein expression clusters in mouse uniparental embryos.(A).The heat map showed the significance value of the biological processes in 10 clusters.(B-D).The interaction networks of GO terms from four categories are displayed, including “translation”(B), “peptide metabolic process”(C), “nucleic acid metabolic process” (D) and “cellular metabolic process” (E).

Figure S3. (A).The protein-protein interaction (PPI) network of the candidate maternal proteins that are strongly negative with the SCMCs. (B). The GO and KEGG annotation for the the candidate maternal proteins that are strongly negative with the SCMCs.

Figure S4. (A) The Venn diagram showed the intersection of the candidate proteins that strongly correlated with 15 key maternal proteins in two reported proteome databases of fertilised embryos identified by Gao and Israel, respectively. (B-D) The UpSet intersection diagram of 15 key maternal proteins and their candidate proteins ($|r| \geq 0.70$ and $p \leq 0.05$) in PA group (B) and ZY group (C-identified by Gao, D-identified by Israel).

Figure S5. The presence of Fbxw24 protein in oocytes. The oocytes of GV and MII stages were collected from the C57BL/6J-*Fbxw24*$^{em(Linker\text{-}3xFlag)}$ mice. Each sample was performed using DAPI to allow for the visualization of DNA (blue). scale bar: 20 μm. GV-germinal vesicle stage; MII-metaphase II stage.

## References


1. Li L, Zheng P, Dean J. Maternal control of early mouse development. Development. 2010;137:859–70. doi:10.1242/dev.039487.
2. Burns KH, Viveiros MM, Ren Y, Wang P, DeMayo FJ, Frail DE, et al. Roles of NPM2 in chromatin and nucleolar organization in oocytes and embryos. Science. 2003;300:633–6. doi:10.1126/science.1081813.
3. Gao Z, Zhang X, Yu X, Qin D, Xiao Y, Yu Y, et al. Zbed3 participates in the subcortical maternal complex and regulates the distribution of organelles. J Mol Cell Biol. 2018;10:74–88. doi:10.1093/jmcb/mjx035.
4. Mahadevan S, Sathappan V, Utama B, Lorenzo I, Kaskar K, van den Veyver IB. Maternally expressed NLRP2 links the subcortical maternal complex (SCMC) to fertility, embryogenesis and epigenetic reprogramming. Sci Rep. 2017;7:44667. doi:10.1038/srep44667.
5. Tashiro F, Kanai-Azuma M, Miyazaki S, Kato M, Tanaka T, Toyoda S, et al. Maternal-effect gene Ces5/Ooep/Moep19/Floped is essential for oocyte cytoplasmic lattice formation and embryonic development at the maternal-zygotic stage transition. Genes Cells. 2010;15:813–28. doi:10.1111/j.1365-2443.2010.01420.x.
6. Yu X-J, Yi Z, Gao Z, Qin D, Zhai Y, Chen X, et al. The subcortical maternal complex controls symmetric division of mouse zygotes by regulating F-actin dynamics. Nat Commun. 2014;5:4887. doi:10.1038/ncomms5887.
7. Tong ZB, Gold L, Pfeifer KE, Dorward H, Lee E, Bondy CA, et al. Mater, a

maternal effect gene required for early embryonic development in mice. Nat Genet. 2000;26:267–8. doi:10.1038/81547.

8. Yurttas P, Vitale AM, Fitzhenry RJ, Cohen-Gould L, Wu W, Gossen JA, Coonrod SA. Role for PADI6 and the cytoplasmic lattices in ribosomal storage in oocytes and translational control in the early mouse embryo. Development. 2008;135:2627–36. doi:10.1242/dev.016329.
9. Fan X, Tang D, Liao Y, Li P, Zhang Y, Wang M, et al. Single-cell RNA-seq analysis of mouse preimplantation embryos by third-generation sequencing. PLoS Biol. 2020;18:e3001017. doi:10.1371/journal.pbio.3001017.
10. Xue Z, Huang K, Cai C, Cai L, Jiang C, Feng Y, et al. Genetic programs in human and mouse early embryos revealed by single-cell RNA sequencing. Nature. 2013;500:593–7. doi:10.1038/nature12364.
11. Israel S, Ernst M, Psathaki OE, Drexler HCA, Casser E, Suzuki Y, et al. An integrated genome-wide multi-omics analysis of gene expression dynamics in the preimplantation mouse embryo. Sci Rep. 2019;9:13356. doi:10.1038/s41598-019-49817-3.
12. Deutsch DR, Fröhlich T, Otte KA, Beck A, Habermann FA, Wolf E, Arnold GJ. Stage-specific proteome signatures in early bovine embryo development. J Proteome Res. 2014;13:4363–76. doi:10.1021/pr500550t.
13. Lucitt MB, Price TS, Pizarro A, Wu W, Yocum AK, Seiler C, et al. Analysis of the zebrafish proteome during embryonic development. Mol Cell Proteomics. 2008;7:981–94. doi:10.1074/mcp.M700382-MCP200.

14. Peshkin L, Wühr M, Pearl E, Haas W, Freeman RM, Gerhart JC, et al. On the Relationship of Protein and mRNA Dynamics in Vertebrate Embryonic Development. Dev Cell. 2015;35:383–94. doi:10.1016/j.devcel.2015.10.010.
15. Wühr M, Freeman RM, Presler M, Horb ME, Peshkin L, Gygi S, Kirschner MW. Deep proteomics of the Xenopus laevis egg using an mRNA-derived reference database. Curr Biol. 2014;24:1467–75. doi:10.1016/j.cub.2014.05.044.
16. Wang S, Kou Z, Jing Z, Zhang Y, Guo X, Dong M, et al. Proteome of mouse oocytes at different developmental stages. Proc Natl Acad Sci U S A. 2010;107:17639–44. doi:10.1073/pnas.1013185107.
17. Gao Y, Liu X, Tang B, Li C, Kou Z, Li L, et al. Protein Expression Landscape of Mouse Embryos during Pre-implantation Development. Cell Rep. 2017;21:3957–69. doi:10.1016/j.celrep.2017.11.111.
18. Israel S, Casser E, Drexler HCA, Fuellen G, Boiani M. A framework for TRIM21-mediated protein depletion in early mouse embryos: recapitulation of Tead4 null phenotype over three days. BMC Genomics. 2019;20:755. doi:10.1186/s12864-019-6106-2.
19. Kaufman MH. Parthenogenetic Activation of Oocytes. Cold Spring Harb Protoc 2018. doi:10.1101/pdb.prot094409.
20. Cuellar O. Animal parthenogenesis. Science. 1977;197:837–43. doi:10.1126/science.887925.
21. Brevini TAL, Pennarossa G, Vanelli A, Maffei S, Gandolfi F. Parthenogenesis in non-rodent species: developmental competence and differentiation plasticity.

Theriogenology. 2012;77:766–72. doi:10.1016/j.theriogenology.2011.11.010.

22. Peng M, Huang H, Jin F. Progress in research on oocytes parthenogenetic activation. Zhejiang Da Xue Xue Bao Yi Xue Ban. 2007;36:307–12. doi:10.3785/j.issn.1008-9292.2007.03.017.

23. Tachibana M, Amato P, Sparman M, Gutierrez NM, Tippner-Hedges R, Ma H, et al. Human embryonic stem cells derived by somatic cell nuclear transfer. Cell. 2013;153:1228–38. doi:10.1016/j.cell.2013.05.006.

24. Ahn J, Hwang I-S, Park M-R, Cho I-C, Hwang S, Lee K. The Landscape of Genomic Imprinting at the Porcine SGCE/PEG10 Locus from Methylome and Transcriptome of Parthenogenetic Embryos. G3 (Bethesda). 2020;10:4037–47. doi:10.1534/g3.120.401425.

25. Ahn J, Wu H, Lee J, Hwang I-S, Yu D, Ahn J-S, et al. Identification of a Novel Imprinted Transcript in the Porcine GNAS Complex Locus Using Methylome and Transcriptome of Parthenogenetic Fetuses. Genes (Basel) 2020. doi:10.3390/genes11010096.

26. Chen F, Fu Q, Pu L, Zhang P, Huang Y, Hou Z, et al. Integrated Analysis of Quantitative Proteome and Transcriptional Profiles Reveals the Dynamic Function of Maternally Expressed Proteins After Parthenogenetic Activation of Buffalo Oocyte. Mol Cell Proteomics. 2018;17:1875–91. doi:10.1074/mcp.RA118.000556.

27. Du Z-Q, Liang H, Liu X-M, Liu Y-H, Wang C, Yang C-X. Single cell RNA-seq reveals genes vital to in vitro fertilised embryos and parthenotes in pigs. Sci Rep.

2021;11:14393. doi:10.1038/s41598-021-93904-3.

28. Kajdasz A, Warzych E, Derebecka N, Madeja ZE, Lechniak D, Wesoly J, Pawlak P. Lipid Stores and Lipid Metabolism Associated Gene Expression in Porcine and Bovine Parthenogenetic Embryos Revealed by Fluorescent Staining and RNA-seq. Int J Mol Sci 2020. doi:10.3390/ijms21186488.
29. Leng L, Sun J, Huang J, Gong F, Yang L, Zhang S, et al. Single-Cell Transcriptome Analysis of Uniparental Embryos Reveals Parent-of-Origin Effects on Human Preimplantation Development. Cell Stem Cell. 2019;25:697-712.e6. doi:10.1016/j.stem.2019.09.004.
30. Zhang C, Li C, Yang L, Leng L, Jovic D, Wang J, et al. The Dynamic Changes of Transcription Factors During the Development Processes of Human Biparental and Uniparental Embryos. Front Cell Dev Biol. 2021;9:709498. doi:10.3389/fcell.2021.709498.
31. Lu X, Gao Z, Qin D, Li L. A Maternal Functional Module in the Mammalian Oocyte-To-Embryo Transition. Trends Mol Med. 2017;23:1014–23. doi:10.1016/j.molmed.2017.09.004.
32. Zaccara S, Ries RJ, Jaffrey SR. Reading, writing and erasing mRNA methylation. Nat Rev Mol Cell Biol. 2019;20:608–24. doi:10.1038/s41580-019-0168-5.
33. Li X, Ito M, Zhou F, Youngson N, Zuo X, Leder P, Ferguson-Smith AC. A maternal-zygotic effect gene, Zfp57, maintains both maternal and paternal imprints. Dev Cell. 2008;15:547–57. doi:10.1016/j.devcel.2008.08.014.
34. Posfai E, Kunzmann R, Brochard V, Salvaing J, Cabuy E, Roloff TC, et al.

Polycomb function during oogenesis is required for mouse embryonic development. Genes Dev. 2012;26:920–32. doi:10.1101/gad.188094.112.

35. Nakamura T, Arai Y, Umehara H, Masuhara M, Kimura T, Taniguchi H, et al. PGC7/Stella protects against DNA demethylation in early embryogenesis. Nat Cell Biol. 2007;9:64–71. doi:10.1038/ncb1519.

36. Otten ABC, Kamps R, Lindsey P, Gerards M, Pendeville-Samain H, Muller M, et al. Tfam Knockdown Results in Reduction of mtDNA Copy Number, OXPHOS Deficiency and Abnormalities in Zebrafish Embryos. Front Cell Dev Biol. 2020;8:381. doi:10.3389/fcell.2020.00381.

37. Yan W, Ma L, Stein P, Pangas SA, Burns KH, Bai Y, et al. Mice deficient in oocyte-specific oligoadenylate synthetase-like protein OAS1D display reduced fertility. Mol Cell Biol. 2005;25:4615–24. doi:10.1128/MCB.25.11.4615-4624.2005.

38. Fukuda T, Tokunaga A, Sakamoto R, Yoshida N. Fbxl10/Kdm2b deficiency accelerates neural progenitor cell death and leads to exencephaly. Mol Cell Neurosci. 2011;46:614–24. doi:10.1016/j.mcn.2011.01.001.

39. Zheng P, Dean J. Role of Filia, a maternal effect gene, in maintaining euploidy during cleavage-stage mouse embryogenesis. Proc Natl Acad Sci U S A. 2009;106:7473–8. doi:10.1073/pnas.0900519106.

40. Hirasawa R, Chiba H, Kaneda M, Tajima S, Li E, Jaenisch R, Sasaki H. Maternal and zygotic Dnmt1 are necessary and sufficient for the maintenance of DNA methylation imprints during preimplantation development. Genes Dev.

2008;22:1607–16. doi:10.1101/gad.1667008.

41. Inoue A, Chen Z, Yin Q, Zhang Y. Maternal Eed knockout causes loss of H3K27me3 imprinting and random X inactivation in the extraembryonic cells. Genes Dev. 2018;32:1525–36. doi:10.1101/gad.318675.118.

42. O'Carroll D, Erhardt S, Pagani M, Barton SC, Surani MA, Jenuwein T. The polycomb-group gene Ezh2 is required for early mouse development. Mol Cell Biol. 2001;21:4330–6. doi:10.1128/MCB.21.13.4330-4336.2001.

43. Larue L, Ohsugi M, Hirchenhain J, Kemler R. E-cadherin null mutant embryos fail to form a trophectoderm epithelium. Proc Natl Acad Sci U S A. 1994;91:8263–7. doi:10.1073/pnas.91.17.8263.

44. Bultman SJ, Gebuhr TC, Pan H, Svoboda P, Schultz RM, Magnuson T. Maternal BRG1 regulates zygotic genome activation in the mouse. Genes Dev. 2006;20:1744–54. doi:10.1101/gad.1435106.

45. Maenohara S, Unoki M, Toh H, Ohishi H, Sharif J, Koseki H, Sasaki H. Role of UHRF1 in de novo DNA methylation in oocytes and maintenance methylation in preimplantation embryos. PLoS Genet. 2017;13:e1007042. doi:10.1371/journal.pgen.1007042.

46. Li R, Albertini DF. The road to maturation: somatic cell interaction and self-organization of the mammalian oocyte. Nat Rev Mol Cell Biol. 2013;14:141–52. doi:10.1038/nrm3531.

47. Bebbere D, Masala L, Albertini DF, Ledda S. The subcortical maternal complex: multiple functions for one biological structure? J Assist Reprod Genet.

2016;33:1431–8. doi:10.1007/s10815-016-0788-z.

48. Akoglu H. User’s guide to correlation coefficients. Turk J Emerg Med. 2018;18:91–3. doi:10.1016/j.tjem.2018.08.001.

49. Toralova T, Kinterova V, Chmelikova E, Kanka J. The neglected part of early embryonic development: maternal protein degradation. Cell Mol Life Sci. 2020;77:3177–94. doi:10.1007/s00018-020-03482-2.

50. Huo L-J, Fan H-Y, Zhong Z-S, Chen D-Y, Schatten H, Sun Q-Y. Ubiquitin-proteasome pathway modulates mouse oocyte meiotic maturation and fertilization via regulation of MAPK cascade and cyclin B1 degradation. Mech Dev. 2004;121:1275–87. doi:10.1016/j.mod.2004.05.007.

51. Tsukamoto S, Kuma A, Murakami M, Kishi C, Yamamoto A, Mizushima N. Autophagy is essential for preimplantation development of mouse embryos. Science. 2008;321:117–20. doi:10.1126/science.1154822.

52. Yamamoto A, Mizushima N, Tsukamoto S. Fertilization-induced autophagy in mouse embryos is independent of mTORC1. Biol Reprod. 2014;91:7. doi:10.1095/biolreprod.113.115816.

53. Higuchi C, Shimizu N, Shin S-W, Morita K, Nagai K, Anzai M, et al. Ubiquitin-proteasome system modulates zygotic genome activation in early mouse embryos and influences full-term development. J Reprod Dev. 2018;64:65–74. doi:10.1262/jrd.2017-127.

54. Akoury E, Gupta N, Bagga R, Brown S, Déry C, Kabra M, et al. Live births in women with recurrent hydatidiform mole and two NLRP7 mutations. Reprod

Biomed Online. 2015;31:120–4. doi:10.1016/j.rbmo.2015.03.011.

55. Li L, Baibakov B, Dean J. A subcortical maternal complex essential for preimplantation mouse embryogenesis. Dev Cell. 2008;15:416–25. doi:10.1016/j.devcel.2008.07.010.

56. Peng H, Chang B, Lu C, Su J, Wu Y, Lv P, et al. Nlrp2, a maternal effect gene required for early embryonic development in the mouse. PLoS One. 2012;7:e30344. doi:10.1371/journal.pone.0030344.

57. Angers S, Li T, Yi X, MacCoss MJ, Moon RT, Zheng N. Molecular architecture and assembly of the DDB1-CUL4A ubiquitin ligase machinery. Nature. 2006;443:590–3. doi:10.1038/nature05175.

58. Jackson S, Xiong Y. CRL4s: the CUL4-RING E3 ubiquitin ligases. Trends Biochem Sci. 2009;34:562–70. doi:10.1016/j.tibs.2009.07.002.

59. Yu C, Zhang Y-L, Pan W-W, Li X-M, Wang Z-W, Ge Z-J, et al. CRL4 complex regulates mammalian oocyte survival and reprogramming by activation of TET proteins. Science. 2013;342:1518–21. doi:10.1126/science.1244587.

60. Anderson KV, Nüsslein-Volhard C. Information for the dorsal—ventral pattern of the Drosophila embryo is stored as maternal mRNA. Nature. 1984;311:223–7. doi:10.1038/311223a0.

61. Anderson KV, Bokla L, Nüsslein-Volhard C. Establishment of dorsal-ventral polarity in the drosophila embryo: The induction of polarity by the Toll gene product. Cell. 1985;42:791–8. doi:10.1016/0092-8674(85)90275-2.

62. Driever W, Nüsslein-Volhard C. The bicoid protein determines position in the

Drosophila embryo in a concentration-dependent manner. Cell. 1988;54:95–104. doi:10.1016/0092-8674(88)90183-3.

63. Nüsslein-Volhard C, Lohs-Schardin M, Sander K, Cremer C. A dorso-ventral shift of embryonic primordia in a new maternal-effect mutant of Drosophila. Nature. 1980;283:474–6. doi:10.1038/283474a0.

64. Nüsslein-Volhard C, Frohnhöfer HG, Lehmann R. Determination of anteroposterior polarity in Drosophila. Science. 1987;238:1675–81. doi:10.1126/science.3686007.

65. Nüsslein-Volhard C, Wieschaus E. Mutations affecting segment number and polarity in Drosophila. Nature. 1980;287:795–801. doi:10.1038/287795a0.

66. Schupbach T, Wieschaus E. Germline autonomy of maternal-effect mutations altering the embryonic body pattern of Drosophila. Developmental Biology. 1986;113:443–8. doi:10.1016/0012-1606(86)90179-x.

67. Zheng W, Liu K. Maternal control of mouse preimplantation development. Results Probl Cell Differ. 2012;55:115–39. doi:10.1007/978-3-642-30406-4_7.

68. Chen Y, Ai A, Tang ZY, Zhou GD, Liu W, Cao Y, Zhang WJ. Mesenchymal-like stem cells derived from human parthenogenetic embryonic stem cells. Stem Cells Dev. 2012;21:143–51. doi:10.1089/scd.2010.0585.

69. Elling U, Taubenschmid J, Wirnsberger G, O'Malley R, Demers S-P, Vanhaelen Q, et al. Forward and reverse genetics through derivation of haploid mouse embryonic stem cells. Cell Stem Cell. 2011;9:563–74. doi:10.1016/j.stem.2011.10.012.

70. Leeb M, Wutz A. Derivation of haploid embryonic stem cells from mouse embryos. Nature. 2011;479:131–4. doi:10.1038/nature10448.
71. Yang H, Liu Z, Ma Y, Zhong C, Yin Q, Zhou C, et al. Generation of haploid embryonic stem cells from Macaca fascicularis monkey parthenotes. Cell Res. 2013;23:1187–200. doi:10.1038/cr.2013.93.
72. Wei Y, Yang C-R, Zhao Z-A. Viable offspring derived from single unfertilised mammalian oocytes. Proc Natl Acad Sci U S A. 2022;119:e2115248119. doi:10.1073/pnas.2115248119.
73. Knowles BB, Evsikov AV, Vries WN de, Peaston AE, Solter D. Molecular control of the oocyte to embryo transition. Philos Trans R Soc Lond B Biol Sci. 2003;358:1381–7. doi:10.1098/rstb.2003.1330.
74. La Chesnaye E de, Kerr B, Paredes A, Merchant-Larios H, Méndez JP, Ojeda SR. Fbxw15/Fbxo12J is an F-box protein-encoding gene selectively expressed in oocytes of the mouse ovary. Biol Reprod. 2008;78:714–25. doi:10.1095/biolreprod.107.063826.
75. Mahadevan S, Sathappan V, Utama B, Lorenzo I, Kaskar K, van den Veyver IB. Erratum: Maternally expressed NLRP2 links the subcortical maternal complex (SCMC) to fertility, embryogenesis and epigenetic reprogramming. Sci Rep. 2017;7:46434. doi:10.1038/srep46434.
76. Yurttas P, Morency E, Coonrod SA. Use of proteomics to identify highly abundant maternal factors that drive the egg-to-embryo transition. Reproduction. 2010;139:809–23. doi:10.1530/REP-09-0538.

77. Wiśniewski JR, Zougman A, Nagaraj N, Mann M. Universal sample preparation method for proteome analysis. Nat Methods. 2009;6:359–62. doi:10.1038/nmeth.1322.

78. Ma J, Chen T, Wu S, Yang C, Bai M, Shu K, et al. iProX: an integrated proteome resource. Nucleic Acids Res. 2019;47:D1211-D1217. doi:10.1093/nar/gky869.

79. Conesa A, Götz S, García-Gómez JM, Terol J, Talón M, Robles M. Blast2GO: a universal tool for annotation, visualization and analysis in functional genomics research. Bioinformatics. 2005;21:3674–6. doi:10.1093/bioinformatics/bti610.

80. Kanehisa M, Sato Y, Kawashima M, Furumichi M, Tanabe M. KEGG as a reference resource for gene and protein annotation. Nucleic Acids Res. 2016;44:D457-62. doi:10.1093/nar/gkv1070.

81. Szklarczyk D, Franceschini A, Wyder S, Forslund K, Heller D, Huerta-Cepas J, et al. STRING v10: protein-protein interaction networks, integrated over the tree of life. Nucleic Acids Res. 2015;43:D447-52. doi:10.1093/nar/gku1003.

82. Shannon P, Markiel A, Ozier O, Baliga NS, Wang JT, Ramage D, et al. Cytoscape: a software environment for integrated models of biomolecular interaction networks. Genome Res. 2003;13:2498–504. doi:10.1101/gr.1239303.

83. MacLean B, Tomazela DM, Shulman N, Chambers M, Finney GL, Frewen B, et al. Skyline: an open source document editor for creating and analyzing targeted proteomics experiments. Bioinformatics. 2010;26:966–8. doi:10.1093/bioinformatics/btq054.

84. Ward MA, Yanagimachi R. Intracytoplasmic Sperm Injection in Mice. Cold

Spring Harb Protoc 2018. doi:10.1101/pdb.prot094482.

85. Mihalas BP, Bromfield EG, Sutherland JM, Iuliis GN de, McLaughlin EA, Aitken RJ, Nixon B. Oxidative damage in naturally aged mouse oocytes is exacerbated by dysregulation of proteasomal activity. J Biol Chem. 2018;293:18944–64. doi:10.1074/jbc.RA118.005751.

86. Ong S-E, Mann M. A practical recipe for stable isotope labeling by amino acids in cell culture (SILAC). Nat Protoc. 2006;1:2650–60. doi:10.1038/nprot.2006.427.

87. Ong S-E, Blagoev B, Kratchmarova I, Kristensen DB, Steen H, Pandey A, Mann M. Stable isotope labeling by amino acids in cell culture, SILAC, as a simple and accurate approach to expression proteomics. Mol Cell Proteomics. 2002;1:376–86. doi:10.1074/mcp.m200025-mcp200.

88. Shevchenko A, Tomas H, Havlis J, Olsen JV, Mann M. In-gel digestion for mass spectrometric characterization of proteins and proteomes. Nat Protoc. 2006;1:2856–60. doi:10.1038/nprot.2006.468.

89. Cox J, Mann M. MaxQuant enables high peptide identification rates, individualized p.p.b.-range mass accuracies and proteome-wide protein quantification. Nat Biotechnol. 2008;26:1367–72. doi:10.1038/nbt.1511.

90. Cox J, Neuhauser N, Michalski A, Scheltema RA, Olsen JV, Mann M. Andromeda: a peptide search engine integrated into the MaxQuant environment. J Proteome Res. 2011;10:1794–805. doi:10.1021/pr101065j.

91. Tyanova S, Temu T, Sinitcyn P, Carlson A, Hein MY, Geiger T, et al. The Perseus computational platform for comprehensive analysis of (prote)omics data. Nat

Methods. 2016;13:731‑40. doi:10.1038/nmeth.3901.
92. Chen F; Ma B; Lin Y; Luo X; Xu T; Zhang Y; Chen F; Li Y; Zhang Y; Luo B; Zhang Q; Xie X. Supporting data for "Comparative maternal protein profiling of mouse biparental and uniparental embryos" GigaScience Database 2022. http://dx.doi.org/10.5524/102243.

Table 1 The expression correlation coefficient of Fbxws and SCMC components

| Gene name | Groups | Ooep | | Nlrp5 | | Tle6 | | Fbxw24 | |
|---|---|---|---|---|---|---|---|---|---|
| | | p | r | p | r | p | r | p | r |
| Fbxw8 | PA_this study | — | — | — | — | — | — | — | — |
| | ZY_Israel | 0.5822 | 0.2864 | 0.6216 | 0.2580 | 0.3570 | 0.4614 | 0.2948 | 0.5159 |
| | ZY_Gao | 0.0009 | -0.9749 | 0.0000 | -0.9946 | 0.0002 | -0.9889 | 0.0021 | -0.9628 |
| Fbxw11 | PA_this study | 0.7422 | 0.1736 | 0.5370 | 0.3195 | 0.7132 | 0.1936 | — | — |
| | ZY_Israel | 0.0570 | -0.7981 | 0.0067 | -0.9325 | 0.0287 | -0.8582 | 0.0502 | -0.8109 |
| | ZY_Gao | 0.0561 | -0.7997 | 0.1031 | -0.7250 | 0.0924 | -0.7404 | 0.0346 | -0.8440 |
| Fbxw13 | PA_this study | — | — | — | — | — | — | — | — |
| | ZY_Israel | 0.3686 | 0.4517 | 0.1762 | 0.6342 | 0.3035 | 0.5081 | 0.1168 | 0.7062 |
| | ZY_Gao | 0.0000 | 0.9949 | 0.0009 | 0.9755 | 0.0004 | 0.9837 | 0.0002 | 0.9898 |
| Fbxw15 | PA_this study | 0.0111 | -0.9128 | 0.0247 | -0.8687 | 0.0063 | -0.9342 | — | — |
| | ZY_Israel | 0.0137 | 0.9027 | 0.0153 | 0.8972 | 0.0228 | 0.8742 | 0.0009 | 0.9755 |
| | ZY_Gao | 0.0010 | 0.9742 | 0.0037 | 0.9499 | 0.0021 | 0.9626 | 0.0014 | 0.9696 |
| Fbxw16 | PA_this study | — | — | — | — | — | — | — | — |
| | ZY_Israel | 0.0246 | 0.8689 | 0.0135 | 0.9037 | 0.0680 | 0.7787 | 0.0090 | 0.9214 |
| | ZY_Gao | 0.0003 | 0.9865 | 0.0019 | 0.9645 | 0.0010 | 0.9744 | 0.0002 | 0.9895 |
| Fbxw18 | PA_this study | — | — | — | — | — | — | — | — |
| | ZY_Israel | 0.0057 | 0.9376 | 0.0065 | 0.9336 | 0.0208 | 0.8797 | 0.0013 | 0.9708 |
| | ZY_Gao | 0.0005 | 0.9810 | 0.0044 | 0.9452 | 0.0026 | 0.9585 | 0.0008 | 0.9775 |
| Fbxw19 | PA_this study | — | — | — | — | — | — | — | — |
| | ZY_Israel | 0.0329 | 0.8479 | 0.0299 | 0.8554 | 0.0337 | 0.8461 | 0.0486 | 0.8141 |
| | ZY_Gao | 0.0000 | 0.9978 | 0.0003 | 0.9850 | 0.0001 | 0.9909 | 0.0001 | 0.9942 |
| Fbxw20 | PA_this study | — | — | — | — | — | — | — | — |
| | ZY_Israel | 0.0462 | 0.8189 | 0.0308 | 0.8530 | 0.0325 | 0.8491 | 0.0025 | 0.9585 |
| | ZY_Gao | 0.0001 | 0.9917 | 0.0013 | 0.9705 | 0.0006 | 0.9798 | 0.0002 | 0.9896 |
| Fbxw21 | PA_this study | — | — | — | — | — | — | — | — |
| | ZY_Israel | 0.0178 | 0.8891 | 0.0019 | 0.9644 | 0.0090 | 0.9216 | 0.0019 | 0.9638 |
| | ZY_Gao | 0.0000 | 0.9944 | 0.0008 | 0.9772 | 0.0003 | 0.9851 | 0.0001 | 0.9923 |
| Fbxw22 | PA_this study | — | — | — | — | — | — | — | — |
| | ZY_Israel | 0.0040 | 0.9480 | 0.0037 | 0.9497 | 0.0251 | 0.8677 | 0.0044 | 0.9454 |
| | ZY_Gao | 0.0001 | 0.9931 | 0.0006 | 0.9795 | 0.0003 | 0.9865 | 0.0002 | 0.9893 |
| Fbxw26 | PA_this study | — | — | — | — | — | — | — | — |
| | ZY_Israel | 0.0328 | 0.8482 | 0.0438 | 0.8238 | 0.0175 | 0.8900 | 0.0640 | 0.7857 |
| | ZY_Gao | 0.0000 | 0.9947 | 0.0002 | 0.9871 | 0.0001 | 0.9921 | 0.0000 | 0.9967 |
| Fbxw28 | PA_this study | — | — | — | — | — | — | — | — |
| | ZY_Israel | 0.0209 | 0.8796 | 0.0238 | 0.8712 | 0.0095 | 0.9192 | 0.0107 | 0.9142 |
| | ZY_Gao | 0.0021 | 0.9621 | 0.0071 | 0.9303 | 0.0044 | 0.9451 | 0.0035 | 0.9510 |

Figure.2

Click here to access/download;Figure;Figure.2.tif

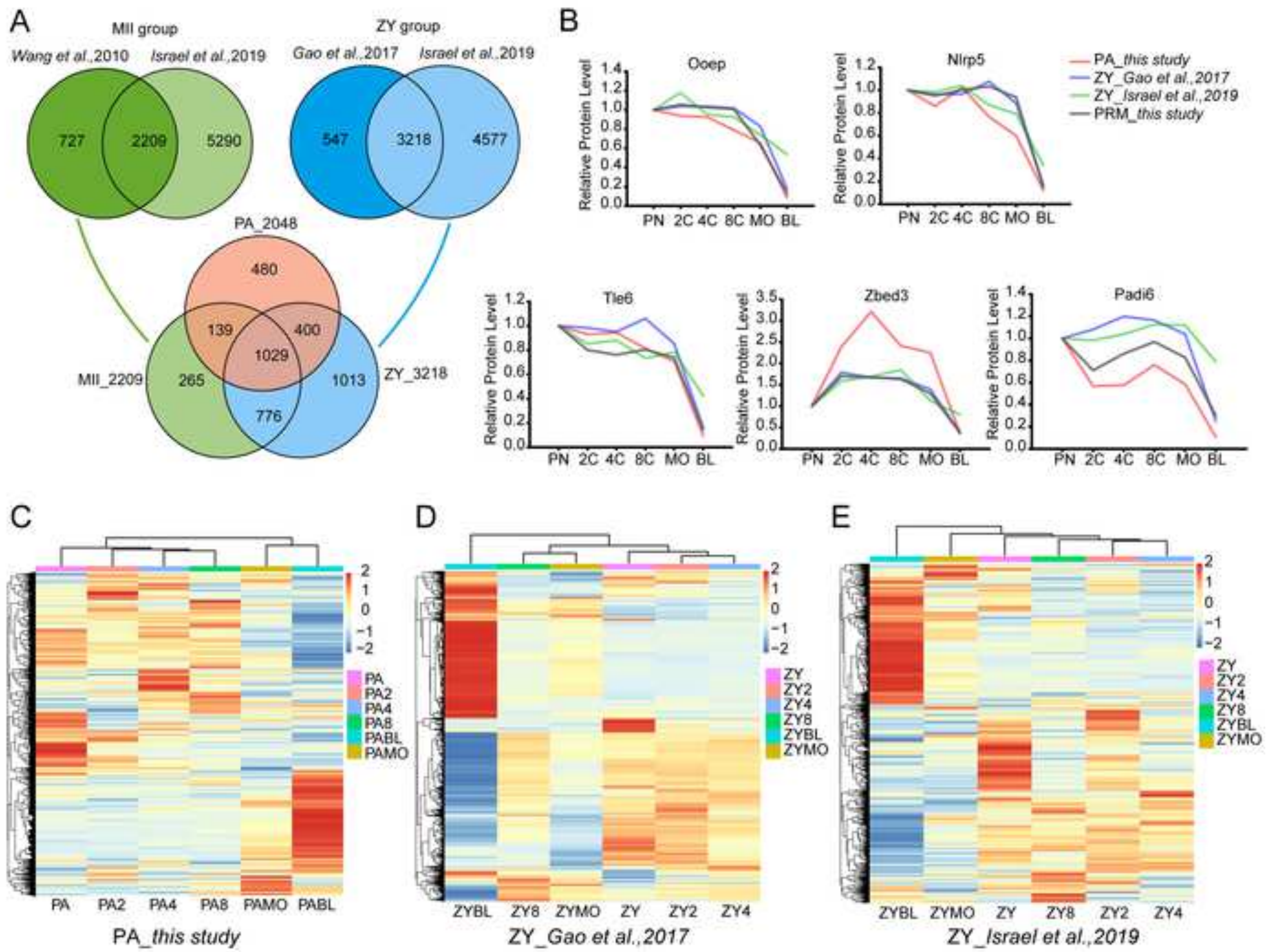

Figure.3

Click here to access/download;Figure;Figure.3.tif

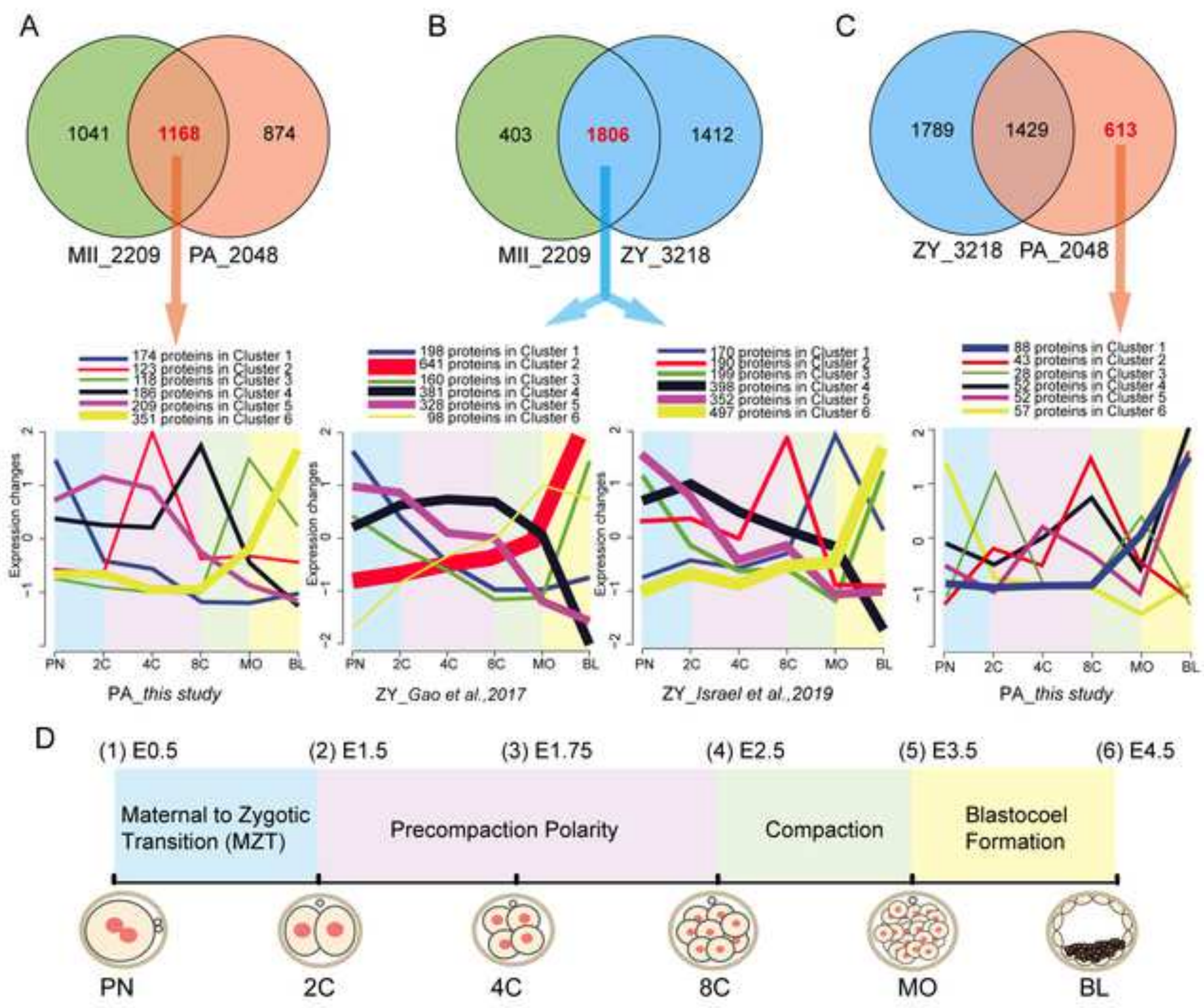

Figure.6

Click here to access/download;Figure;Figure.6.tif

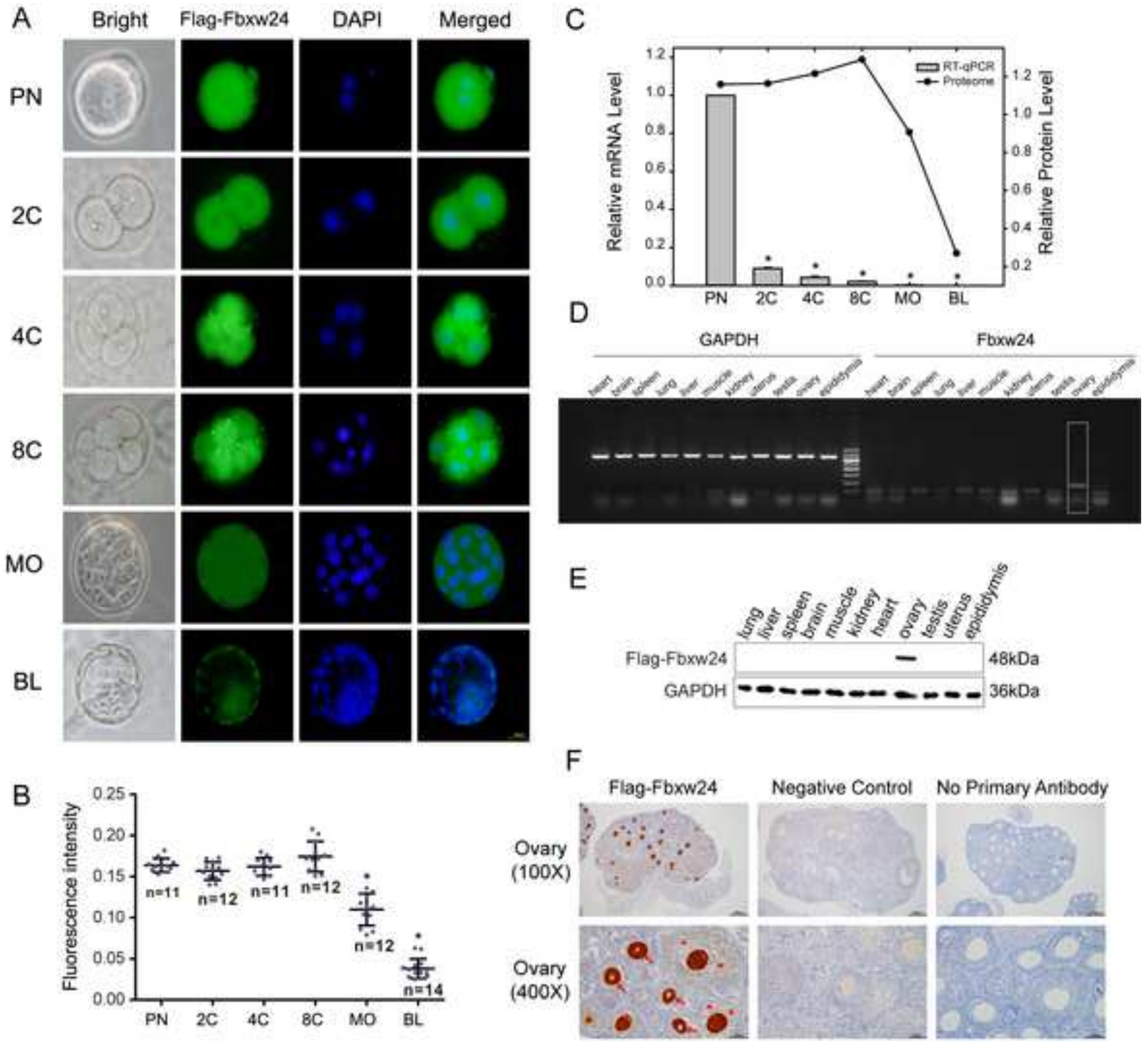

Figure.7

Click here to access/download;Figure;Figure.7.tif

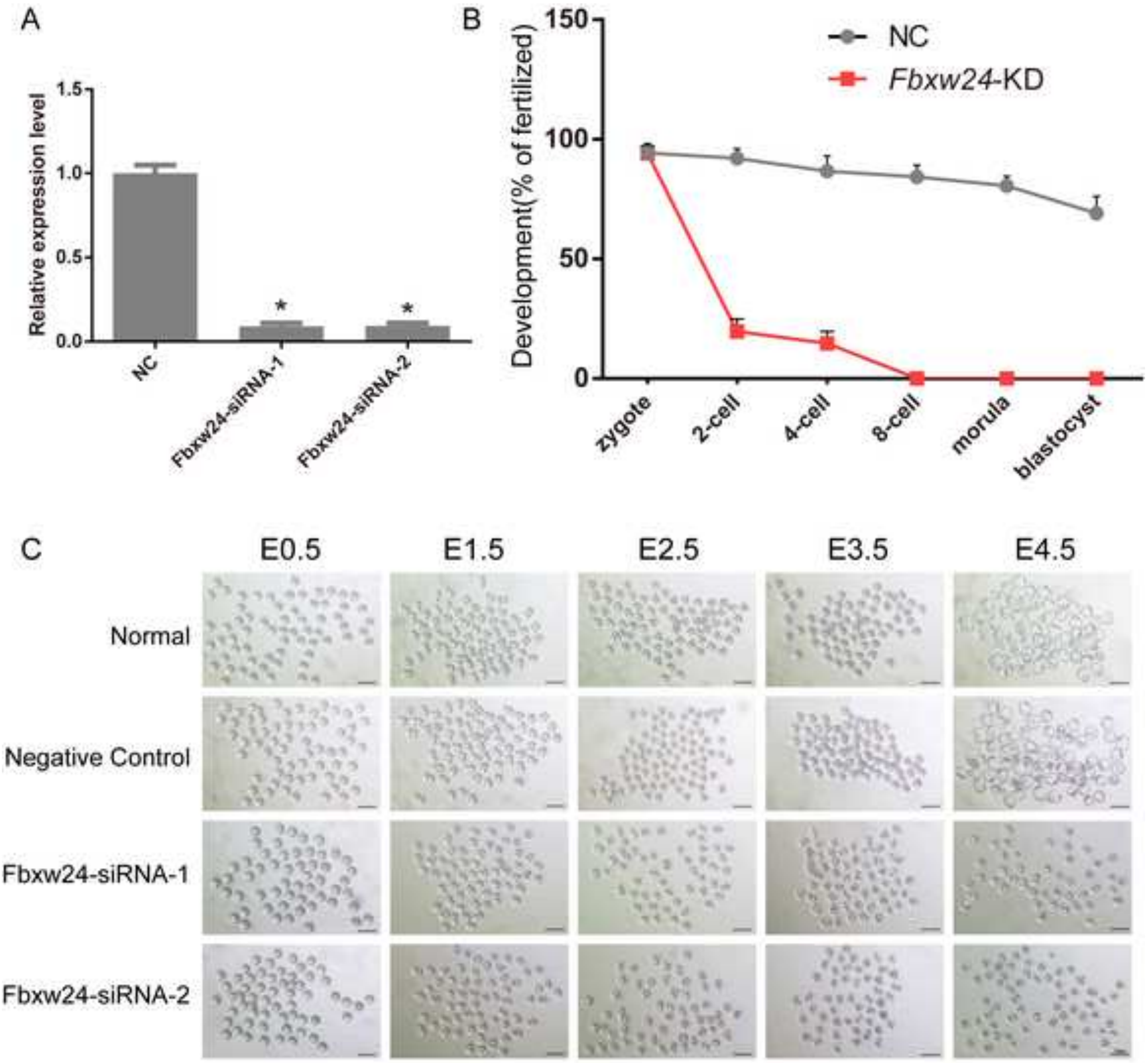

Figure.8

Click here to access/download;Figure;Figure.8.tif

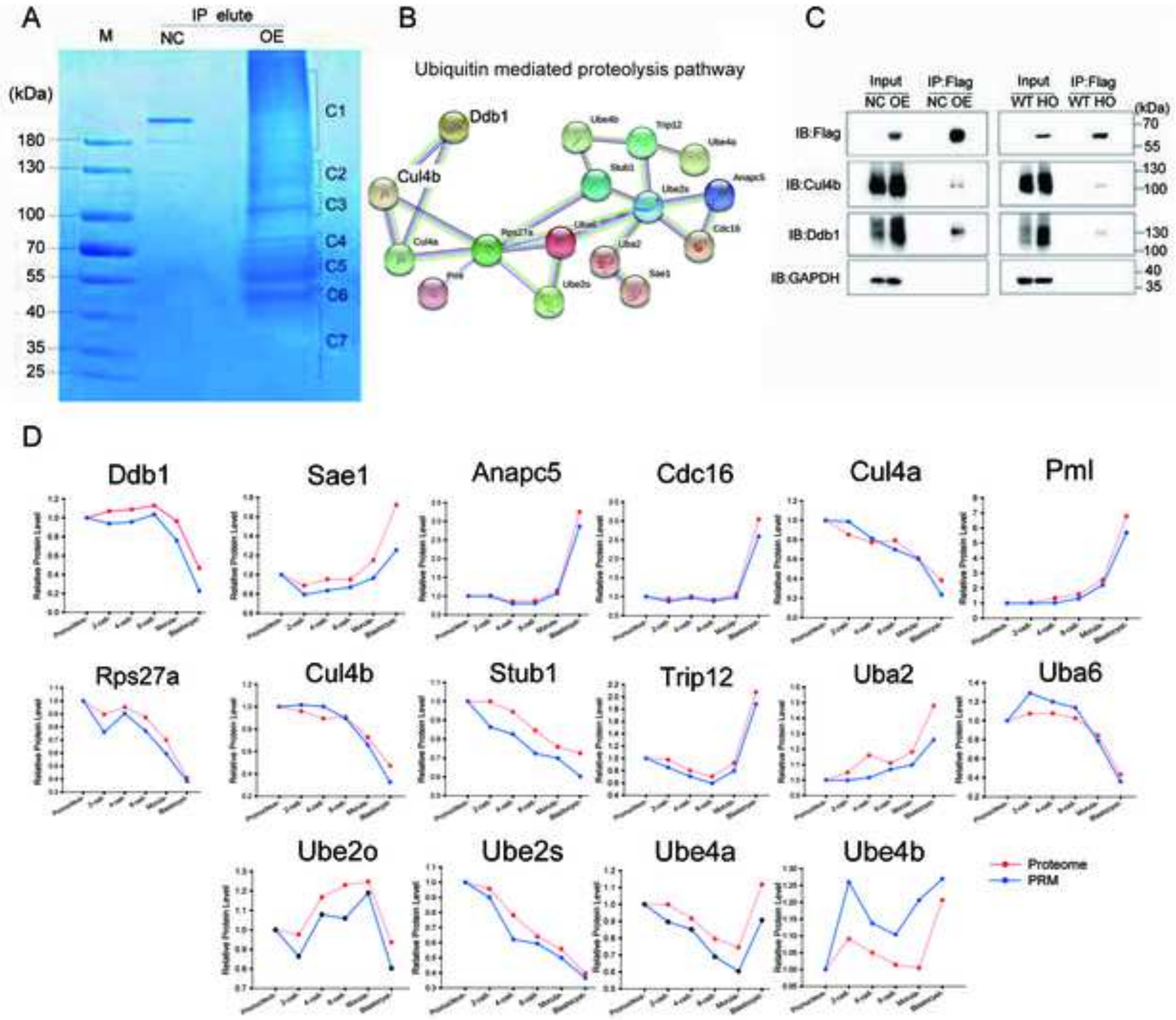

Figure.1

Click here to access/download;Figure;Figure.1.tif

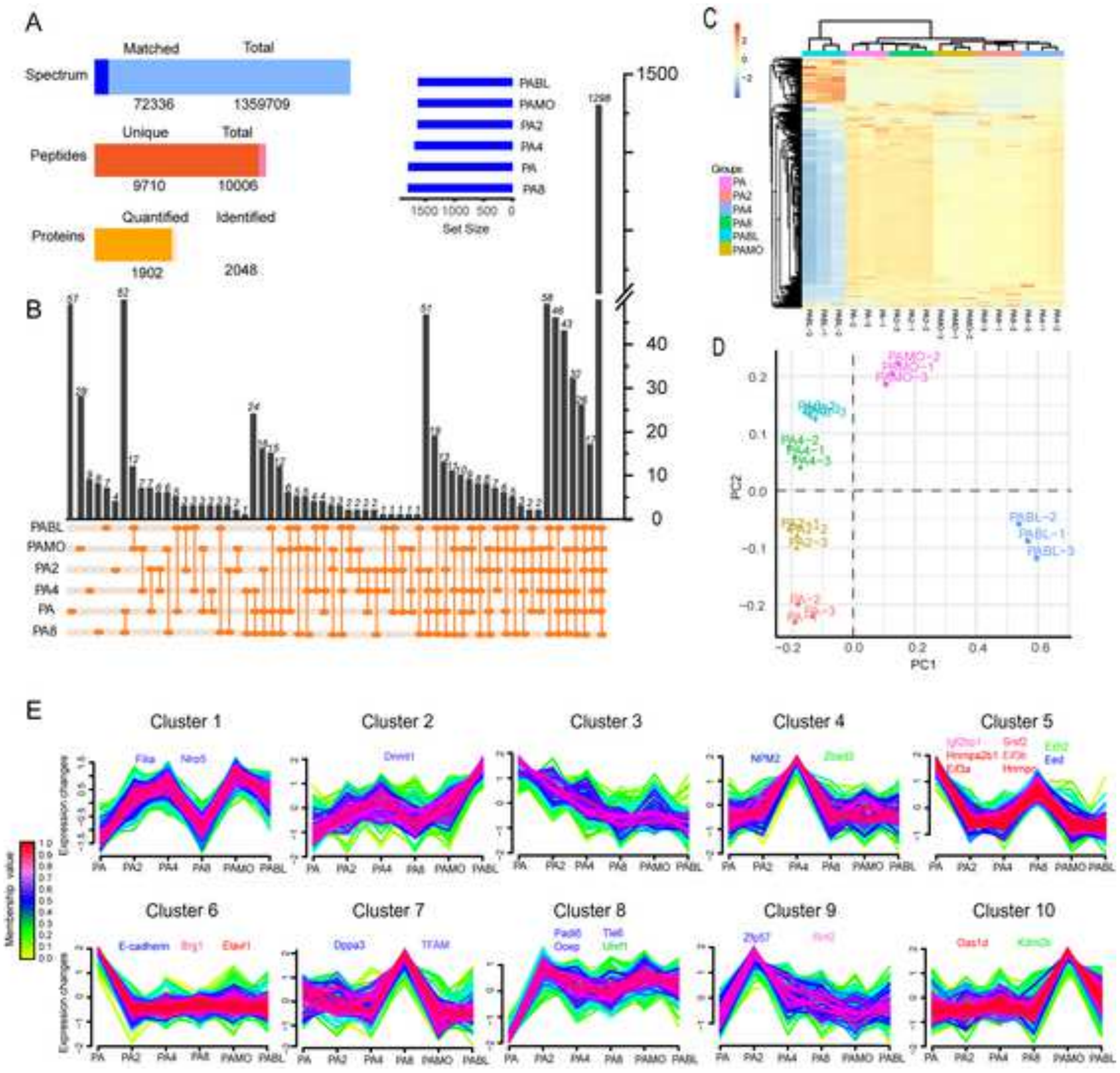

Figure.4

Click here to access/download;Figure;Figure.4.tif

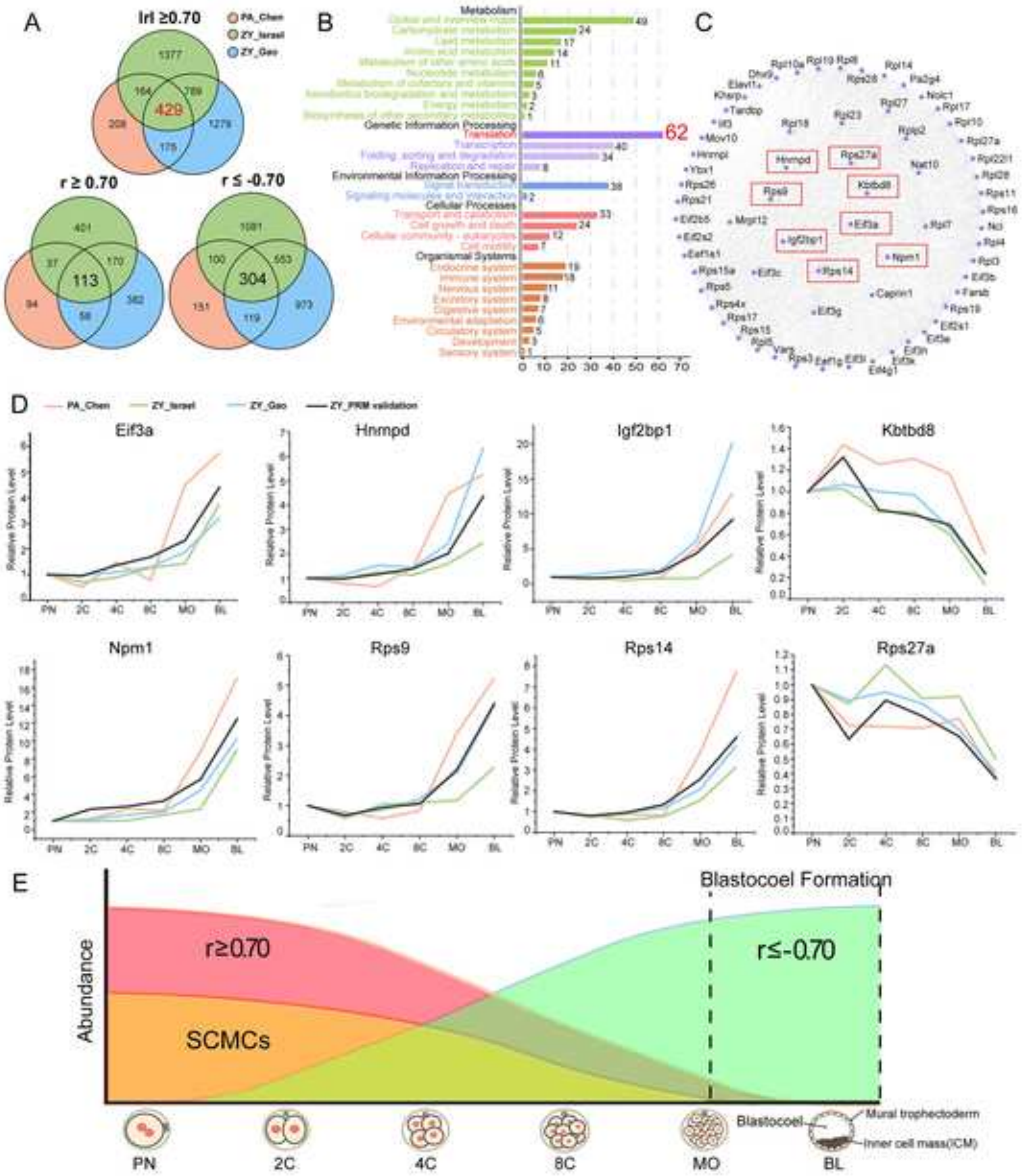

Figure.5

Click here to access/download;Figure;Figure.5.tif

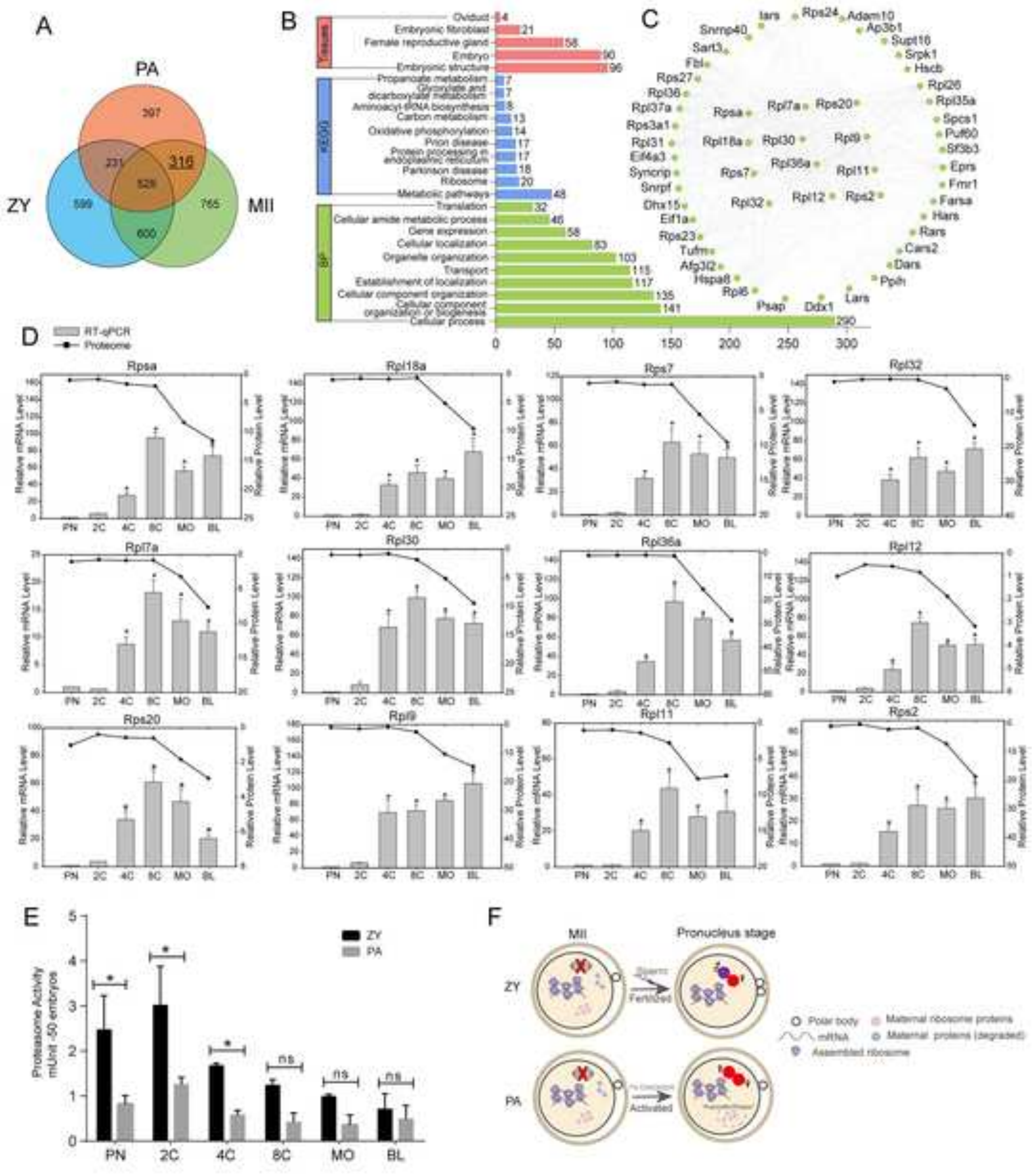

Figure.S5

Click here to access/download
**Supplementary Material**
Figure.S5.tif

Cover letter

Click here to access/download
**Supplementary Material**
Cover letter.docx

Figure.S1

Click here to access/download
**Supplementary Material**
Figure.S1.tif

Figure.S2

Click here to access/download
**Supplementary Material**
Figure.S2.tif

Figure.S3

Click here to access/download
**Supplementary Material**
Figure.S3.tif

Figure.S4

Click here to access/download
**Supplementary Material**
Figure.S4.tif

Click here to access/download
**Supplementary Material**
Table S1.xlsx

Table S2

Click here to access/download
**Supplementary Material**
Table S2.xlsx

Table S3

Click here to access/download
**Supplementary Material**
Table S3.xlsx

Table S4

Click here to access/download
**Supplementary Material**
Table S4.xlsx

Table S5

Click here to access/download
**Supplementary Material**
Table S5.xlsx

Table S6

Click here to access/download
**Supplementary Material**
Table S6.xlsx

Table S7

Click here to access/download
**Supplementary Material**
Table S7.xlsx

Table S8

Click here to access/download
**Supplementary Material**
Table S8.xlsx

Table S9

Click here to access/download
**Supplementary Material**
Table S9.xlsx

Click here to access/download
**Supplementary Material**
Table S10.xlsx

Table S11

Click here to access/download
**Supplementary Material**
Table S11.xlsx
