# Supplementary figures and images for: Comparative maternal protein profiling of mouse biparental and uniparental embryos
Source: Gigascience. 2022 Sep 3;11:giac084. doi: 10.1093/gigascience/giac084 (PMC9440387; doi:10.1093/gigascience/giac084)

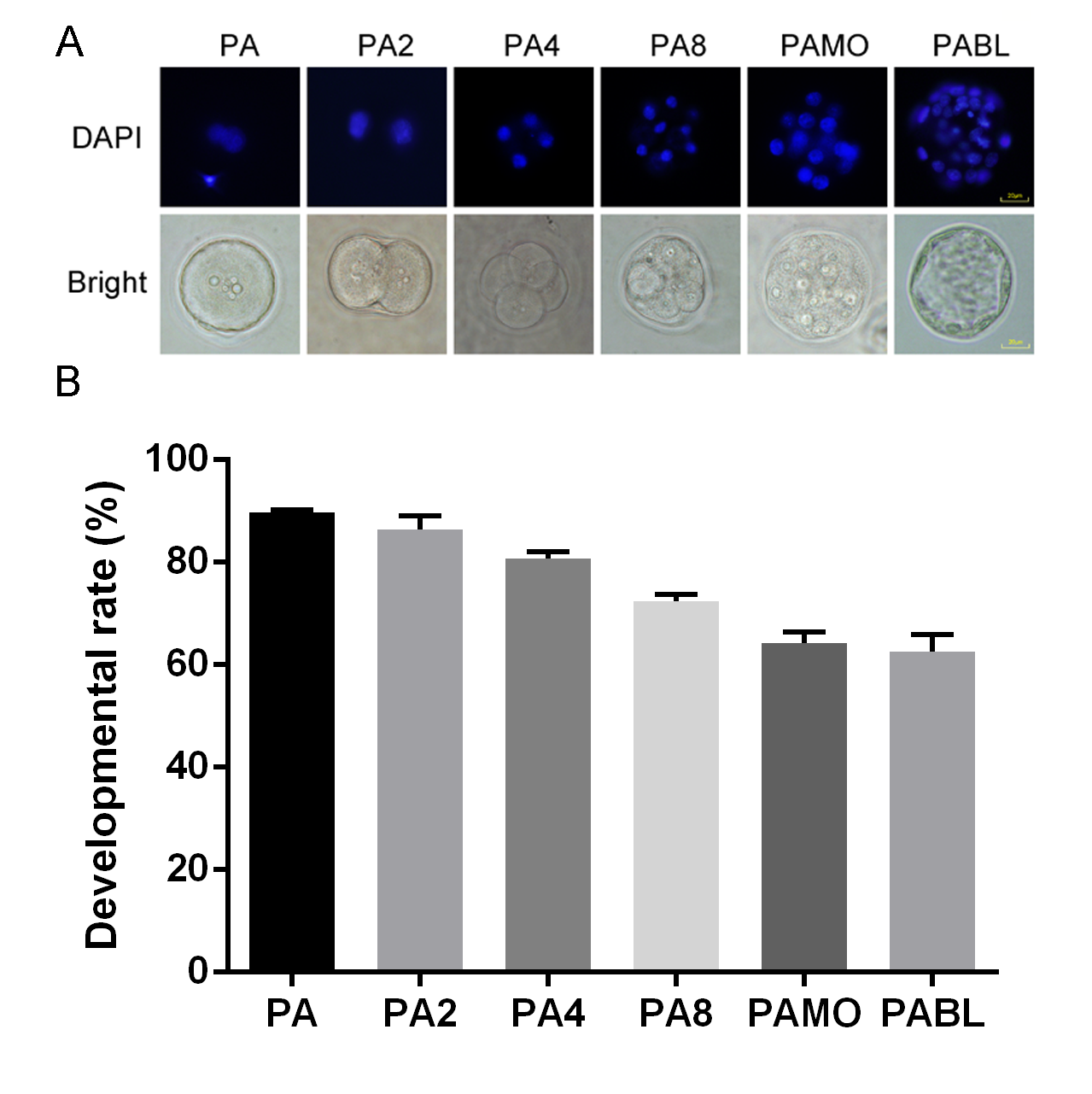

Supplement: giac084_Supplemental_Figures_and_Tables [file giac084_supplemental_figures_and_tables.zip › Figure.S1.tif]

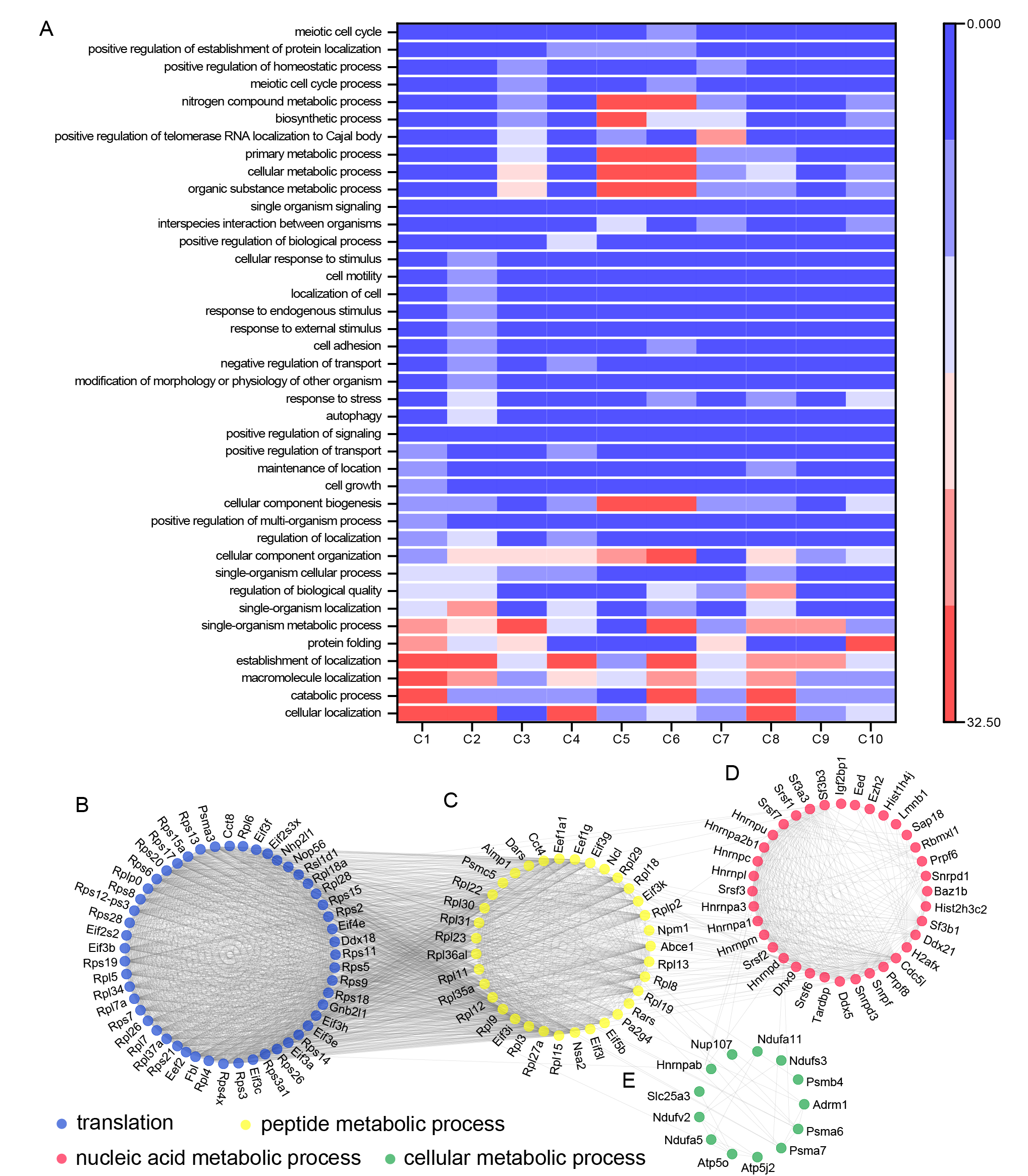

Supplement: giac084_Supplemental_Figures_and_Tables [file giac084_supplemental_figures_and_tables.zip › Figure.S2.tif]

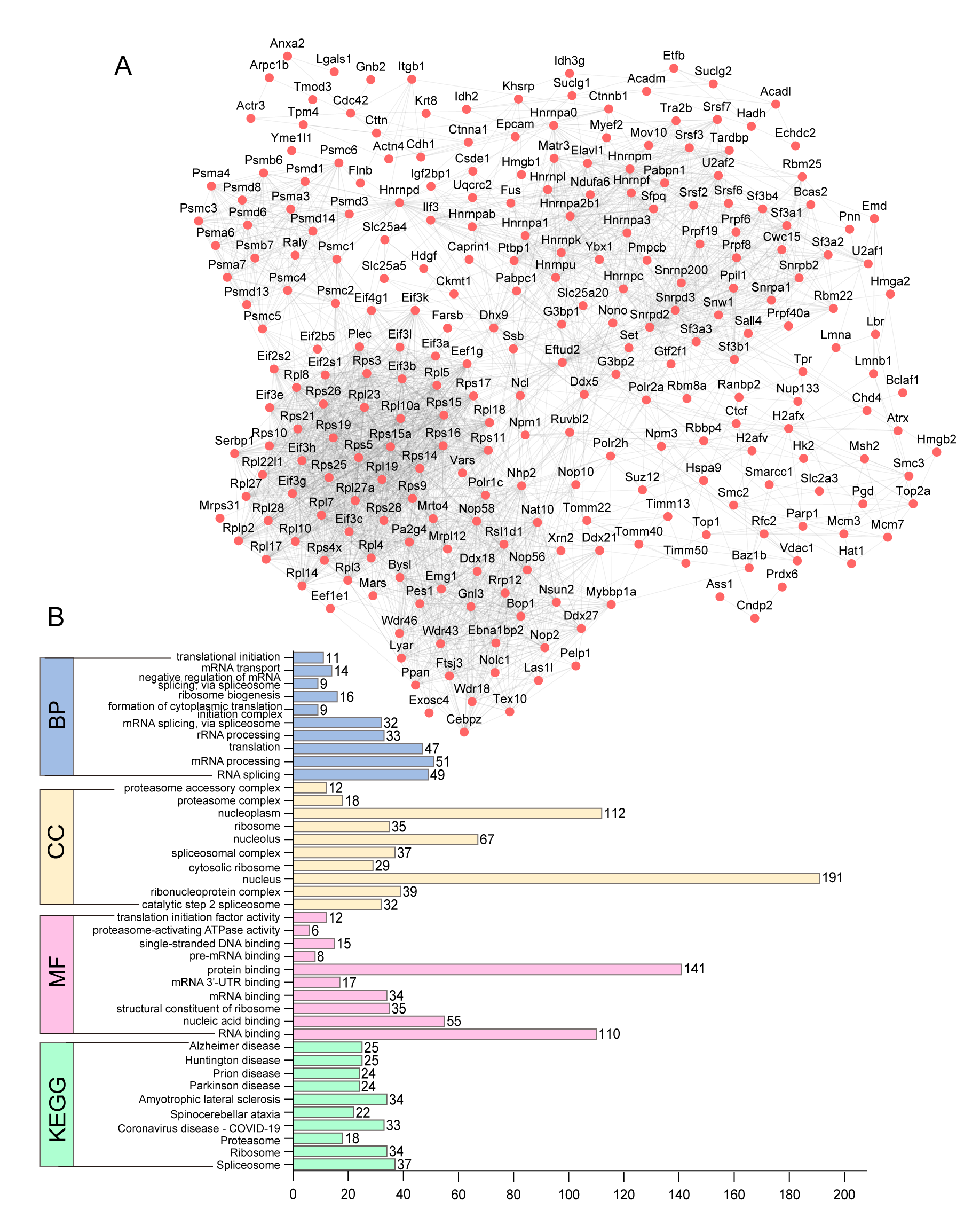

Supplement: giac084_Supplemental_Figures_and_Tables [file giac084_supplemental_figures_and_tables.zip › Figure.S3.tif]

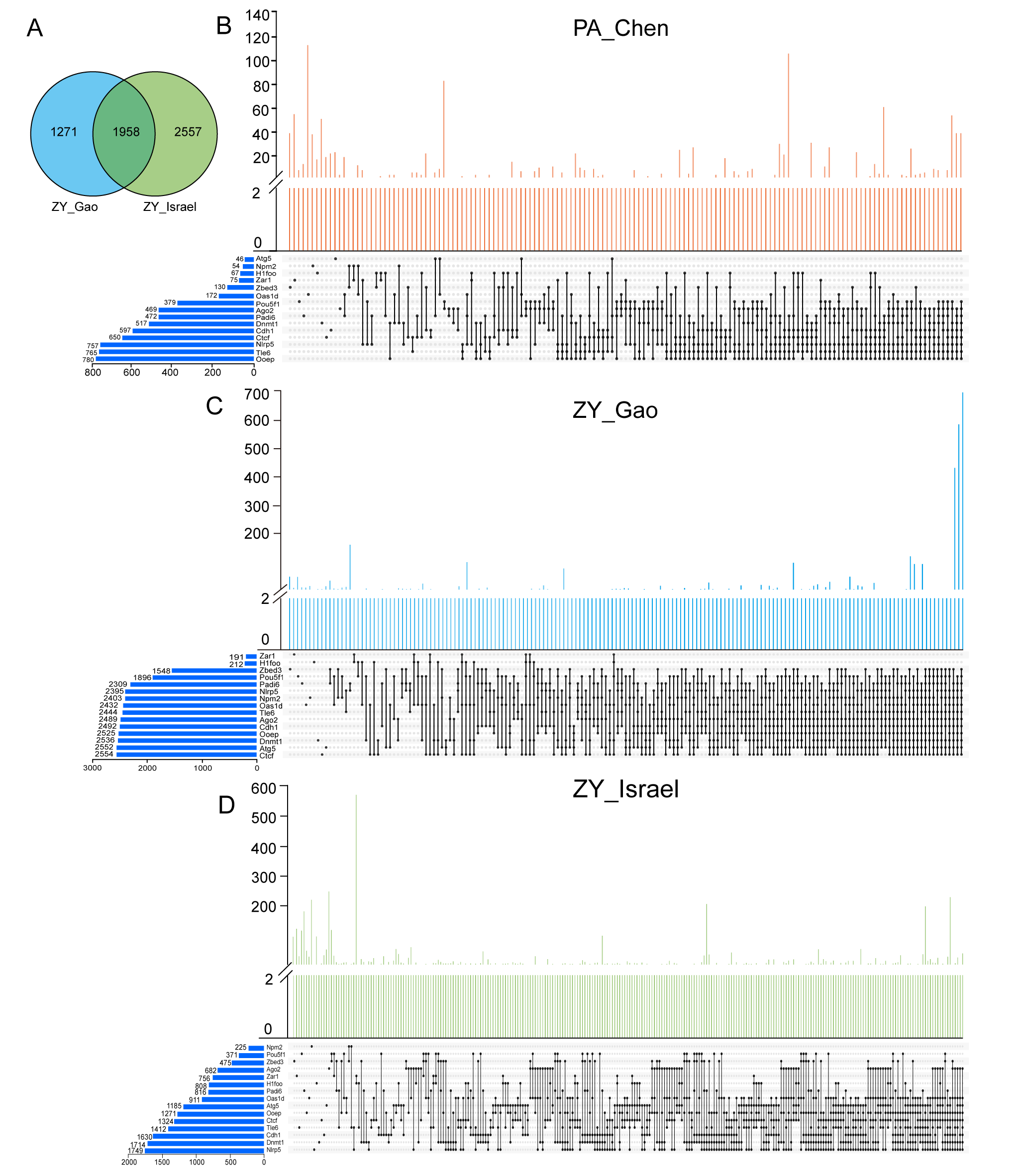

Supplement: giac084_Supplemental_Figures_and_Tables [file giac084_supplemental_figures_and_tables.zip › Figure.S4.tif]

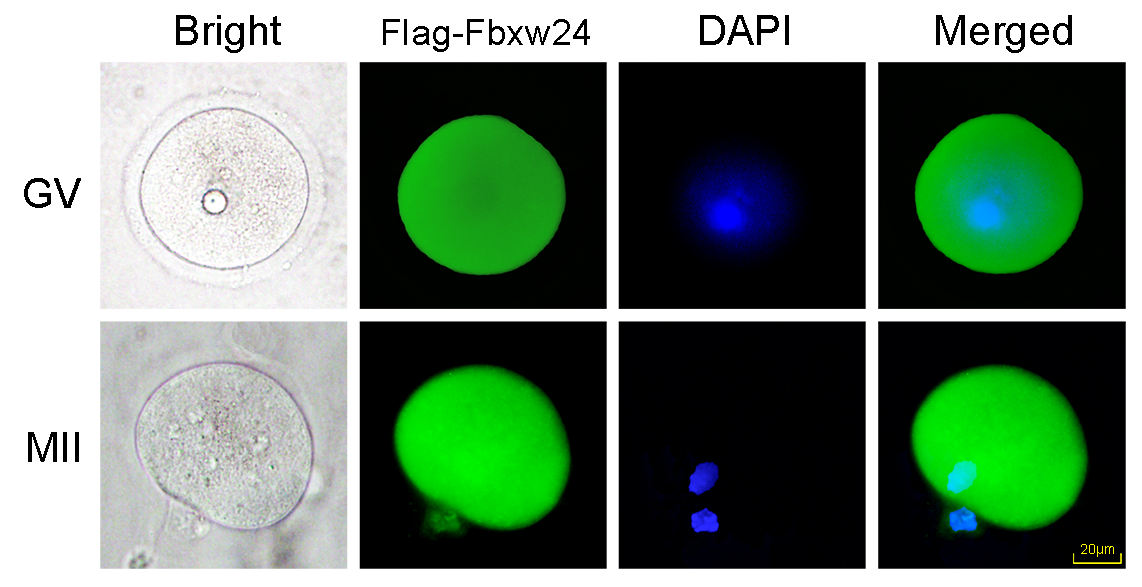

Supplement: giac084_Supplemental_Figures_and_Tables [file giac084_supplemental_figures_and_tables.zip › Figure.S5.tif]
