# Supplementary material for: Comparative maternal protein profiling of mouse biparental and uniparental embryos
Source: Gigascience. 2022 Sep 3;11:giac084. doi: 10.1093/gigascience/giac084 (PMC9440387; doi:10.1093/gigascience/giac084)
Supplement: giac084_Response_to_Reviewer_Comments_Original_Submission [file giac084_response_to_reviewer_comments_original_submission.pdf]

Author's Response To Reviewer Comments

Close

Dear Editor:

Thank you for your letter and all comments concerning our manuscript "Comparative maternal protein profiling of mouse biparental and uniparental embryos" (GIGA-D-22-00094), and they are highly valuable and helpful for improving our paper. We have studied these comments carefully and have made corrections which we hope meet with approval. For the detailed response, please see below.

Responds to the Reviewer #1:

In the revised version of the article, the authors largely corrected the article based on my previous comments. I am satisfied with the revised version.
Only a few minor errors remained to be corrected, which are given below.

1). Please write the phrase "et al." in an italic format in the following sites, Gao et al., Israel et al., etc.
Response: Thank you for your comments. The corresponding corrections have been made in the revised manuscript: Line 66, Line 71, Line 75, Line 565, Line 588, Line 589.

2). Please use full stop instead of comma in the following numbers 5,217; 1,709 etc.
Response: Thank you for your comments. The numbers, including 5,217; 1,709 etc ,
represents the amount of protein, not a decimal point, and we think this typing of number is common in many published documents.

3). The phrase "in vivo" should be written in an italic format.
Response: Thank you for your comments.The corresponding corrections have been made in the revised manuscript: Line 477, Line 578.

4). Please correct the "mice ovaries" into mouse ovaries.
Response: Thank you for your comments. The corresponding corrections have been made in the revised manuscript: Line 478, Line 817, Line 923.

5). Other typing errors should be corrected throughout the article.
Response: Thank you for your comments. We have checked and made corrections throughout the manuscript, and all details are presented in the revised manuscript.

6). Figures 1, 4, and 5 qualities should be increased to provide more easily reading the typing and images.
Response: Thank you for your comments. The resolution of Figures 1, 4, and 5 has been increased from 300dpi to 600dpi, and we think it may increased the qualities of these figures.

Responds to the Reviewer #2:

1). if the Authors say that Fbxw24 is a maternal factor, then they need to show that Fbxw24 is present in metaphase II oocytes prior to parthenogenetic activation or fertilization.
Response: Thank you for your comments. Ⅰ). Fbxw24 protein was detected by immunofluorescence assay using the oocytes of germinal vesicle(GV) stage and metaphase II (MII) stage that obtained from C57BL/6J-Fbxw24em(Linker-3xFlag) mice. The results show that Fbxw24 are detected in both GV and MII oocytes in protein level (as shown in the figure below). This result is supplemented in the revised manuscript as Figure S5 and its corresponding descriptions were also added in revised manuscript: Line 436-439, Line 642-643 and Line 1152-1155.

Ⅱ). The immunohistochemistry results also indicate that the Fbxw24 protein was located in oocytes (red arrows) and granulosa cells (red arrowheads) at different follicular stages (Fig. 6F) (Line 455-457 in the

revised manuscript)

Ⅲ). The Fbxw24 also detected in the protein database of MII oocytes reported by Israel et al., (PMID: 31527703, PMID:31638890).

2). If the proteomic dataset of this study does not include Fbxw24 (Table 1 and Supplementary Table 1), then this is somewhat a logical glitch
Response: Thank you for your comments. Ⅰ). Although Fbxw24 was not detected in the proteome of parthenogenetic embryos, the selection of fbxw24 is based on the comparison results of the two embryonic proteome (biparental/fertilized and uniparental/parthenogenetic embryos). During the analysis, a family of F-box/WD40 repeat-containing proteins (Fbxws) attracted our attention, and the selection of Fbxw24 for further analysis based on the following detailed reasons:
①We found that only Fbxw11 and Fbxw15 were detected in uniparental embryos(PA group, identified in this study), while Fbxw8, Fbxw11, Fbxw13, Fbxw15, Fbxw16, Fbxw18, Fbxw19, Fbxw20, Fbxw21, Fbxw22, Fbxw24, Fbxw26, Fbxw28 were detected in biparental embryos (ZY group, identified by Gao and Israel) (Table 1). Obviously, the quantity of Fbxws in biparental embryos is much more than uniparental embryos.
②Furthermore, in uniparental embryos, Fbxw11 was not correlated with the SCMC components, but in biparental embryos it had a strong negative correlation ($r \leq -0.70$, marked in green, Table 1) with the SCMC components; Also the expression correlation between Fbxw15 and the SCMC components is quite opposite in two embryos: Fbxw15 has strong negative correlations ($r \leq -0.70$, marked in green, Table 1) with the SCMC components in uniparental embryos, but strong positive correlations ($r \geq 0.70$, marked in red, Table 1) with the SCMC components in biparental embryos.
③Finally, Fbxw24 was selected for further analysis, because its correlation relationship with other Fbxws is similar to that of the SCMC components in biparental embryos (Table 1), which reveal it probably be of importance in normal embryonic development from zygote.
④To date, the role of Fbxw24 during early embryonic development remain unstudied. (The corresponding descriptions were presented in revised manuscript, Line 415-434).

Ⅱ). The selection of fbxw24 was based on the comparison results of the two embryonic proteome, and the parthenogenetic embryo proteome reported in this study is a critical reference. The finding that Fbxw24 may play a key role as a maternal factor in the development of preimplantation embryos is a successful case of using parthenogenetic embryos as a reference. It also shows that it may be valuable for the identification of key maternal factors by comparing the maternal protein files of parthenogenetic embryos and fertilized embryos.

3). How can it be claimed that it is a maternal factor, if it is not detected in oocytes? I understand that probably there is no valid antibody to reveal native Fbxw24 by immunofluorescence or by western blotting, however, the claim that Fbxw24 is a maternal factor demands evidence that Fbxw24 is present in metaphase II oocytes as protein. This is my major point. Maybe the Authors can 'borrow' the information from other studies, and/or expand the use of their FLAG system.
Response: Thank you for your comments. Ⅰ). Fbxw24 protein was detected by immunofluorescence assay using the oocytes of germinal vesicle(GV) stage and metaphase II (MII) stage that obtained from C57BL/6J-Fbxw24em(Linker-3xFlag) mice.
The results show that Fbxw24 are detected in both GV and MII oocytes in protein level(as shown in the figure below). This result is supplemented in the revised manuscript as Figure S5 and its corresponding descriptions were also added in revised manuscript: Line 436-439, Line 642-643 and Line 1152-1155.

Ⅱ). The immunohistochemistry results also indicate that the Fbxw24 protein was located in oocytes (red arrows) and granulosa cells (red arrowheads) at different follicular stages (Fig. 6F) (Line 455-457 in the revised manuscript)

Ⅲ). The Fbxw24 also detected in the protein database of MII oocytes reported by Israel et al., (PMID: 31527703, PMID:31638890).

4). Line 128: I understand what the Authors are saying; 'commonly' may be interpreted as meaning 'in common' that is, shared; I guess the Authors mean the union of the 1900, 1944 and 1960, not the intersection (in common). I suggest to check that the sentence is conveying the Authors' intended meaning.
Response: Thank you for your comments. As you understand, the 2,048 proteins mean the union of the

1900, 1944 and 1960 in deed. We have made the corresponding corrections: In three repetitions, 1,900, 1,944 and 1,960 proteins were detected respectively, and the number of their union was 2,048, of which 1,902 proteins were quantified (Line127-129 in the revised manuscript).

5). Line 408: "The expression and functional analysis of a new maternal factor". It appears that Fbxw24 was not detected in the parthenogenetic proteome. If so, then to name it 'maternal factor' does not seem justified. The Authors need to show that Fbxw24 is present as protein in metaphase II oocytes. If there is no suitable commercial antibody available against Fbxw24, would it then be possible to use the Flag system of Figure 6A to show expression in metaphase II oocytes? As alternative, would it be possible to infer the presence of Fbxw24 in MII oocytes from other studies?
Response: Thank you for your comments. Ⅰ). Fbxw24 protein was detected by immunofluorescence assay using the oocytes of germinal vesicle(GV) stage and metaphase II (MII) stage that obtained from C57BL/6J-Fbxw24em(Linker-3xFlag) mice.
The results show that Fbxw24 are detected in both GV and MII oocytes in protein level(as shown in the figure below). This result is supplemented in the revised manuscript as Figure S5 and its corresponding descriptions were also added in revised manuscript: Line 436-439, Line 642-643 and Line 1152-1155.

Ⅱ). The immunohistochemistry results also indicate that the Fbxw24 protein was located in oocytes (red arrows) and granulosa cells (red arrowheads) at different follicular stages (Fig. 6F) (Line 455-457 in the revised manuscript)

Ⅲ). The Fbxw24 also detected in the protein database of MII oocytes reported by Israel et al., (PMID: 31527703, PMID:31638890).

6). And would it be possible to add parthenogenetic embryos to Figure 6A?
Response: Thank you for your comments. In this study, during the comparison of the two embryonic proteome, the role of parthenogenetic embryos is as a reference and it is valuable for the identification of new key maternal factors. But parthenogenesis is not the common reproductive mode of mammals, and we think that the further functional research on a detailed maternal factor (such as Fbxw24) should mainly focus on fertilized embryos rather than parthenogenetic embryos.

7).Line 546: typo, preimplatation ('n' is missing)
Response: Many thanks for your careful check, and we have corrected it (Line 548 in the revised manuscript).

8). Line 634: 'mice were free to eat'; I suggest to say 'mice were fed ad libitum'.
Response: Thank you for your suggestions. We have made the corresponding corrections (Line 635 in the revised manuscript).

9). Line 444: I suggest to say 'transcript level' instead of 'transcriptional activity'
Response: Thank you for your suggestions. We have made the corresponding corrections (Line 446 in the revised manuscript).

Thank you again for your many professional suggestions, and they hold a very highly value for guiding our future studies.

We tried our best to improve the manuscript and made corresponding corrections in the revised manuscript. And we appreciate for Editors/Reviewers' warm work earnestly, and hope that the correction will meet with approval.

Once again, thank you very much for your comments and suggestions.

Best regards,

Dr. Fumei Chen
Department of Histology and Embryology, School of Pre-Clinical Medicine, Guangxi Medical University, Nanning, Guangxi, P. R. China
E-Mail: gxchenfumei@163.com

Close

Editorial Manager®

Cookie Preference Center

We use cookies which are necessary to make our site work. We may also use additional cookies to analyze, improve and personalize our content and your digital experience. For more information, see our Cookie Policy and the list of Google Ad-Tech Vendors.

You may choose not to allow some types of cookies. However, blocking some types may impact your experience of our site and the services we are able to offer. See the different category headings below to find out more or change your settings.

Allow all

Manage Consent Preferences

Strictly Necessary Cookies

Always active

These cookies are necessary for the website to function and cannot be switched off in our systems. They are usually only set in response to actions made by you which amount to a request for services, such as setting your privacy preferences, logging in or filling in forms. You can set your browser to block or alert you about these cookies, but some parts of the site will not then work. These cookies do not store any personally identifiable information.

Functional Cookies

Always active

These cookies enable the website to provide enhanced functionality and personalisation. They may be set by us or by third party providers whose services we have added to our pages. If you do not allow these cookies then some or all of these services may not function properly.

Performance Cookies

☑

These cookies allow us to count visits and traffic sources so we can measure and improve the performance of our site. They help us to know which pages are the most and least popular and see how visitors move around the site.

Targeting Cookies

These cookies may be set through our site by our advertising partners. They may be used by those companies to build a profile of your interests and show you relevant adverts on other sites. If you do not allow these cookies, you will experience less targeted advertising.

Back

Clear

Apply Cancel

Consent Leg.Interest

Confirm my choices
