# Supplementary material for: Comparative maternal protein profiling of mouse biparental and uniparental embryos
Source: Gigascience. 2022 Sep 3;11:giac084. doi: 10.1093/gigascience/giac084 (PMC9440387; doi:10.1093/gigascience/giac084)
Supplement: giac084_Reviewer_2_Report_Original_Submission — Michele Boiani -- 6/2/2022 Reviewed [file giac084_reviewer_2_report_original_submission.pdf]

Reviewer Report

Title: Comparative maternal protein profiling of mouse biparental and uniparental embryos

Version: Original Submission **Date:** 6/2/2022

Reviewer name: Michele Boiani

Reviewer Comments to Author:

I appreciate the considerable care and effort that that has been put into this revised version of the resource study by Fumei Chen and Colleagues (the Authors). The study aimed to compare parthenogenetic and fertilized embryos of the mouse, with an aim to identify those maternal proteins that undergo degradation after fertilization but escape degradation after parthenogenesis. I concur with the Authors on the relevance of this question for a more modern understanding of early embryonic processes. I appreciate, in particular, that the Authors named the limitations of their study in the Discussion. Every study has limitations, it is not a stigma to acknowledge limitations, on the contrary, it shows that the Authors are scientists with an open mind. I also appreciate the rebuttal letter / covering letter, which was very insightful; and I admit I learned from reading the covering letter. I sincerely thank the Authors.

I have some remarks and suggestions, listed below, and one main critique: if the Authors say that Fbxw24 is a maternal factor, then they need to show that Fbxw24 is present in metaphase II oocytes prior to parthenogenetic activation or fertilization. If the proteomic dataset of this study does not include Fbxw24 (Table 1 and Supplementary Table 1), then this is somewhat a logical glitch. How can it be claimed that it is a maternal factor, if it is not detected in oocytes? I understand that probably there is no valid antibody to reveal native Fbxw24 by immunofluorescence or by western blotting, however, the claim that Fbxw24 is a maternal factor demands evidence that Fbxw24 is present in metaphase II oocytes as protein. This is my major point. Maybe the Authors can 'borrow' the information from other studies, and/or expand the use of their FLAG system (Figure 6A).

Remarks and suggestions

Line 128: I understand what the Authors are saying; 'commonly' may be interpreted as meaning 'in common' that is, shared; I guess the Authors mean the union of the 1900, 1944 and 1960, not the intersection (in common). I suggest to check that the sentence is conveying the Authors' intended meaning.

Line 146: I appreciate that the Authors are now providing the developmental rates to blastocyst (Figure S1), which increase the confidence in the interpretation of the proteomic results. Well done.

The Results also contain some Discussion, but I do not hold this against the Authors. If the Journal is fine with mixing the Results and Discussion a bit, then I'm fine too.

Line 235: in the parthenotes the major change of proteome was observed at the morula stage. If the parthenogenetic and fertilized embryos are cultured under similar conditions, as the Authors wrote, then it means that regulation of gene expression differs between the two classes of embryos. This is a very important finding. I appreciate the explanations provided at line 299 and in Table 1. Well done.

Line 408: "The expression and functional analysis of a new maternal factor". It appears that Fbxw24 was

not detected in the parthenogenetic proteome. If so, then to name it 'maternal factor' does not seem justified. The Authors need to show that Fbxw24 is present as protein in metaphase II oocytes. If there is no suitable commercial antibody available against Fbxw24, would it then be possible to use the Flag system of Figure 6A to show expression in metaphase II oocytes? As alternative, would it be possible to infer the presence of Fbxw24 in MII oocytes from other studies? And would it be possible to add parthenogenetic embryos to Figure 6A? This is my major point about the revised version of the manuscript.

Line 546: typo, preimplatation ('n' is missing)

Line 579: Authors write: "We speculate that Fbxw24 may also be involved in the degradation of maternal proteins in early embryos." This is a very sensible and plausible hypothesis. I suggest the Authors could find additional support for this speculation, as follows. Please look at the studies on Btg4 e.g. the following BiorXiv preprint in which the respective Authors wrote: "we could see accumulation of Padi6, Zp2, Nobox, Nlrp14, Ubb, Fbxw24, Tle6, and Zar1 mRNA in Btg4 KD oocytes similar to that in Btg4 KO oocytes" (Liu et al., Poly(A) tail length is a major regulator of maternal gene expression during the mammalian oocyte-to-embryo transition). I suggest there might be a parallel between the case of Fbxw24 and that of Btg4. Just an idea, which the Authors are free to follow up or not.

Line 634: 'mice were free to eat'; I suggest to say 'mice were fed ad libitum'.

Line 444: I suggest to say 'transcript level' instead of 'transcriptional activity'

As a suggestion - dear Authors please feel free to take it or leave it: if the Authors study Fbxw24 in the mouse, I suggest to check the human ortholog and consider whether it could reveal something useful to add in Discussion.

As a suggestion - dear Authors please feel free to take it or leave it: perhaps the Authors could say more in regards to Fbxw24: I highlight the following excerpt from the Schultz et al. paper "The oocyte-to-embryo transition in mouse: past, present, and future", as it mentions Fbxw24: "Several E3 ubiquitin ligases are among the most abundant proteins in mouse oocytes, both at the transcript level [48] and at the protein level [53, 129], and some of them (Fbxw24, Rnf114, Rnf141, Smurf2, Cul1) are recruited during oocyte maturation."

As a suggestion - dear Authors please feel free to take it or leave it: the analysis of parthenogenetic embryos offers an opportunity to test some predictions related to the expression of imprinted protein-coding genes. Igf2 and Igf2r, for example, are reciprocally imprinted and expressed from the paternal and maternal allele, respectively. I note in the Authors' dataset (Table S1) that Igf2r protein is detected whereas Igf2 protein is not detected (sporadically). This matches the expectations. Therefore, I suggest this is a successful 'internal control' for the quality of the dataset, and the Authors could mention it.

As a suggestion - dear Authors please feel free to take it or leave it: I'd be more careful with the impact of the agent of parthenogenetic activation on gene expression. The Authors consider that the parthenogenetic proteome is the way it is mainly because the spermatozoon is missing and not so much because of the method of oocyte activation. I agree that the lack of sperm is the main factor, however, the Calcium waves can influence the gene expression. I recommend that the Authors be more cautious about the use of Ethanol as agent for oocyte activation, because even if Ethanol can be used, it does not mean it is a good idea to use it. Since parthenogenetic blastocyst do not develop to term (unless genetically modified or via the production of chimeras with fertilized embryos), it is hard to separate the contribution of imprinting imbalance and Calcium waves on gene expression. After ROSI, however, it

was apparent that $SrCl_2$ was superior to Ethanol in terms of full developmental rates (PMID 16452719). To me it means that $SrCl_2$ supports a more physiologic activation of the oocyte, compared to Ethanol. It is likely that this difference is mediated by the profile of gene expression. Therefore, as I said, I'd be more careful with the impact of the agent of parthenogenetic activation on gene expression.
In conclusion, I welcome the revision of the manuscript and I support its acceptance for publication, pending a few small adjustments as listed above.
Thank you for this thorough and very good revision, and best of luck also for your future studies.
Michele Boiani

Methods

Are the methods appropriate to the aims of the study, are they well described, and are necessary controls included? Choose an item.

Conclusions

Are the conclusions adequately supported by the data shown? Choose an item.

Reporting Standards

Does the manuscript adhere to the journal's guidelines on [minimum standards of reporting?] Choose an item.

Choose an item.

Statistics

Are you able to assess all statistics in the manuscript, including the appropriateness of statistical tests used? Choose an item.

Quality of Written English

Please indicate the quality of language in the manuscript: Choose an item.

Declaration of Competing Interests

Please complete a declaration of competing interests, considering the following questions:

- Have you in the past five years received reimbursements, fees, funding, or salary from an organisation that may in any way gain or lose financially from the publication of this manuscript, either now or in the future?
- Do you hold any stocks or shares in an organisation that may in any way gain or lose financially from the publication of this manuscript, either now or in the future?

- Do you hold or are you currently applying for any patents relating to the content of the manuscript?
- Have you received reimbursements, fees, funding, or salary from an organization that holds or has applied for patents relating to the content of the manuscript?
- Do you have any other financial competing interests?
- Do you have any non-financial competing interests in relation to this paper?

If you can answer no to all of the above, write 'I declare that I have no competing interests' below. If your reply is yes to any, please give details below.

I declare that I have no competing interests

I agree to the open peer review policy of the journal. I understand that my name will be included on my report to the authors and, if the manuscript is accepted for publication, my named report including any attachments I upload will be posted on the website along with the authors' responses. I agree for my report to be made available under an Open Access Creative Commons CC-BY license (http://creativecommons.org/licenses/by/4.0/). I understand that any comments which I do not wish to be included in my named report can be included as confidential comments to the editors, which will not be published.

Choose an item.

To further support our reviewers, we have joined with Publons, where you can gain additional credit to further highlight your hard work (see: https://publons.com/journal/530/gigascience). On publication of this paper, your review will be automatically added to Publons, you can then choose whether or not to claim your Publons credit. I understand this statement.

Yes Choose an item.
